# Supplementary material for: Practical access to axially chiral sulfonamides and biaryl amino phenols via organocatalytic atroposelective N-alkylation
Source: Nat Commun. 2019 Jul 11;10:3061. doi: 10.1038/s41467-019-10940-4 (PMC6624369; doi:10.1038/s41467-019-10940-4)
Supplement: Supplementary file 1 — Supplementary Information [file 41467_2019_10940_MOESM1_ESM.pdf]

Practical Access to Axially Chiral Sulfonamides and Biaryl Amino Phenols *via* Organocatalytic Atroposelective *N*-Alkylation

Lu et al.

Supplementary Methods

General information. ^{1}**H** and ^{13}**C NMR** spectra were recorded on a Bruker AMX500 (500 MHz) spectrometer. Chemical shifts were reported in parts per million (ppm), and the residual solvent peak was used as an internal reference: ^{1}H (chloroform δ 7.26; DMSO δ 2.50; acetone-d_6 δ 2.05), ^{13}C (chloroform δ 77.0; DMSO δ 39.5; acetone-d_6 δ 205.87, 30.60). Data are reported as follows: chemical shift, multiplicity (s = singlet, d = doublet, t = triplet, q = quartet, m = multiplet, br = broad), coupling constants (Hz) and integration. Melting point (**MP**) was obtained on Buchi B-540. For thin layer chromatography (**TLC**), Merck pre-coated TLC plates (Merck 60 F254) were used, and compounds were visualized with a UV light at 254nm. High resolution mass spectra (**HRMS**) were obtained on a Finnigan/MAT 95XL-T spectrometer. **Optical rotations** were recorded on an mrc AP81 automatic polarimeter. Enantiomeric excesses (**ee**) were determined by HPLC analysis on Agilent HPLC units, including the following instruments: pump, LC-20AD; detector, SPD-20A; column, Chiralcel OD-H, Chiralpak AD-H, AS-H, ID or IE.

All commercially available reagents listed below were used as received for the reactions without any purification. 3,5-dimethylaniline, 2-naphthol, 3-Bromo-2-naphthol, 6-Bromo-2-naphthol, 7-Bromo-2-naphthol, 7-Methoxy-2-naphthol, 6-Hydroxy-2-naphthoic acid, 9-Phenanthrol, 3,5-Dibromophenol and 4-Bromo-3,5-dimethylphenol were purchased from Sigma aldrich. Liquid reagents were handled with a micropipette. THF was dried on alumina columns using a solvent dispensing system.

For the kinetic resolution, selectivity factors (S) were calculated according to Kagan's equation: $S = \ln((1\text{-}c)(1\text{-}ee_{rsm}))/\ln((1\text{-}c)(1+ee_{rsm}))$, wherein c is conversion of the reaction, ee_{rsm} is the enantiomeric excess of the recovered starting material. Conversions (Conv.) were calculated by ^{1}H NMR.

The MBH cabonate **2a** and **2b** were synthesized in one step from commercially available materials by literature methods.[1]

Substrates syntheses:

Synthesis of 1a-1k:

These substrates were synthesized following our previous method.[2]

Synthesis of 1l:

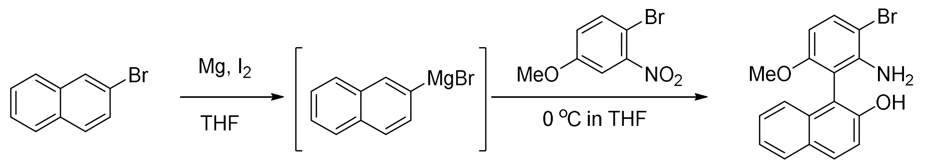

Our previous one-pot method was followed to yield this intermediate in 52% yield as a brown solid.[3] **MP**: 155 - 156 °C. **^{1}H NMR** (400 MHz, $CDCl_3$): δ 7.93 – 7.84 (m, 2H), 7.56 (dd, *J* = 8.9, 2.3 Hz, 1H), 7.42 – 7.32 (m, 4H), 6.45 (dd, *J* = 8.9, 2.4 Hz, 1H), 5.25 (s, 1H), 3.98 (s, 2H), 3.67 (s, 3H). **^{13}C NMR** (100MHz, $CDCl_3$): δ 158.37 , 151.46 , 144.25 , 133.40 , 132.57 , 130.40 , 129.37 , 128.33 , 126.90 , 124.11 , 123.60 , 117.78 , 112.92 , 106.77 , 102.25 , 101.38 , 55.98. **HRMS (ESI)** m/z Calcd for $[C_{17}H_{14}BrNNaO_2, M + Na]^+$: 366.0100; Found: 366.0104.

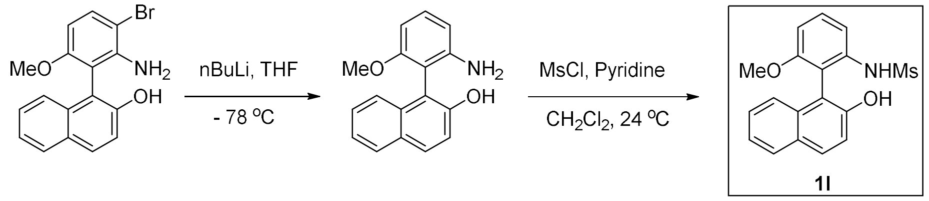

A solution of amino alcohol (1 mmol) in anhydrous THF (5 mL) was cooled to -78 °C. n-Butyllithium (6 mmol, 2 M in cyclohexane) was added slowly to the reaction flask and stirred for 1 h at -78 °C. The reaction mixture was then left to stir at room temperature overnight. Reaction quenched with NH_4Cl and extracted with ethyl acetate. Combined organic layers washed with brine and dried with Na_2SO_4 and evaporated *in vacuo*. Crude residue was purified by column chromatography (81% yield). Off-white solid. **MP**: 180 - 181 °C. **^{1}H NMR** (400 MHz, $CDCl_3$): δ 7.77 – 7.67 (m, 2H), 7.28 – 7.14 (m, 5H), 6.42 – 6.38 (m, 2H), 5.27 (s, 1H), 3.54 (s, 3H), 3.39 (s, 2H). **^{13}C NMR** (101 MHz, $CDCl_3$) δ 159.16 , 151.49 , 146.96 , 132.95 , 130.65 , 129.94 , 129.34 , 128.25 , 126.61 , 124.42 , 123.39 , 117.76 , 113.32 , 108.73 , 106.15 , 101.26 , 55.77 . **HRMS (ESI)** m/z Calcd for $[C_{17}H_{16}NO_2, M + H]^+$: 266.1176; Found 266.1167.

A solution of amino alcohol (0.6 mmol) in anhydrous CH_2Cl_2 (15 mL) was cooled to 0 °C. Pyridine (0.9 mmol) and MsCl (0.6 mmol) were added successively to the reaction flask. The reaction mixture was then left to stir at room temperature overnight. The solvent was evaporated *in vacuo*. Crude residue was purified by column chromatography (62% yield). White solid. **MP:** 214 - 215 °C. **^{1}H NMR** (400 MHz, Acetone-d_6): δ 8.52 (br, 1H), 7.91 – 7.86 (m, 2H), 7.49 (t, *J* = 8.2 Hz, 1H), 7.42 (dd, *J* = 8.3, 1.1 Hz, 1H), 7.40 – 7.30 (m, 3H), 7.21 (ddd, *J* = 7.4, 2.2, 0.9 Hz, 1H), 7.01 (dd, *J* = 8.2, 1.2 Hz, 1H), 6.61 (br, 1H), 3.65 (s, 3H), 2.79 (s, 3H). **^{13}C NMR** (100MHz, Acetone-d_6): δ 158.77 , 152.72 , 137.80 , 133.81 , 130.31 , 129.79 , 129.07 , 128.23 , 126.70 , 123.81 , 123.19 , 118.47 , 115.69 , 112.46 , 112.29 , 107.51 , 55.22 , 38.88 . **HRMS (ESI)** m/z Calcd for $[C_{18}H_{16}NO_4S, M - H]^-$: 342.0806; Found: 342.0808.

Synthesis of 1m-1o:

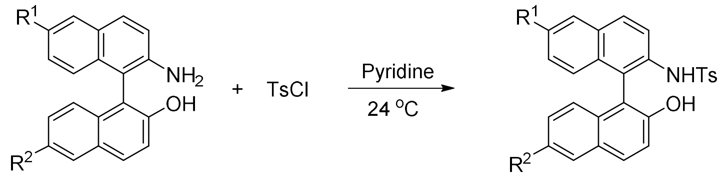

The NOBIN analogs were synthesized by the known procedures.[4,5]

A solution of NOBIN analog (0.6 mmol) in Pyridine (1mL), TsCl (0.6 mmol) were added to the reaction flask. The reaction mixture was then left to stir at room temperature overnight. The solvent was evaporated *in vacuo*. Crude residue was purified by column chromatography.

Synthesis of 1p:

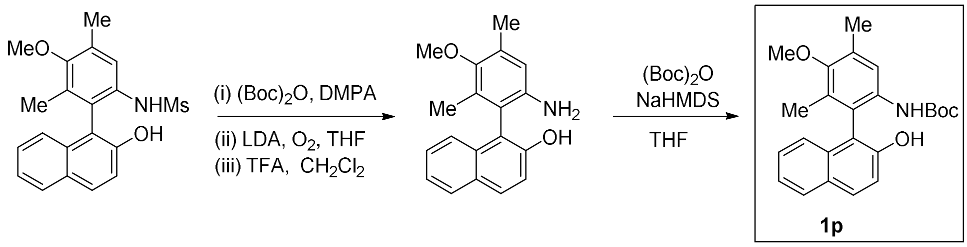

The first step followed our previous procedure to deliver the free amino phenol intermediate.[2]

To a solution of the amino phenol (1 mmol) in THF at -78 °C was added a solution of NaHMDS (3.3 equiv) in THF (5 mL) via syringe. The reaction mixture was warmed to room temperature and stirred for 30 minutes. The reaction was then recooled to -78 °C and a solution of $(Boc)_2O$ (1 equiv) in THF (5 mL) was added dropwise to the reaction mixture. The reaction was allowed to stir at -78 °C for 2 hours, then gradually warm to room temperature. The reaction was then heated to 65 °C and allowed to stir overnight. The reaction was quenched by the addition of 20 mL of water and 2 M HCl was added gradually till pH = 3. The reaction mixture was extracted with ethyl acetate (3 X 15 mL), the combined organic phase was washed with brine, dried over Na_2SO_4, filtered and concentrated *in vacuo*. The crude residue was purified by column to yield the desired compounds (82% yield) as a white solid. **MP**: 197 – 198 °C. **^{1}H NMR** (500 MHz, $CDCl_3$): δ 7.92 – 7.86 (m, 3H), 7.38 (qdd, *J* = 6.8, 4.1, 1.8 Hz, 2H), 7.33 (dd, *J* = 8.9, 1.5 Hz, 1H), 7.21 – 7.15 (m, 1H), 5.91 (s, 1H), 5.30 (br, 1H), 3.77 (s, 3H), 2.44 (s, 3H), 1.85 (s, 3H), 1.36 (s, 9H). **^{13}C NMR** (125 MHz, $CDCl_3$): δ 153.61 , 153.43 , 151.23 , 133.55 , 132.76 , 132.59 , 132.11 , 130.54 , 129.28 , 128.33 , 127.21 , 123.80 , 123.75 , 121.57 , 121.13 , 117.79 , 114.76 , 80.52 , 60.13 , 28.16 , 16.59 , 13.12 . **HRMS (ESI)** m/z Calcd for $[C_{24}H_{26}NO_4, M - H]^-$: 392.1868; Found: 392.1866.

Synthesis of 1q:

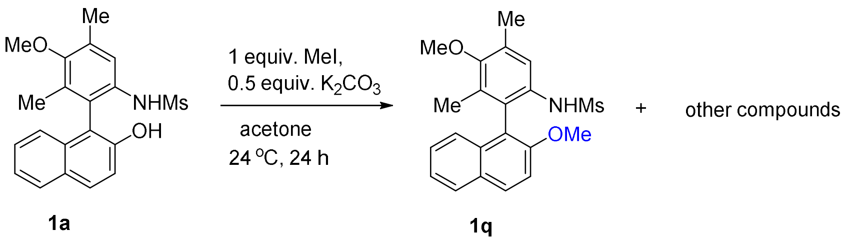

To a solution of **1a** (0.4 mmol) in acetone (4 mL) were added K_2CO_3 (0.2 mmol) and MeI (0.4 mmol). The reaction mixture was allowed to stir at room temperature overnight. The solvent was evaporated *in vacuo*. The crude residue was purified by column chromatography to produce the desired product **1q** (25% yield) as a syrup. **^{1}H NMR** (400 MHz, CD_2Cl_2): δ 7.93 (dd, J = 9.1, 0.8 Hz, 1H), 7.84 – 7.78 (m, 1H), 7.36 (d, J = 9.1 Hz, 1H), 7.34 – 7.32 (m, 1H), 7.32 – 7.25 (m, 2H), 7.10 – 7.05 (m, 1H), 5.64 (br, 1H), 3.79 (s, 3H), 3.67 (s, 3H), 2.49 (s, 3H), 2.32 (s, 3H), 1.70 (s, 3H). **^{13}C NMR** (75 MHz, CD_2Cl_2): δ 154.66 , 154.10 , 132.73 , 131.70 , 131.40 , 131.05 , 130.73 , 129.22 , 128.35 , 127.42 , 126.93 , 124.00 , 123.51 , 121.22 , 117.39 , 113.08 , 59.76 , 56.05 , 39.03 , 16.09 , 13.05 . **HRMS (ESI)** m/z Calcd for $[C_{21}H_{23}NNaO_4S, M + Na]^+$: 408.1240; Found: 408.1242.

Attempt of kinetic resolution of 1a catalyzed by NHC:

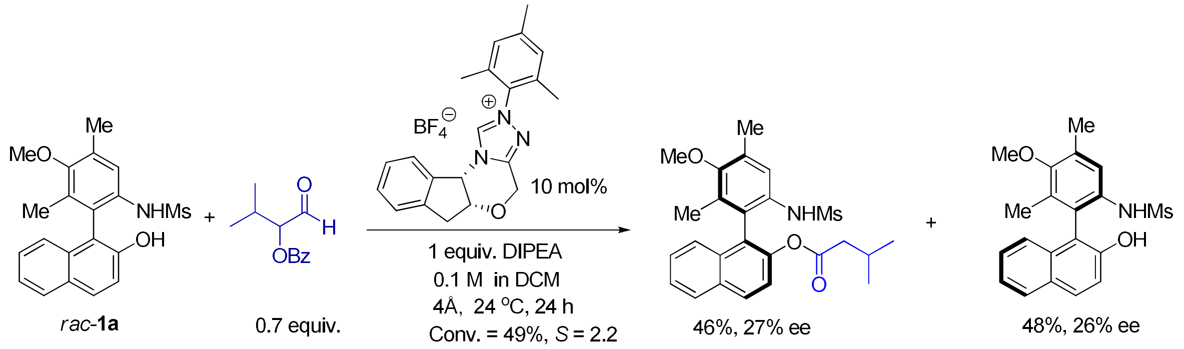

To a 4 mL vial was added the racemic **1a** (0.075 mmol), triazolium salt (0.0075 mmol) and 4Å MS (25 mg). The mixture was taken into the glovebox, where aldehyde (9 µL, 0.052 mmol), anhydrous DCM (0.75 mL) and DIPEA (18 µL, 0.075 mmol) were added. The reaction mixture was taken outside the glovebox. The vial was then sealed and the reaction mixture was allowed to stir at ambient temperature for 24 h. The crude reaction mixture was directly purified by silica gel column chromatography with hexanes/ethyl acetate (10:1 v/v) as eluent to afford the ester product and the recovered starting material in pure form.

Recovered 1a:

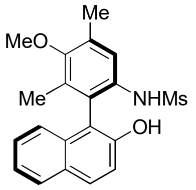

Syrup. **^{1}H NMR** (500 MHz, $CDCl_3$) δ 7.92 – 7.87 (m, 2H), 7.53 (s, 1H), 7.43 – 7.37 (m, 2H), 7.29 (d, J = 8.9 Hz, 1H), 7.14 – 7.12 (m, 1H), 5.82 (s, 1H), 5.29 (s, 1H), 3.80 (s, 3H), 2.73 (s, 3H), 2.44 (s, 3H),

1.91 (s, 3H). **^{13}C NMR** (125 MHz, $CDCl_3$) δ 155.01 , 150.98 , 133.24 , 132.98 , 132.43 , 132.00 , 131.17 , 129.40 , 128.77 , 127.67 , 124.19 , 123.64 , 123.15 , 121.52 , 117.78 , 114.09 , 60.15 , 39.59 , 16.58 , 13.34 . **HRMS (ESI)** m/z Calcd for $[C_{20}H_{20}NO_4S, M - H]^-$: 370.1119; Found: 370.1119.

Optical Rotation: $[\alpha]^{25}_{D}$ + 4.0 (*c* = 0.2, $CHCl_3$). 26% ee (HPLC condition: Chiralcel AD-H column, *n*-Hexane/*i*-PrOH = 90:10, flow rate = 1.0 mL/min, wavelength = 254 nm, t_R = 10.02 min for major isomer, t_R = 12.42 min for minor isomer).

Supplementary Figure 1. HPLC Trace of **1a** from NHC-Catalyzed Kinetic Resolution.

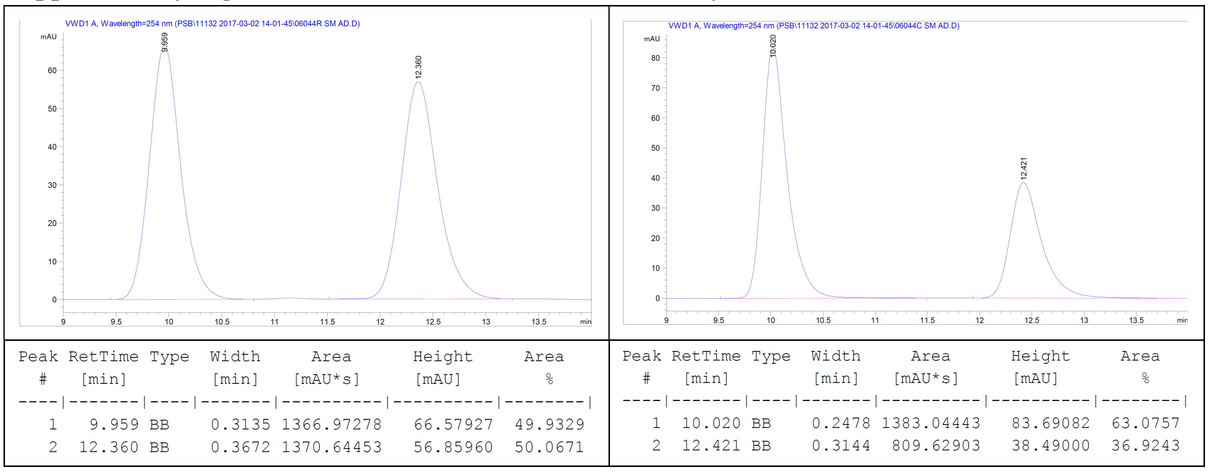

Peak #	RetTime [min]	Type	Width [min]	Area [mAU*s]	Height [mAU]	Area %
1	9.959	BB	0.3135	1366.97278	66.57927	49.9329
2	12.360	BB	0.3672	1370.64453	56.85960	50.0671

Peak #	RetTime [min]	Type	Width [min]	Area [mAU*s]	Height [mAU]	Area %
1	10.020	BB	0.2478	1383.04443	83.69082	63.0757
2	12.421	BB	0.3144	809.62903	38.49000	36.9243

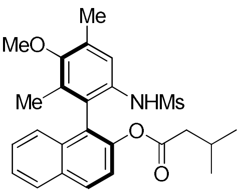

Syrup. **^{1}H NMR** (500 MHz, $CDCl_3$) δ 8.03 – 8.02 (m, 1H), 7.99 – 7.95 (m, 1H), 7.57 – 7.42 (m, 3H), 7.36 – 7.30 (m, 2H), 6.06 (s, 1H), 3.76 (s, 3H), 2.66 (s, 3H), 2.42 (s, 3H), 2.24 – 2.21 (m, 2H), 2.00 – 1.88 (m, 1H), 1.87 (s, 3H), 0.81 (d, *J* = 3.5 Hz, 3H), 0.79 (d, *J* = 3.5 Hz, 3H). **^{13}C NMR** (125 MHz, $CDCl_3$) δ 171.53, 154.26, 146.53 , 132.24 , 132.12 , 132.04 , 131.95 , 131.33 , 130.53 , 128.50 , 127.41 , 126.24 , 124.94 , 124.88 , 124.80 , 121.75 , 120.61 , 59.87 , 42.95 , 39.56 , 25.63 , 21.95, 21.91 , 16.33 , 13.25. **HRMS (ESI)** m/z Calcd for $[C_{25}H_{29}NNaO_5S, M + Na]^+$: 478.1658; Found: 478.1654.

Optical Rotation: $[\alpha]^{25}_{D}$ - 8.0 (*c* = 1.0, CH_2Cl_2). 27% ee (HPLC condition: Chiralcel ID column, *n*-Hexane/*i*-PrOH = 95:5, flow rate = 1.0 mL/min, wavelength = 254 nm, t_R = 31.59 min for major isomer, t_R = 37.63 min for minor isomer).

Supplementary Figure 2. HPLC Trace of Acylation Product from NHC-Catalyzed Kinetic Resolution.

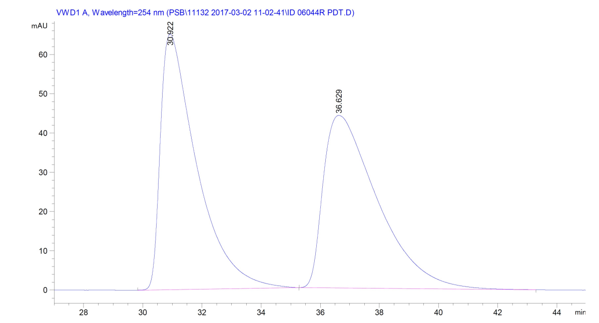

Peak #	RetTime [min]	Type	Width [min]	Area [mAU*s]	Height [mAU]	Area %
1	30.922	BB	1.2275	5551.97949	65.06358	49.7668
2	36.629	BB	1.8058	5604.01611	43.94561	50.2332

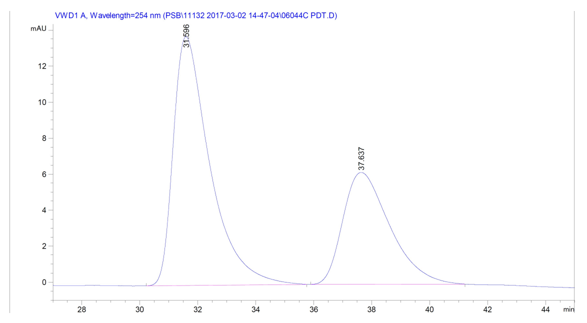

Peak #	RetTime [min]	Type	Width [min]	Area [mAU*s]	Height [mAU]	Area %
1	31.596	BB	1.2359	1213.57764	13.82730	63.4266
2	37.637	BB	1.3437	699.78015	6.21472	36.5734

Representative procedure for the kinetic resolution of 1 catalyzed by (DHQD)$_2$AQN:

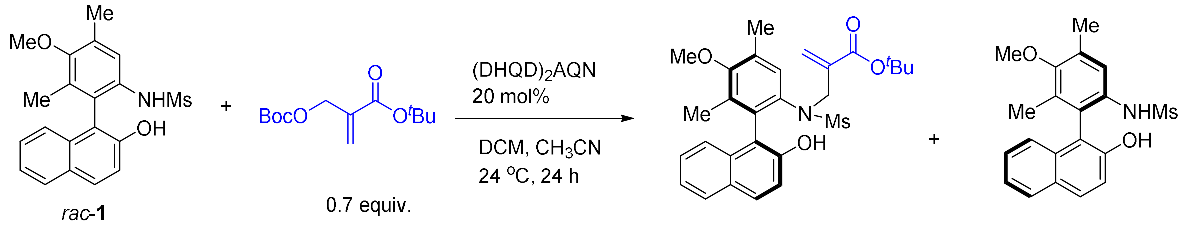

To a 4 mL vial containing **1** (0.04 mmol) and (DHQD)$_2$AQN (7.0 mg, 20 mol%) were added CH_2Cl_2 (0.5 mL), CH_3CN (0.5 mL) and MBH carbonate (6 μL). The reaction mixture was allowed to stir for 24 h at 24 °C. The volatiles were removed *in vacuo* at 24 °C and the residue was purified by silica gel column chromatography with hexanes/ethyl acetate (10:1 v/v) as the eluent to afford the product **4** and unreacted starting material **1**.

For the preparation of authentic racemic products, DABCO was used instead of (DHQD)$_2$AQN following the same procedure.

Note: NMR for the products were taken at 80 °C in DMSO-d$_6$.

Characterization of compounds

Recovered 1a

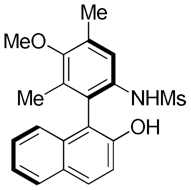

Syrup. ^{1}**H NMR** (500 MHz, $CDCl_3$) δ 7.92 – 7.87 (m, 2H), 7.53 (s, 1H), 7.43 – 7.37 (m, 2H), 7.29 (d, J = 8.9 Hz, 1H), 7.14 – 7.12 (m, 1H), 5.82 (s, 1H), 5.29 (s, 1H), 3.80 (s, 3H), 2.73 (s, 3H), 2.44 (s, 3H), 1.91 (s, 3H). ^{13}**C NMR** (125 MHz, $CDCl_3$) δ 155.01 , 150.98 , 133.24 , 132.98 , 132.43 , 132.00 , 131.17 , 129.40 , 128.77 , 127.67 , 124.19 , 123.64 , 123.15 , 121.52 , 117.78 , 114.09 , 60.15 , 39.59 , 16.58 , 13.34 . **HRMS (ESI)** m/z Calcd for $[C_{20}H_{20}NO_4S, M - H]^-$: 370.1119; Found: 370.1119.

Optical Rotation: $[\alpha]^{25}_D$ + 12.0 (c = 0.2, $CHCl_3$). The absolute configuration of **1a** was assigned by analogy to **1b**. 98% ee (HPLC condition: Chiralcel AD-H column, n-Hexane/i-PrOH = 90:10, flow rate = 1.0 mL/min, wavelength = 254 nm, t_R = 8.60 min for major isomer, t_R = 10.66 min for minor isomer).

Supplementary Figure 3. HPLC Trace of Recovered **1a**.

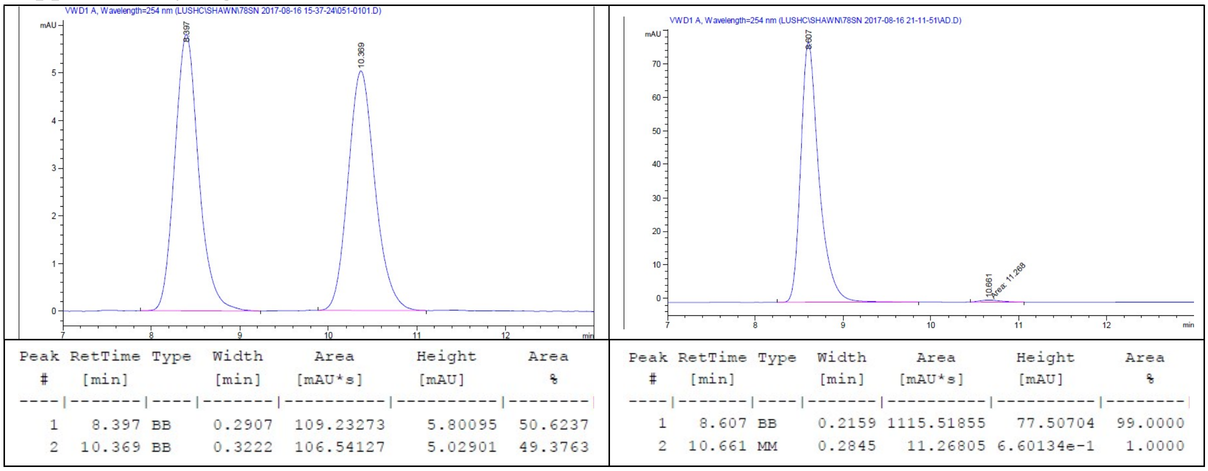

Peak #	RetTime [min]	Type	Width [min]	Area [mAU*s]	Height [mAU]	Area %
1	8.397	BB	0.2907	109.23273	5.80095	50.6237
2	10.369	BB	0.3222	106.54127	5.02901	49.3763

Peak #	RetTime [min]	Type	Width [min]	Area [mAU*s]	Height [mAU]	Area %
1	8.607	BB	0.2159	1115.51855	77.50704	99.0000
2	10.661	MM	0.2845	11.26805	6.60134e-1	1.0000

Product 3a

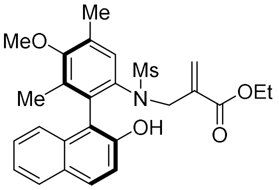

Syrup. ^{1}**H NMR** (500 MHz, DMSO-d_6, 80 °C) δ 9.08 (br, 1H), 7.87 – 7.81 (m, 2H), 7.32 – 7.23 (m, 3H), 7.14 (s, 1H), 7.10 – 7.04 (m, 1H), 6.02 (s, 1H), 5.56 (s, 1H), 4.14 (d, J = 16.6 Hz, 1H), 4.05 (q, J = 7.2 Hz, 2H), 3.93 (d, J = 16.2 Hz, 1H), 3.75 (s, 3H), 2.47 (s, 3H), 2.35 (s, 3H), 1.74 (s, 3H), 1.17 (t, J = 7.1 Hz, 3H). ^{13}**C NMR** (125 MHz, DMSO-d_6, 80 °C) δ 166.07 , 156.88 , 152.29 , 136.60 , 135.84 , 135.52 , 133.94 , 132.32 , 132.03 , 130.12 , 129.75 , 128.60 , 128.24 , 128.03 , 126.50 , 124.76 , 123.15 , 118.97 ,

117.97 , 60.72 , 60.03 , 50.37 , 40.18 , 16.22 , 14.26 , 13.35 . **HRMS (ESI)** m/z Calcd for [$C_{26}H_{29}NNaO_6S$, M + Na]$^+$: 506.1608; Found: 506.1604.

Optical Rotation: $[\alpha]^{25}_{D}$ - 28.0 (c = 1.0, CH_2Cl_2). 60% ee (HPLC condition: Chiralcel AD-H column, n-Hexane/i-PrOH = 90:10, flow rate = 1.0 mL/min, wavelength = 254 nm, t_R = 9.15 min for minor isomer, t_R = 10.11 min for major isomer).

Supplementary Figure 4. HPLC Trace of Product **3a**.

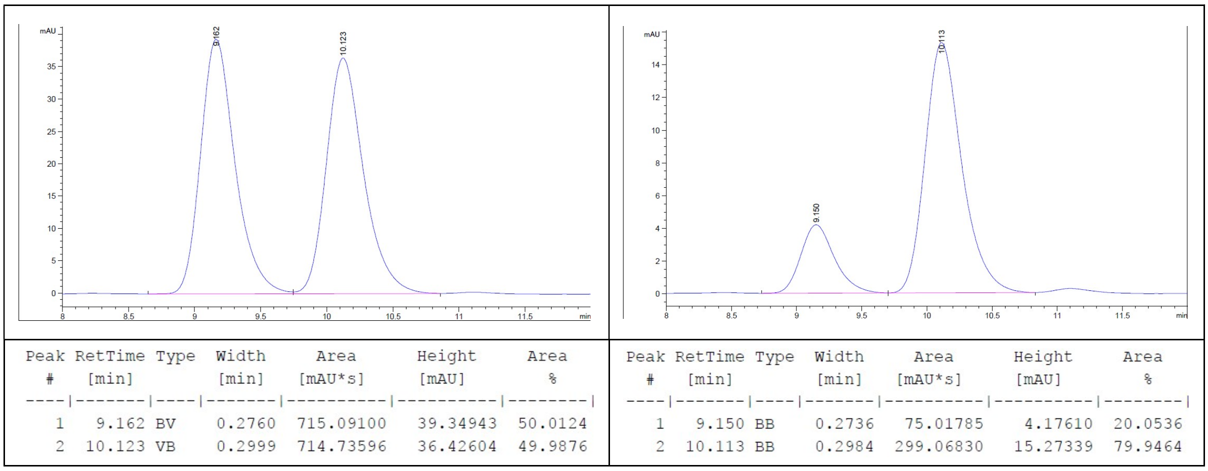

Peak #	RetTime [min]	Type	Width [min]	Area [mAU*s]	Height [mAU]	Area %
1	9.162	BV	0.2760	715.09100	39.34943	50.0124
2	10.123	VB	0.2999	714.73596	36.42604	49.9876

Peak #	RetTime [min]	Type	Width [min]	Area [mAU*s]	Height [mAU]	Area %
1	9.150	BB	0.2736	75.01785	4.17610	20.0536
2	10.113	BB	0.2984	299.06830	15.27339	79.9464

Product 4a

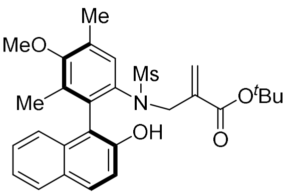

Syrup. **^{1}H NMR** (500 MHz, DMSO-d_6, 80 ºC) δ 9.05 (br, 1H), 7.84 – 7.80 (m, 2H), 7.33 – 7.22 (m, 3H), 7.13 (s, 1H), 7.08 – 7.03 (m, 1H), 5.94 (s, 1H), 5.50 (s, 1H), 4.09 (d, J = 16.5 Hz, 1H), 3.86 (d, J = 16.5 Hz, 1H), 3.73 (s, 3H), 2.45 (s, 3H), 2.34 (s, 3H), 1.72 (s, 3H), 1.38 (s, 9H). **^{13}C NMR** (125 MHz, DMSO-d_6, 80 ºC) δ 164.43 , 155.92 , 151.33 , 136.97 , 134.96 , 134.50 , 133.00 , 131.37 , 131.17 , 129.10 , 128.79 , 127.66 , 127.28 , 126.34 , 125.55 , 123.82 , 122.21 , 118.03 , 117.03 , 79.99 , 59.06 , 49.32 , 27.18 , 15.29 , 12.38 . **HRMS (ESI)** m/z Calcd for [$C_{28}H_{33}NNaO_6S$, M + Na]$^+$: 534.1921; Found: 534.1926.

Optical Rotation: $[\alpha]^{25}_{D}$ - 25.0 (c = 0.5, CH_2Cl_2). 65% ee (HPLC condition: Chiralcel ID column, n-Hexane/i-PrOH = 90:10, flow rate = 1.0 mL/min, wavelength = 254 nm, t_R = 11.26 min for major isomer, t_R = 12.61 min for minor isomer).

Supplementary Figure 5. HPLC Trace of Product **4a**.

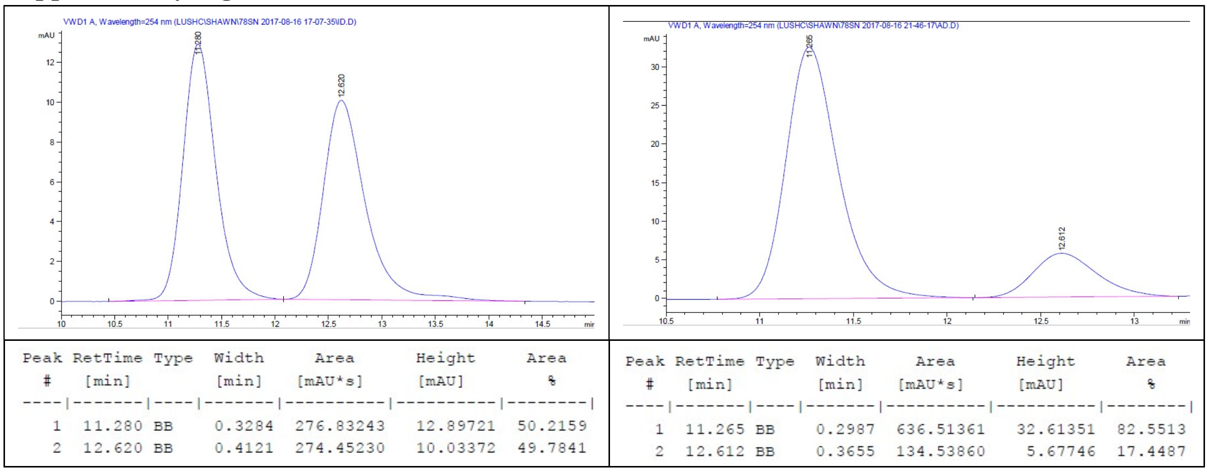

Peak #	RetTime [min]	Type	Width [min]	Area [mAU*s]	Height [mAU]	Area %
1	11.280	BB	0.3284	276.83243	12.89721	50.2159
2	12.620	BB	0.4121	274.45230	10.03372	49.7841

Peak #	RetTime [min]	Type	Width [min]	Area [mAU*s]	Height [mAU]	Area %
1	11.265	BB	0.2987	636.51361	32.61351	82.5513
2	12.612	BB	0.3655	134.53860	5.67746	17.4487

Recovered 1b

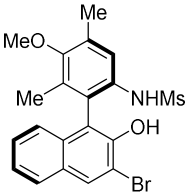

Syrup. ^{1}**H NMR** (500 MHz, $CDCl_3$): δ 8.21 (s, 1H), 7.83 – 7.79 (m, 1H), 7.53 (s, 1H), 7.42 (tt, *J* = 8.3, 3.4 Hz, 2H), 7.18 – 7.14 (m, 1H), 5.74 (s, 1H), 5.72 (s, 1H), 3.79 (s, 3H), 2.72 (s, 3H), 2.45 (s, 3H), 1.88 (s, 3H). ^{13}**C NMR** (125 MHz, $CDCl_3$): δ 154.95 , 147.02 , 132.82 , 132.72 , 132.19 , 132.12 , 131.21 , 129.84 , 128.10 , 127.78 , 125.25 , 124.94 , 123.66 , 121.76 , 116.29 , 112.43 , 60.12 , 39.52 , 16.55 , 13.40 . **HRMS (ESI)** m/z Calcd for $[C_{20}H_{19}NO_4SBr, M - H]^-$: 448.0224; Found: 448.0230.

Optical Rotation: $[\alpha]^{25}_{D}$ + 12.0 (*c* = 0.4, $CHCl_3$). The absolute configuration of **1b** was assigned by conversion to **6a** followed by single crystal X-ray analysis. 98% ee (HPLC condition: Chiralpak AD-H column, *n*-Hexane/*i*-PrOH = 90:10, flow rate = 1.0 mL/min, wavelength = 254 nm, t_R = 11.88 min for major isomer, t_R = 12.78 min for minor isomer).

Supplementary Figure 6. HPLC Trace of Recovered **1b**.

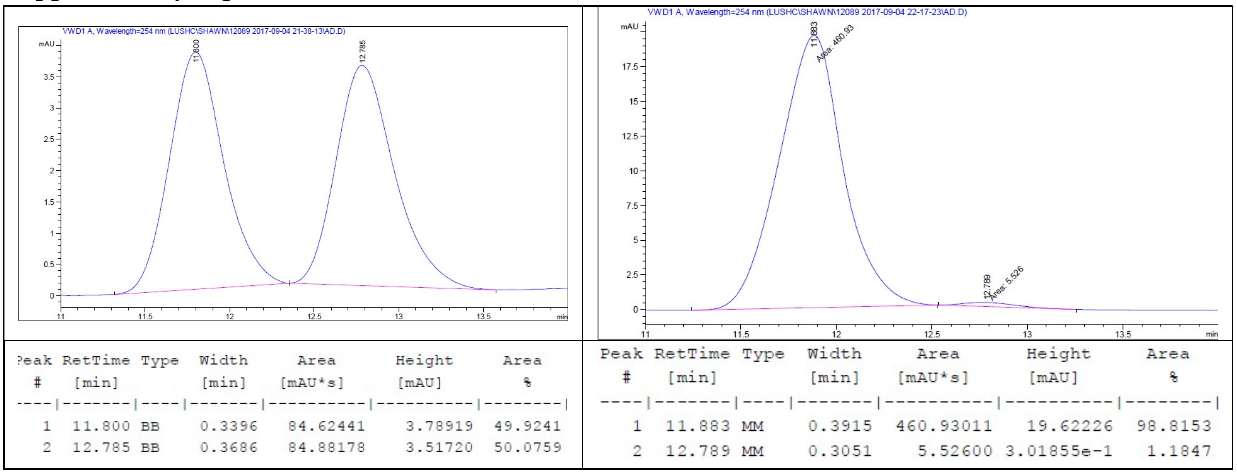

Peak #	RetTime [min]	Type	Width [min]	Area [mAU*s]	Height [mAU]	Area %
1	11.800	BB	0.3396	84.62441	3.78919	49.9241
2	12.785	BB	0.3686	84.88178	3.51720	50.0759

Peak #	RetTime [min]	Type	Width [min]	Area [mAU*s]	Height [mAU]	Area %
1	11.883	MM	0.3915	460.93011	19.62226	98.8153
2	12.789	MM	0.3051	5.52600	3.01855e-1	1.1847

Product 4b

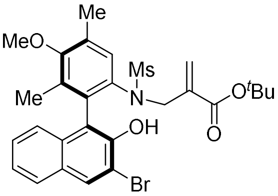

White solid. **MP**: 112 - 113 °C. **^{1}H NMR** (500 MHz, DMSO-d_6, 80 °C): δ 8.41 (br, 1H), 8.29 (s, 1H), 7.85 (dd, J = 7.8, 1.5 Hz, 1H), 7.35 (ddd, J = 8.1, 6.7, 1.4 Hz, 1H), 7.31 (ddd, J = 8.2, 6.8, 1.5 Hz, 1H), 7.19 (s, 1H), 7.02 (d, J = 8.2 Hz, 1H), 5.92 (s, 1H), 5.43 (s, 1H), 4.00 (d, J = 16.4 Hz, 1H), 3.87 (d, J = 16.4 Hz, 1H), 3.76 (s, 3H), 2.61 (s, 3H), 2.37 (s, 3H), 1.73 (s, 3H), 1.40 (s, 9H). **^{13}C NMR** (125MHz, DMSO-d_6, 80 °C): δ 164.40 , 156.25 , 147.42 , 136.41 , 135.10 , 133.37 , 131.93 , 131.73 , 131.34 , 130.91 , 130.27 , 128.59 , 126.80 , 126.54 , 126.08 , 124.09 , 123.65 , 119.96 , 113.18 , 80.16 , 59.09 , 49.71 , 39.23 , 27.18 , 15.34 , 12.54 . **HRMS (ESI)** m/z Calcd for [$C_{28}H_{32}BrNNaO_6S$, M + Na]$^+$: 612.1026; Found: 612.1022.

Optical Rotation: $[\alpha]^{25}_D$ – 35.0 (c = 1.0, CH_2Cl_2). 73% ee (HPLC condition: Chiralpak AD-H column, n-Hexane/i-PrOH = 90:10, flow rate = 1.0 mL/min, wavelength = 254 nm, t_R = 6.91 min for major isomer, t_R = 7.65 min minor for isomer).

Supplementary Figure 7. HPLC Trace of Product **4b**.

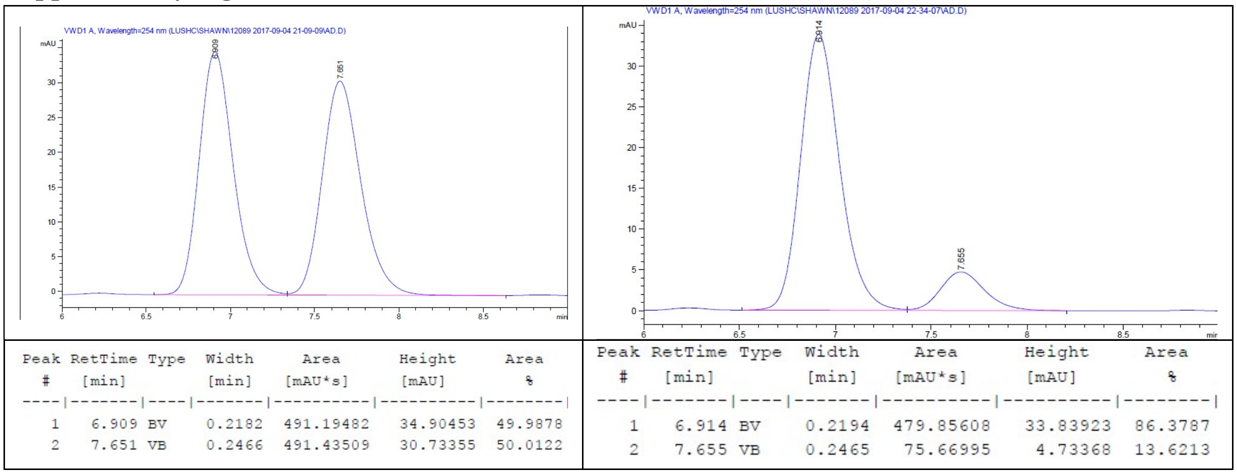

Peak #	RetTime [min]	Type	Width [min]	Area [mAU*s]	Height [mAU]	Area %
1	6.909	BV	0.2182	491.19482	34.90453	49.9878
2	7.651	VB	0.2466	491.43509	30.73355	50.0122

Peak #	RetTime [min]	Type	Width [min]	Area [mAU*s]	Height [mAU]	Area %
1	6.914	BV	0.2194	479.85608	33.83923	86.3787
2	7.655	VB	0.2465	75.66995	4.73368	13.6213

Recovered 1c

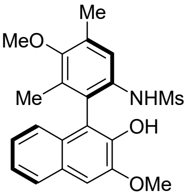

Syrup. **^{1}H NMR** (400 MHz, $CDCl_3$): δ 7.69 – 7.67 (m, 1H), 7.42 (s, 1H), 7.28 (ddd, J = 8.1, 6.9, 1.3 Hz, 1H), 7.21 – 7.14 (m, 2H), 7.07 – 7.00 (m, 1H), 6.05 (s, 1H), 5.78 (s, 1H), 4.01 (s, 3H), 3.69 (s, 3H), 2.54 (s, 3H), 2.33 (s, 3H), 1.79 (s, 3H). **^{13}C NMR** (100 MHz, $CDCl_3$): δ 154.86 , 146.87 , 143.00 , 132.04 , 132.02 , 131.02 , 129.27 , 127.91 , 127.37 , 125.86 , 125.39 , 124.75 , 123.45 , 121.75 , 114.93 , 106.69 ,

60.05 , 56.05 , 39.15 , 16.49 , 13.47 . **HRMS (ESI)** m/z Calcd for [$C_{21}H_{22}NO_5S$, M - H]$^-$: 400.1224; Found: 400.1231.

Optical Rotation: $[\alpha]^{25}_{D}$ - 13.0 (*c* = 0.2, $CHCl_3$). The absolute configuration of **1c** was assigned by analogy to **1b**. 97% ee (HPLC condition: Chiralpak AD-H column, *n*-Hexane/*i*-PrOH = 90:10, flow rate = 1.0 mL/min, wavelength = 254 nm, t_R = 17.36 min for major isomer, t_R = 23.88 min for minor isomer).

Supplementary Figure 8. HPLC Trace of Recovered **1c**.

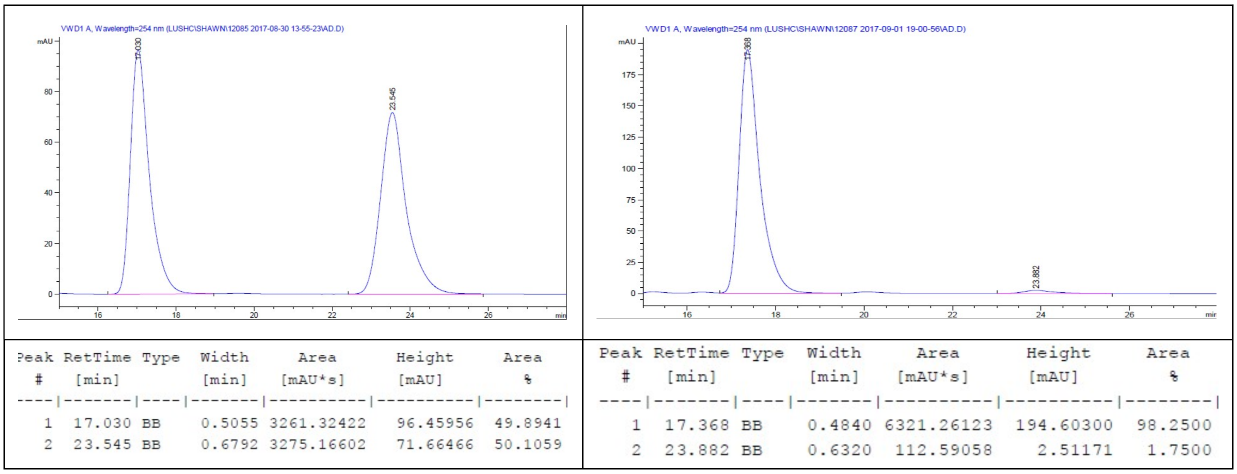

Peak #	RetTime [min]	Type	Width [min]	Area [mAU*s]	Height [mAU]	Area %
1	17.030	BB	0.5055	3261.32422	96.45956	49.8941
2	23.545	BB	0.6792	3275.16602	71.66466	50.1059

Peak #	RetTime [min]	Type	Width [min]	Area [mAU*s]	Height [mAU]	Area %
1	17.368	BB	0.4840	6321.26123	194.60300	98.2500
2	23.882	BB	0.6320	112.59058	2.51171	1.7500

Product 4c

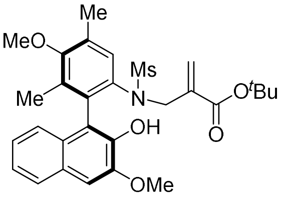

Syrup: **^{1}H NMR** (500 MHz, DMSO-d_6): δ 8.44 (br, 1H), 7.77 (d, *J* = 8.1 Hz, 1H), 7.40 (s, 1H), 7.27 (ddd, *J* = 8.1, 6.8, 1.2 Hz, 1H), 7.17 – 7.10 (m, 2H), 7.00 (d, *J* = 8.4 Hz, 1H), 5.95 (s, 1H), 5.50 (s, 1H), 4.07 (d, *J* = 16.7 Hz, 1H), 4.02 (s, 3H), 3.88 (d, *J* = 16.4 Hz, 1H), 3.74 (s, 3H), 2.46 (s, 3H), 2.35 (s, 3H), 1.73 (s, 3H), 1.40 (s, 9H). **^{13}C NMR** (125 MHz, DMSO-d_6): δ 165.36 , 156.84 , 149.05 , 144.21 , 137.92 , 135.82 , 135.16 , 132.14 , 130.10 , 129.08 , 128.90 , 127.31 , 127.07 , 124.62 , 124.21 , 123.83 , 118.85 , 107.25 , 104.99 , 80.93 , 60.03 , 56.38 , 50.34 , 40.23 , 28.12 , 16.25 , 13.33 . **HRMS (ESI)** m/z Calcd for [$C_{29}H_{35}NNaO_7S$, M + Na]$^+$: 564.2026; Found: 564.2021.

Optical Rotation: $[\alpha]^{25}_{D}$ + 8.0 (*c* = 1.0, CH_2Cl_2). 51% ee (HPLC condition: Chiralpak AD-H column, *n*-Hexane/*i*-PrOH = 90:10, flow rate = 1.0 mL/min, wavelength = 254 nm, t_R = 13.47 min for minor isomer, t_R = 16.03 min for major isomer).

Supplementary Figure 9. HPLC Trace of Product **4c**.

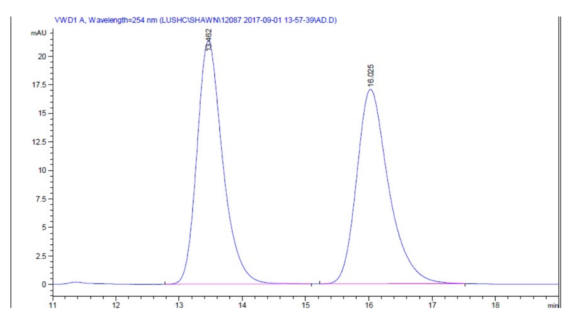

Peak #	RetTime [min]	Type	Width [min]	Area [mAU*s]	Height [mAU]	Area %
1	13.462	BB	0.4297	602.08337	21.30266	50.1175
2	16.025	BB	0.5251	599.25977	17.04644	49.8825

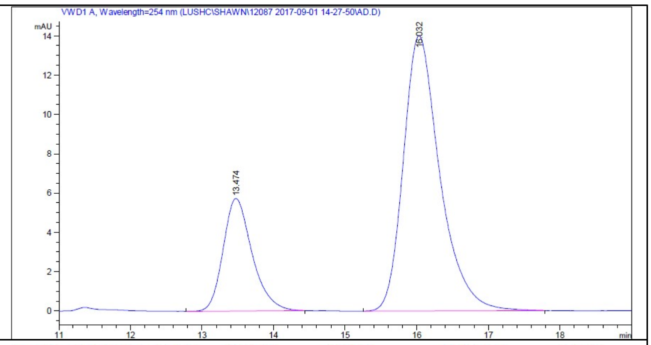

Peak #	RetTime [min]	Type	Width [min]	Area [mAU*s]	Height [mAU]	Area %
1	13.474	BB	0.4206	160.73965	5.72367	24.4490
2	16.032	BB	0.5299	496.70993	14.00249	75.5510

Recovered 1d

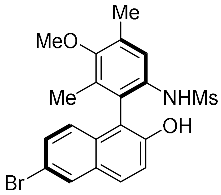

Syrup. **^{1}H NMR** (500 MHz, $CDCl_3$): δ 8.04 (d, J = 1.9 Hz, 1H), 7.81 (d, J = 9.0 Hz, 1H), 7.50 (s, 1H), 7.45 (dd, J = 9.0, 1.9 Hz, 1H), 7.31 (d, J = 9.0 Hz, 1H), 7.01 (d, J = 8.9 Hz, 1H), 5.79 (s, 1H), 5.44 (s, 1H), 3.79 (s, 3H), 2.75 (s, 3H), 2.43 (s, 3H), 1.87 (s, 3H). **^{13}C NMR** (125 MHz, $CDCl_3$): δ 155.05 , 151.37 , 133.51 , 132.94 , 131.91 , 131.08 , 130.84 , 130.70 , 130.47 , 130.18 , 125.04 , 123.15 , 121.55 , 119.07 , 117.94 , 114.52 , 60.16 , 39.80 , 16.59 , 13.33 . **HRMS (ESI)** m/z Calcd for $[C_{20}H_{19}NO_4SBr, M - H]^-$: 448.0224; Found: 448.0222.

Optical Rotation: $[\alpha]^{25}_D$ + 18.0 (c = 1.0, $CHCl_3$). The absolute configuration of **1d** was assigned by analogy to **1b**. 98% ee (HPLC condition: Chiralpak ID column, n-Hexane/i-PrOH = 90:10, flow rate = 1.0 mL/min, wavelength = 254 nm, t_R = 10.07 min for minor isomer, t_R = 11.51 min for major isomer).

Supplementary Figure 10. HPLC Trace of Recovered **1d**.

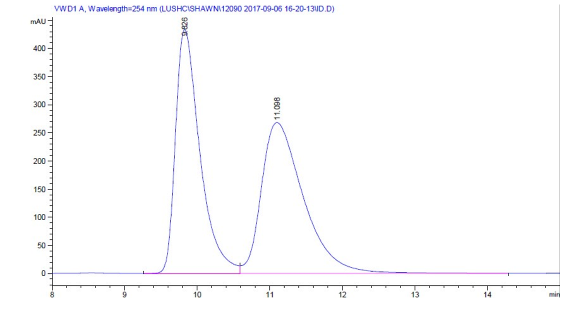

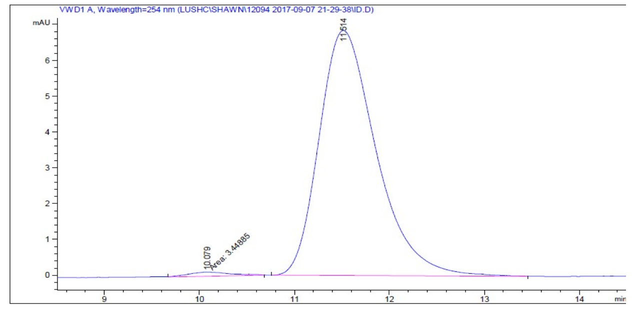

Peak #	RetTime [min]	Type	Width [min]	Area [mAU*s]	Height [mAU]	Area %
1	9.826	BV	0.3536	1.01352e4	433.75708	48.4516
2	11.098	VB	0.6121	1.07830e4	268.20438	51.5484

Peak #	RetTime [min]	Type	Width [min]	Area [mAU*s]	Height [mAU]	Area %
1	10.079	MM	0.4976	3.44885	1.15515e-1	1.1918
2	11.514	BB	0.6177	285.92834	6.84037	98.8082

Product 4d

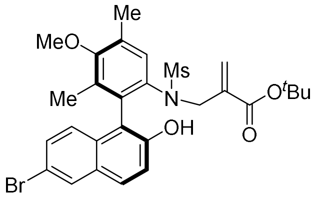

Syrup: ^{1}**H NMR** (500 MHz, DMSO-d_6, 80 °C): δ 9.31 (br, 1H), 8.07 (d, J = 2.0 Hz, 1H), 7.85 (d, J = 8.9 Hz, 1H), 7.37 – 7.32 (m, 2H), 7.15 (s, 1H), 7.00 (d, J = 9.0 Hz, 1H), 5.95 (s, 1H), 5.50 (s, 1H), 4.10 (d, J = 16.5 Hz, 1H), 3.90 (d, J = 16.5 Hz, 1H), 3.74 (s, 3H), 2.59 (s, 3H), 2.35 (s, 3H), 1.72 (s, 3H), 1.40 (s, 9H). ^{13}**C NMR** (125 MHz, DMSO-d_6, 80 °C): δ 165.35 , 156.90 , 152.87 , 137.74 , 135.98 , 134.96 , 132.62 , 132.31 , 131.86 , 130.41 , 129.96 , 129.81 , 129.27 , 129.06 , 127.44 , 127.29 , 120.18 , 118.30 , 116.19 , 81.01 , 60.04 , 50.42 , 40.49 , 28.13 , 16.24 , 13.35 . **HRMS (ESI)** m/z Calcd for [$C_{28}H_{32}BrNNaO_6S$, M + Na]$^+$: 612.1026; Found: 612.1029.

Optical Rotation: $[\alpha]^{25}_{D}$ – 20.0 (c = 1.0, CH_2Cl_2). 66% ee (HPLC condition: Chiralpak ID column, n-Hexane/i-PrOH = 90:10, flow rate = 1.0 mL/min, wavelength = 254 nm, t_R = 10.02 min for major isomer, t_R = 11.57 min for minor isomer).

Supplementary Figure 11. HPLC Trace of Product **4d**.

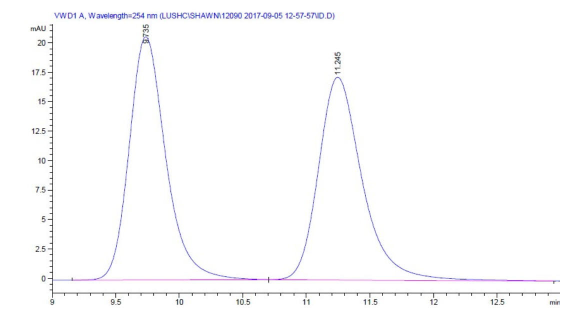

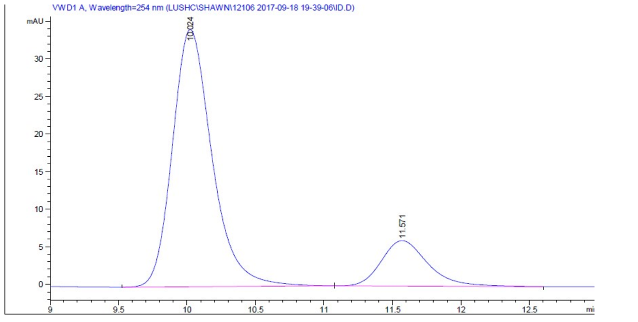

Peak #	RetTime [min]	Type	Width [min]	Area [mAU*s]	Height [mAU]	Area %
1	9.735	BB	0.3058	413.77029	20.64384	50.1041
2	11.245	BB	0.3624	412.05130	17.20715	49.8959

Peak #	RetTime [min]	Type	Width [min]	Area [mAU*s]	Height [mAU]	Area %
1	10.024	BB	0.3069	689.10620	34.22895	82.8203
2	11.571	BB	0.3584	142.94388	6.05619	17.1797

Recovered 1e

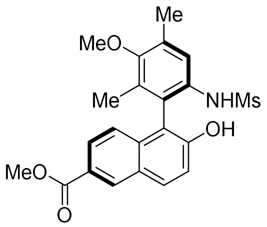

Syrup. **^{1}H NMR** (500 MHz, $CDCl_3$): δ 8.62 (d, J = 1.6 Hz, 1H), 8.00 (d, J = 9.0 Hz, 1H), 7.95 (dd, J = 8.7, 1.8 Hz, 1H), 7.49 (s, 1H), 7.36 (d, J = 8.9 Hz, 1H), 7.17 (d, J = 8.8 Hz, 1H), 5.92 (br, 1H), 5.83 (s, 1H), 3.98 (s, 3H), 3.78 (s, 3H), 2.73 (s, 3H), 2.42 (s, 3H), 1.87 (s, 3H). **^{13}C NMR** (125MHz, $CDCl_3$): δ 167.14 , 155.06 , 153.31 , 135.09 , 133.44 , 132.92 , 132.57 , 131.89 , 131.80 , 128.35 , 127.04 , 125.66 , 123.45 , 123.37 , 121.64 , 118.88 , 114.58 , 60.15 , 52.31 , 39.76 , 16.58 , 13.35 . **HRMS (ESI)** m/z Calcd for $[C_{22}H_{22}NO_6S, M - H]^-$: 428.1173; Found: 428.1174.

Optical Rotation: $[\alpha]^{25}_{D}$ + 23.0 (c = 1.0, $CHCl_3$). The absolute configuration of **1e** was assigned by analogy to **1b**. 95% ee (HPLC condition: Chiralpak AD-H column, n-Hexane/i-PrOH = 90:10, flow rate = 1.0 mL/min, wavelength = 254 nm, t_R = 15.45 min for minor isomer, t_R = 19.40 min for major isomer).

Supplementary Figure 12. HPLC Trace of Recovered **1e**.

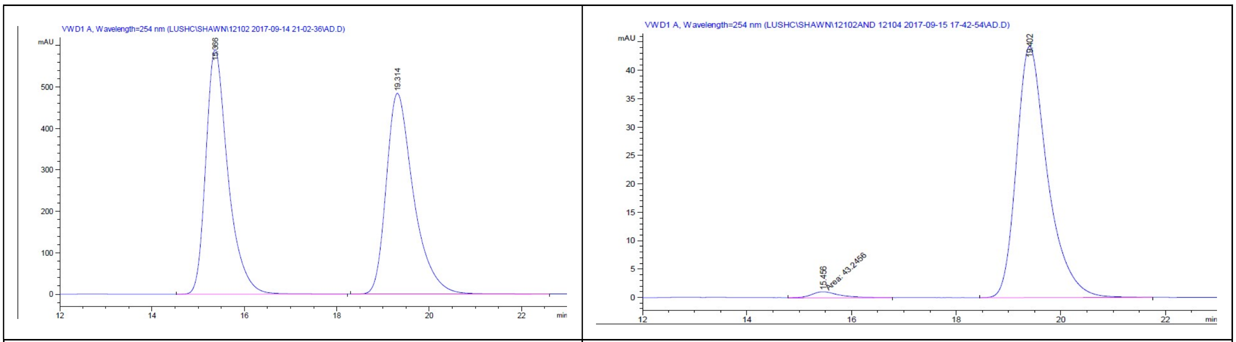

Peak #	RetTime [min]	Type	Width [min]	Area [mAU*s]	Height [mAU]	Area %
1	15.366	BB	0.5009	1.96546e4	589.57190	49.7112
2	19.314	BB	0.6170	1.98830e4	484.24576	50.2888

Peak #	RetTime [min]	Type	Width [min]	Area [mAU*s]	Height [mAU]	Area %
1	15.456	MM	0.6545	43.24559	1.10122	2.3192
2	19.402	BB	0.6140	1821.47021	44.27100	97.6808

Product 4e

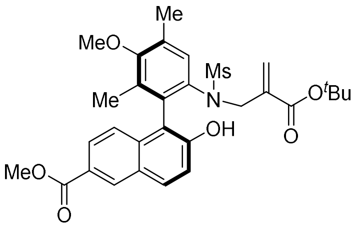

Syrup. **^{1}H NMR** (500 MHz, DMSO-d_6, 80 °C): δ 9.60 (br, 1H), 8.54 (d, J = 1.7 Hz, 1H), 8.05 (d, J = 8.9 Hz, 1H), 7.73 (dt, J = 8.9, 1.3 Hz, 1H), 7.44 – 7.37 (m, 1H), 7.16 (s, 1H), 7.13 (d, J = 8.9 Hz, 1H), 5.93 (s, 1H), 5.48 (s, 1H), 4.08 (d, J = 16.6 Hz, 1H), 3.91 – 3.88 (m, 4H), 3.75 (s, 3H), 2.56 (s, 3H), 2.36 (s, 3H), 1.73 (s, 3H), 1.38 (s, 9H). **^{13}C NMR** (125 MHz, DMSO-d_6, 80 °C): δ 165.97 , 164.37 , 155.96 , 153.83 , 136.75 , 135.43 , 135.06 , 134.01 , 131.34 , 130.93 , 130.51 , 130.13 , 129.48 , 126.56 , 126.40 , 124.50 , 124.33 , 123.61 , 118.97 , 117.30 , 80.02 , 59.08 , 51.21 , 49.49 , 27.13 , 15.30 , 12.39 . **HRMS (ESI)** m/z Calcd for $[C_{30}H_{35}NNaO_8S, M + Na]^+$: 592.2083; Found: 592.2087.

Optical Rotation: $[\alpha]^{25}_{D}$ - 20.0 (c = 1.0, CH_2Cl_2). 66% ee (HPLC condition: Chiralpak AD-H column, n-Hexane/i-PrOH = 90:10, flow rate = 1.0 mL/min, wavelength = 254 nm, t_R = 13.49 min for major isomer, t_R = 15.85 min for minor isomer).

Supplementary Figure 13. HPLC Trace of Product **4e**.

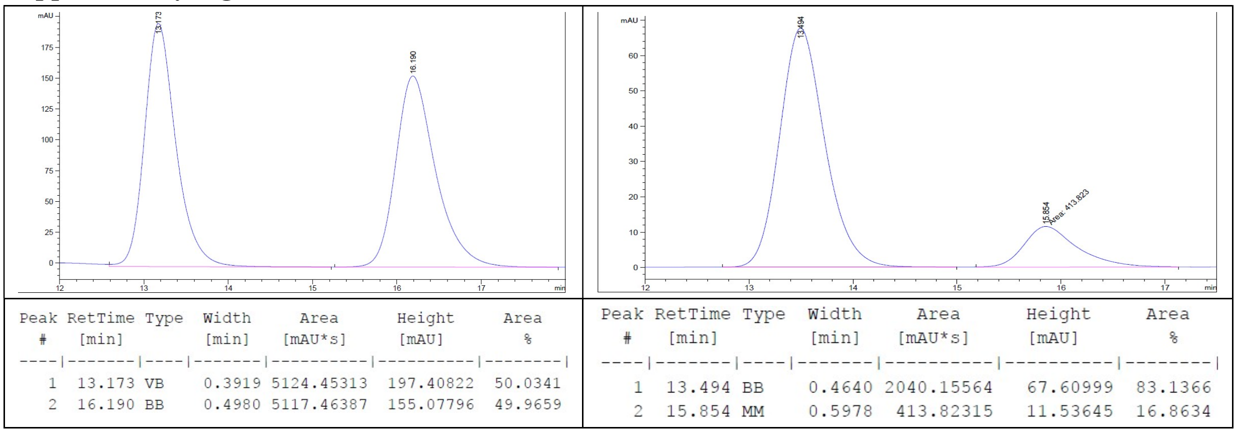

Peak #	RetTime [min]	Type	Width [min]	Area [mAU*s]	Height [mAU]	Area %
1	13.173	VB	0.3919	5124.45313	197.40822	50.0341
2	16.190	BB	0.4980	5117.46387	155.07796	49.9659

Peak #	RetTime [min]	Type	Width [min]	Area [mAU*s]	Height [mAU]	Area %
1	13.494	BB	0.4640	2040.15564	67.60999	83.1366
2	15.854	MM	0.5978	413.82315	11.53645	16.8634

Recovered 1f

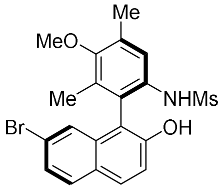

Syrup. **^{1}H NMR** (500 MHz, $CDCl_3$): δ 7.87 (d, J = 8.9 Hz, 1H), 7.75 (d, J = 8.7 Hz, 1H), 7.53 (s, 1H), 7.48 (dd, J = 8.7, 1.9 Hz, 1H), 7.30 (d, J = 9.0 Hz, 1H), 7.25 (d, J = 1.9 Hz, 1H), 5.76 (s, 1H), 5.41 (s, 1H), 3.81 (s, 3H), 2.80 (s, 3H), 2.45 (s, 3H), 1.90 (s, 3H). **^{13}C NMR** (125 MHz, $CDCl_3$): δ 155.01 , 152.05 , 133.78 , 133.73 , 132.99 , 132.02 , 131.12 , 130.43 , 127.79 , 127.59 , 125.24 , 122.54 , 122.27 , 121.21 , 118.36 , 113.44 , 60.20 , 39.70 , 16.64 , 13.34 . **HRMS (ESI)** m/z Calcd for $[C_{20}H_{19}NO_4SBr, M - H]^-$: 448.0224; Found: 448.0232.

Optical Rotation: $[\alpha]^{25}_D$ + 68.0 (c = 0.5, $CHCl_3$). The absolute configuration of **1f** was assigned by analogy to **1b**. 99% ee (HPLC condition: Chiralpak ID column, n-Hexane/i-PrOH = 90:10, flow rate = 1.0 mL/min, wavelength = 254 nm, t_R = 8.61 min for major isomer, t_R = 9.88 min for minor isomer).

Supplementary Figure 14. HPLC Trace of Recovered **1f**.

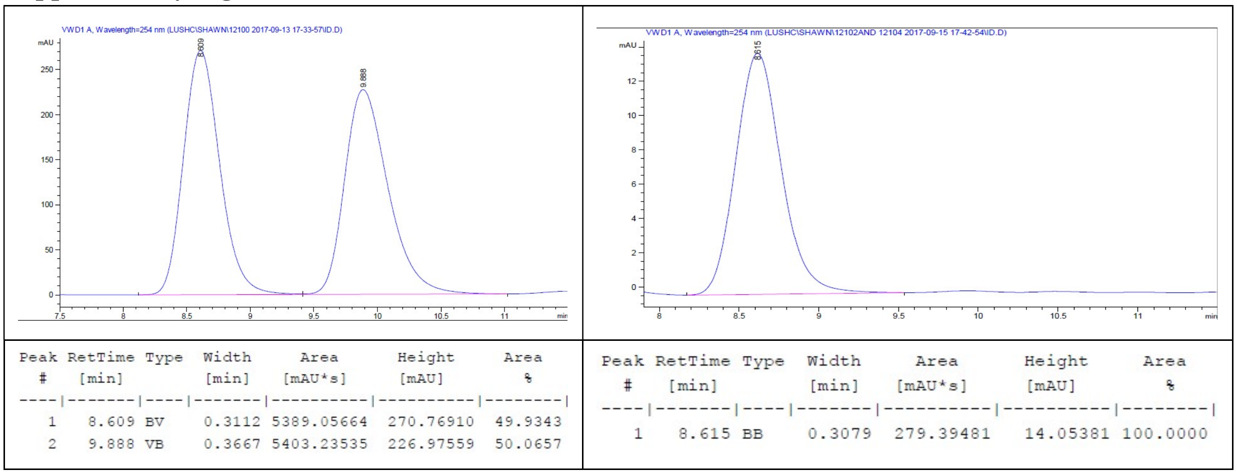

Peak #	RetTime [min]	Type	Width [min]	Area [mAU*s]	Height [mAU]	Area %
1	8.609	BV	0.3112	5389.05664	270.76910	49.9343
2	9.888	VB	0.3667	5403.23535	226.97559	50.0657

Peak #	RetTime [min]	Type	Width [min]	Area [mAU*s]	Height [mAU]	Area %
1	8.615	BB	0.3079	279.39481	14.05381	100.0000

Product 4f

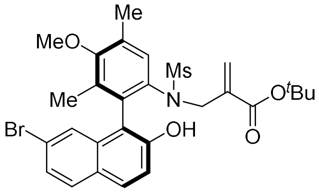

Syrup. **^{1}H NMR** (500 MHz, DMSO-d_6, 80 °C): δ 9.40 (br, 1H), 7.87 (d, J = 8.9 Hz, 1H), 7.79 (d, J = 8.7 Hz, 1H), 7.37 (dd, J = 8.7, 2.0 Hz, 1H), 7.33 (d, J = 8.9 Hz, 1H), 7.23 (s, 1H), 7.15 (s, 1H), 5.96 (s, 1H), 5.49 (s, 1H), 4.08 (d, J = 16.4 Hz, 1H), 3.91 (d, J = 16.4 Hz, 1H), 3.75 (s, 3H), 2.60 (s, 3H), 2.36 (s, 3H), 1.74 (s, 3H), 1.40 (s, 9H). **^{13}C NMR** (125 MHz, DMSO-d_6): δ 165.33 , 156.90 , 153.41 , 137.70 , 136.07 , 135.27 , 134.83 , 132.31 , 131.82 , 130.51 , 130.34 , 129.92 , 127.60 , 127.05 , 126.94 , 126.15 , 120.48 , 119.49 , 117.35 , 81.03 , 60.05 , 50.49 , 40.54 , 28.14 , 16.27 , 13.37 . **HRMS (ESI)** m/z Calcd for $[C_{28}H_{32}BrNNaO_6S, M + Na]^+$: 612.1026; Found: 612.1024.

Optical Rotation: $[\alpha]^{25}_D$ - 22.0 (c = 1.0, CH_2Cl_2). 53% ee (HPLC condition: Chiralpak AD-H column, n-Hexane/i-PrOH = 90:10, flow rate = 1.0 mL/min, wavelength = 254 nm, t_R = 7.36 min for major isomer, t_R = 8.74 min for minor isomer).

Supplementary Figure 15. HPLC Trace of Product **4f**.

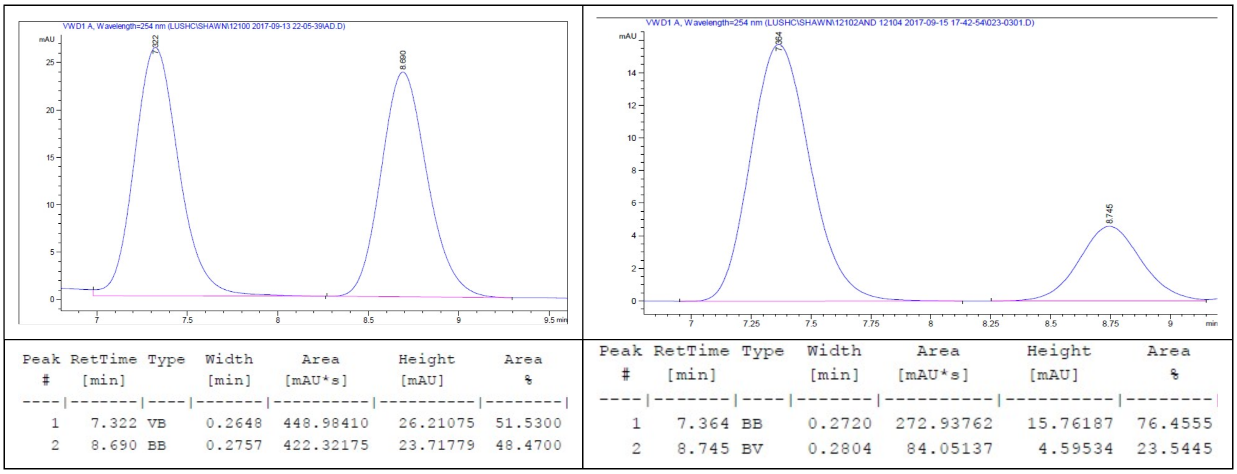

Peak #	RetTime [min]	Type	Width [min]	Area [mAU*s]	Height [mAU]	Area %
1	7.322	VB	0.2648	448.98410	26.21075	51.5300
2	8.690	BB	0.2757	422.32175	23.71779	48.4700

Peak #	RetTime [min]	Type	Width [min]	Area [mAU*s]	Height [mAU]	Area %
1	7.364	BB	0.2720	272.93762	15.76187	76.4555
2	8.745	BV	0.2804	84.05137	4.59534	23.5445

Recovered 1g

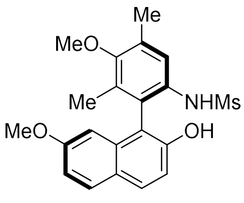

Syrup. **^{1}H NMR** (400 MHz, $CDCl_3$): δ 7.82 (d, J = 8.8 Hz, 1H), 7.78 (d, J = 8.9 Hz, 1H), 7.53 (s, 1H), 7.13 (d, J = 8.8 Hz, 1H), 7.07 (dd, J = 8.9, 2.5 Hz, 1H), 6.41 (d, J = 2.5 Hz, 1H), 5.84 (s, 1H), 5.09 (br, 1H), 3.80 (s, 3H), 3.71 (s, 3H), 2.76 (s, 3H), 2.44 (s, 3H), 1.94 (s, 3H). **^{13}C NMR** (100 MHz, $CDCl_3$): δ 159.29 , 155.09 , 151.53 , 133.87 , 133.25 , 132.88 , 132.01 , 130.89 , 130.39 , 124.75 , 123.67 , 121.67 , 115.79 , 115.07 , 113.22 , 102.55 , 60.13 , 55.28 , 39.62 , 16.59 , 13.25 . **HRMS (ESI)** m/z Calcd for $[C_{21}H_{22}NO_5S, M - H]^-$: 400.1224; Found: 400.1231.

Optical Rotation: $[\alpha]^{25}_{D}$ + 28.0 (c = 0.2, $CHCl_3$). The absolute configuration of **1g** was assigned by analogy to **1b**. 98% ee (HPLC condition: Chiralpak AD-H column, n-Hexane/i-PrOH = 90:10, flow rate = 1.0 mL/min, wavelength = 254 nm, t_R = 9.44 min for major isomer, t_R = 14.68 min for minor isomer).

Supplementary Figure 16. HPLC Trace of Recovered **1g**.

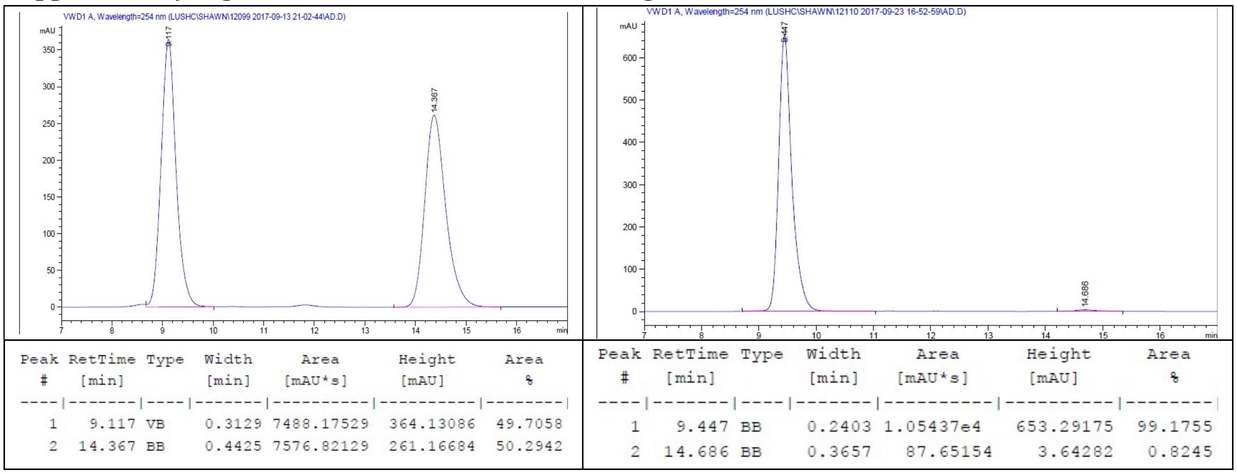

Peak #	RetTime [min]	Type	Width [min]	Area [mAU*s]	Height [mAU]	Area %
1	9.117	VB	0.3129	7488.17529	364.13086	49.7058
2	14.367	BB	0.4425	7576.82129	261.16684	50.2942

Peak #	RetTime [min]	Type	Width [min]	Area [mAU*s]	Height [mAU]	Area %
1	9.447	BB	0.2403	1.05437e4	653.29175	99.1755
2	14.686	BB	0.3657	87.65154	3.64282	0.8245

Product 4g

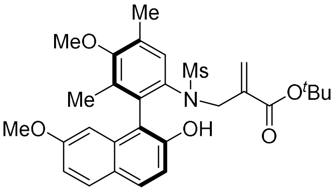

Syrup. **^{1}H NMR** (500 MHz, DMSO-d_6, 80 °C): δ 9.08 (br, 1H), 7.75 (d, J = 8.8 Hz, 1H), 7.73 (d, J = 8.9 Hz, 1H), 7.16 – 7.11 (m, 2H), 6.93 (dd, J = 8.9, 2.5 Hz, 1H), 6.43 (d, J = 2.5 Hz, 1H), 5.97 (s, 1H), 5.55 (s, 1H), 4.11 (d, J = 16.7 Hz, 1H), 3.90 (d, J = 16.6 Hz, 1H), 3.73 (s, 3H), 3.60 (s, 3H), 2.51 (s, 3H), 2.35 (s, 3H), 1.75 (s, 3H), 1.39 (s, 9H). **^{13}C NMR** (125 MHz, DMSO-d_6, 80 °C): δ 165.37 , 158.25 , 156.92 , 152.87 , 137.88 , 135.89 , 135.57 , 135.40 , 132.24 , 132.17 , 129.91 , 129.79 , 129.48 , 127.20 , 124.02 , 117.25 , 116.33 , 115.31 , 104.36 , 80.95 , 60.01 , 55.12 , 50.22 , 40.30 , 28.10 , 16.27 , 13.25 . **HRMS (ESI)** m/z Calcd for $[C_{29}H_{35}NNaO_7S, M + Na]^+$: 564.2026; Found: 564.2029.

Optical Rotation: $[\alpha]^{25}_{D}$ – 25.0 (c = 1.0, CH_2Cl_2). 68% ee (HPLC condition: Chiralpak OD-H column, n-Hexane/i-PrOH = 98:2, flow rate = 1.0 mL/min, wavelength = 254 nm, t_R = 17.62 min for minor isomer, t_R = 21.06 min for major isomer).

Supplementary Figure 17. HPLC Trace of Product **4g**.

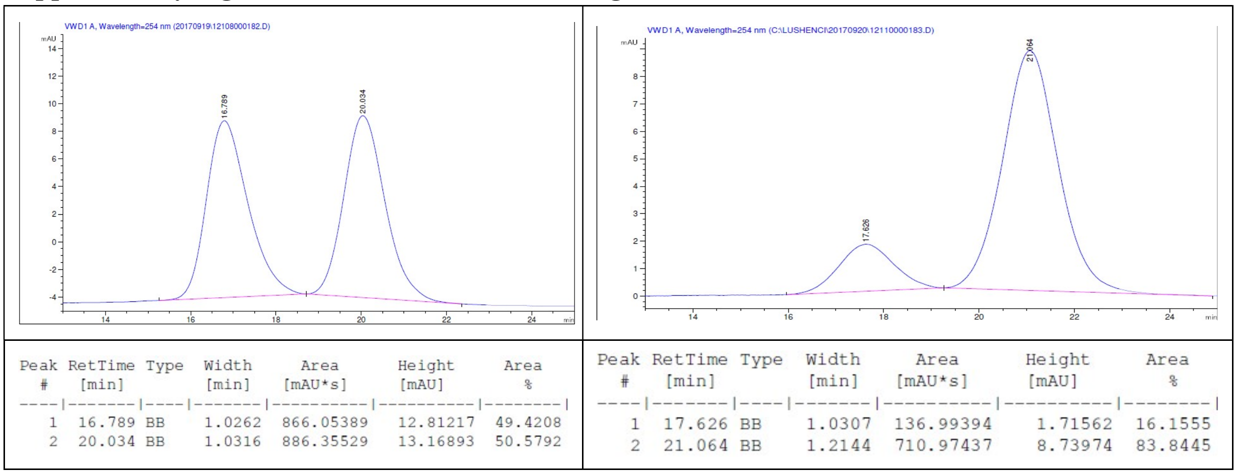

Peak #	RetTime [min]	Type	Width [min]	Area [mAU*s]	Height [mAU]	Area %
1	16.789	BB	1.0262	866.05389	12.81217	49.4208
2	20.034	BB	1.0316	886.35529	13.16893	50.5792

Peak #	RetTime [min]	Type	Width [min]	Area [mAU*s]	Height [mAU]	Area %
1	17.626	BB	1.0307	136.99394	1.71562	16.1555
2	21.064	BB	1.2144	710.97437	8.73974	83.8445

Recovered 1h

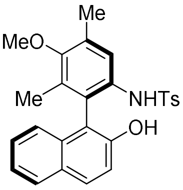

Syrup. **^{1}H NMR** (500 MHz, $CDCl_3$): δ 7.87 (d, *J* = 8.9 Hz, 1H), 7.84 (d, *J* = 8.0 Hz, 1H), 7.56 (s, 1H), 7.40 (dd, *J* = 8.6, 2.2 Hz, 2H), 7.36 (ddd, *J* = 8.0, 6.8, 1.2 Hz, 1H), 7.23 (d, *J* = 8.8 Hz, 1H), 7.21 – 7.17 (m, 1H), 7.11 (d, *J* = 8.1 Hz, 2H), 6.81 – 6.75 (m, 1H), 6.11 (s, 1H), 4.86 (br, 1H), 3.75 (s, 3H), 2.40 (s, 6H), 1.78 (s, 3H). **^{13}C NMR** (125 MHz, $CDCl_3$): δ 154.52 , 151.03 , 143.82 , 135.96 , 132.89 , 132.72 , 132.39 , 131.91 , 131.01 , 129.54 , 129.21 , 128.45 , 127.42 , 127.18 , 123.78 , 123.23 , 122.68 , 120.80 , 117.64 , 113.68 , 60.10 , 21.59 , 16.60 , 13.21 . **HRMS (ESI)** m/z Calcd for $[C_{26}H_{24}NO_4S, M - H]^-$: 446.1432; Found: 446.1430.

Optical Rotation: $[\alpha]^{25}_{D}$ + 50.0 (*c* = 0.2, $CHCl_3$). The absolute configuration of **1h** was assigned by analogy to **1b**. 95% ee (HPLC condition: Chiralpak ID column, *n*-Hexane/*i*-PrOH = 80:20, flow rate = 1.0 mL/min, wavelength = 254 nm, t_R = 9.36 min for major isomer, t_R = 11.38 min for minor isomer).

Supplementary Figure 18. HPLC Trace of Recovered **1h**.

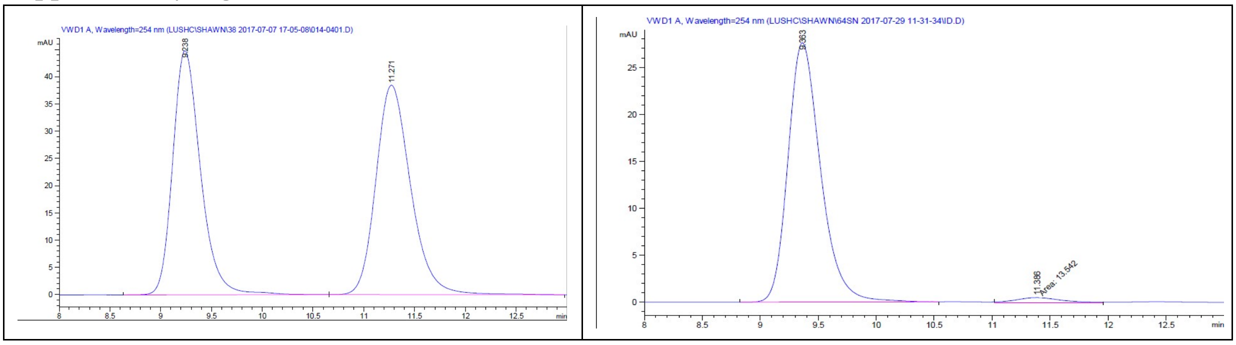

Peak #	RetTime [min]	Type	Width [min]	Area [mAU*s]	Height [mAU]	Area %
1	9.238	BB	0.2915	854.86621	44.81683	48.2714
2	11.271	BB	0.3659	916.09235	38.46063	51.7286

Peak #	RetTime [min]	Type	Width [min]	Area [mAU*s]	Height [mAU]	Area %
1	9.363	BB	0.2995	540.59430	27.60513	97.5562
2	11.386	MM	0.4307	13.54197	5.24025e-1	2.4438

Product 4h

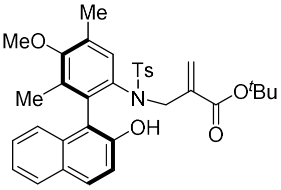

Syrup. **^{1}H NMR** (500 MHz DMSO-d_6, 80 ºC): δ 8.90 (br, 1H), 7.84 – 7.78 (m, 2H), 7.28 (ddd, J = 8.0, 6.7, 1.3 Hz, 1H), 7.26 – 7.21 (m, 2H), 7.17 – 7.06 (m, 5H), 6.95 (s, 1H), 5.78 (s, 1H), 5.16 (s, 1H), 4.09 (d, J = 16.6 Hz, 1H), 3.82 (d, J = 16.4 Hz, 1H), 3.75 (s, 3H), 2.33 (s, 3H), 2.31 (s, 3H), 1.74 (s, 3H), 1.38 (s, 9H). **^{13}C NMR** (125 MHz, DMSO-d_6, 80 ºC): δ 165.31 , 156.92 , 152.25 , 143.43 , 137.14 , 137.06 , 136.03 , 135.80 , 134.02 , 132.51 , 131.34 , 129.90 , 129.50 , 129.41 , 128.69 , 128.17 , 128.03 , 127.66 , 126.33 , 125.09 , 123.02 , 118.88 , 118.07 , 80.79 , 60.07 , 50.67 , 28.12 , 21.28 , 16.23 , 13.41 . **HRMS (ESI)** m/z Calcd for [$C_{34}H_{37}NNaO_6S$, M + Na]$^+$: 610.2234; Found: 610.2236.

Optical Rotation: $[\alpha]^{25}_D$ + 14.0 (c = 1.0, CH_2Cl_2). 72% ee (HPLC condition: Chiralpak AD-H column, n-Hexane/i-PrOH = 90:10, flow rate = 1.0 mL/min, wavelength = 254 nm, t_R = 11.97 min for minor isomer, t_R = 14.93 min for major isomer).

Supplementary Figure 19. HPLC Trace of Product **4h**.

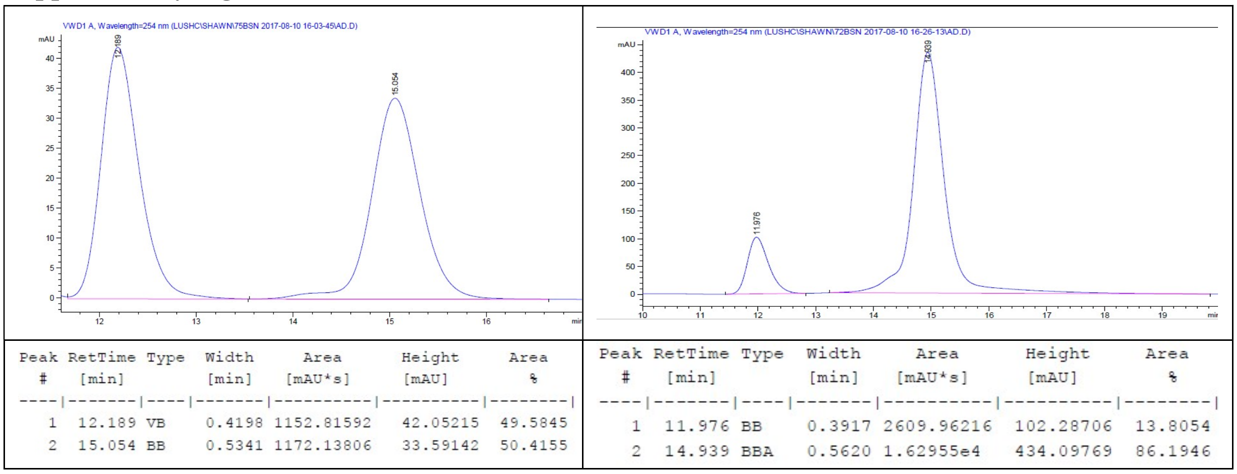

Peak #	RetTime [min]	Type	Width [min]	Area [mAU*s]	Height [mAU]	Area %
1	12.189	VB	0.4198	1152.81592	42.05215	49.5845
2	15.054	BB	0.5341	1172.13806	33.59142	50.4155

Peak #	RetTime [min]	Type	Width [min]	Area [mAU*s]	Height [mAU]	Area %
1	11.976	BB	0.3917	2609.96216	102.28706	13.8054
2	14.939	BBA	0.5620	1.62955e4	434.09769	86.1946

Recovered 1i

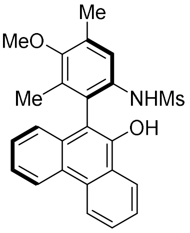

Syrup. **^{1}H NMR** (400 MHz, $CDCl_3$): δ 8.78 – 8.75 (m, 1H), 8.75 – 8.71 (m, 1H), 8.41 (ddd, J = 8.2, 1.5, 0.6 Hz, 1H), 7.81 (ddd, J = 8.4, 7.0, 1.5 Hz, 1H), 7.73 (ddd, J = 8.2, 7.0, 1.2 Hz, 1H), 7.61 – 7.55 (m, 2H), 7.47 (ddd, J = 8.2, 7.0, 1.2 Hz, 1H), 7.15 (ddd, J = 8.1, 1.4, 0.6 Hz, 1H), 5.89 (s, 1H), 5.38 (s, 1H), 3.82 (s, 3H), 2.76 (s, 3H), 2.48 (s, 3H), 1.97 (s, 3H). **^{13}C NMR** (100MHz, $CDCl_3$): δ 155.09 , 147.39 , 133.67 , 133.40 , 132.52 , 131.69 , 130.70 , 128.15 , 127.72 , 127.14 , 127.01 , 125.00 , 124.72 , 123.76 , 123.35 , 123.28 , 122.96 , 122.74 , 121.27 , 110.06 , 60.20 , 39.72 , 16.64 , 13.34 . **HRMS (ESI)** m/z Calcd for $[C_{24}H_{22}NO_4S, M - H]^-$: 420.1276; Found: 420.1274.

Optical Rotation: $[\alpha]^{25}_{D}$ + 16.0 (c = 0.4, $CHCl_3$). The absolute configuration of **1i** was assigned by analogy to **1b**. 99% ee (HPLC condition: Chiralpak AD-H column, n-Hexane/i-PrOH = 90:10, flow rate = 1.0 mL/min, wavelength = 254 nm, t_R = 10.45 min for major isomer, t_R = 21.85 min for minor isomer).

Supplementary Figure 20. HPLC Trace of Recovered **1i**.

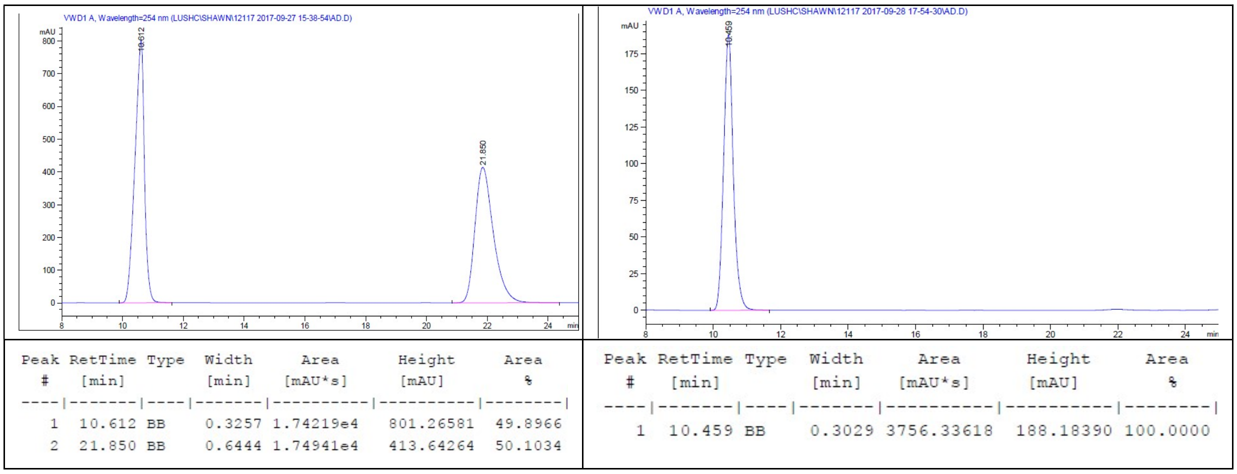

Peak #	RetTime [min]	Type	Width [min]	Area [mAU*s]	Height [mAU]	Area %
1	10.612	BB	0.3257	1.74219e4	801.26581	49.8966
2	21.850	BB	0.6444	1.74941e4	413.64264	50.1034

Peak #	RetTime [min]	Type	Width [min]	Area [mAU*s]	Height [mAU]	Area %
1	10.459	BB	0.3029	3756.33618	188.18390	100.0000

Product 4i

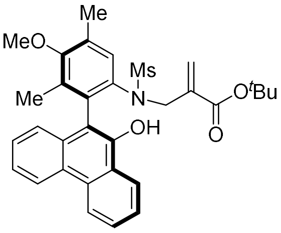

Syrup. **^{1}H NMR** (500 MHz, DMSO-d_6, 80 °C): δ 8.84 (d, J = 8.2 Hz, 1H), 8.76 (d, J = 8.2 Hz, 1H), 8.41 (dd, J = 8.2, 1.5 Hz, 1H), 7.77 (ddd, J = 8.4, 7.0, 1.5 Hz, 1H), 7.71 (ddd, J = 8.2, 7.0, 1.3 Hz, 1H), 7.50 (ddd, J = 8.3, 6.9, 1.4 Hz, 1H), 7.40 (ddd, J = 8.1, 6.9, 1.3 Hz, 1H), 7.21 (s, 1H), 7.08 (dd, J = 8.3, 1.3 Hz, 1H), 5.88 (s, 1H), 5.48 (s, 1H), 4.04 (d, J = 16.4 Hz, 1H), 3.87 (d, J = 16.4 Hz, 1H), 3.78 (d, J = 1.4 Hz, 3H), 2.59 (s, 3H), 2.39 (s, 3H), 1.80 (s, 3H), 1.37 (s, 9H). **^{13}C NMR** (125 MHz, DMSO-d_6, 80 °C): δ 165.42 , 157.32 , 147.55 , 137.43 , 136.22 , 134.85 , 133.13 , 132.67 , 132.07 , 131.42 , 131.02 , 127.89 , 127.70 , 127.28 , 126.95 , 126.71 , 126.48 , 125.33 , 124.54 , 123.47 , 123.24 , 123.23 , 115.26 , 81.12 , 60.04 , 50.46 , 28.10 , 16.33 , 13.50 . **HRMS (ESI)** m/z Calcd for $[C_{32}H_{35}NNaO_6S, M + Na]^+$: 584.2077; Found: 584.2074.

Optical Rotation: $[\alpha]^{25}_{D}$ + 16.0 (c = 1.0, CH_2Cl_2). 68% ee (HPLC condition: Chiralpak IE column, n-Hexane/i-PrOH = 95:5, flow rate = 1.0 mL/min, wavelength = 254 nm, t_R = 24.80 min for

minor isomer, t_R = 26.78 min for major isomer).

Supplementary Figure 21. HPLC Trace of Product **4i**.

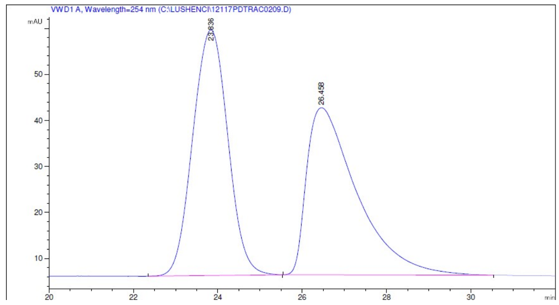

Peak #	RetTime [min]	Type	Width [min]	Area [mAU*s]	Height [mAU]	Area %
1	23.836	BB	0.9192	3078.33569	53.37529	50.5432
2	26.458	BB	1.2288	3012.16431	36.31038	49.4568

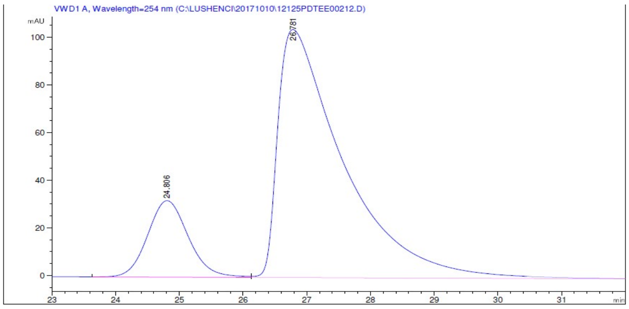

Peak #	RetTime [min]	Type	Width [min]	Area [mAU*s]	Height [mAU]	Area %
1	24.806	BV	0.6806	1426.99304	32.09324	16.0772
2	26.781	VBA	1.0330	7448.90039	104.11079	83.9228

Recovered 1j

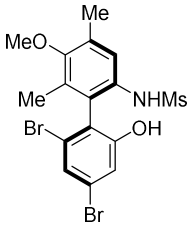

Syrup. **^{1}H NMR** (500 MHz, Acetone-d_6): δ 9.07 (br, 1H), 7.45 (d, J = 1.9 Hz, 1H), 7.41 (s, 1H), 7.24 (d, J = 1.9 Hz, 1H), 7.05 (br, 1H), 3.73 (s, 3H), 2.92 (s, 3H), 2.34 (s, 3H), 1.95 (s, 3H). **^{13}C NMR** (125MHz, Acetone-d_6): δ 156.80 , 154.35 , 131.53 , 131.50 , 130.81 , 127.81 , 126.45 , 126.25 , 124.33 , 122.43 , 121.93 , 118.63 , 59.32 , 39.41 , 15.65 , 12.60 . **HRMS (ESI)** m/z Calcd for [$C_{16}H_{16}Br_2NO_4S$, M - H]$^-$: 475.9173; Found: 475.9175.

Optical Rotation: $[\alpha]^{25}_D$ + 22.0 (c = 1.0, $CHCl_3$). The absolute configuration of **1j** was assigned by analogy to **1b**. 73% ee (HPLC condition: Chiralpak AD-H column, n-Hexane/i-PrOH = 90:10, flow rate = 1.0 mL/min, wavelength = 254 nm, t_R = 6.58 min for major isomer, t_R = 7.85 min for minor isomer).

Supplementary Figure 22. HPLC Trace of Recovered **1j**.

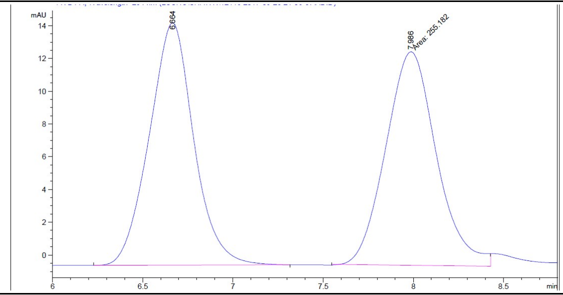

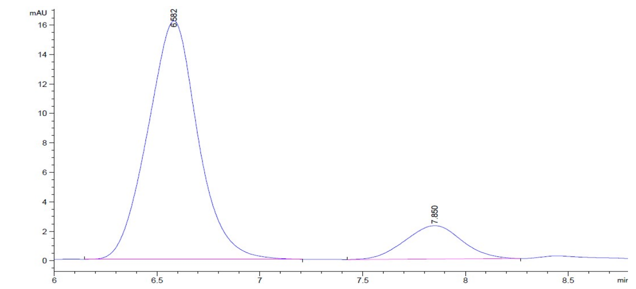

Peak #	RetTime [min]	Type	Width [min]	Area [mAU*s]	Height [mAU]	Area %
1	6.664	BB	0.2563	253.79059	14.78525	49.8633
2	7.986	MM	0.3264	255.18239	13.03028	50.1367

Peak #	RetTime [min]	Type	Width [min]	Area [mAU*s]	Height [mAU]	Area %
1	6.582	BB	0.2548	274.68600	16.19757	86.3502
2	7.850	BB	0.2960	43.42095	2.26197	13.6498

Product 4j

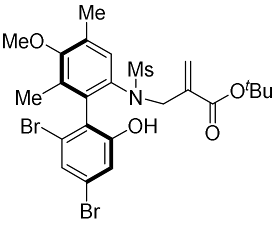

Syrup. **^{1}H NMR** (500 MHz, DMSO-d_6, 80 ºC): δ ^{1}H NMR (500 MHz, DMSO-d_6) δ 9.94 (br, 1H), 7.36 (s, 1H), 7.18 (s, 1H), 7.15 (s, 1H), 6.05 (s, 1H), 5.59 (s, 1H), 4.15 (s, 2H), 3.71 (s, 3H), 2.94 (s, 3H), 2.30 (s, 3H), 1.86 (s, 3H), 1.44 (s, 9H). **^{13}C NMR** (125MHz, DMSO-d_6, 80 ºC): δ 165.28 , 157.36 , 156.70 , 137.84 , 135.54 , 135.07 , 131.48 , 130.82 , 126.72 , 126.68 , 126.52 , 125.65 , 125.17 , 121.97 , 118.65 , 81.03 , 60.03 , 50.70 , 40.55 , 28.20 , 16.23 , 13.38 . **HRMS (ESI)** m/z Calcd for [$C_{24}H_{29}Br_2NNaO_6S$, M + Na]$^+$: 639.9974; Found: 639.9978.

Optical Rotation: $[\alpha]^{25}_{D}$ + 21.0 (c = 1.0, CH_2Cl_2). 83% ee (HPLC condition: Chiralpak As-H column, n-Hexane/i-PrOH = 90:10, flow rate = 1.0 mL/min, wavelength = 254 nm, t_R = 5.65 min for major isomer, t_R = 6.68 min for minor isomer).

Supplementary Figure 23. HPLC Trace of Product **4j**.

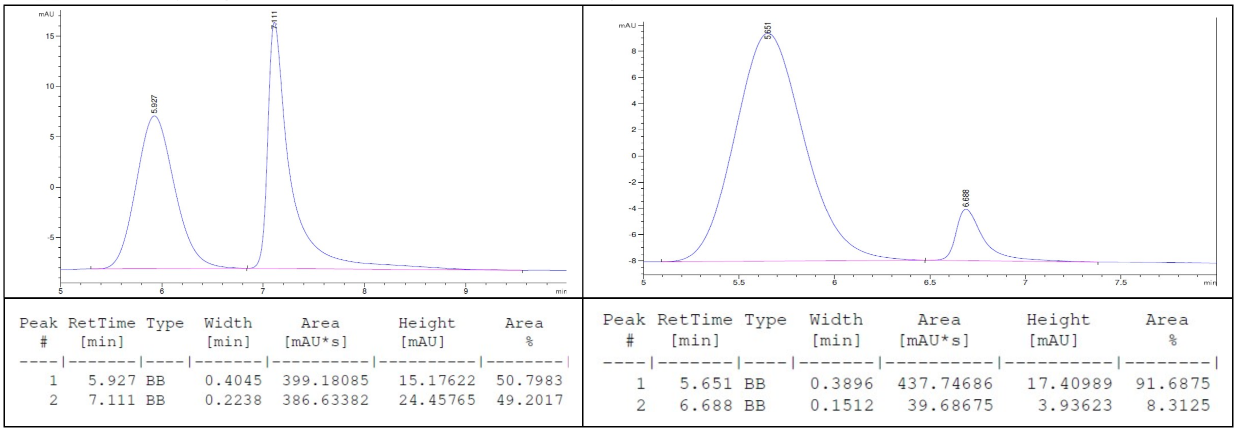

Peak #	RetTime [min]	Type	Width [min]	Area [mAU*s]	Height [mAU]	Area %
1	5.927	BB	0.4045	399.18085	15.17622	50.7983
2	7.111	BB	0.2238	386.63382	24.45765	49.2017

Peak #	RetTime [min]	Type	Width [min]	Area [mAU*s]	Height [mAU]	Area %
1	5.651	BB	0.3896	437.74686	17.40989	91.6875
2	6.688	BB	0.1512	39.68675	3.93623	8.3125

Recovered 1k

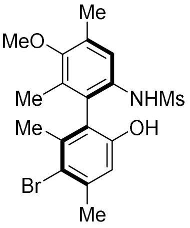

White solid. **Mp**: 229 – 231 ºC. **^{1}H NMR** (400 MHz, $CDCl_3$): δ 7.42 (s, 1H), 6.84 (s, 1H), 5.85 (s, 1H), 4.83 (s, 1H), 3.75 (s, 3H), 2.91 (s, 3H), 2.46 (s, 3H), 2.38 (s, 3H), 2.06 (s, 3H), 1.93 (s, 3H). **^{13}C NMR** (100MHz, $CDCl_3$): δ 154.65 , 151.58 , 140.61 , 137.62 , 133.09 , 132.09 , 131.34 , 124.58 , 120.34 , 120.26 , 119.74 , 116.00 , 60.11 , 39.78 , 24.20 , 20.82 , 16.52 , 13.28 . **HRMS (ESI)** m/z Calcd for [$C_{18}H_{21}$ $BrNO_4S$, M - H]$^-$: 426.0381; Found: 426.0384.

Optical Rotation: $[\alpha]^{25}_{D}$ - 17.0 (c = 1.0, $CHCl_3$). The absolute configuration of **1k** was assigned by analogy to **1b**. 84% ee (HPLC condition: Chiralpak AD-H column, n-Hexane/i-PrOH = 90:10, flow rate = 1.0 mL/min, wavelength = 254 nm, t_R = 6.26 min for major isomer, t_R = 11.56 min for minor isomer).

Supplementary Figure 24. HPLC Trace of Recovered **1k**.

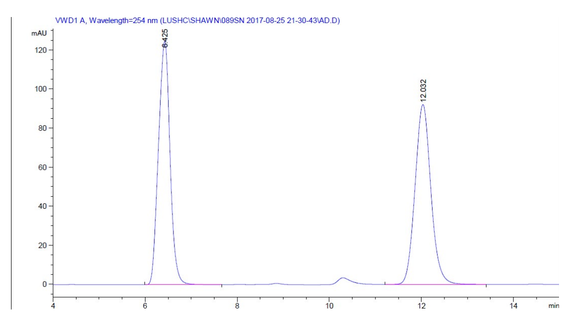

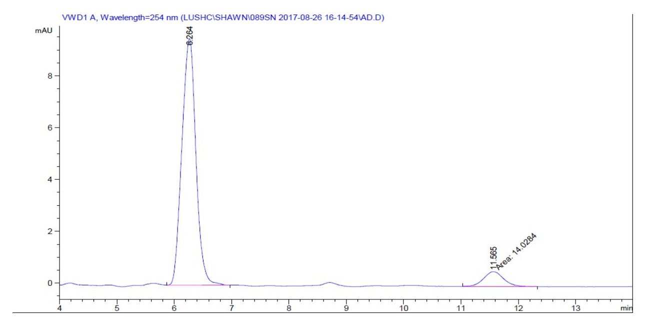

Peak #	RetTime [min]	Type	Width [min]	Area [mAU*s]	Height [mAU]	Area %
1	6.425	BB	0.2803	2185.35034	124.82055	49.7231
2	12.032	BB	0.3687	2209.68579	92.15477	50.2769

Peak #	RetTime [min]	Type	Width [min]	Area [mAU*s]	Height [mAU]	Area %
1	6.264	BB	0.2699	161.45512	9.51951	92.0059
2	11.565	MM	0.4086	14.02837	5.72159e-1	7.9941

Product 4k

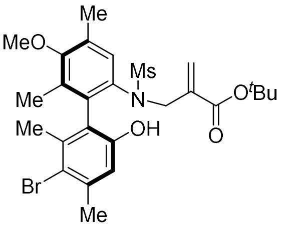

Syrup. **^{1}H NMR** (500 MHz, DMSO-d_6, 80 ºC): δ 8.95 (s, 1H), 7.05 (s, 1H), 6.82 (s, 1H), 6.06 (s, 1H), 5.64 (s, 1H), 4.18 (d, J = 16.5 Hz, 1H), 4.05 (d, J = 16.5 Hz, 1H), 3.71 (s, 3H), 2.87 (s, 3H), 2.39 (s, 3H), 2.30 (s, 3H), 1.97 (s, 3H), 1.81 (s, 3H), 1.43 (s, 9H). **^{13}C NMR** (125 MHz, DMSO-d_6, 80 ºC): δ 165.55 , 156.76 , 153.71 , 138.03 , 137.92 , 137.77 , 136.90 , 135.17 , 131.57 , 131.40 , 130.01 , 127.87 , 124.95 , 117.48 , 116.15 , 81.12 , 59.98 , 50.31 , 40.71 , 28.17 , 23.95 , 21.67 , 16.14 , 13.40 . **HRMS (ESI)** m/z Calcd for [$C_{26}H_{34}BrNNaO_6S$, M + Na]$^+$: 590.1182; Found: 590.1183.

Optical Rotation: $[\alpha]^{25}_{D}$ + 12.0 (c = 1.0, CH_2Cl_2). 66% ee (HPLC condition: Chiralpak AD-H column, n-Hexane/i-PrOH = 90:10, flow rate = 1.0 mL/min, wavelength = 254 nm, t_R = 6.28 min for minor isomer, t_R = 13.76 min for major isomer).

Supplementary Figure 25. HPLC Trace of Product **4k**.

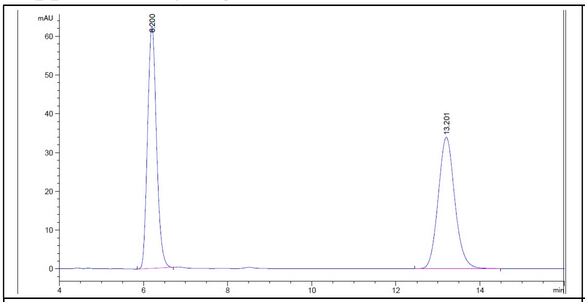

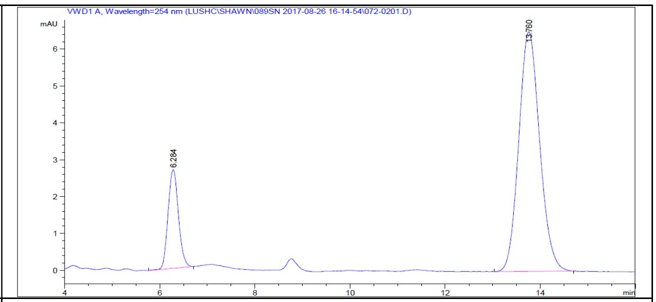

Peak #	RetTime [min]	Type	Width [min]	Area [mAU*s]	Height [mAU]	Area %
1	6.200	BB	0.2316	923.73944	62.44061	49.8660
2	13.201	BB	0.4208	928.70361	33.88350	50.1340

Peak #	RetTime [min]	Type	Width [min]	Area [mAU*s]	Height [mAU]	Area %
1	6.284	BB	0.2377	40.50815	2.67419	16.9589
2	13.760	BB	0.4643	198.35278	6.49481	83.0411

Recovered 1l

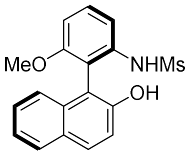

Syrup. **^{1}H NMR** (400 MHz, Acetone-d_6): δ 8.52 (br, 1H), 7.91 – 7.86 (m, 2H), 7.49 (t, J = 8.2 Hz, 1H), 7.42 (dd, J = 8.3, 1.1 Hz, 1H), 7.40 – 7.30 (m, 3H), 7.21 (ddd, J = 7.4, 2.2, 0.9 Hz, 1H), 7.01 (dd, J = 8.2, 1.2 Hz, 1H), 6.61 (br, 1H), 3.65 (s, 3H), 2.79 (s, 3H). **^{13}C NMR** (100MHz, Acetone-d_6): δ 158.77 , 152.72 , 137.80 , 133.81 , 130.31 , 129.79 , 129.07 , 128.23 , 126.70 , 123.81 , 123.19 , 118.47 , 115.69 , 112.46 , 112.29 , 107.51 , 55.22 , 38.88 . **HRMS (ESI)** m/z Calcd for [$C_{18}H_{16}NO_4S$, M - H]$^-$: 342.0806; Found: 342.0808.

Optical Rotation: $[\alpha]^{25}_{D}$ + 8.0 (c = 0.2, $CHCl_3$). The absolute configuration of **1l** was assigned by analogy to **1b**. 95% ee (HPLC condition: Chiralpak AD-H column, n-Hexane/i-PrOH = 90:10, flow rate = 1.0 mL/min, wavelength = 254 nm, t_R = 14.18 min for major isomer, t_R = 18.24 min for minor isomer).

Supplementary Figure 26. HPLC Trace of Recovered **1l**.

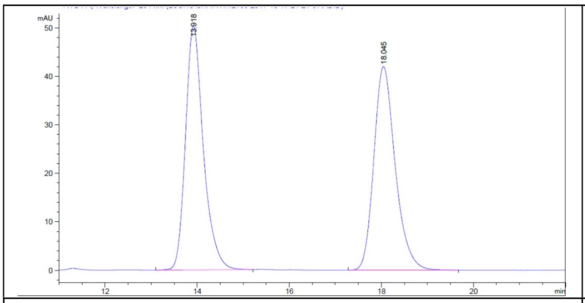

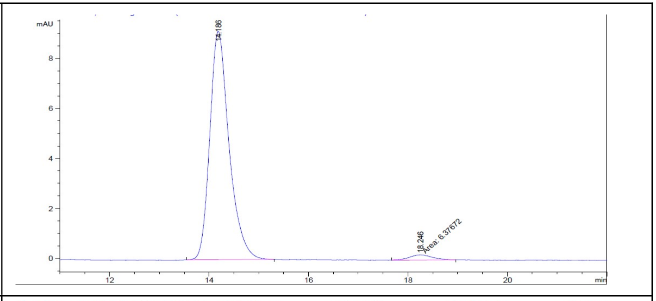

Peak #	RetTime [min]	Type	Width [min]	Area [mAU*s]	Height [mAU]	Area %
1	13.918	BB	0.4056	1355.11426	50.25290	50.0123
2	18.045	BB	0.4895	1354.44702	41.97271	49.9877

Peak #	RetTime [min]	Type	Width [min]	Area [mAU*s]	Height [mAU]	Area %
1	14.186	BB	0.4075	246.34785	9.13756	97.4768
2	18.246	MM	0.5218	6.37672	2.03658e-1	2.5232

Product 4l

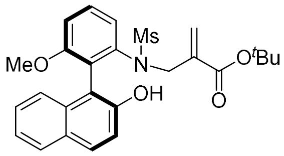

Syrup. **^{1}H NMR** (500 MHz, DMSO-d_6, 80 ºC) δ 9.01 (s, 1H), 7.81 (td, J = 7.4, 1.1 Hz, 2H), 7.45 (t, J = 8.2 Hz, 1H), 7.27 (d, J = 8.8 Hz, 1H), 7.26 – 7.21 (m, 2H), 7.16 (dd, J = 8.4, 1.1 Hz, 1H), 7.10 (ddd, J = 9.1, 7.4, 1.3 Hz, 2H), 5.95 (d, J = 1.4 Hz, 1H), 5.52 (d, J = 1.4 Hz, 1H), 4.12 (d, J = 16.5 Hz, 1H), 3.88 (d, J = 16.5 Hz, 1H), 3.58 (s, 3H), 2.53 (s, 3H), 1.39 (s, 9H). **^{13}C NMR** (125 MHz, DMSO-d_6, 80 ºC) δ 165.26 , 159.44 , 152.68 , 141.36 , 137.81 , 134.48 , 129.52 , 128.89 , 128.52 , 128.07 , 127.17 , 126.13 , 125.79 , 125.08 , 124.53 , 122.94 , 118.94 , 115.44 , 112.00 , 80.97 , 56.58 , 50.03 , 40.70 , 28.14 . **HRMS (ESI)** m/z Calcd for [$C_{26}H_{29}NNaO_6S$, M + Na]$^+$: 506.1608; Found: 506.1606.

Optical Rotation: $[\alpha]^{25}_D$ + 12.0 (c = 0.5, CH_2Cl_2). 68% ee (HPLC condition: Chiralpak OD-H column, n-Hexane/i-PrOH = 95:5, flow rate = 0.7 mL/min, wavelength = 254 nm, t_R = 22.15 min for minor isomer, t_R = 26.30 min for major isomer).

Supplementary Figure 27. HPLC Trace of Product **4l**.

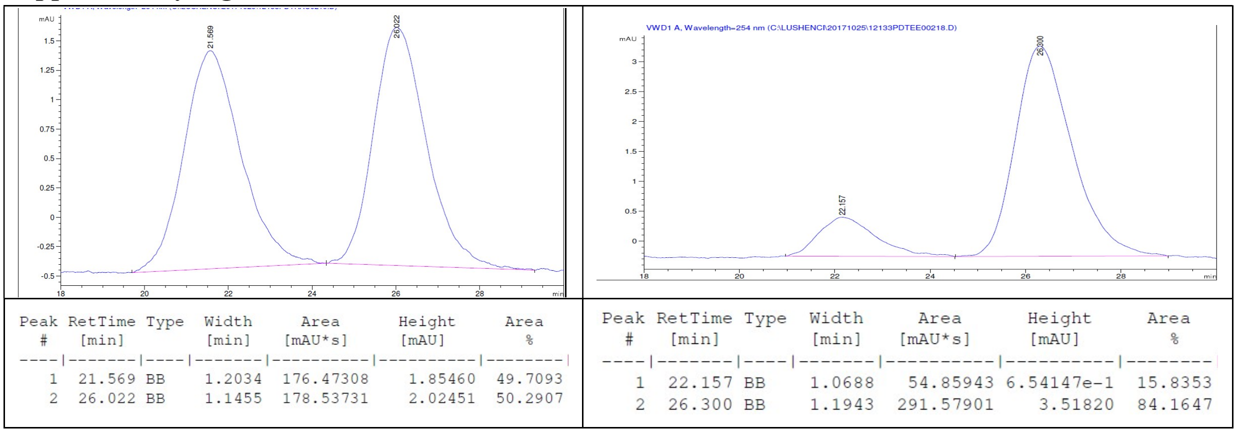

Peak #	RetTime [min]	Type	Width [min]	Area [mAU*s]	Height [mAU]	Area %
1	21.569	BB	1.2034	176.47308	1.85460	49.7093
2	26.022	BB	1.1455	178.53731	2.02451	50.2907

Peak #	RetTime [min]	Type	Width [min]	Area [mAU*s]	Height [mAU]	Area %
1	22.157	BB	1.0688	54.85943	6.54147e-1	15.8353
2	26.300	BB	1.1943	291.57901	3.51820	84.1647

Recovered 1m

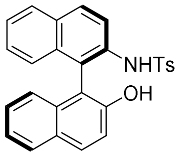

White solid. **MP**: 181 – 182 ºC. **^{1}H NMR** (500 MHz, $CDCl_3$) δ 8.15 (d, J = 9.0 Hz, 1H), 8.02 (d, J = 9.1 Hz, 1H), 7.99 (d, J = 9.0 Hz, 1H), 7.91 (d, J = 8.2 Hz, 2H), 7.48 (d, J = 8.2 Hz, 2H), 7.45 – 7.41 (m, 1H), 7.38 (ddd, J = 8.1, 7.0, 1.1 Hz, 1H), 7.32 (d, J = 8.9 Hz, 1H), 7.29 – 7.25 (m, 1H), 7.16 – 7.10 (m, 3H), 7.05 (d, J = 8.5 Hz, 1H), 6.66 (d, J = 8.5 Hz, 1H), 6.50 (br, 1H), 4.62 (br, 1H), 2.37 (s, 3H). **^{13}C NMR** (125 MHz, $CDCl_3$) δ 151.87 , 144.09 , 135.85 , 134.67 , 132.96 , 131.56 , 131.14 , 130.66 , 129.67 , 129.31 , 128.45 , 128.27 , 127.61 , 127.51 , 127.23 , 125.65 , 125.26 , 123.96 , 123.72 , 119.12 , 118.26 , 117.80 , 111.89 , 21.56 . **HRMS (ESI)** m/z Calcd for [$C_{27}H_{21}NO_3S$, M - H]$^-$: 438.1170; Found: 438.1174.

Optical Rotation: $[\alpha]^{25}_D$ + 30.0 (c = 0.5, CH_2Cl_2). The absolute configuration of **1m** was assigned by analogy to **1b**. 98% ee (HPLC condition: Chiralcel AD-H column, n-Hexane/i-PrOH =

80:20, flow rate = 1.0 mL/min, wavelength = 254 nm, t_R = 7.11 min for minor isomer, t_R = 7.83 min for major isomer).

Supplementary Figure 28. HPLC Trace of Recovered **1m**.

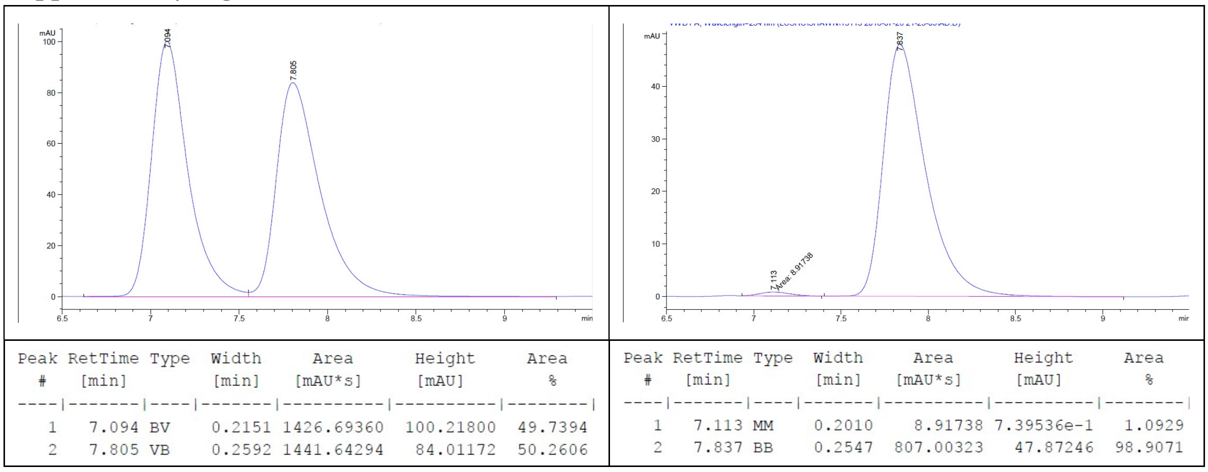

Peak #	RetTime [min]	Type	Width [min]	Area [mAU*s]	Height [mAU]	Area %
1	7.094	BV	0.2151	1426.69360	100.21800	49.7394
2	7.805	VB	0.2592	1441.64294	84.01172	50.2606

Peak #	RetTime [min]	Type	Width [min]	Area [mAU*s]	Height [mAU]	Area %
1	7.113	MM	0.2010	8.91738	7.39536e-1	1.0929
2	7.837	BB	0.2547	807.00323	47.87246	98.9071

Product 4m

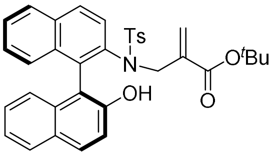

Syrup. **^{1}H NMR** (500 MHz, DMSO-d_6, 80 °C) δ 9.13 (br, 1H), 8.02 (dd, *J* = 9.0, 2.1 Hz, 2H), 7.92 (d, *J* = 8.9 Hz, 1H), 7.89 (d, *J* = 8.1 Hz, 1H), 7.56 – 7.46 (m, 2H), 7.34 – 7.26 (m, 3H), 7.23 – 7.18 (m, 2H), 7.15 – 7.05 (m, 4H), 6.90 (d, *J* = 8.5 Hz, 1H), 5.79 (s, 1H), 5.21 (s, 1H), 4.25 (d, *J* = 16.5 Hz, 1H), 3.96 (d, *J* = 16.4 Hz, 1H), 2.34 (s, 3H), 1.37 (s, 9H). **^{13}C NMR** (125 MHz, DMSO-d_6, 80 °C) δ 165.20 , 153.16 , 143.62 , 138.33 , 137.15 , 134.70 , 134.39 , 134.14 , 133.04 , 130.04 , 129.74 , 129.55 , 129.22 , 128.62 , 128.32 , 128.26 , 128.17 , 128.07 , 127.58 , 126.81 , 126.78 , 126.70 , 126.36 , 125.63 , 123.08 , 118.82 , 116.35 , 80.87 , 50.52 , 28.14 , 21.31 . **HRMS (ESI)** m/z Calcd for [$C_{35}H_{33}NNaO_5S$, M + Na]$^+$: 602.1971; Found: 602.1975.

Optical Rotation: $[\alpha]^{25}_D$ - 36.0 (*c* = 1.0, CH_2Cl_2). 71% ee (HPLC condition: Chiralcel AD-H column, *n*-Hexane/*i*-PrOH = 80:20, flow rate = 1.0 mL/min, wavelength = 254 nm, t_R = 17.40 min for major isomer, t_R = 21.82 min for minor isomer).

Supplementary Figure 29. HPLC Trace of Product **4m**.

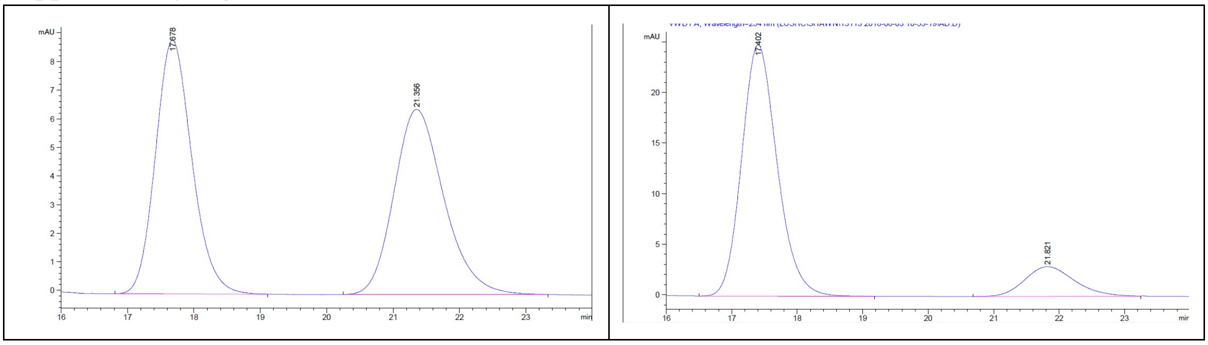

Peak #	RetTime [min]	Type	Width [min]	Area [mAU*s]	Height [mAU]	Area %
1	17.678	BB	0.5827	338.86722	8.87109	50.0496
2	21.356	BB	0.7838	338.19522	6.46997	49.9504

Peak #	RetTime [min]	Type	Width [min]	Area [mAU*s]	Height [mAU]	Area %
1	17.402	BB	0.5782	934.78473	24.83263	85.6904
2	21.821	BV	0.7811	156.10153	2.93678	14.3096

Recovered 1n

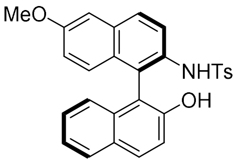

White solid. **MP**: 201– 202 ºC. **^{1}H NMR** (500 MHz, $CDCl_3$) δ 8.10 (d, J = 9.0 Hz, 1H), 7.96 (d, J = 8.9 Hz, 1H), 7.90 (t, J = 8.7 Hz, 2H), 7.43 (d, J = 8.2 Hz, 2H), 7.36 (ddd, J = 8.1, 6.7, 1.1 Hz, 1H), 7.29 (d, J = 9.1 Hz, 1H), 7.20 (d, J = 2.2 Hz, 1H), 7.11 (ddd, J = 8.3, 6.8, 1.3 Hz, 1H), 7.07 (d, J = 8.0 Hz, 2H), 6.96 – 6.91 (m, 2H), 6.64 (d, J = 8.4 Hz, 1H), 6.43 (br, 1H), 4.75 (br, 1H), 3.91 (s, 3H), 2.36 (s, 3H). **^{13}C NMR** (125 MHz, $CDCl_3$) δ 157.55 , 151.76 , 143.94 , 135.87 , 132.99 , 132.57 , 132.50 , 131.42 , 129.61 , 129.24 , 129.21 , 128.40 , 128.19 , 127.46 , 127.15 , 127.00 , 123.87 , 123.75 , 120.43 , 120.17 , 119.34 , 117.78 , 112.22 , 106.35 , 55.40 , 29.73 . **HRMS (ESI)** m/z Calcd for [$C_{28}H_{23}NO_4S$, M - H]$^-$: 468.1276; Found: 468.1272.

Optical Rotation: $[\alpha]^{25}_{D}$ + 30.0 (c = 0.5, CH_2Cl_2). The absolute configuration of **1n** was assigned by analogy to **1b**. 99% ee (HPLC condition: Chiralcel AD-H column, n-Hexane/i-PrOH = 80:20, flow rate = 1.0 mL/min, wavelength = 254 nm, t_R = 8.37 min for minor isomer, t_R = 10.35 min for major isomer).

Supplementary Figure 30. HPLC Trace of Recovered **1n**.

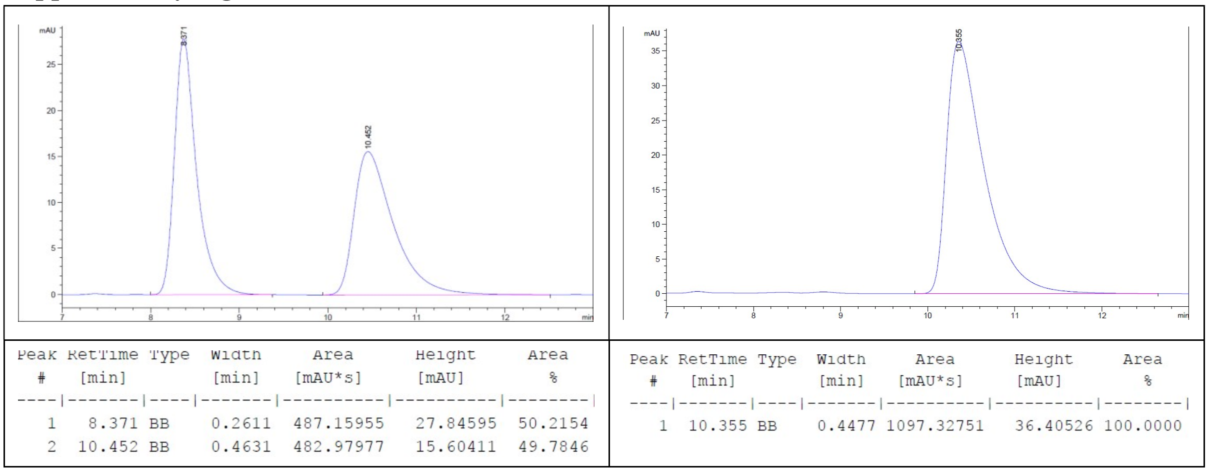

Peak #	RetTime [min]	Type	Width [min]	Area [mAU*s]	Height [mAU]	Area %
1	8.371	BB	0.2611	487.15955	27.84595	50.2154
2	10.452	BB	0.4631	482.97977	15.60411	49.7846

Peak #	RetTime [min]	Type	Width [min]	Area [mAU*s]	Height [mAU]	Area %
1	10.355	BB	0.4477	1097.32751	36.40526	100.0000

Product 4n

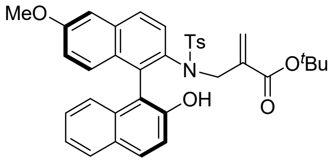

Syrup. **^{1}H NMR** (500 MHz, DMSO-d_6, 80 °C) δ 9.07 (br, 1H), 7.93 – 7.84 (m, 3H), 7.44 – 7.39 (m, 2H), 7.31 (d, J = 8.9 Hz, 1H), 7.27 (ddd, J = 8.1, 6.8, 1.2 Hz, 1H), 7.20 (d, J = 7.9 Hz, 2H), 7.13 (ddd, J = 8.4, 6.8, 1.4 Hz, 1H), 7.09 (d, J = 8.0 Hz, 2H), 6.99 – 6.93 (m, 2H), 6.93 – 6.88 (m, 1H), 5.79 (d, J = 1.4 Hz, 1H), 5.20 (d, J = 1.4 Hz, 1H), 4.23 (d, J = 16.4 Hz, 1H), 3.96 (dd, J = 16.4, 1.2 Hz, 1H), 3.91 (s, 3H), 2.34 (s, 3H), 1.38 (s, 9H). **^{13}C NMR** (125 MHz, DMSO-d_6, 80 °C) δ 165.23 , 158.36 , 153.07 , 143.54 , 137.18 , 136.17 , 134.73 , 134.49 , 134.39 , 129.96 , 129.71 , 129.52 , 129.38 , 128.58 , 128.38 , 128.14 , 128.03 , 127.56 , 127.20 , 126.74 , 126.32 , 125.68 , 123.06 , 119.09 , 118.80 , 116.53 , 107.21 , 80.85 , 55.88 , 50.54 , 28.14 , 21.31 . **HRMS (ESI)** m/z Calcd for $[C_{36}H_{35}NNaO_6S, M + Na]^+$: 632.2077; Found: 632.2072.

Optical Rotation: $[\alpha]^{25}_D$ - 55.0 (c = 1.0, CH_2Cl_2). 68% ee (HPLC condition: Chiralcel AD-H column, n-Hexane/i-PrOH = 80:20, flow rate = 1.0 mL/min, wavelength = 254 nm, t_R = 16.42 min for major isomer, t_R = 30.40 min for minor isomer).

Supplementary Figure 31. HPLC Trace of Product **4n**.

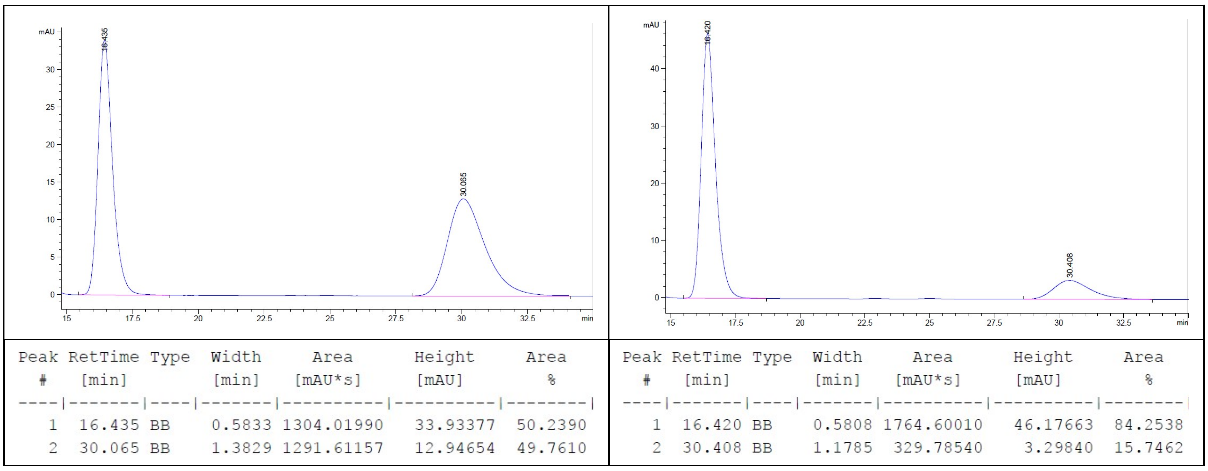

Peak #	RetTime [min]	Type	Width [min]	Area [mAU*s]	Height [mAU]	Area %
1	16.435	BB	0.5833	1304.01990	33.93377	50.2390
2	30.065	BB	1.3829	1291.61157	12.94654	49.7610

Peak #	RetTime [min]	Type	Width [min]	Area [mAU*s]	Height [mAU]	Area %
1	16.420	BB	0.5808	1764.60010	46.17663	84.2538
2	30.408	BB	1.1785	329.78540	3.29840	15.7462

Recovered 1o

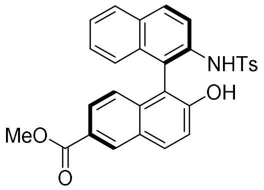

White solid. **MP**: 224 – 225 °C. **^{1}H NMR** (500 MHz, $CDCl_3$) δ 8.63 (d, J = 1.6 Hz, 1H), 8.16 (d, J = 9.0 Hz, 1H), 8.08 (d, J = 8.9 Hz, 1H), 8.04 (d, J = 9.0 Hz, 1H), 7.96 – 7.88 (m, 1H), 7.60 (dd, J = 8.8, 1.7 Hz, 1H), 7.48 – 7.37 (m, 4H), 7.29 – 7.24 (m, 1H), 7.06 (d, J = 8.0 Hz, 2H), 7.02 – 6.94 (m, 1H), 6.57 (d, J = 8.8 Hz, 1H), 6.47 (br, 1H), 5.12 (br, 1H), 3.99 (s, 3H), 2.36 (s, 3H). **^{13}C NMR** (125 MHz, $CDCl_3$) δ 167.07 , 153.97 , 144.30 , 135.87 , 135.59 , 134.68 , 132.95 , 132.85 , 131.36 , 131.20 , 130.92 , 129.68 ,

128.37 , 128.25 , 127.73 , 127.07 , 126.86 , 125.78 , 125.42 , 125.07 , 123.90 , 119.41 , 118.78 , 117.89 , 112.38 , 52.25 , 21.48 . **HRMS (ESI)** m/z Calcd for $[C_{29}H_{23}NO_5S, M - H]^-$: 496.1225; Found: 496.1228.

Optical Rotation: $[\alpha]^{25}_D$ + 15.0 (*c* = 0.5, CH_2Cl_2). The absolute configuration of **1o** was assigned by analogy to **1b**. 97% ee (HPLC condition: Chiralcel AD-H column, *n*-Hexane/*i*-PrOH = 80:20, flow rate = 1.0 mL/min, wavelength = 254 nm, t_R = 8.74 min for major isomer, t_R = 11.59 min for minor isomer).

Supplementary Figure 32. HPLC Trace of Recovered **1o**.

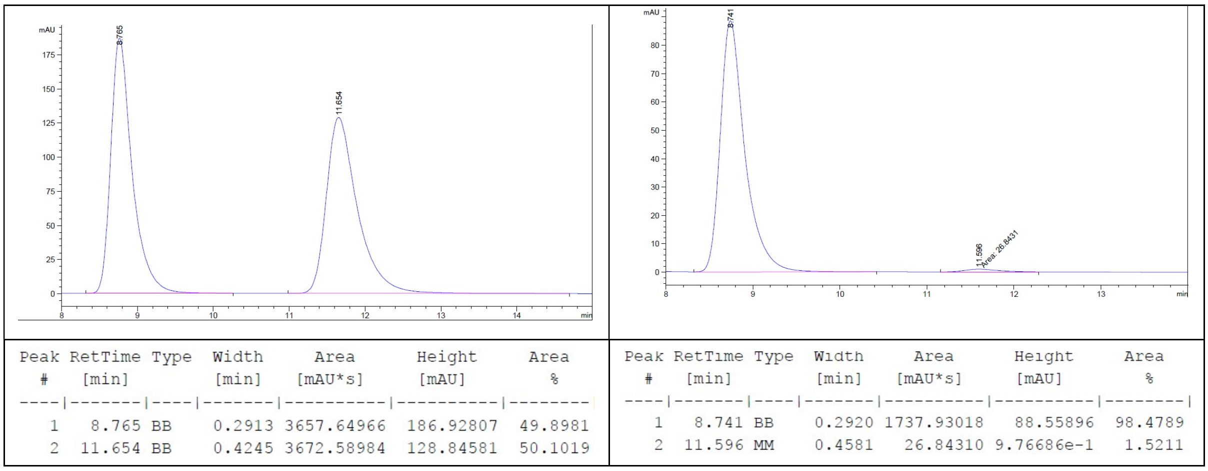

Peak #	RetTime [min]	Type	Width [min]	Area [mAU*s]	Height [mAU]	Area %
1	8.765	BB	0.2913	3657.64966	186.92807	49.8981
2	11.654	BB	0.4245	3672.58984	128.84581	50.1019

Peak #	RetTime [min]	Type	Width [min]	Area [mAU*s]	Height [mAU]	Area %
1	8.741	BB	0.2920	1737.93018	88.55896	98.4789
2	11.596	MM	0.4581	26.84310	9.76686e-1	1.5211

Product 4o

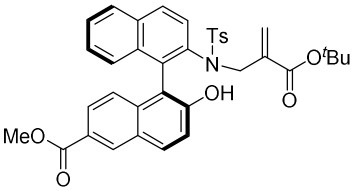

Syrup. **^{1}H NMR** (500 MHz, DMSO-d_6, 80 ºC) δ 9.67 (br, 1H), 8.58 (d, *J* = 1.7 Hz, 1H), 8.12 (d, *J* = 8.9 Hz, 1H), 8.03 (d, *J* = 8.6 Hz, 2H), 7.58 (dd, *J* = 9.0, 1.8 Hz, 1H), 7.56 – 7.52 (m, 1H), 7.46 (d, *J* = 8.8 Hz, 1H), 7.42 (d, *J* = 8.9 Hz, 1H), 7.34 – 7.29 (m, 1H), 7.26 (d, *J* = 8.0 Hz, 2H), 7.12 (d, *J* = 8.0 Hz, 2H), 7.03 (d, *J* = 8.5 Hz, 1H), 6.91 (d, *J* = 8.9 Hz, 1H), 5.79 (s, 1H), 5.23 (s, 1H), 4.27 (d, *J* = 16.5 Hz, 1H), 4.03 (d, *J* = 16.6 Hz, 1H), 3.90 (s, 3H), 2.34 (s, 3H), 1.36 (s, 9H). **^{13}C NMR** (125 MHz, DMSO-d_6, 80 ºC) δ 166.97 , 165.11 , 155.67 , 143.68 , 138.45 , 137.25 , 137.13 , 133.94 , 133.92 , 133.06 , 131.78 , 130.95 , 129.60 , 129.13 , 128.68 , 128.36 , 128.02 , 127.47 , 126.99 , 126.93 , 126.51 , 125.97 , 125.30 , 124.45 , 119.79 , 116.56 , 80.91 , 52.20 , 50.92 , 28.10 , 21.29 . **HRMS (ESI)** m/z Calcd for $[C_{37}H_{35}NNaO_7S, M + Na]^+$: 660.2026; Found: 660.2022.

Optical Rotation: $[\alpha]^{25}_D$ - 42.0 (*c* = 1.0, CH_2Cl_2). 73% ee (HPLC condition: Chiralcel IA column, *n*-Hexane/*i*-PrOH = 80:20, flow rate = 1.0 mL/min, wavelength = 254 nm, t_R = 17.16 min for minor isomer, t_R = 19.11 min for major isomer).

Supplementary Figure 33. HPLC Trace of Product **4o**.

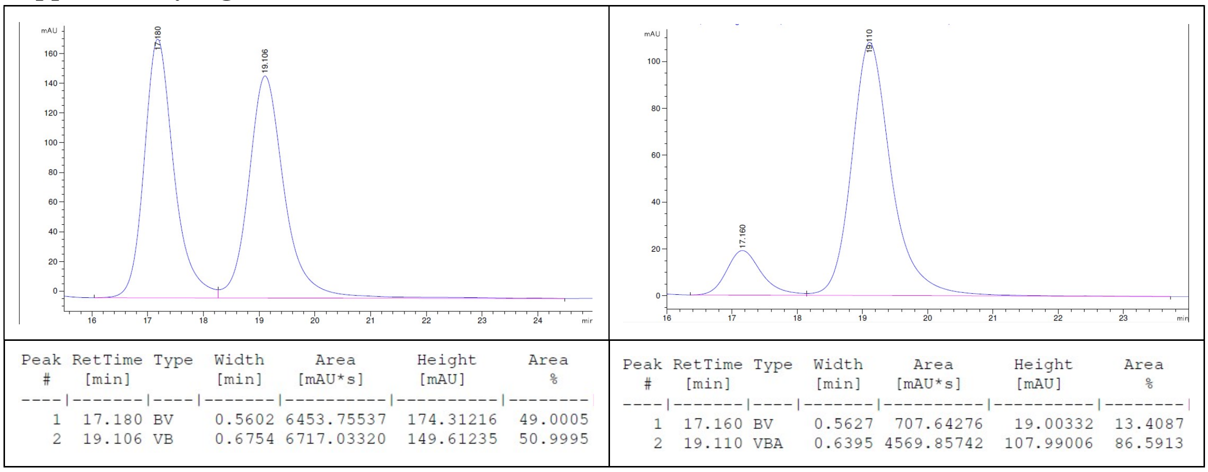

Peak #	RetTime [min]	Type	Width [min]	Area [mAU*s]	Height [mAU]	Area %
1	17.180	BV	0.5602	6453.75537	174.31216	49.0005
2	19.106	VB	0.6754	6717.03320	149.61235	50.9995

Peak #	RetTime [min]	Type	Width [min]	Area [mAU*s]	Height [mAU]	Area %
1	17.160	BV	0.5627	707.64276	19.00332	13.4087
2	19.110	VBA	0.6395	4569.85742	107.99006	86.5913

Recovered 1p

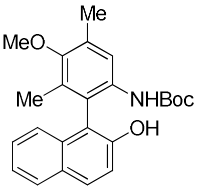

White solid. **MP**: 197 – 198 ºC. **^{1}H NMR** (500 MHz, $CDCl_3$): δ 7.92 – 7.86 (m, 3H), 7.38 (qdd, *J* = 6.8, 4.1, 1.8 Hz, 2H), 7.33 (dd, *J* = 8.9, 1.5 Hz, 1H), 7.21 – 7.15 (m, 1H), 5.91 (s, 1H), 5.30 (br, 1H), 3.77 (s, 3H), 2.44 (s, 3H), 1.85 (s, 3H), 1.36 (s, 9H). **^{13}C NMR** (125 MHz, $CDCl_3$): δ 153.61 , 153.43 , 151.23 , 133.55 , 132.76 , 132.59 , 132.11 , 130.54 , 129.28 , 128.33 , 127.21 , 123.80 , 123.75 , 121.57 , 121.13 , 117.79 , 114.76 , 80.52 , 60.13 , 28.16 , 16.59 , 13.12 . **HRMS (ESI)** m/z Calcd for $[C_{24}H_{26}NO_4$, M - H$]^-$: 392.1868; Found: 392.1866.

Optical Rotation: $[\alpha]^{25}_{D}$ + 20.0 (*c* = 0.4, $CHCl_3$). 19% ee (HPLC condition: Chiralpak ID column, *n*-Hexane/*i*-PrOH = 95:5, flow rate = 1.0 mL/min, wavelength = 254 nm, t_R = 5.62 min for major isomer, t_R = 6.21 min for minor isomer).

Supplementary Figure 34. HPLC Trace of Recovered **1p**.

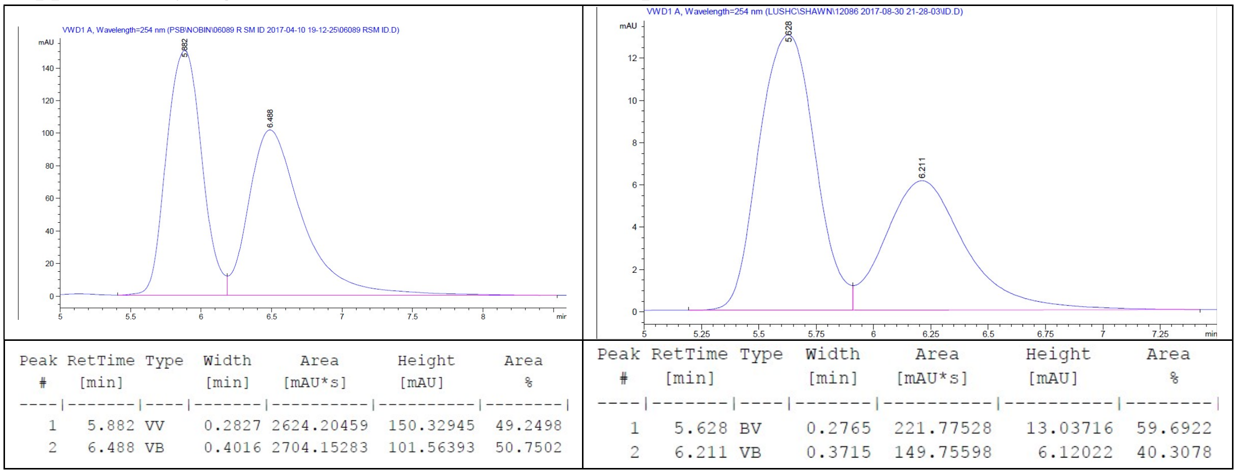

Peak #	RetTime [min]	Type	Width [min]	Area [mAU*s]	Height [mAU]	Area %
1	5.882	VV	0.2827	2624.20459	150.32945	49.2498
2	6.488	VB	0.4016	2704.15283	101.56393	50.7502

Peak #	RetTime [min]	Type	Width [min]	Area [mAU*s]	Height [mAU]	Area %
1	5.628	BV	0.2765	221.77528	13.03716	59.6922
2	6.211	VB	0.3715	149.75598	6.12022	40.3078

Product 4p

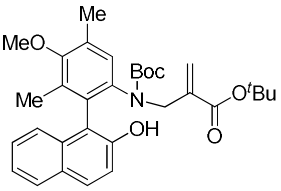

Syrup. ^{1}**H NMR** (500 MHz, DMSO-d_6, 80 ºC): δ 8.04 (d, J = 9.0 Hz, 1H), 7.97 – 7.91 (m, 1H), 7.56 – 7.52 (m, 2H), 7.38 (dddd, J = 13.7, 6.8, 5.2, 1.5 Hz, 2H), 7.17 – 7.11 (m, 1H), 6.28 (s, 1H), 6.04 (s, 1H), 5.55 (s, 1H), 4.86 – 4.71 (m, 2H), 3.70 (s, 3H), 2.35 (s, 3H), 1.74 (s, 3H), 1.45 (s, 9H), 1.23 (s, 9H). ^{13}**C NMR** (125 MHz, DMSO-d_6, 80 ºC): δ 164.49 , 153.70 , 153.66 , 153.11 , 138.44 , 133.05 , 132.72 , 130.49 , 129.85 , 129.66 , 128.54 , 127.35 , 126.98 , 125.22 , 124.35 , 124.34 , 122.40 , 119.85 , 116.06 , 81.24 , 79.44 , 67.81 , 60.01 , 28.35 , 28.18 , 16.46 , 13.53 . **HRMS (ESI)** m/z Calcd for [$C_{32}H_{39}NNaO_6$, M + Na]$^+$: 556.2669; Found: 556.2667.

Optical Rotation: $[\alpha]^{25}_{D}$ + 4.0 (c = 1.0, CH_2Cl_2). 6% ee (HPLC condition: Chiralpak ID column, n-Hexane/i-PrOH = 95:5, flow rate = 1.0 mL/min, wavelength = 254 nm, t_R = 5.46 min for major isomer, t_R = 6.18 min for minor isomer).

Supplementary Figure 35. HPLC Trace of Product **4p**.

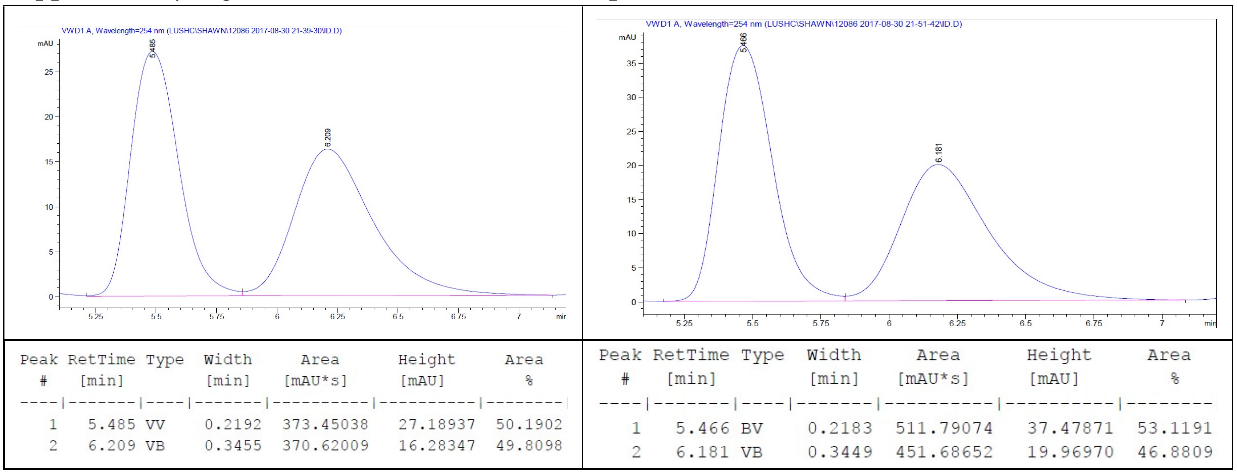

Peak #	RetTime [min]	Type	Width [min]	Area [mAU*s]	Height [mAU]	Area %
1	5.485	VV	0.2192	373.45038	27.18937	50.1902
2	6.209	VB	0.3455	370.62009	16.28347	49.8098

Peak #	RetTime [min]	Type	Width [min]	Area [mAU*s]	Height [mAU]	Area %
1	5.466	BV	0.2183	511.79074	37.47871	53.1191
2	6.181	VB	0.3449	451.68652	19.96970	46.8809

Recovered 1q

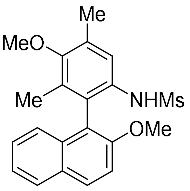

Syrup. ^{1}**H NMR** (400 MHz, CD_2Cl_2): δ 7.93 (dd, J = 9.1, 0.8 Hz, 1H), 7.84 – 7.78 (m, 1H), 7.36 (d, J = 9.1 Hz, 1H), 7.34 – 7.32 (m, 1H), 7.32 – 7.25 (m, 2H), 7.10 – 7.05 (m, 1H), 5.64 (br, 1H), 3.79 (s, 3H), 3.67 (s, 3H), 2.49 (s, 3H), 2.32 (s, 3H), 1.70 (s, 3H). ^{13}**C NMR** (75 MHz, CD_2Cl_2): δ 154.66 , 154.10 , 132.73 , 131.70 , 131.40 , 131.05 , 130.73 , 129.22 , 128.35 , 127.42 , 126.93 , 124.00 , 123.51 , 121.22 , 117.39 , 113.08 , 59.76 , 56.05 , 39.03 , 16.09 , 13.05 . **HRMS (ESI)** m/z Calcd for [$C_{21}H_{23}NNaO_4S$, M + Na]$^+$: 408.1240; Found: 408.1242.

Optical Rotation: $[\alpha]^{25}_{D}$ + 2.4 (c = 0.5, $CHCl_3$). 13% ee (HPLC condition: Chiralpak ID column,

n-Hexane/*i*-PrOH = 80:20, flow rate = 1.0 mL/min, wavelength = 254 nm, t_R = 11.19 min for major isomer, t_R = 12.56 min for minor isomer).

Supplementary Figure 36. HPLC Trace of Recovered **1q**.

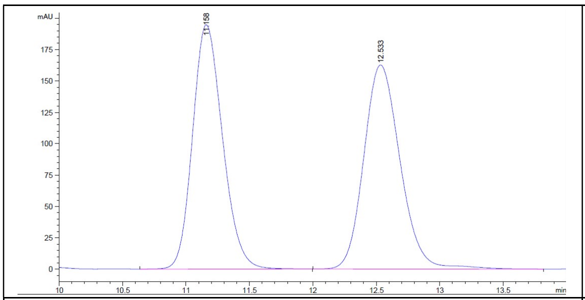

Peak #	RetTime [min]	Type	Width [min]	Area [mAU*s]	Height [mAU]	Area %
1	11.158	BB	0.2546	3194.81519	194.53256	49.6784
2	12.533	BB	0.3082	3236.18481	162.57556	50.3216

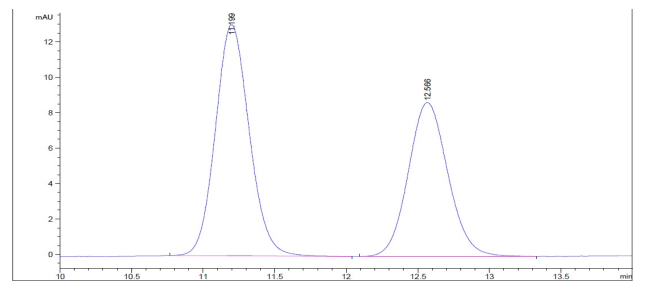

Peak #	RetTime [min]	Type	Width [min]	Area [mAU*s]	Height [mAU]	Area %
1	11.199	BB	0.2541	214.82658	13.04622	56.2575
2	12.566	BB	0.2975	167.03668	8.67994	43.7425

Product 4q

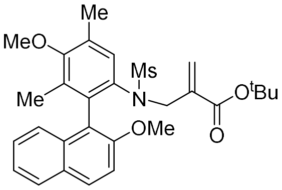

Syrup. **^{1}H NMR** (500 MHz, DMSO-d_6, 80 ºC): δ 8.03 (d, *J* = 9.1 Hz, 1H), 7.95 – 7.88 (m, 1H), 7.54 (d, *J* = 9.0 Hz, 1H), 7.37 – 7.27 (m, 2H), 7.16 (s, 1H), 7.13 (d, *J* = 8.5 Hz, 1H), 5.94 (s, 1H), 5.45 (s, 1H), 4.00 (d, *J* = 16.8 Hz, 1H), 3.87 – 3.78 (m, 4H), 3.74 (s, 3H), 2.42 (s, 3H), 2.36 (s, 3H), 1.70 (s, 3H), 1.40 (s, 9H). **^{13}C NMR** (125MHz, DMSO-d_6, 80 ºC): δ 165.32 , 156.76 , 154.19 , 137.67 , 135.95 , 135.34 , 133.40 , 131.96 , 131.93 , 130.26 , 130.14 , 129.07 , 128.28 , 126.89 , 126.76 , 125.15 , 123.93 , 120.58 , 114.04 , 80.98 , 60.06 , 56.26 , 50.43 , 40.71 , 28.13 , 16.24 , 13.42 . **HRMS (ESI)** m/z Calcd for [$C_{29}H_{35}NNaO_6S$, M + Na]$^+$: 548.2077; Found: 548.2075.

Optical Rotation: $[\alpha]^{25}_D$ – 3.4 (*c* = 0.5, CH_2Cl_2). 18% ee (HPLC condition: Chiralpak AD-H column, *n*-Hexane/*i*-PrOH = 80:20, flow rate = 1.0 mL/min, wavelength = 254 nm, t_R = 6.08 min for minor isomer, t_R = 7.43 min for major isomer).

Supplementary Figure 37. HPLC Trace of Product **4q**.

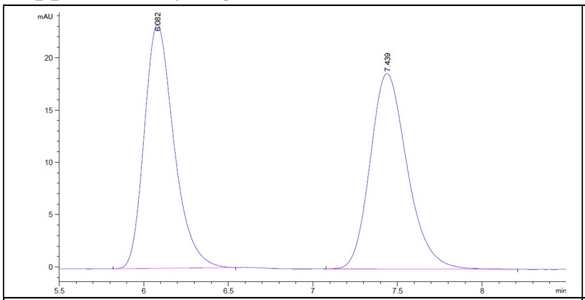

Peak #	RetTime [min]	Type	Width [min]	Area [mAU*s]	Height [mAU]	Area %
1	6.082	BB	0.1862	283.83948	23.28338	49.8917
2	7.439	BB	0.2349	285.07138	18.69304	50.1083

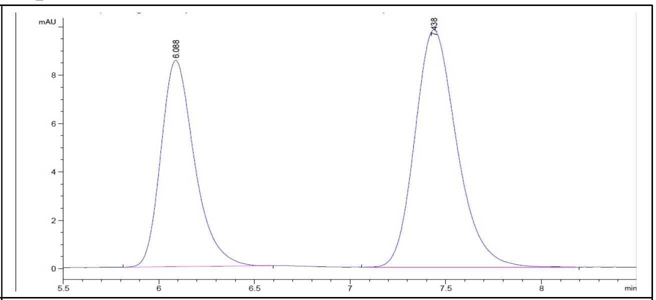

Peak #	RetTime [min]	Type	Width [min]	Area [mAU*s]	Height [mAU]	Area %
1	6.088	BB	0.1879	104.39327	8.52537	41.0949
2	7.438	BB	0.2330	149.63657	9.80908	58.9051

Derivatization to access more NOBINs analogs and application to ATH:

Deallylation followed by kinetic resolution to access (*S*)-1b

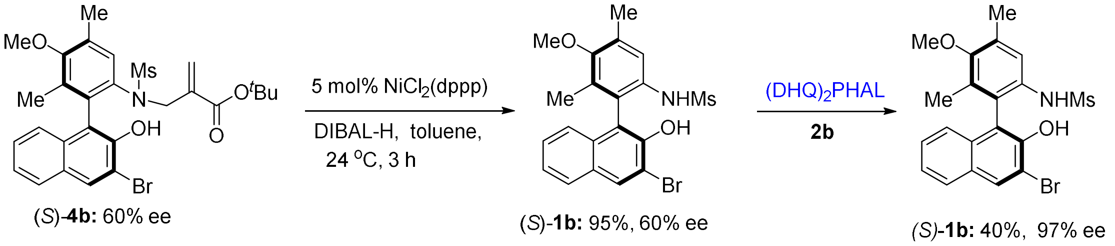

The deallylaltion step followed a literature procedure.[6] To a solution of (*S*)-**4b** (0.10 mmol) and $NiCl_2$(dppp) (5 mol %, 0.005 mmol) in toluene (1.0 mL) was added a 1M solution of diisobutylaluminum hydride in toluene (1.0 mL, 1.0 mmol) at 0 °C, and the reaction mixture was stirred for 3 h at 24 °C. The mixture was quenched with 1 M aqueous NaOH (1.0 mL), and the mixture was diluted with water (20 mL). The solution was extracted with ethyl acetate (3 × 15 mL). The combined extracts were dried over Na_2SO_4, and the resulting crude material was purified by thin layer chromatography on silica gel (hexane/ethyl acetate = 3/1 as eluent) to give (*S*)-**1b** (95% yield).

The following kinetic resolution followed our standard procedure using $(DHQ)_2PHAL$ as the catalyst. The unreacted (*S*)-**1b** was recovered in 40% yield with 97% ee.

Optical Rotation: $[\alpha]^{25}_{D}$ – 12.0 (*c* = 0.5, $CHCl_3$). 97% ee (HPLC condition: Chiralpak AD-H column, *n*-Hexane/*i*-PrOH = 90:10, flow rate = 1.0 mL/min, wavelength = 254 nm, t_R = 11.46 min for minor isomer, t_R = 12.64 min for major isomer).

Supplementary Figure 38. HPLC Trace of (*S*)-**1b**.

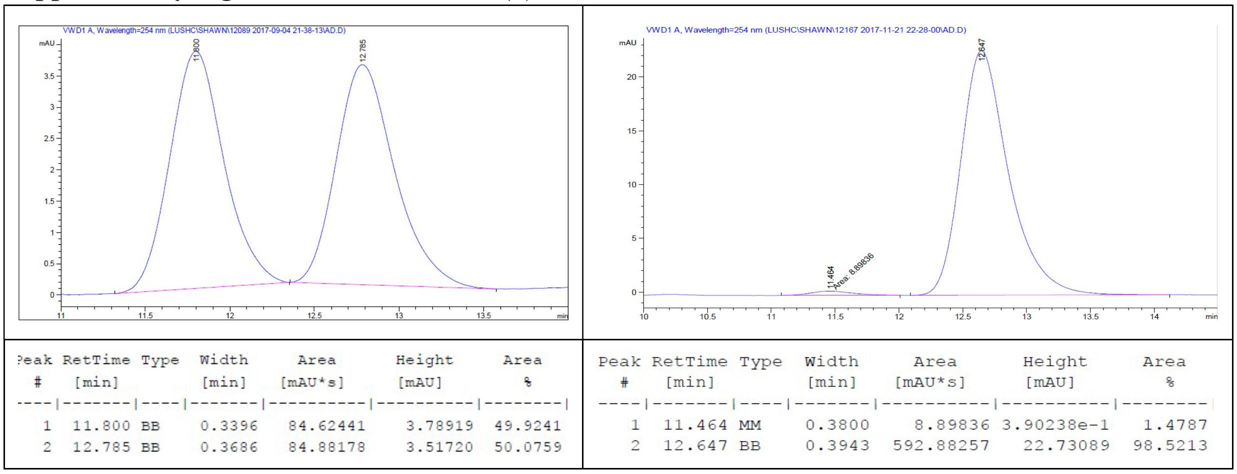

Peak #	RetTime [min]	Type	Width [min]	Area [mAU*s]	Height [mAU]	Area %
1	11.800	BB	0.3396	84.62441	3.78919	49.9241
2	12.785	BB	0.3686	84.88178	3.51720	50.0759

Peak #	RetTime [min]	Type	Width [min]	Area [mAU*s]	Height [mAU]	Area %
1	11.464	MM	0.3800	8.89836	3.90238e-1	1.4787
2	12.647	BB	0.3943	592.88257	22.73089	98.5213

General procedure for Pd-catalyzed Suzuki cross coupling:

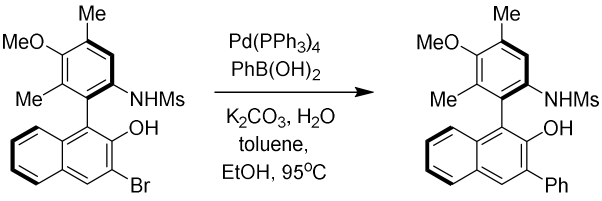

(*R*)-**1b** **5a**

The mixture of (*R*)-**1b** (45 mg, 0.1 mmol), phenyl boronic acid (15 mg, 0.2 mmol), $Pd(PPh_3)_4$ (0.02 mmol.), K_3PO_4 (5.5 mg, 0.4 mmol) in toluene (0.6 mL), EtOH (0.2 mL) and H_2O (0.3 mL) was heated at 95 °C for 12 h under N_2 atmosphere. After cooling to room temperature, the reaction was quenched by adding H_2O. The crude mixture was extracted with EtOAc (3 × 20 mL) and the combined organic extracts were washed with brine, dried over Na_2SO_4, and concentrated *in vacuo*. The residue was purified by column chromatography (silica gel, hexanes/EtOAc = 100:1 v/v) to give the desired product **5a** as a white solid (36 mg, 82% yield).

MP: 206 – 207 °C. **^{1}H NMR** (500 MHz, $CDCl_3$): δ 7.94 (s, 1H), 7.93 – 7.90 (m, 1H), 7.68 – 7.64 (m, 2H), 7.57 – 7.53 (m, 3H), 7.50 – 7.46 (m, 1H), 7.45 – 7.39 (m, 2H), 7.18 – 7.14 (m, 1H), 5.90 (s, 1H), 5.34 (s, 1H), 3.80 (s, 3H), 2.77 (s, 3H), 2.46 (s, 3H), 1.96 (s, 3H). **^{13}C NMR** (125 MHz, $CDCl_3$): δ 154.98 , 148.32 , 136.75 , 132.94 , 132.67 , 132.07 , 131.81 , 131.07 , 130.54 , 129.41 , 129.38 , 128.98 , 128.80 , 128.23 , 127.65 , 124.60 , 124.36 , 123.18 , 121.37 , 114.95 , 60.14 , 39.52 , 16.58 , 13.42 . **HRMS (ESI)** m/z Calcd for $[C_{26}H_{25}NNaO_4S, M + Na]^+$: 470.1396; Found: 470.1392.

Optical Rotation: $[\alpha]^{25}_{D}$ + 8.0 (*c* = 0.3, $CHCl_3$). 98% ee (HPLC condition: Chiralpak AD-H column, *n*-Hexane/*i*-PrOH = 92:8, flow rate = 1.0 mL/min, wavelength = 254 nm, t_R = 15.13 min for major isomer, t_R = 17.36 min for minor isomer).

Supplementary Figure 39. HPLC Trace of **5a**.

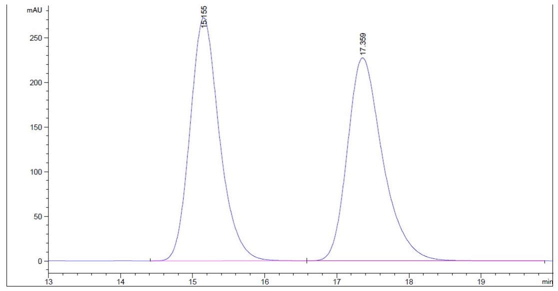

Peak #	RetTime [min]	Type	Width [min]	Area [mAU*s]	Height [mAU]	Area %
1	15.155	BB	0.4323	7727.96484	272.13968	49.9995
2	17.359	BB	0.5154	7728.11523	227.44086	50.0005

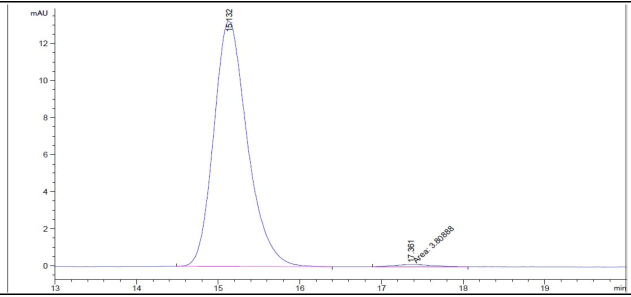

Peak #	RetTime [min]	Type	Width [min]	Area [mAU*s]	Height [mAU]	Area %
1	15.132	BB	0.4330	377.79932	13.23781	99.0019
2	17.361	MM	0.5330	3.80888	1.19092e-1	0.9981

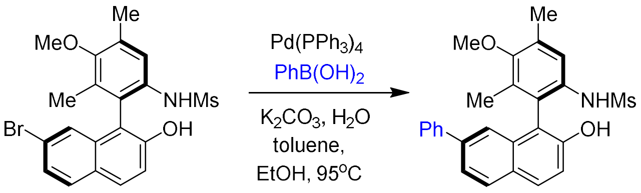

(*R*)-**1f:** 97% ee   **5b:** 82%, 97% ee

The same procedure was followed to yield **5b** as an oil. **^{1}H NMR** (500 MHz, $CDCl_3$): δ 7.97 (d, *J* = 8.5 Hz, 1H), 7.94 (d, *J* = 9.0 Hz, 1H), 7.66 (dd, *J* = 8.5, 1.8 Hz, 1H), 7.55 – 7.50 (m, 3H), 7.44 (dd, *J* = 8.4, 6.8 Hz, 2H), 7.37 (t, *J* = 7.3 Hz, 1H), 7.31 (d, *J* = 8.8 Hz, 1H), 7.28 (d, *J* = 6.6 Hz, 1H), 5.85 (s, 1H), 5.15 (s, 1H), 3.81 (s, 3H), 2.75 (s, 3H), 2.46 (s, 3H), 1.96 (s, 3H). **^{13}C NMR** (125 MHz, $CDCl_3$): δ 155.12 , 151.35 , 140.89 , 140.66 , 133.46 , 132.98 , 132.69 , 132.10 , 130.94 , 129.32 , 128.83 , 128.58 , 127.60 , 127.45 , 124.13 , 123.31 , 121.73 , 120.95 , 117.72 , 114.36 , 60.17 , 39.70 , 16.62 , 13.40 . **HRMS (ESI)** m/z Calcd for $[C_{26}H_{25}NNaO_4S, M + Na]^+$: 470.1396; Found: 470.1398.

Optical Rotation: $[\alpha]^{25}_D$ + 60.0 (*c* = 0.4, $CHCl_3$). 97% ee (HPLC condition: Chiralpak AD-H column, *n*-Hexane/*i*-PrOH = 90:10, flow rate = 1.0 mL/min, wavelength = 254 nm, t_R = 10.05 min for major isomer, t_R = 19.54 min for minor isomer).

Supplementary Figure 40. HPLC Trace of **5b**.

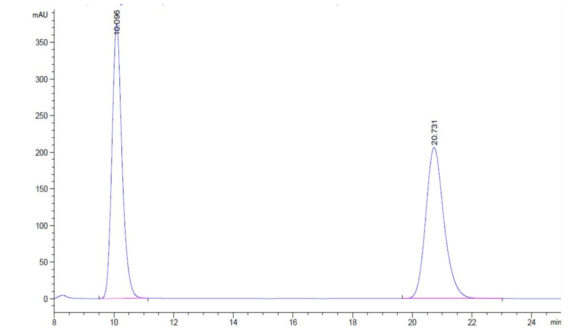

Peak #	RetTime [min]	Type	Width [min]	Area [mAU*s]	Height [mAU]	Area %
1	10.096	BB	0.3561	8777.12109	375.00723	50.1677
2	20.731	BB	0.6455	8718.42578	206.12692	49.8323

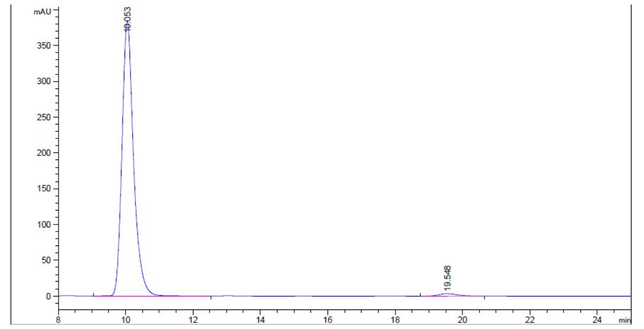

Peak #	RetTime [min]	Type	Width [min]	Area [mAU*s]	Height [mAU]	Area %
1	10.053	BB	0.3597	9187.23242	384.64966	98.6055
2	19.548	BB	0.5613	129.92493	3.35892	1.3945

Access to free amino phenols and derivatization:

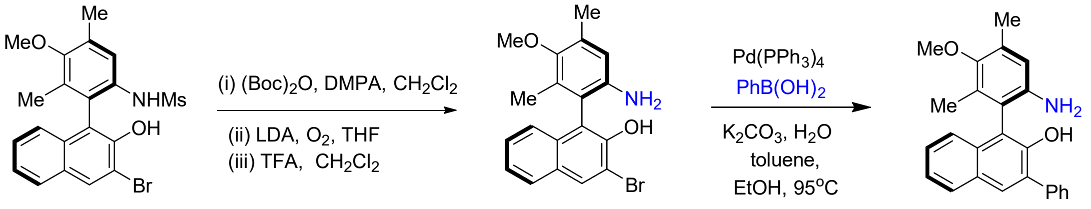

(*R*)-**1b:** 99% ee   **6a:** 82%, 99% ee   **6b:** 65%, 99% ee

The synthesis of **6a** followed our previous procedure[2] and the cross coupling step used the procedure shown in 5.2.

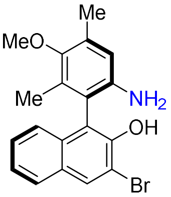

6a: Brown solid. **MP:** 176 - 177 °C. **^{1}H NMR** (400 MHz, DMSO-d_6): δ 8.33 (s, 1H), 7.92 – 7.86 (m, 1H), 7.44 – 7.33 (m, 2H), 7.03 – 6.95 (m, 1H), 6.87 (s, 1H), 3.67 (s, 3H), 2.31 (s, 3H), 1.70 (s, 3H). **^{13}C NMR** (125 MHz, DMSO-d_6): δ 152.84, 149.32, 134.50, 132.62, 132.55, 131.80, 131.42, 129.68, 127.80, 127.54, 124.51, 124.08, 123.60, 119.46, 118.28, 114.55, 60.08, 16.46, 13.55. **HRMS (ESI)** m/z Calcd for [$C_{19}H_{18}BrNNaO_2$, M + Na]$^+$: 394.0413; Found: 394.0416.

Optical Rotation: $[\alpha]^{25}_{D}$ + 9.0 (c = 0.4, $CHCl_3$). 99% ee (HPLC condition: Chiralpak OD-H column, n-Hexane/i-PrOH = 90:10, flow rate = 1.0 mL/min, wavelength = 254 nm, t_R = 8.57 min for minor isomer, t_R = 16.93 min for major isomer).

Supplementary Figure 41. HPLC Trace of **6a**.

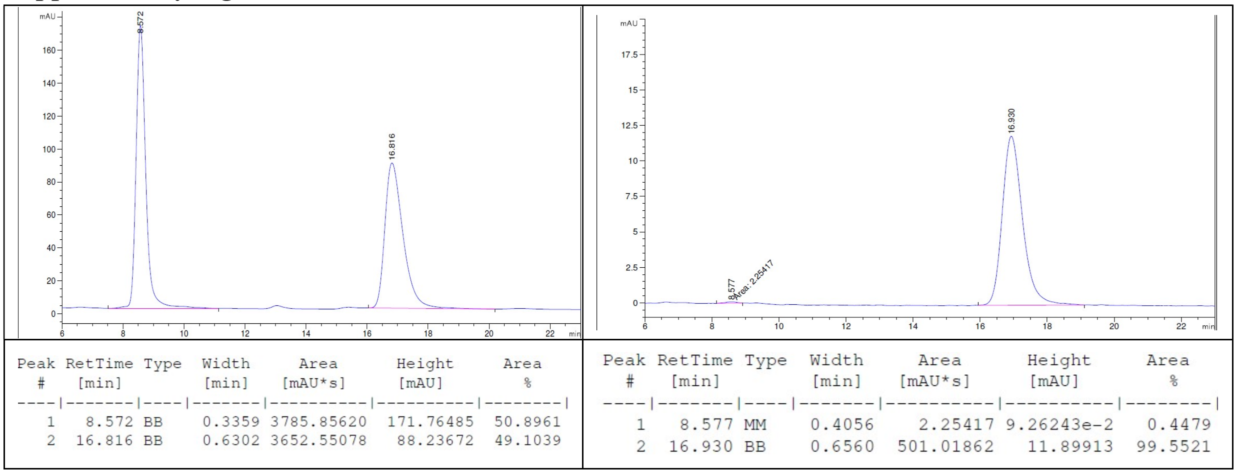

Peak #	RetTime [min]	Type	Width [min]	Area [mAU*s]	Height [mAU]	Area %
1	8.572	BB	0.3359	3785.85620	171.76485	50.8961
2	16.816	BB	0.6302	3652.55078	88.23672	49.1039

Peak #	RetTime [min]	Type	Width [min]	Area [mAU*s]	Height [mAU]	Area %
1	8.577	MM	0.4056	2.25417	9.26243e-2	0.4479
2	16.930	BB	0.6560	501.01862	11.89913	99.5521

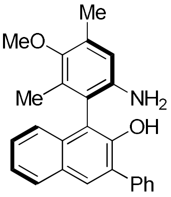

6b: Syrup. **^{1}H NMR** (500 MHz, $CDCl_3$): δ 7.92 (s, 1H), 7.89 (dd, J = 6.2, 3.3 Hz, 1H), 7.77 – 7.73 (m, 2H), 7.50 (t, J = 7.7 Hz, 2H), 7.45 – 7.37 (m, 3H), 7.33 (dt, J = 6.1, 3.5 Hz, 1H), 6.63 (s, 1H), 3.75 (s, 3H), 2.37 (s, 3H), 1.93 (s, 3H). **^{13}C NMR** (125 MHz, $CDCl_3$): δ 150.13 , 148.64 , 141.64 , 137.97 , 132.55 , 132.20 , 132.11 , 130.49 , 130.09 , 129.58 , 129.38 , 128.34 , 128.27 , 127.48 , 126.87 , 123.94 , 123.90 , 116.76 , 116.42 , 115.66 , 60.26 , 16.34 , 13.15 . **HRMS (ESI)** m/z Calcd for [$C_{25}H_{23}NNaO_2$, M + Na]$^+$: 392.1621; Found: 392.1624.

Optical Rotation: $[\alpha]^{25}_{D}$ + 40.0 (c = 0.2, $CHCl_3$). 99% ee (HPLC condition: Chiralpak OD-H column, n-Hexane/i-PrOH = 90:10, flow rate = 1.0 mL/min, wavelength = 254 nm, t_R = 8.43 min for major isomer, t_R = 14.04 min for minor isomer).

Supplementary Figure 42. HPLC Trace of **6b**.

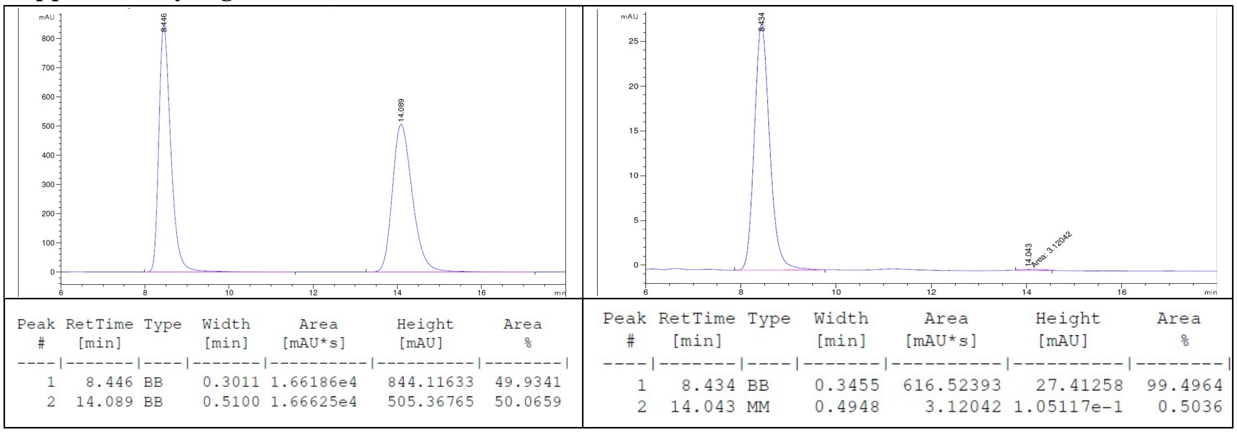

Peak #	RetTime [min]	Type	Width [min]	Area [mAU*s]	Height [mAU]	Area %
1	8.446	BB	0.3011	1.66186e4	844.11633	49.9341
2	14.089	BB	0.5100	1.66625e4	505.36765	50.0659

Peak #	RetTime [min]	Type	Width [min]	Area [mAU*s]	Height [mAU]	Area %
1	8.434	BB	0.3455	616.52393	27.41258	99.4964
2	14.043	MM	0.4948	3.12042	1.05117e-1	0.5036

Access to pyridine-containing amino alcohol 7 and application to ATH:

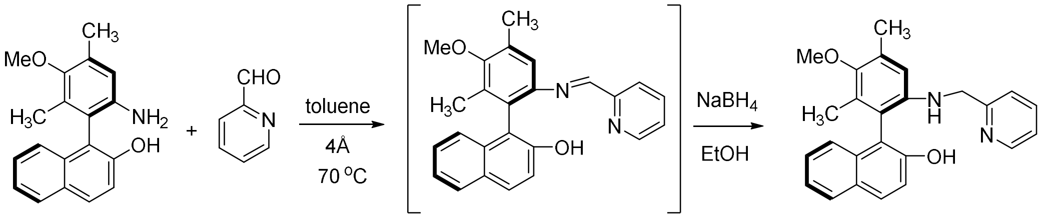

98% ee

7, 50%, 98% ee

The synthesis of (*R*)-1-(6-amino-3-methoxy-2,4-dimethylphenyl)naphthalen-2-ol followed our previous procedure[2]

(*R*)-1-(6-amino-3-methoxy-2,4-dimethylphenyl)naphthalen-2-ol (60 mg, 0.2 mmol) was mixed with Pyridine-2-carboxaldehyde (24 mg, 0.22 mmol) in dry toluene (1mL). 140 mg 4Å molecular sieves were added , and the solution was warmed up to 70 °C and kept for one day at this temperature. The solution was filtered and the solvent was removed under reduced pressure. The resulting light yellow solid was dissolved in EtOH (2. mL) and cooled to 0 °C. $NaBH_4$ (0.8 mmol) was added to the reaction flask. The reaction mixture was then left to stir at 24°C for 4 h. The solvent was removed and the residue was treated with H_2O (5 mL) and extracted with ethyl acetate (10 mL x 3) and washed with brine (10 mL). The combined organic layers were dried with anhydrous Na_2SO_4 and the solvent was removed under reduced pressure, The residue was further purified by flash column chromatography (hexane/ethyl acetate = 2:1) to give **7** (38 mg, 50% yield). ^{1}H NMR (500 MHz, CD_2Cl_2) δ 8.49 (d, *J* = 4.6 Hz, 1H), 7.96 – 7.87 (m, 2H), 7.80 – 7.71 (m, 1H), 7.43 – 7.36 (m, 3H), 7.36 – 7.28 (m, 2H), 7.29 – 7.23 (m, 1H), 6.42 (s, 1H), 4.54 (d, *J* = 17.2 Hz, 1H), 4.38 (d, *J* = 17.2 Hz, 1H), 4.24 – 4.19 (m, 1H), 3.70 (s, 3H), 2.28 (s, 3H), 1.81 (s, 3H). ^{13}C NMR (125 MHz, CD_2Cl_2) δ 158.59 , 151.70 , 149.44 , 142.19 , 133.36 , 131.93 , 131.78 , 130.86 , 129.82 , 129.52 , 128.16 , 126.70 , 124.08 , 123.45 , 122.31 , 121.94 , 118.66 , 116.46 , 111.30 , 59.95 , 48.18 , 16.27 , 12.91 . **HRMS (ESI)** m/z Calcd for [$C_{25}H_{24}N_2NaO_2$, M + Na]$^+$: 407.1730; Found: 407.1734.

Optical Rotation: $[\alpha]^{25}_D$ - 36.0 (*c* = 1.0, CH_2Cl_2). 98% ee (HPLC condition: Chiralcel OD-H column, *n*-Hexane/*i*-PrOH = 90:10, flow rate = 1.0 mL/min, wavelength = 254 nm, t_R = 7.71 min for major isomer, t_R = 11.85 min for minor isomer).

Supplementary Figure 43. HPLC Trace of **7**.

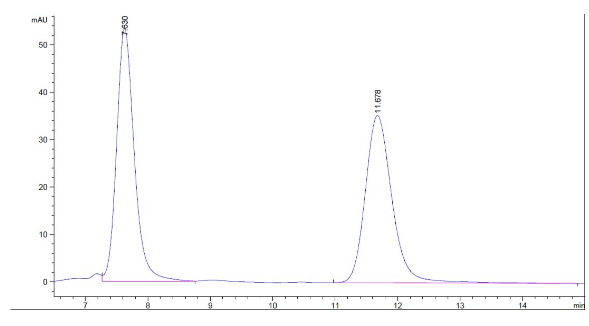

Peak #	RetTime [min]	Type	Width [min]	Area [mAU*s]	Height [mAU]	Area %
1	7.630	VB	0.3004	1049.02820	53.35166	50.0117
2	11.678	BB	0.4543	1048.53625	35.32781	49.9883

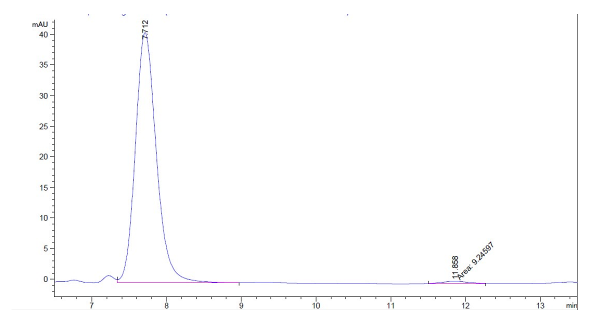

Peak #	RetTime [min]	Type	Width [min]	Area [mAU*s]	Height [mAU]	Area %
1	7.712	VB	0.3000	798.55054	40.85883	98.8554
2	11.858	MM	0.4175	9.24597	3.69097e-1	1.1446

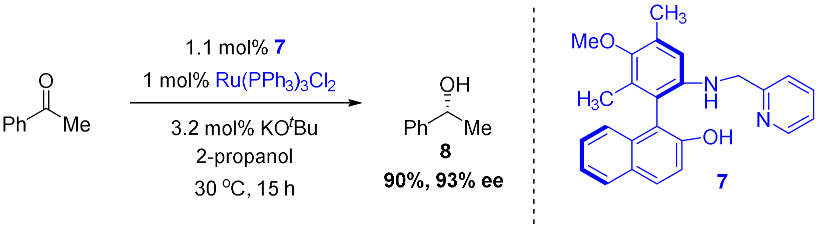

The ligand **7** (4.7 μmol) was dissolved in 2-propanol (5.2 mL) under N_2 and a solution of KO^{t}Bu in 2-propanol (0.95 mL of 0.01 mol/L) was added. $Ru(PPh_3)_3Cl_2$ (4.1 mg 4.3 μmol) dissolved in 2-propanol (2.5 mL), was added. The solution was stirred at room temperature for 1.5 h to accomplish the formation of the catalyst. Then, the reaction vessel was thermostated to 30 ºC. Addition of acetophenone (50 μL) afforded a substrate concentration of 0.05 mol/L. The reaction was started by adding another 0.43 mL of the 0.01 mol/L KO^{t}Bu solution. After 15 h the reaction was stopped by addition of 0.1 mol/L solution of acetic acid in 2-propanol (0.15 mL). After removal the solvent, the crude residue was purified by column chromatography with hexanes/ethyl acetate (10:1 v/v) as eluent to afford the product **8**. ^{1}H NMR (500 MHz, $CDCl_3$): δ 7.43 – 7.35 (m, 4H), 7.33 – 7.29 (m, 1H), 4.92 (qd, J = 6.4, 3.1 Hz, 1H), 2.04 – 1.96 (m, 1H), 1.53 (d, J = 6.5 Hz, 3H). ^{13}C NMR (125 MHz, $CDCl_3$): δ 145.82 , 128.50 , 127.48 , 125.39 , 70.42 , 25.16 .

Optical Rotation: $[\alpha]^{25}_{D}$ 36.0 (c = 1.0, $CHCl_3$). The absolute configuration of **8** was assigned by comparing its specific rotation with that of the same compound reported in the literature.[7] 93% ee (HPLC condition: Chiralcel OD-H column, n-Hexane/i-PrOH = 98:2, flow rate = 1.0 mL/min, wavelength = 254 nm, t_R = 14.95 min for major isomer, t_R = 19.16 min for minor isomer).

Supplementary Figure 44. HPLC Trace of **8**.

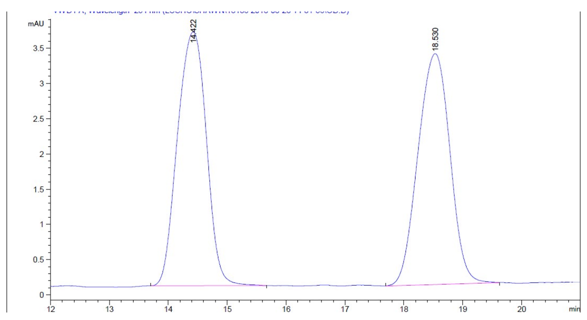

Peak #	RetTime [min]	Type	Width [min]	Area [mAU*s]	Height [mAU]	Area %
1	14.422	BB	0.5512	122.84039	3.59868	49.8765
2	18.530	BB	0.5959	123.44855	3.27370	50.1235

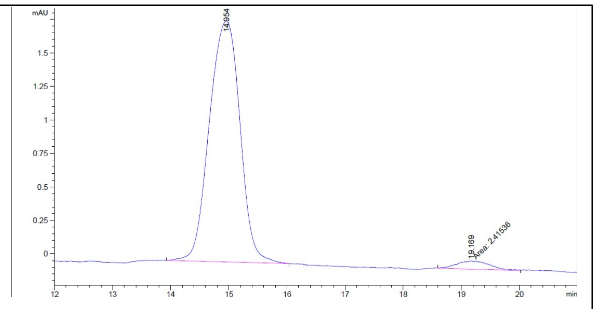

Peak #	RetTime [min]	Type	Width [min]	Area [mAU*s]	Height [mAU]	Area %
1	14.954	BB	0.5756	65.98298	1.80010	96.4687
2	19.169	MM	0.6535	2.41536	6.16019e-2	3.5313

Supplementary Table 1. Optimization of the Reaction Conditions[a]

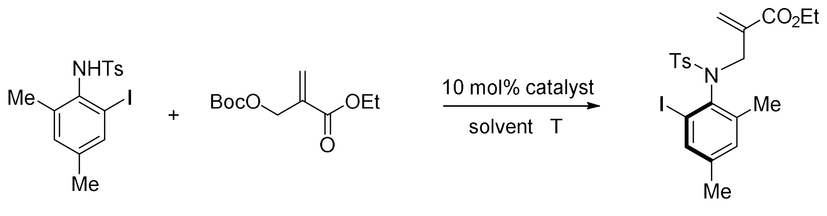

9b 2a 10ba

entry	cat	solvent	T,(°C)	**10ba**, yield(%)[b], ee (%)[c]
1	*β*-ICD	CH_2Cl_2	24	99, -41
2	$(DHQD)_2$AQN	CH_2Cl_2	24	90, -30
3	$(DHQD)_2$Pyr	CH_2Cl_2	24	86, -50
4	$(DHQD)_2$PHAL	CH_2Cl_2	24	92, -67
5	$(DHQ)_2$AQN	CH_2Cl_2	24	91, 41
6	$(DHQ)_2$Pyr	CH_2Cl_2	24	86, 72
7	$(DHQ)_2$PHAL	CH_2Cl_2	24	96, 78
8	$(DHQ)_2$PHAL	THF	24	95, 72
9	$(DHQ)_2$PHAL	CH_3CN	24	95, 80
10	$(DHQ)_2$PHAL	EtOAc	24	92, 73
11	$(DHQ)_2$PHAL	$CHCl_3$	24	91, 74
12	$(DHQ)_2$PHAL	toluene	24	81, 51
13[d]	$(DHQ)_2$PHAL	CH_3CN	-20	90, 87
14[d]	**$(DHQ)_2$PHAL**	**1:1 CH_2Cl_2/CH_3CN**	**-20**	**85, 92**
15[d]	$(DHQD)_2$PHAL	1:1 CH_2Cl_2/CH_3CN	-20	86, -87

[a] Unless noted otherwise, the reactions were performed with **9b** (0.04 mmol, 1.0 equiv.), **2a** (1.5 equiv.), catalyst (10 mol%) in solvent (0.5 mL) at 24 °C for 18 h. [b] isolated yield. [c] Determined by chiral HPLC. [d] 96 h instead of 18 h.

Representative procedure for synthesis of 10:

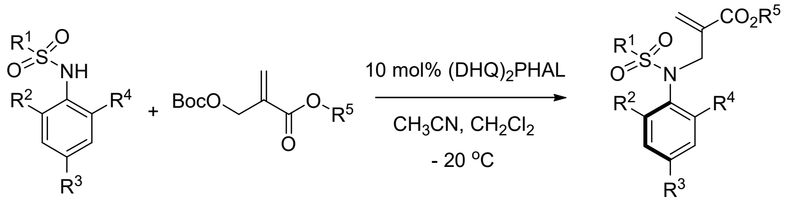

9 2 10

To a 4 mL vial containing **9** (0.04 mmol) and $(DHQ)_2$PHAL (3.0 mg, 10 mol%) were added CH_2Cl_2 (0.25 mL) and CH_3CN (0.25 mL). The reaction mixture was allowed to stir for 10 minutes at -20 °C. Then MBH carbonate (12 μL) was added. Once the starting material **9** consumed completely. The volatiles were removed *in vacuo* at 24 °C and the residue was purified by silica gel column chromatography with hexanes/ethyl acetate (10:1 v/v) as the eluent to afford the product **10**.

Characterization of compounds:

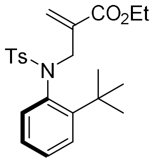

9aa. Syrup. **^{1}H NMR** (400 MHz, $CDCl_3$) δ 7.55 (d, *J* = 8.3 Hz, 2H), 7.48 (dd, *J* = 8.2, 1.6 Hz, 1H), 7.27 – 7.21 (m, 2H), 7.16 (td, *J* = 8.0, 1.5 Hz, 1H), 6.88 (td, *J* = 8.0, 7.1, 1H), 6.39 (dd, *J* = 8.0, 1.5 Hz, 1H), 6.14 (d, *J* = 1.2 Hz, 1H), 5.45 (d, *J* = 1.1 Hz, 1H), 4.63 (dd, *J* = 13.6, 0.8 Hz, 1H), 4.03 (dd, *J* = 13.6, 0.7 Hz, 1H), 3.96 – 3.84 (m, 2H), 2.38 (s, 3H), 1.41 (s, 9H), 1.04 (t, *J* = 7.1 Hz, 3H). **^{13}C NMR** (100 MHz, $CDCl_3$) δ 166.08 , 150.73 , 143.61 , 136.64 , 135.55 , 135.17 , 131.58 , 130.80 , 129.74 , 129.33 , 128.94 , 128.32 , 125.66 , 61.01 , 52.29 , 36.91 , 33.01 , 21.58 , 13.94 . **HRMS (ESI)** m/z Calcd for $[C_{23}H_{29}NNaO_4S, M + Na]^+$: 438.1709; Found: 438.1706.

Optical Rotation: $[\alpha]^{25}_D$ - 20.0 (*c* = 1.0, $CHCl_3$). 71% ee (HPLC condition: Chiralpak AD-H column, *n*-Hexane/*i*-PrOH = 90:10, flow rate = 1.0 mL/min, wavelength = 254 nm, t_R = 7.35 min for minor isomer, t_R = 9.10 min for major isomer).

Supplementary Figure 45. HPLC Trace of **9aa**.

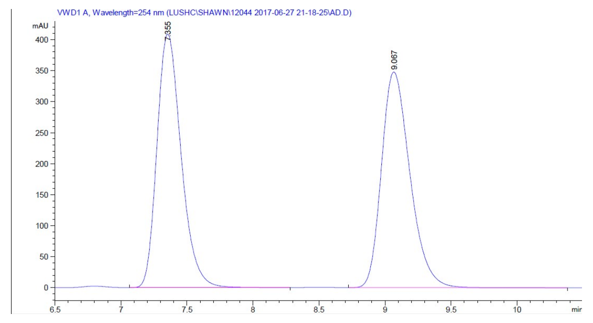

Peak #	RetTime [min]	Type	Width [min]	Area [mAU*s]	Height [mAU]	Area %
1	7.355	VB	0.1977	5209.39307	408.79929	49.9687
2	9.067	BB	0.2320	5215.92676	347.66235	50.0313

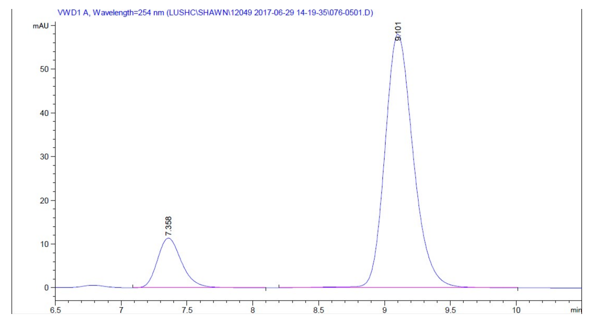

Peak #	RetTime [min]	Type	Width [min]	Area [mAU*s]	Height [mAU]	Area %
1	7.358	BB	0.1967	144.83440	11.36150	14.3210
2	9.101	BB	0.2293	866.50732	58.00057	85.6790

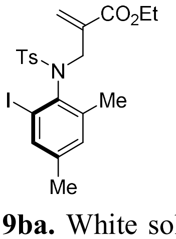

9ba. White solid. **MP:** 122-124 °C. **^{1}H NMR** (500 MHz, CD_2Cl_2) δ 7.82 – 7.76 (m, 2H), 7.59 (d, *J* = 1.9 Hz, 1H), 7.40 – 7.34 (m, 2H), 7.06 (d, *J* = 1.2 Hz, 1H), 6.31 (s, 1H), 5.82 (s, 1H), 4.61 (d, *J* = 14.3 Hz, 1H), 4.57 (d, *J* = 14.1 Hz, 1H), 4.13 – 3.95 (m, 2H), 2.49 (s, 3H), 2.30 (s, 3H), 2.18 (s, 3H), 1.17 (t, *J* = 7.1 Hz, 3H). **^{13}C NMR** (125 MHz, CD_2Cl_2) δ 166.01 , 143.76 , 141.94 , 140.00 , 139.06 , 138.33 , 137.35 , 135.98 , 132.22 , 131.49 , 129.52 , 128.23 , 100.97 , 60.97 , 49.97 , 21.43 , 20.18 , 13.78 . **HRMS (ESI)** m/z Calcd for $[C_{21}H_{24}INNaO_4S, M + Na]^+$: 536.0363; Found: 536.0367.

Optical Rotation: $[\alpha]^{25}_D$ - 30.0 (*c* = 1.0, $CHCl_3$). 92% ee (HPLC condition: Chiralpak AD-H

column, n-Hexane/i-PrOH = 90:10, flow rate = 1.0 mL/min, wavelength = 254 nm, t_R = 10.96 min for minor isomer, t_R = 11.74 min for major isomer).

Supplementary Figure 46. HPLC Trace of **9ba**.

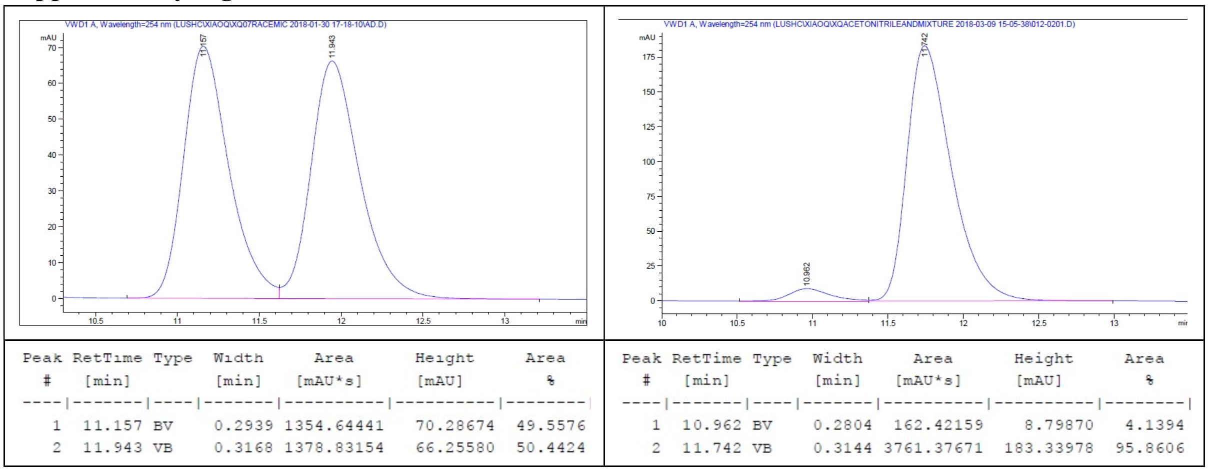

Peak #	RetTime [min]	Type	Width [min]	Area [mAU*s]	Height [mAU]	Area %
1	11.157	BV	0.2939	1354.64441	70.28674	49.5576
2	11.943	VB	0.3168	1378.83154	66.25580	50.4424

Peak #	RetTime [min]	Type	Width [min]	Area [mAU*s]	Height [mAU]	Area %
1	10.962	BV	0.2804	162.42159	8.79870	4.1394
2	11.742	VB	0.3144	3761.37671	183.33978	95.8606

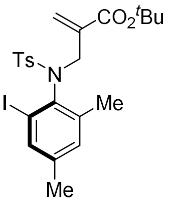

9bb. Syrup. **^{1}H NMR** (400 MHz, $CDCl_3$) δ 7.66 (d, J = 8.3 Hz, 2H), 7.42 (dt, J = 2.1, 0.7 Hz, 1H), 7.24 – 7.18 (m, 2H), 6.94 – 6.90 (m, 1H), 6.18 (d, J = 1.4 Hz, 1H), 5.73 (d, J = 1.4 Hz, 1H), 4.50 (dd, J = 14.2, 0.6 Hz, 1H), 4.40 (d, J = 14.3 Hz, 1H), 2.35 (s, 3H), 2.16 (s, 3H), 2.15 (s, 3H), 1.24 (s, 9H). **^{13}C NMR** (100 MHz, $CDCl_3$) δ 165.18 , 143.41 , 142.44 , 139.83 , 139.07 , 138.57 , 137.30 , 132.29 , 131.27 , 129.46 , 128.32 , 100.37 , 81.01 , 49.72 , 27.74 , 21.59 , 20.60 , 20.33 . **HRMS (ESI)** m/z Calcd for $[C_{23}H_{28}INNaO_4S, M + Na]^+$: 564.0676; Found: 564.0679.

Optical Rotation: $[\alpha]^{25}_D$ - 30.0 (c = 1.0, $CHCl_3$). 92% ee (HPLC condition: Chiralpak AD-H column, n-Hexane/i-PrOH = 90:10, flow rate = 1.0 mL/min, wavelength = 254 nm, t_R = 8.12 min for major isomer, t_R = 9.50 min for minor isomer).

Supplementary Figure 47. HPLC Trace of **9bb**.

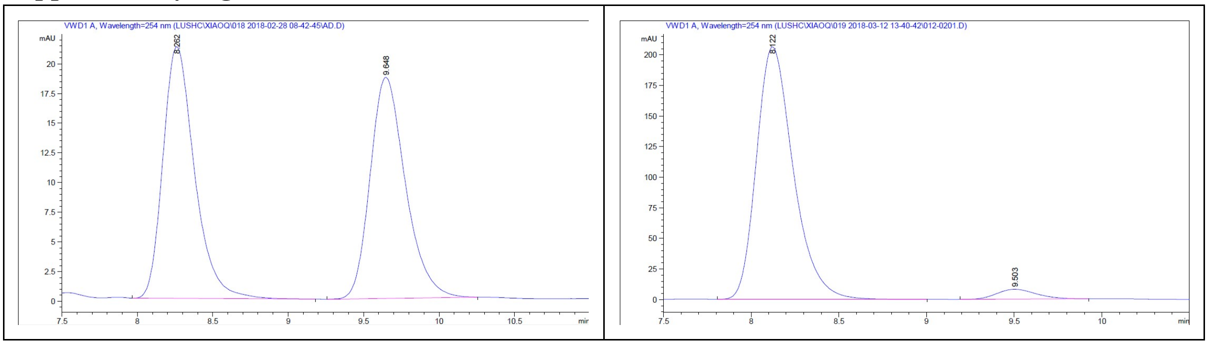

Peak #	RetTime [min]	Type	Width [min]	Area [mAU*s]	Height [mAU]	Area %
1	8.262	BB	0.2232	309.25885	21.20106	50.4792
2	9.648	BB	0.2500	303.38675	18.62873	49.5208

Peak #	RetTime [min]	Type	Width [min]	Area [mAU*s]	Height [mAU]	Area %
1	8.122	VB	0.2235	3015.02222	206.32355	95.9936
2	9.503	BB	0.2434	125.83624	8.04706	4.0064

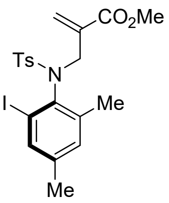

9bc. Syrup. **^{1}H NMR** (500 MHz, $CDCl_3$) δ 7.78 (d, J = 8.3 Hz, 2H), 7.53 (d, J = 2.0 Hz, 1H), 7.37 – 7.29 (m, 2H), 7.01 (d, J = 2.0 Hz, 1H), 6.30 (d, J = 1.3 Hz, 1H), 5.79 (d, J = 1.2 Hz, 1H), 4.64 – 4.57 (m, 1H), 4.55 (d, J = 14.2 Hz, 1H), 3.59 (s, 3H), 2.45 (s, 3H), 2.25 (s, 3H), 2.20 (s, 3H). **^{13}C NMR** (125 MHz, $CDCl_3$) δ 166.56 , 143.53 , 141.96 , 139.85 , 139.10 , 138.30 , 137.23 , 135.53 , 132.28 , 131.92 , 129.45 , 128.38 , 100.81 , 51.94 , 50.13 , 21.60 , 20.36 , 20.31 . **HRMS (ESI)** m/z Calcd for [$C_{20}H_{22}INNaO_4S$, M + Na]$^+$: 522.0206; Found: 522.0202.

Optical Rotation: $[\alpha]^{25}_D$ - 30.0 (c = 1.0, $CHCl_3$). 93% ee (HPLC condition: Chiralpak AD-H column, n-Hexane/i-PrOH = 95:5, flow rate = 1.0 mL/min, wavelength = 254 nm, t_R = 29.35 min for minor isomer, t_R = 34.78 min for major isomer).

Supplementary Figure 48. HPLC Trace of **9bc**.

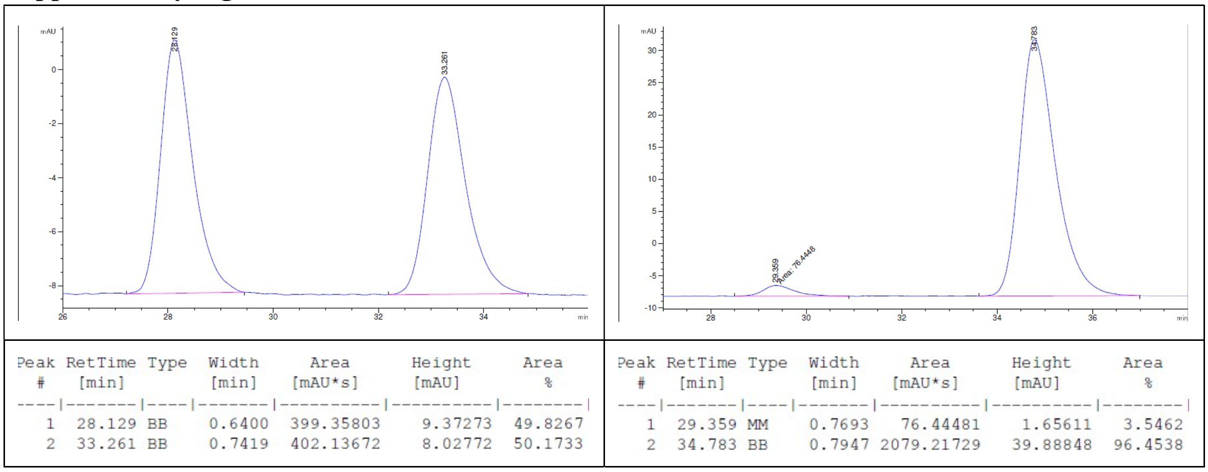

Peak #	RetTime [min]	Type	Width [min]	Area [mAU*s]	Height [mAU]	Area %
1	28.129	BB	0.6400	399.35803	9.37273	49.8267
2	33.261	BB	0.7419	402.13672	8.02772	50.1733

Peak #	RetTime [min]	Type	Width [min]	Area [mAU*s]	Height [mAU]	Area %
1	29.359	MM	0.7693	76.44481	1.65611	3.5462
2	34.783	BB	0.7947	2079.21729	39.88848	96.4538

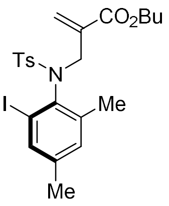

9bd. Syrup. **^{1}H NMR** (500 MHz, $CDCl_3$) δ 7.77 (d, J = 8.4 Hz, 2H), 7.52 (d, J = 2.0 Hz, 1H), 7.31 (d, J = 8.1 Hz, 2H), 7.01 (d, J = 2.0 Hz, 1H), 6.33 (d, J = 1.3 Hz, 1H), 5.87 (d, J = 1.3 Hz, 1H), 4.61 (d, J = 14.2 Hz, 1H), 4.54 (d, J = 14.3 Hz, 1H), 4.02 (dt, J = 10.7, 6.8 Hz, 1H), 3.94 (dt, J = 10.8, 6.6 Hz, 1H), 2.45 (s, 3H), 2.25 (s, 3H), 2.21 (s, 3H), 1.54 – 1.45 (m, 2H), 1.38 – 1.26 (m, 2H), 0.91 (t, J = 7.4 Hz, 3H).

^{13}C NMR (125 MHz, $CDCl_3$) δ 166.20 , 143.49 , 142.08 , 139.81 , 139.10 , 138.39 , 137.35 , 135.97 , 132.26 , 131.70 , 129.47 , 128.34 , 100.67 , 64.88 , 50.04 , 30.38 , 21.60 , 20.42 , 20.36 , 19.11 , 13.71 . **HRMS (ESI)** m/z Calcd for $[C_{23}H_{28}INNaO_4S, M + Na]^+$: 564.0676; Found: 564.0673.

Optical Rotation: $[\alpha]^{25}_D$ - 25.0 (c = 1.0, $CHCl_3$). 93% ee (HPLC condition: Chiralpak OD-H column, n-Hexane/i-PrOH = 99:1, flow rate = 1.0 mL/min, wavelength = 254 nm, t_R = 15.72 min for major isomer, t_R = 17.82 min for minor isomer).

Supplementary Figure 49. HPLC Trace of **9bd**.

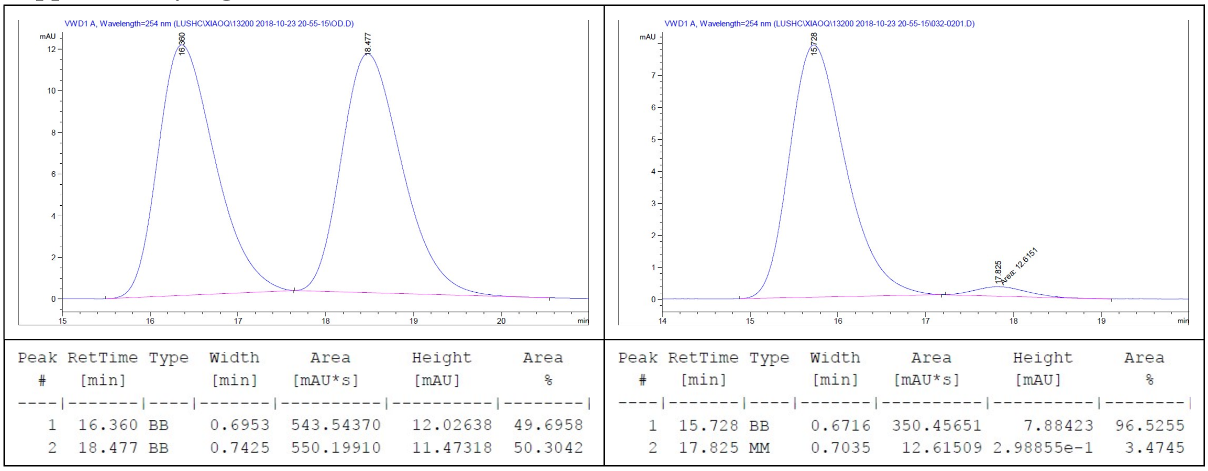

Peak #	RetTime [min]	Type	Width [min]	Area [mAU*s]	Height [mAU]	Area %
1	16.360	BB	0.6953	543.54370	12.02638	49.6958
2	18.477	BB	0.7425	550.19910	11.47318	50.3042

Peak #	RetTime [min]	Type	Width [min]	Area [mAU*s]	Height [mAU]	Area %
1	15.728	BB	0.6716	350.45651	7.88423	96.5255
2	17.825	MM	0.7035	12.61509	2.98855e-1	3.4745

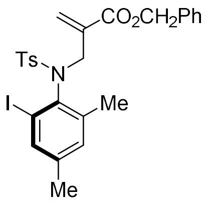

9be. Syrup. **^{1}H NMR** (500 MHz, $CDCl_3$) δ 7.79 – 7.74 (m, 2H), 7.48 (d, J = 2.0 Hz, 1H), 7.40 – 7.34 (m, 3H), 7.31 – 7.26 (m, 4H), 6.96 (d, J = 1.9 Hz, 1H), 6.38 (d, J = 1.2 Hz, 1H), 5.88 (d, J = 1.1 Hz, 1H), 5.10 (d, J = 12.3 Hz, 1H), 5.00 (d, J = 12.4 Hz, 1H), 4.62 (d, J = 14.3 Hz, 1H), 4.57 (d, J = 14.3 Hz, 1H), 2.45 (s, 3H), 2.23 (s, 3H), 2.15 (s, 3H). **^{13}C NMR** (125 MHz, $CDCl_3$) δ 165.92 , 143.49 , 141.93 , 139.80 , 139.10 , 138.31 , 137.32 , 135.73 , 135.58 , 132.27 , 132.20 , 129.46 , 128.42 , 128.38 , 128.24 , 128.19 , 100.80 , 66.72 , 50.13 , 21.61 , 20.40 , 20.36 . **HRMS (ESI)** m/z Calcd for $[C_{26}H_{26}INNaO_4S, M + Na]^+$: 598.0519; Found: 598.0514.

Optical Rotation: $[\alpha]^{25}_D$ - 35.0 (c = 1.0, $CHCl_3$). 92% ee (HPLC condition: Chiralpak AD-H column, n-Hexane/i-PrOH = 95:5, flow rate = 1.0 mL/min, wavelength = 254 nm, t_R = 28.42 min for minor isomer, t_R = 31.29 min for major isomer).

Supplementary Figure 50. HPLC Trace of **9be**.

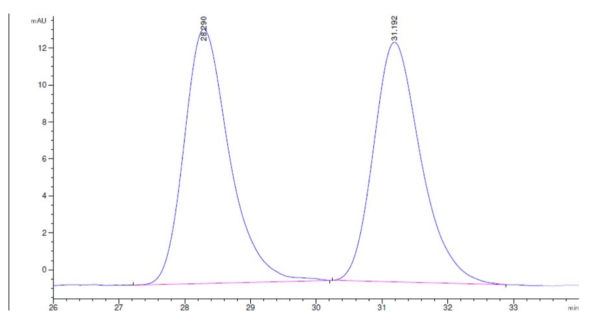

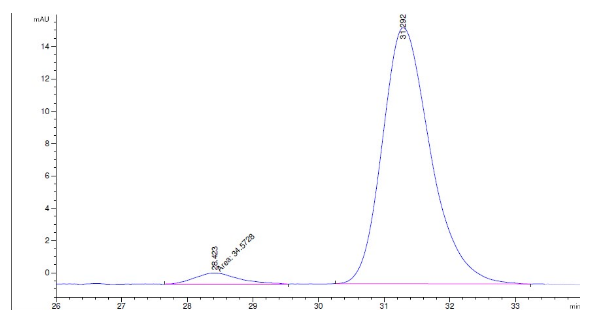

Peak #	RetTime [min]	Type	Width [min]	Area [mAU*s]	Height [mAU]	Area %
1	28.290	BB	0.7197	659.80054	13.76255	50.3478
2	31.192	BB	0.7442	650.68579	12.96951	49.6522

Peak #	RetTime [min]	Type	Width [min]	Area [mAU*s]	Height [mAU]	Area %
1	28.423	MM	0.8115	34.57281	7.10093e-1	4.1008
2	31.292	BB	0.7734	808.50885	15.83960	95.8992

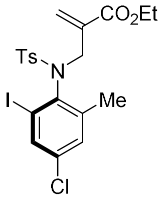

9ca. White solid. **MP:** 132-134 ºC. **^{1}H NMR** (500 MHz, $CDCl_3$) δ 7.77 (d, J = 8.3 Hz, 2H), 7.69 (d, J = 2.5 Hz, 1H), 7.35 – 7.31 (m, 2H), 7.24 – 7.18 (m, 1H), 6.35 (d, J = 1.2 Hz, 1H), 5.88 (d, J = 1.2 Hz, 1H), 4.60 (d, J = 14.2 Hz, 1H), 4.53 (d, J = 14.3 Hz, 1H), 4.09 (dq, J = 10.8, 7.1 Hz, 1H), 4.02 (dq, J = 10.8, 7.1 Hz, 1H), 2.46 (s, 3H), 2.24 (s, 3H), 1.17 (t, J = 7.1 Hz, 3H). **^{13}C NMR** (125 MHz, $CDCl_3$) δ 166.00 , 143.88 , 143.85 , 138.99 , 138.10 , 137.77 , 135.72 , 134.36 , 132.00 , 131.23 , 129.61 , 128.31 , 100.98 , 61.10 , 50.02 , 21.62 , 20.55 , 13.94 . **HRMS (ESI)** m/z Calcd for $[C_{20}H_{21}ClINNaO_4S, M + Na]^+$: 555.9816; Found: 555.9818.

Optical Rotation: $[\alpha]^{25}_D$ - 40.0 (c = 1.0, $CHCl_3$). 91% ee (HPLC condition: Chiralpak OD-H column, n-Hexane/i-PrOH = 90:10, flow rate = 1.0 mL/min, wavelength = 254 nm, t_R = 7.48 min for major isomer, t_R = 8.62 min for minor isomer).

Supplementary Figure 51. HPLC Trace of **9ca**.

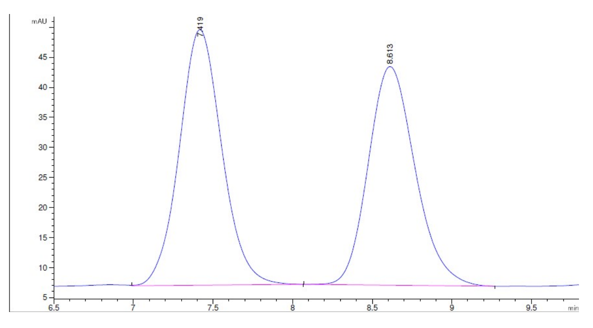

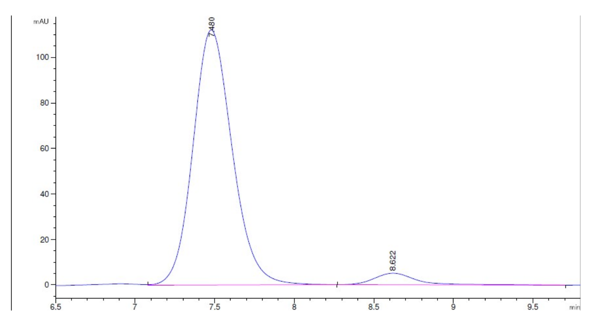

Peak #	RetTime [min]	Type	Width [min]	Area [mAU*s]	Height [mAU]	Area %
1	7.419	VB	0.2853	790.64771	42.54605	51.4537
2	8.613	BB	0.3177	745.97339	36.40962	48.5463

Peak #	RetTime [min]	Type	Width [min]	Area [mAU*s]	Height [mAU]	Area %
1	7.480	VB	0.2644	1926.21643	112.20238	95.3309
2	8.622	BB	0.2833	94.34232	5.05534	4.6691

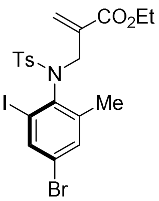

9da. White solid. **MP:** 128-129 ºC. **^{1}H NMR** (500 MHz, $CDCl_3$) δ 7.84 (d, *J* = 2.3 Hz, 1H), 7.76 (d, *J* = 8.2 Hz, 2H), 7.37 (d, *J* = 2.3 Hz, 1H), 7.33 (d, *J* = 8.1 Hz, 2H), 6.35 (d, *J* = 1.1 Hz, 1H), 5.88 (d, *J* = 1.1 Hz, 1H), 4.59 (d, *J* = 14.3 Hz, 1H), 4.52 (d, *J* = 14.3 Hz, 1H), 4.14 – 3.97 (m, 2H), 2.46 (s, 3H), 2.24 (s, 3H), 1.22 – 1.10 (m, 3H). **^{13}C NMR** (125 MHz, $CDCl_3$) δ 165.99 , 144.33 , 143.86 , 140.47 , 139.49 , 138.08 , 135.70 , 134.20 , 132.01 , 129.61 , 128.31 , 122.58 , 101.41 , 61.11 , 49.97 , 21.62 , 20.46 , 13.94 . **HRMS (ESI)** m/z Calcd for $[C_{20}H_{21}BrINNaO_4S, M + Na]^+$: 555.9816; Found: 555.9818.

Optical Rotation: $[\alpha]^{25}_{D}$ - 25.0 (*c* = 0.5, $CHCl_3$). 91% ee (HPLC condition: Chiralpak OD-H column, *n*-Hexane/*i*-PrOH = 95:5, flow rate = 1.0 mL/min, wavelength = 254 nm, t_R = 10.13 min for major isomer, t_R = 12.07 min for minor isomer).

Supplementary Figure 52. HPLC Trace of **9da**.

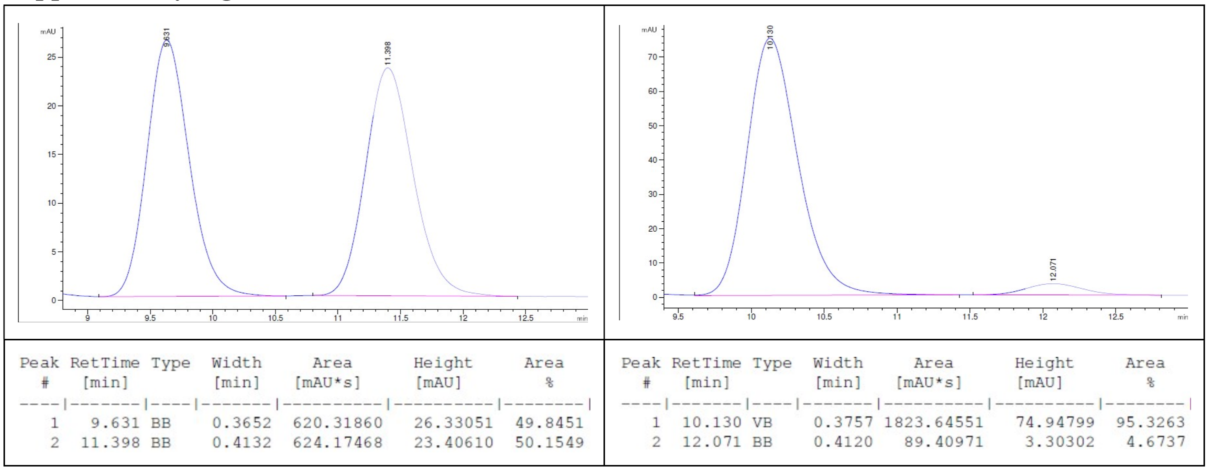

Peak #	RetTime [min]	Type	Width [min]	Area [mAU*s]	Height [mAU]	Area %
1	9.631	BB	0.3652	620.31860	26.33051	49.8451
2	11.398	BB	0.4132	624.17468	23.40610	50.1549

Peak #	RetTime [min]	Type	Width [min]	Area [mAU*s]	Height [mAU]	Area %
1	10.130	VB	0.3757	1823.64551	74.94799	95.3263
2	12.071	BB	0.4120	89.40971	3.30302	4.6737

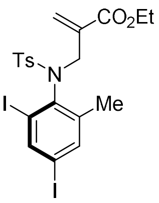

9ea. Syrup. **^{1}H NMR** (500 MHz, $CDCl_3$) δ 8.03 (d, *J* = 2.0 Hz, 1H), 7.79 – 7.72 (m, 2H), 7.55 (d, *J* = 2.0 Hz, 1H), 7.33 (d, *J* = 8.1 Hz, 2H), 6.35 (d, *J* = 1.2 Hz, 1H), 5.87 (d, *J* = 1.1 Hz, 1H), 4.59 (d, *J* = 14.2 Hz, 1H), 4.52 (d, *J* = 14.3 Hz, 1H), 4.14 – 3.97 (m, 2H), 2.46 (s, 3H), 2.21 (s, 3H), 1.16 (t, *J* = 7.1 Hz, 3H). **^{13}C NMR** (125 MHz, $CDCl_3$) δ 165.99 , 146.09 , 144.66 , 143.85 , 140.25 , 138.08 , 135.70 , 132.00 , 129.61 , 128.31 , 101.88 , 94.83 , 61.11 , 49.95 , 21.63 , 20.22 , 13.94 . **HRMS (ESI)** m/z Calcd for $[C_{20}H_{21}I_2NNaO_4S, M + Na]^+$: 647.9173; Found: 647.9177.

Optical Rotation: $[\alpha]^{25}_{D}$ - 15.0 (*c* = 1.0, $CHCl_3$). 89% ee (HPLC condition: Chiralpak AD-H

column, n-Hexane/i-PrOH = 98:2, flow rate = 1.0 mL/min, wavelength = 254 nm, t_R = 33.30 min for major isomer, t_R = 37.02 min for minor isomer).

Supplementary Figure 53. HPLC Trace of **9ea**.

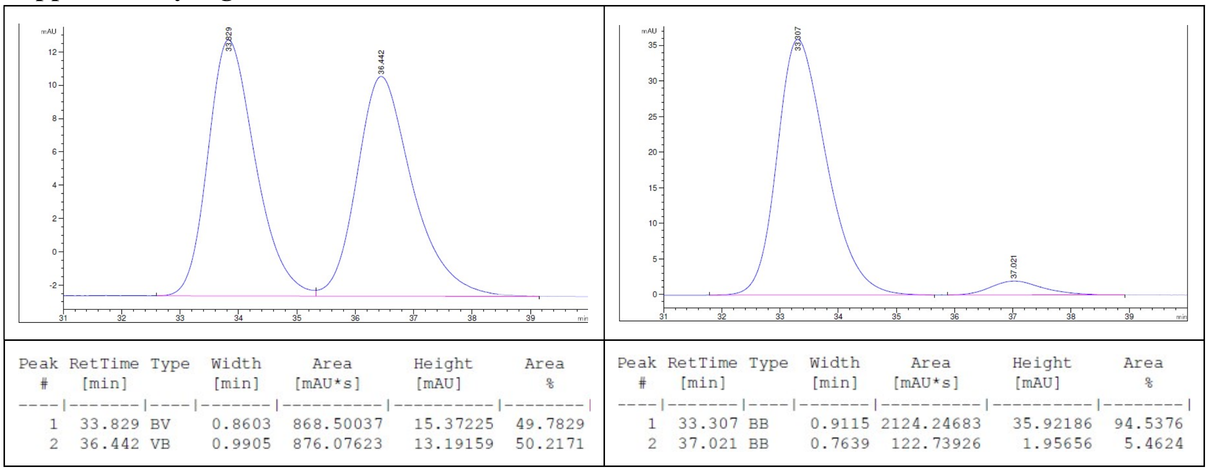

Peak #	RetTime [min]	Type	Width [min]	Area [mAU*s]	Height [mAU]	Area %
1	33.829	BV	0.8603	868.50037	15.37225	49.7829
2	36.442	VB	0.9905	876.07623	13.19159	50.2171

Peak #	RetTime [min]	Type	Width [min]	Area [mAU*s]	Height [mAU]	Area %
1	33.307	BB	0.9115	2124.24683	35.92186	94.5376
2	37.021	BB	0.7639	122.73926	1.95656	5.4624

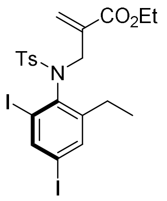

9fa. Syrup. **^{1}H NMR** (500 MHz, $CDCl_3$) δ 8.03 (d, J = 2.1 Hz, 1H), 7.76 (d, J = 8.3 Hz, 2H), 7.61 (d, J = 2.1 Hz, 1H), 7.33 (d, J = 8.1 Hz, 2H), 6.35 (d, J = 1.1 Hz, 1H), 5.85 (d, J = 1.1 Hz, 1H), 4.57 (d, J = 14.3 Hz, 1H), 4.52 (d, J = 14.3 Hz, 1H), 4.14 – 3.96 (m, 2H), 2.65 (dq, J = 15.1, 7.5 Hz, 1H), 2.53 – 2.47 (m, 1H), 2.46 (s, 3H), 1.16 (t, J = 7.5 Hz, 6H). **^{13}C NMR** (125 MHz, $CDCl_3$) δ 165.90 , 150.25 , 145.91 , 143.81 , 139.47 , 138.34 , 138.05 , 135.53 , 132.13 , 129.56 , 128.37 , 101.81 , 95.28 , 61.09 , 49.98 , 25.19 , 21.62 , 14.27 , 13.94 . **HRMS (ESI)** m/z Calcd for $[C_{21}H_{23}I_2NNaO_4S, M + Na]^+$: 661.9329; Found: 661.9324.

Optical Rotation: $[\alpha]^{25}_D$ + 20.0 (c = 1.0, $CHCl_3$). 95% ee (HPLC condition: Chiralpak AD-H column, n-Hexane/i-PrOH = 98:2, flow rate = 1.0 mL/min, wavelength = 254 nm, t_R = 23.34 min for major isomer, t_R = 27.21for minor isomer).

Supplementary Figure 54. HPLC Trace of **9fa**.

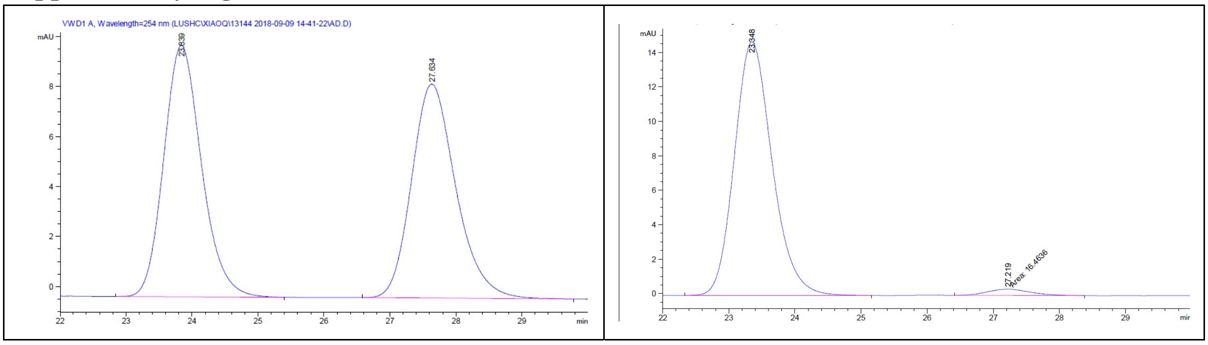

Peak #	RetTime [min]	Type	Width [min]	Area [mAU*s]	Height [mAU]	Area %
1	23.839	BB	0.6063	400.01193	10.05135	50.0716
2	27.634	BB	0.7050	398.86850	8.55420	49.9284

Peak #	RetTime [min]	Type	Width [min]	Area [mAU*s]	Height [mAU]	Area %
1	23.348	BB	0.6071	583.21448	14.72318	97.2546
2	27.219	MM	0.7520	16.46364	3.64873e-1	2.7454

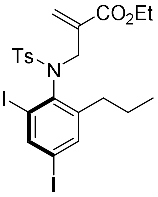

9ga. Syrup. **^{1}H NMR** (500 MHz, $CDCl_3$) δ 8.04 (d, J = 2.0 Hz, 1H), 7.77 (d, J = 8.4 Hz, 2H), 7.60 (d, J = 2.0 Hz, 1H), 7.33 (d, J = 8.0 Hz, 2H), 6.35 (d, J = 1.2 Hz, 1H), 5.84 (d, J = 1.3 Hz, 1H), 4.55 (s, 2H), 4.12 – 3.99 (m, 2H), 2.50 (ddd, J = 15.0, 11.7, 5.2 Hz, 1H), 2.46 (s, 3H), 2.36 (ddd, J = 15.0, 11.7, 5.1 Hz, 1H), 1.68 – 1.61 (m, 1H), 1.52 – 1.41 (m, 1H), 1.17 (t, J = 7.1 Hz, 3H), 0.91 (t, J = 7.3 Hz, 4H). **^{13}C NMR** (125 MHz, $CDCl_3$) δ 165.91 , 148.86 , 145.94 , 143.81 , 139.51 , 138.67 , 138.06 , 135.49 , 132.12 , 129.55 , 128.37 , 102.32 , 95.20 , 61.09 , 50.03 , 34.24 , 23.43 , 21.60 , 14.16 , 13.96 . **HRMS (ESI)** m/z Calcd for $[C_{22}H_{25}I_2NNaO_4S, M + Na]^+$: 675.9486; Found: 675.9488.

Optical Rotation: $[\alpha]^{25}_D$ - 15.0 (c = 1.0, $CHCl_3$). 95% ee (HPLC condition: Chiralpak AD-H column, n-Hexane/i-PrOH = 95:5, flow rate = 1.0 mL/min, wavelength = 254 nm, t_R = 14.46 min for major isomer, t_R = 15.78 for minor isomer).

Supplementary Figure 55. HPLC Trace of **9ga**.

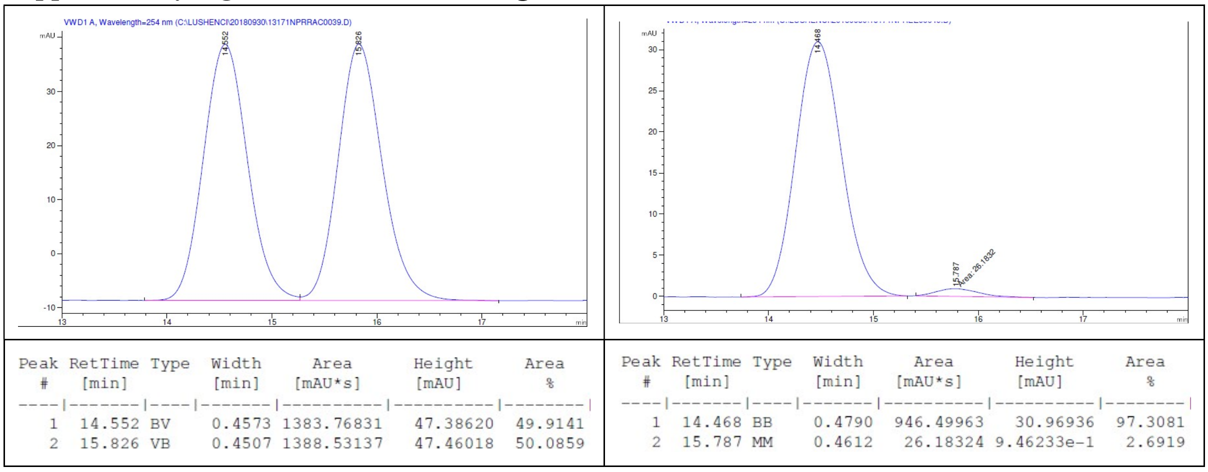

Peak #	RetTime [min]	Type	Width [min]	Area [mAU*s]	Height [mAU]	Area %
1	14.552	BV	0.4573	1383.76831	47.38620	49.9141
2	15.826	VB	0.4507	1388.53137	47.46018	50.0859

Peak #	RetTime [min]	Type	Width [min]	Area [mAU*s]	Height [mAU]	Area %
1	14.468	BB	0.4790	946.49963	30.96936	97.3081
2	15.787	MM	0.4612	26.18324	9.46233e-1	2.6919

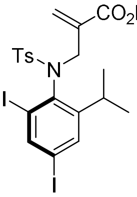

9ha. Syrup. **^{1}H NMR** (500 MHz, $CDCl_3$) δ 8.02 (d, J = 2.0 Hz, 1H), 7.82 (d, J = 8.1 Hz, 2H), 7.60 (d, J = 2.0 Hz, 1H), 7.33 (d, J = 8.0 Hz, 2H), 6.38 (d, J = 1.2 Hz, 1H), 5.81 (s, 1H), 4.63 (d, J = 14.3 Hz, 1H), 4.48 (d, J = 14.4 Hz, 1H), 4.07 (p, J = 7.2 Hz, 2H), 3.18 (p, J = 6.8 Hz, 1H), 2.45 (s, 3H), 1.26 (d, J = 6.6

Hz, 3H), 1.16 (t, J = 7.1 Hz, 3H), 1.11 (d, J = 6.8 Hz, 3H). **^{13}C NMR** (125 MHz, $CDCl_3$) δ 165.83 , 155.28 , 145.94 , 143.84 , 138.49 , 137.96 , 137.09 , 135.33 , 132.50 , 129.48 , 128.68 , 101.94 , 95.62 , 61.09 , 49.78 , 29.98 , 24.92 , 24.38 , 21.63 , 13.98 . **HRMS (ESI)** m/z Calcd for [$C_{22}H_{25}I_2NNaO_4S$, M + Na]$^+$: 675.9486; Found: 675.9482.

Optical Rotation: $[\alpha]^{25}_{D}$ + 15.0 (c = 1.0, $CHCl_3$). 92% ee (HPLC condition: Chiralpak AD-H column, n-Hexane/i-PrOH = 98:2, flow rate = 1.0 mL/min, wavelength = 254 nm, t_R = 16.18 min for major isomer, t_R = 21.63 for minor isomer).

Supplementary Figure 56. HPLC Trace of **9ha**.

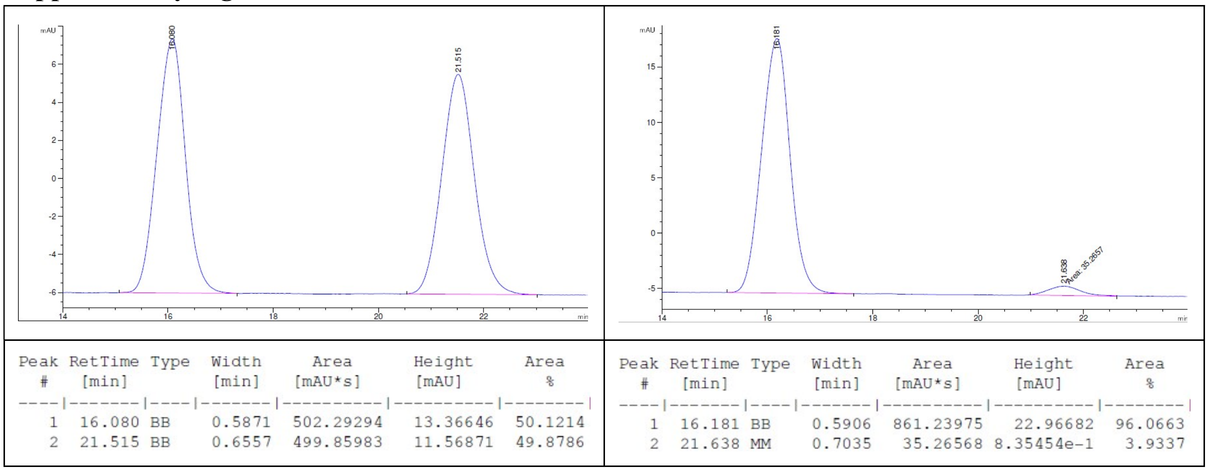

Peak #	RetTime [min]	Type	Width [min]	Area [mAU*s]	Height [mAU]	Area %
1	16.080	BB	0.5871	502.29294	13.36646	50.1214
2	21.515	BB	0.6557	499.85983	11.56871	49.8786

Peak #	RetTime [min]	Type	Width [min]	Area [mAU*s]	Height [mAU]	Area %
1	16.181	BB	0.5906	861.23975	22.96682	96.0663
2	21.638	MM	0.7035	35.26568	8.35454e-1	3.9337

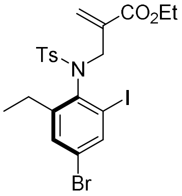

9ia. Syrup. **^{1}H NMR** (500 MHz, $CDCl_3$) δ 7.84 (d, J = 2.3 Hz, 1H), 7.79 – 7.75 (m, 2H), 7.43 (d, J = 2.3 Hz, 1H), 7.33 (d, J = 8.0 Hz, 2H), 6.36 (d, J = 1.2 Hz, 1H), 5.85 (d, J = 1.0 Hz, 1H), 4.56 (s, 1H), 4.53 (d, J = 14.3 Hz, 1H), 4.14 – 3.96 (m, 2H), 2.68 (dq, J = 15.1, 7.5 Hz, 1H), 2.52 (dq, J = 15.2, 7.6 Hz, 1H), 2.46 (s, 3H), 1.17 (td, J = 7.4, 3.5 Hz, 7H). **^{13}C NMR** (125 MHz, $CDCl_3$) δ 165.89 , 149.94 , 143.82 , 140.30 , 138.74 , 138.06 , 135.55 , 132.23 , 132.13 , 129.57 , 128.37 , 123.04 , 101.34 , 61.08 , 50.00 , 25.40 , 21.62 , 14.22 , 13.94 . **HRMS (ESI)** m/z Calcd for [$C_{21}H_{23}BrINNaO_4S$, M + Na]$^+$: 613.9468; Found: 613.9464.

Optical Rotation: $[\alpha]^{25}_{D}$ - 15.0 (c = 1.0, $CHCl_3$). 95% ee (HPLC condition: Chiralpak AD-H column, n-Hexane/i-PrOH = 98:2, flow rate = 1.0 mL/min, wavelength = 254 nm, t_R = 21.47 min for major isomer, t_R = 23.61 for minor isomer).

Supplementary Figure 57. HPLC Trace of **9ia**.

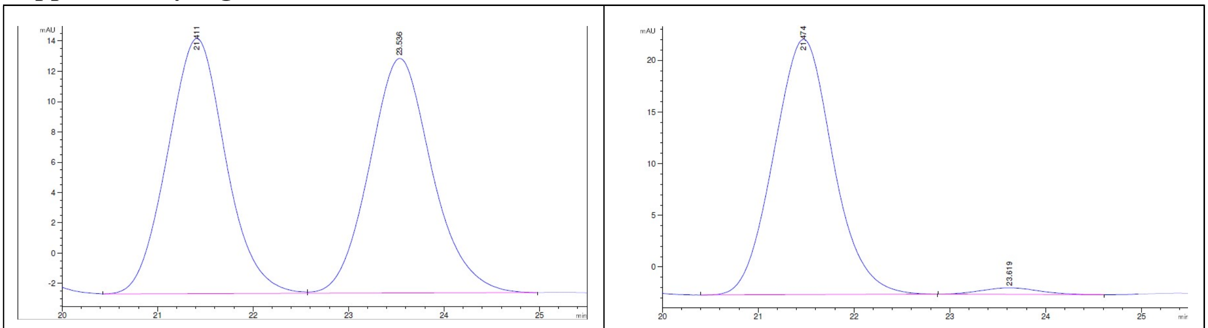

Peak #	RetTime [min]	Type	Width [min]	Area [mAU*s]	Height [mAU]	Area %
1	21.411	BV	0.6563	711.64154	16.84031	50.2233
2	23.536	VB	0.6921	705.31238	15.47550	49.7767

Peak #	RetTime [min]	Type	Width [min]	Area [mAU*s]	Height [mAU]	Area %
1	21.474	BB	0.6705	1061.72559	24.70267	97.5454
2	23.619	BB	0.4956	26.71742	6.40243e-1	2.4546

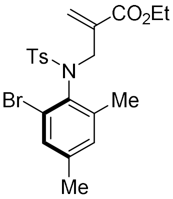

9ja. Syrup. **^{1}H NMR** (500 MHz, $CDCl_3$) δ 7.75 (d, *J* = 8.3 Hz, 2H), 7.30 (d, *J* = 8.1 Hz, 2H), 7.20 (d, *J* = 2.0 Hz, 1H), 7.03 – 6.96 (m, 1H), 6.29 (t, *J* = 0.9 Hz, 1H), 5.80 (d, *J* = 1.1 Hz, 1H), 4.64 (d, *J* = 14.1 Hz, 1H), 4.47 (d, *J* = 14.1 Hz, 1H), 4.18 – 3.95 (m, 2H), 2.45 (s, 3H), 2.28 (s, 4H), 2.27 (s, 3H), 1.17 (t, *J* = 7.1 Hz, 3H). **^{13}C NMR** (125 MHz, $CDCl_3$) δ 166.05 , 143.41 , 142.82 , 139.67 , 137.96 , 135.93 , 133.84 , 132.10 , 131.30 , 131.29 , 129.38 , 128.14 , 124.59 , 60.96 , 49.73 , 21.58 , 20.65 , 20.00 , 13.91 . **HRMS (ESI)** m/z Calcd for [$C_{21}H_{24}BrNNaO_4S$, M + Na]$^+$: 488.0501; Found: 488.0505.

Optical Rotation: $[\alpha]^{25}_{D}$ +20.0 (*c* = 1.0, $CHCl_3$). 93% ee (HPLC condition: Chiralpak OD-H column, *n*-Hexane/*i*-PrOH = 95:5, flow rate = 1.0 mL/min, wavelength = 254 nm, t_R = 9.72 min for major isomer, t_R = 10.86 for minor isomer).

Supplementary Figure 58. HPLC Trace of **9ja**.

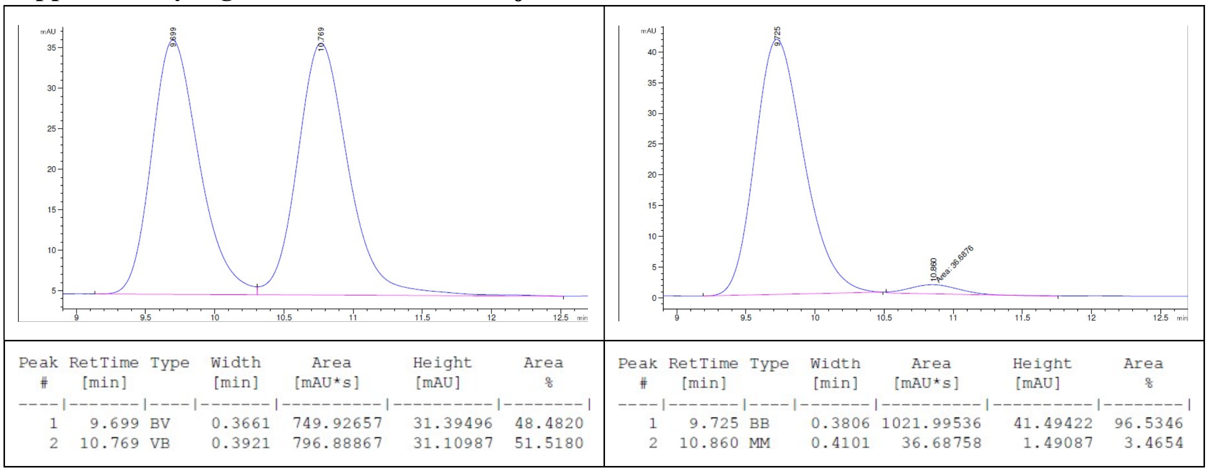

Peak #	RetTime [min]	Type	Width [min]	Area [mAU*s]	Height [mAU]	Area %
1	9.699	BV	0.3661	749.92657	31.39496	48.4820
2	10.769	VB	0.3921	796.88867	31.10987	51.5180

Peak #	RetTime [min]	Type	Width [min]	Area [mAU*s]	Height [mAU]	Area %
1	9.725	BB	0.3806	1021.99536	41.49422	96.5346
2	10.860	MM	0.4101	36.68758	1.49087	3.4654

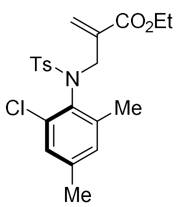

9ka. Syrup. **^{1}H NMR** (400 MHz, $CDCl_3$) δ 7.72 (d, *J* = 8.3 Hz, 2H), 7.29 (d, *J* = 8.2 Hz, 2H), 6.98 (d, *J* = 2.0 Hz, 1H), 6.96 (d, *J* = 2.0 Hz, 1H), 6.27 (d, *J* = 1.2 Hz, 1H), 5.77 (d, *J* = 1.1 Hz, 1H), 4.66 (dd, *J* = 14.1, 0.8 Hz, 1H), 4.39 (d, *J* = 14.1 Hz, 1H), 4.16 – 3.98 (m, 2H), 2.45 (s, 3H), 2.28 (s, 3H), 2.28 (s, 3H), 1.18 (t, *J* = 7.1 Hz, 3H). **^{13}C NMR** (100 MHz, $CDCl_3$) δ 165.99 , 143.38 , 142.65 , 139.40 , 137.72 , 135.92 , 134.29 , 132.31 , 131.01 , 130.63 , 129.37 , 128.64 , 127.99 , 60.95 , 49.72 , 21.57 , 20.78 , 19.65 , 13.92 . **HRMS (ESI)** m/z Calcd for $[C_{21}H_{24}ClNNaO_4S, M + Na]^+$: 444.1007; Found: 444.1004.

Optical Rotation: $[\alpha]^{25}_D$ +35.0 (*c* = 1.0, $CHCl_3$). 87% ee (HPLC condition: Chiralpak OD-H column, *n*-Hexane/*i*-PrOH = 98:2, flow rate = 1.0 mL/min, wavelength = 254 nm, t_R = 14.42 min for major isomer, t_R = 15.92 for minor isomer).

Supplementary Figure 59. HPLC Trace of **9ka**.

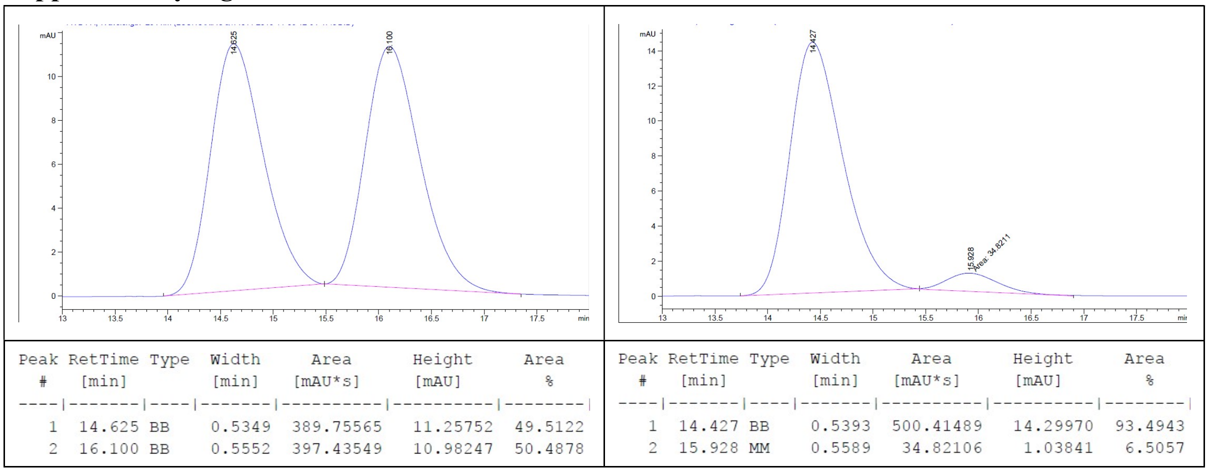

Peak #	RetTime [min]	Type	Width [min]	Area [mAU*s]	Height [mAU]	Area %
1	14.625	BB	0.5349	389.75565	11.25752	49.5122
2	16.100	BB	0.5552	397.43549	10.98247	50.4878

Peak #	RetTime [min]	Type	Width [min]	Area [mAU*s]	Height [mAU]	Area %
1	14.427	BB	0.5393	500.41489	14.29970	93.4943
2	15.928	MM	0.5589	34.82106	1.03841	6.5057

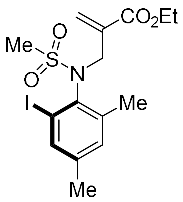

9la. Syrup. **^{1}H NMR** (400 MHz, $CDCl_3$) δ 7.48 (d, *J* = 2.0 Hz, 1H), 6.96 (d, *J* = 2.0 Hz, 1H), 6.16 (d, *J* = 1.1 Hz, 1H), 5.38 (d, *J* = 1.1 Hz, 1H), 4.64 (d, *J* = 14.1 Hz, 1H), 4.19 – 4.05 (m, 3H), 3.33 (s, 3H), 2.31 (s, 3H), 2.17 (s, 3H), 1.19 (t, *J* = 7.1 Hz, 3H). **^{13}C NMR** (100 MHz, $CDCl_3$) δ 166.28 , 140.94 , 140.13 , 138.62 , 137.23 , 135.17 , 132.60 , 131.79 , 103.07 , 61.27 , 50.46 , 42.85 , 20.38 , 20.14 , 14.14 . **HRMS (ESI)** m/z Calcd for $[C_{15}H_{20}INNaO_4S, M + Na]^+$: 460.0050; Found: 460.0054.

Optical Rotation: $[\alpha]^{25}_D$ - 40.0 (*c* = 1.0, $CHCl_3$). 91% ee (HPLC condition: Chiralpak AD-H column, *n*-Hexane/*i*-PrOH = 90:10, flow rate = 1.0 mL/min, wavelength = 254 nm, t_R = 11.16 min for major isomer, t_R = 13.97 min for minor isomer).

Supplementary Figure 60. HPLC Trace of **9la**.

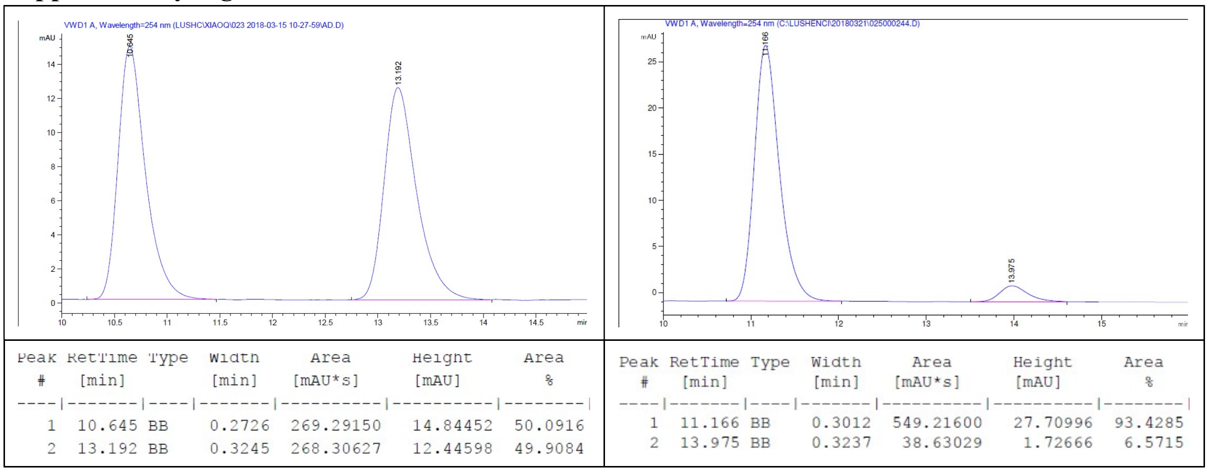

Peak #	RetTime [min]	Type	Width [min]	Area [mAU*s]	Height [mAU]	Area %
1	10.645	BB	0.2726	269.29150	14.84452	50.0916
2	13.192	BB	0.3245	268.30627	12.44598	49.9084

Peak #	RetTime [min]	Type	Width [min]	Area [mAU*s]	Height [mAU]	Area %
1	11.166	BB	0.3012	549.21600	27.70996	93.4285
2	13.975	BB	0.3237	38.63029	1.72666	6.5715

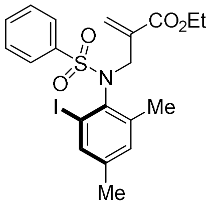

9ma. Syrup. **^{1}H NMR** (500 MHz, $CDCl_3$) δ 7.89 (dd, *J* = 8.3, 1.4 Hz, 2H), 7.63 – 7.58 (m, 1H), 7.55 – 7.50 (m, 3H), 7.02 (d, *J* = 2.0 Hz, 1H), 6.33 (d, *J* = 1.2 Hz, 1H), 5.84 (d, *J* = 1.1 Hz, 1H), 4.65 (d, *J* = 14.2 Hz, 1H), 4.57 (d, *J* = 14.1 Hz, 1H), 4.09 (dq, *J* = 10.7, 7.2 Hz, 1H), 4.01 (dq, *J* = 10.7, 7.0 Hz, 1H), 2.26 (s, 3H), 2.21 (s, 3H), 1.16 (t, *J* = 7.1 Hz, 3H). **^{13}C NMR** (125 MHz, $CDCl_3$) δ 166.09 , 142.15 , 141.26 , 139.93 , 139.14 , 137.10 , 135.84 , 132.77 , 132.29 , 131.84 , 128.91 , 128.28 , 100.44 , 61.00 , 50.08 , 20.37 , 20.35 , 13.93 . **HRMS (ESI)** m/z Calcd for $[C_{20}H_{22}INNaO_4S, M + Na]^+$: 522.0206; Found: 522.0202.

Optical Rotation: $[\alpha]^{25}_D$ - 40.0 (*c* = 1.0, $CHCl_3$). 90% ee (HPLC condition: Chiralpak OD-H column, *n*-Hexane/*i*-PrOH = 98:2, flow rate = 1.0 mL/min, wavelength = 254 nm, t_R = 16.48 min for major isomer, t_R = 18.65 for minor isomer).

Supplementary Figure 61. HPLC Trace of **9ma**.

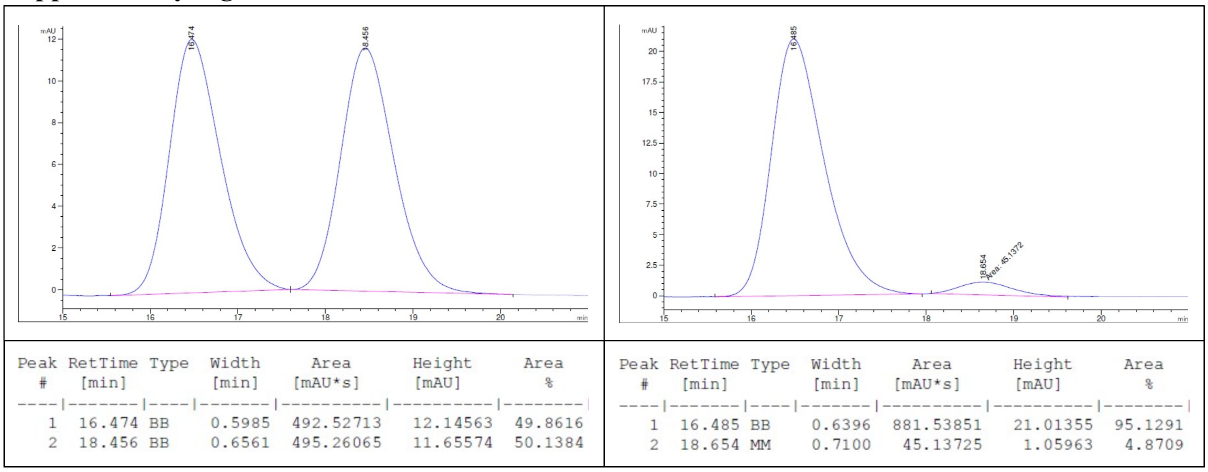

Peak #	RetTime [min]	Type	Width [min]	Area [mAU*s]	Height [mAU]	Area %
1	16.474	BB	0.5985	492.52713	12.14563	49.8616
2	18.456	BB	0.6561	495.26065	11.65574	50.1384

Peak #	RetTime [min]	Type	Width [min]	Area [mAU*s]	Height [mAU]	Area %
1	16.485	BB	0.6396	881.53851	21.01355	95.1291
2	18.654	MM	0.7100	45.13725	1.05963	4.8709

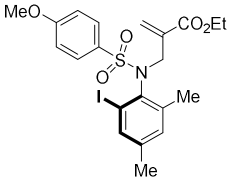

9na. Syrup. **^{1}H NMR** (500 MHz, $CDCl_3$) δ 7.86 – 7.80 (m, 2H), 7.53 (d, *J* = 2.0 Hz, 1H), 7.05 – 7.01 (m, 1H), 6.98 (d, *J* = 8.9 Hz, 2H), 6.31 (d, *J* = 1.2 Hz, 1H), 5.82 (d, *J* = 1.1 Hz, 1H), 4.60 (d, *J* = 14.3 Hz, 1H), 4.54 (d, *J* = 14.2 Hz, 1H), 4.13 – 4.06 (m, 1H), 4.06 – 3.98 (m, 1H), 3.89 (s, 3H), 2.25 (s, 3H), 2.23 (s, 3H), 1.16 (t, *J* = 7.1 Hz, 3H). **^{13}C NMR** (125 MHz, $CDCl_3$) δ 166.15 , 163.07 , 142.13 , 139.82 , 139.08 , 137.40 , 135.98 , 133.22 , 132.28 , 131.60 , 130.42 , 113.96 , 100.54 , 60.97 , 55.61 , 49.99 , 20.42 , 20.35 , 13.92 . **HRMS (ESI)** m/z Calcd for $[C_{21}H_{24}INNaO_5S, M + Na]^+$: 552.0312; Found: 552.0316.

Optical Rotation: $[\alpha]^{25}_{D}$ - 28.0 (*c* = 1.0, $CHCl_3$). 93% ee (HPLC condition: Chiralpak IC column, *n*-Hexane/*i*-PrOH = 80:20, flow rate = 1.0 mL/min, wavelength = 254 nm, t_R = 16.48 min for major isomer, t_R = 18.65 for minor isomer).

Supplementary Figure 62. HPLC Trace of **9na**.

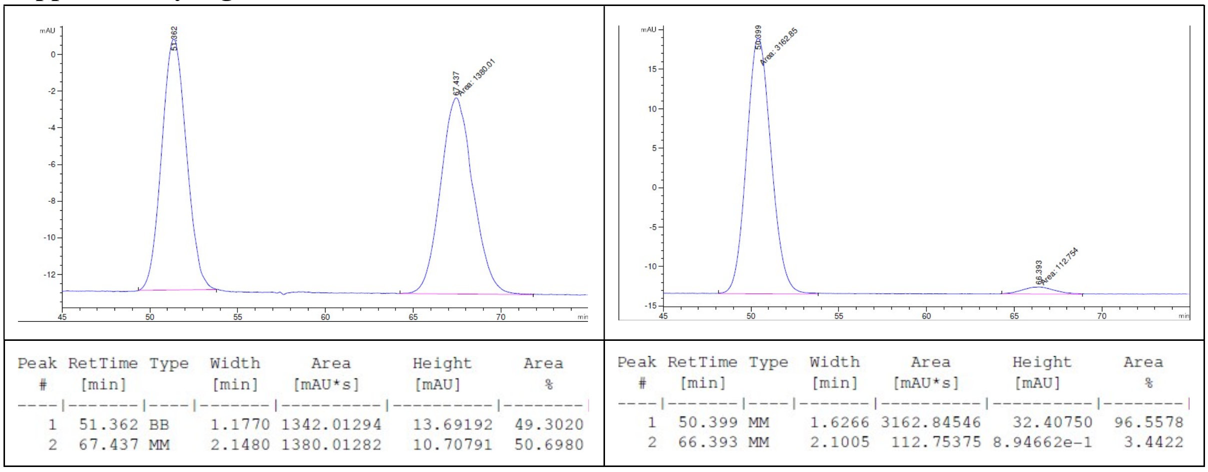

Peak #	RetTime [min]	Type	Width [min]	Area [mAU*s]	Height [mAU]	Area %
1	51.362	BB	1.1770	1342.01294	13.69192	49.3020
2	67.437	MM	2.1480	1380.01282	10.70791	50.6980

Peak #	RetTime [min]	Type	Width [min]	Area [mAU*s]	Height [mAU]	Area %
1	50.399	MM	1.6266	3162.84546	32.40750	96.5578
2	66.393	MM	2.1005	112.75375	8.94662e-1	3.4422

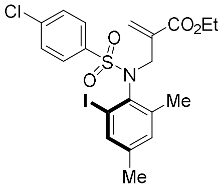

9oa. Syrup. **^{1}H NMR** (500 MHz, $CDCl_3$) δ 7.86 – 7.81 (m, 2H), 7.53 (t, *J* = 1.4 Hz, 1H), 7.51 – 7.48 (m, 2H), 7.04 – 7.02 (m, 1H), 6.32 (d, *J* = 1.1 Hz, 1H), 5.80 (s, 1H), 4.63 (d, *J* = 14.2 Hz, 1H), 4.55 (d, *J* = 14.2 Hz, 1H), 4.14 – 4.07 (m, 1H), 4.06 – 3.97 (m, 1H), 2.26 (s, 3H), 2.24 (s, 3H), 1.17 (t, *J* = 7.1, 3H). **^{13}C NMR** (125 MHz, $CDCl_3$) δ 166.01 , 142.25 , 140.13 , 139.78 , 139.24 , 139.17 , 136.82 , 135.68 , 132.36 , 131.92 , 129.79 , 129.13 , 100.22 , 61.07 , 50.20 , 20.43 , 20.36 , 13.93 . **HRMS (ESI)** m/z Calcd for $[C_{20}H_{21}ClINNaO_4S, M + Na]^+$: 555.9816; Found: 555.9818.

Optical Rotation: $[\alpha]^{25}_{D}$ - 22.0 (*c* = 1.0, $CHCl_3$). 91% ee (HPLC condition: Chiralpak AD-H

column, *n*-Hexane/*i*-PrOH = 98:2, flow rate = 1.0 mL/min, wavelength = 254 nm, t_R = 25.17 min for minor isomer, t_R = 27.32 min for major isomer).

Supplementary Figure 63. HPLC Trace of **9oa**.

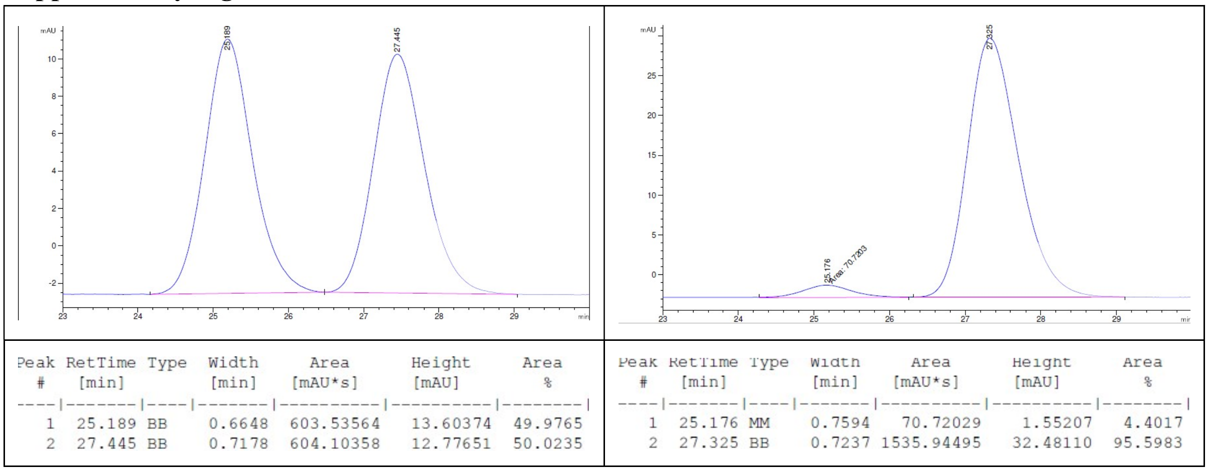

Peak #	RetTime [min]	Type	Width [min]	Area [mAU*s]	Height [mAU]	Area %
1	25.189	BB	0.6648	603.53564	13.60374	49.9765
2	27.445	BB	0.7178	604.10358	12.77651	50.0235

Peak #	RetTime [min]	Type	Width [min]	Area [mAU*s]	Height [mAU]	Area %
1	25.176	MM	0.7594	70.72029	1.55207	4.4017
2	27.325	BB	0.7237	1535.94495	32.48110	95.5983

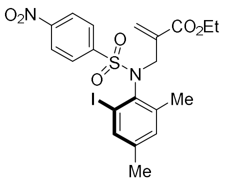

9pa. White solid. **MP:** 135-137 °C. **^{1}H NMR** (500 MHz, $CDCl_3$) δ 8.37 (d, *J* = 8.8 Hz, 2H), 8.08 (d, *J* = 8.8 Hz, 2H), 7.52 (d, *J* = 1.9 Hz, 1H), 7.06 (d, *J* = 1.9 Hz, 1H), 6.33 (d, *J* = 1.2 Hz, 1H), 5.78 (d, *J* = 1.1 Hz, 1H), 4.68 (d, *J* = 14.0 Hz, 1H), 4.60 (d, *J* = 14.0 Hz, 1H), 4.11 (dq, *J* = 10.9, 7.2 Hz, 1H), 4.04 (dq, *J* = 10.9, 7.1 Hz, 1H), 2.27 (s, 3H), 2.26 (s, 3H), 1.18 (t, *J* = 7.1 Hz, 3H). **^{13}C NMR** (125 MHz, $CDCl_3$) δ 165.87 , 150.15 , 146.73 , 142.35 , 140.53 , 139.25 , 136.20 , 135.34 , 132.53 , 132.28 , 129.64 , 124.12 , 99.91 , 61.17 , 50.54 , 20.44 , 20.39 , 13.96 . **HRMS (ESI)** m/z Calcd for [$C_{20}H_{21}IN_2NaO_6S$, M + Na]$^+$: 567.0057; Found: 567.0054.

Optical Rotation: $[\alpha]^{25}_D$ - 17.0 (*c* = 1.0, $CHCl_3$). 91% ee (HPLC condition: Chiralpak IC column, *n*-Hexane/*i*-PrOH = 70:30, flow rate = 1.0 mL/min, wavelength = 254 nm, t_R = 26.38 min for major isomer, t_R = 44.70 for minor isomer).

Supplementary Figure 64. HPLC Trace of **9pa**.

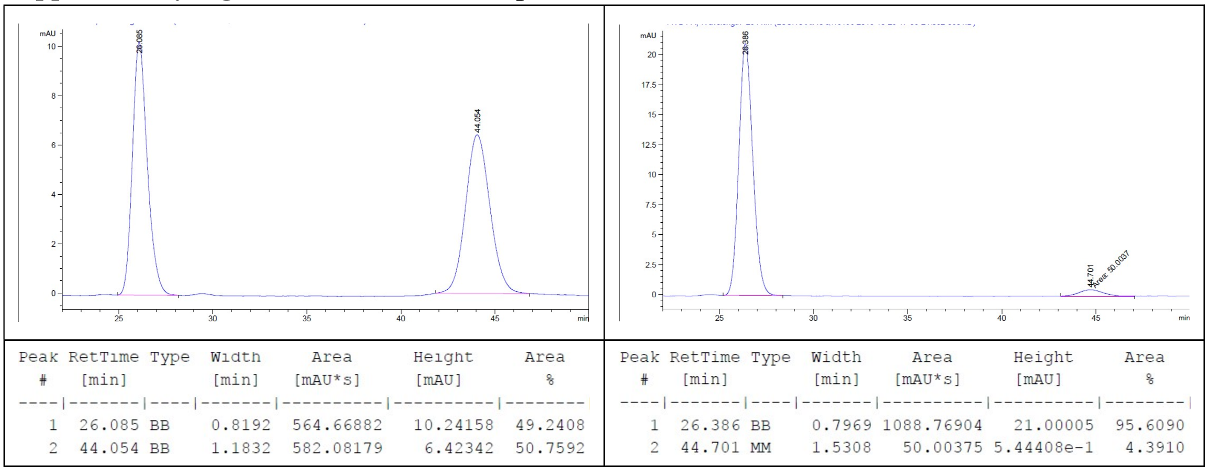

Peak #	RetTime [min]	Type	Width [min]	Area [mAU*s]	Height [mAU]	Area %
1	26.085	BB	0.8192	564.66882	10.24158	49.2408
2	44.054	BB	1.1832	582.08179	6.42342	50.7592

Peak #	RetTime [min]	Type	Width [min]	Area [mAU*s]	Height [mAU]	Area %
1	26.386	BB	0.7969	1088.76904	21.00005	95.6090
2	44.701	MM	1.5308	50.00375	5.44408e-1	4.3910

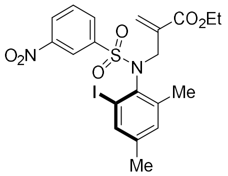

9qa. White solid. **MP:** 142-144 °C. **^{1}H NMR** (500 MHz, $CDCl_3$) δ 8.74 (t, *J* = 2.0 Hz, 1H), 8.46 (ddd, *J* = 8.2, 2.2, 1.0 Hz, 1H), 8.21 (dt, *J* = 7.8, 1.4 Hz, 1H), 7.75 (t, *J* = 8.0 Hz, 1H), 7.51 (d, *J* = 2.0 Hz, 1H), 7.07 (d, *J* = 2.1 Hz, 1H), 6.34 (d, *J* = 1.1 Hz, 1H), 5.79 (d, *J* = 1.0 Hz, 1H), 4.67 (d, *J* = 14.1 Hz, 1H), 4.59 (d, *J* = 14.1 Hz, 1H), 4.11 (dt, *J* = 10.8, 7.1 Hz, 1H), 4.02 (dq, *J* = 10.8, 7.1 Hz, 1H), 2.29 (s, 3H), 2.27 (s, 3H), 1.17 (t, *J* = 7.1 Hz, 3H). **^{13}C NMR** (125 MHz, $CDCl_3$) δ 165.86 , 148.20 , 143.15 , 142.33 , 140.56 , 139.26 , 136.33 , 135.35 , 133.95 , 132.57 , 132.25 , 130.15 , 127.21 , 123.70 , 99.72 , 61.15 , 50.56 , 20.46 , 20.40 , 13.94 . **HRMS (ESI)** m/z Calcd for $[C_{20}H_{21}IN_2NaO_6S, M + Na]^+$: 567.0057; Found: 567.0060.

Optical Rotation: $[\alpha]^{25}_D$ - 65.0 (*c* = 1.0, $CHCl_3$). 93% ee (HPLC condition: Chiralpak IC column, *n*-Hexane/*i*-PrOH = 90:10, flow rate = 1.0 mL/min, wavelength = 254 nm, t_R = 54.67 min for major isomer, t_R = 58.46 for minor isomer).

Supplementary Figure 65. HPLC Trace of **9qa**.

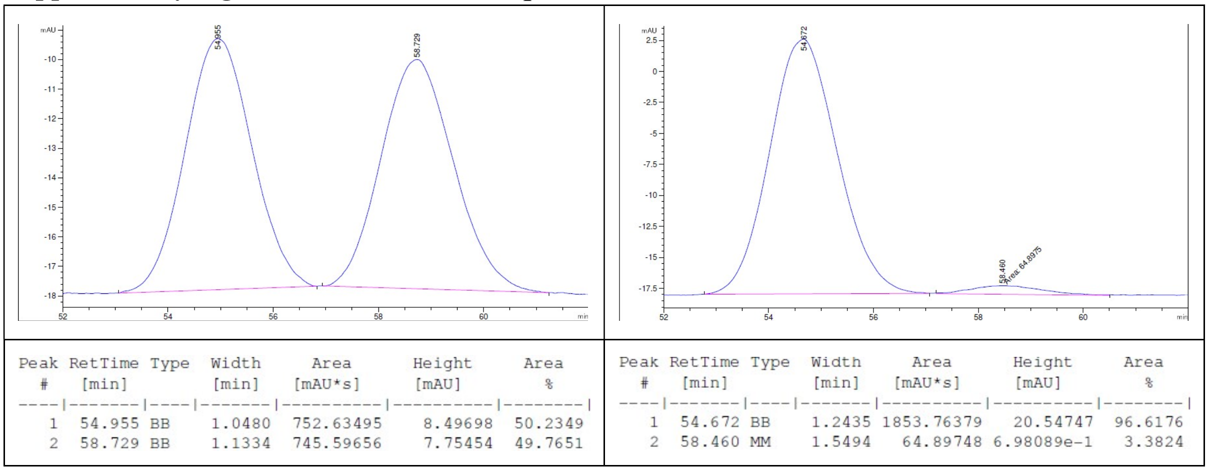

Peak #	RetTime [min]	Type	Width [min]	Area [mAU*s]	Height [mAU]	Area %
1	54.955	BB	1.0480	752.63495	8.49698	50.2349
2	58.729	BB	1.1334	745.59656	7.75454	49.7651

Peak #	RetTime [min]	Type	Width [min]	Area [mAU*s]	Height [mAU]	Area %
1	54.672	BB	1.2435	1853.76379	20.54747	96.6176
2	58.460	MM	1.5494	64.89748	6.98089e-1	3.3824

The procedure for the synthesis of 11, 12 and 13:

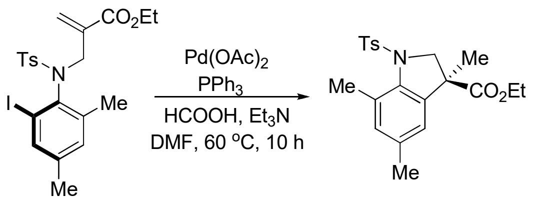

10ba: 92% ee **11**: 72%, 83% ee
90% Chirality transfer

To a dry Schlenk tube equipped with a magnetic stir bar, was added **10ba** (0.1 mmol), $Pd(OAc)_2$ (0.005 mmol), PPh_3 (0.01 mmol). The tube was closed with a septum, evacuated, and refilled with argon. Dry DMF (0.5 mL) was added and the reaction mixture was then stirred at 25 °C for 10 minutes. After that, HCOOH (0.2 mmol) and Et_3N (0.25 mmol) were added and the mixture was stirred at 60 °C for 10 hours. Upon the reaction completed, the mixture was purified via column chromatography on silica gel to afford the desired product **11**.

Syrup, **^{1}H NMR** (500 MHz, CD_2Cl_2) δ 7.58 (d, J = 8.3 Hz, 2H), 7.28 (d, J = 8.0 Hz, 2H), 7.00 (d, J = 1.7 Hz, 1H), 6.98 (d, J = 1.7 Hz, 1H), 4.66 (d, J = 12.8 Hz, 1H), 4.01 (qq, J = 7.2, 3.7 Hz, 2H), 3.87 (d, J = 12.8 Hz, 1H), 2.44 (s, 6H), 2.36 (s, 3H), 1.30 (s, 3H), 1.24 (t, J = 7.1 Hz, 3H). **^{13}C NMR** (125 MHz, CD_2Cl_2) δ 173.45 , 143.90 , 138.64 , 138.38 , 136.11 , 135.95 , 132.13 , 129.78 , 129.44 , 127.48 , 122.80 , 61.32 , 61.06 , 51.43 , 25.38 , 21.30 , 20.81 , 20.23 , 13.79 . **HRMS (ESI)** m/z Calcd for $[C_{21}H_{25}NNaO_4S, M + Na]^+$: 410.1396; Found: 410.1392.

Optical Rotation: $[\alpha]^{25}_D$ - 12.0 (c = 1.0, $CHCl_3$). 83% ee (HPLC condition: Chiralpak OD-H column, n-Hexane/i-PrOH = 98:2, flow rate = 1.0 mL/min, wavelength = 254 nm, t_R = 13.42 min for minor isomer, t_R = 15.83 min for major isomer).

Supplementary Figure 66. HPLC Trace of **11**.

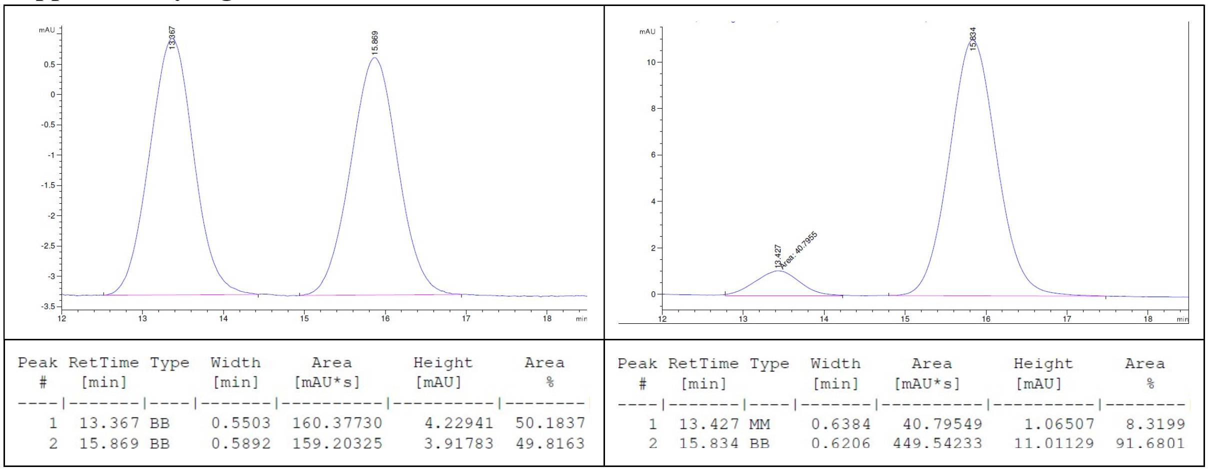

Peak #	RetTime [min]	Type	Width [min]	Area [mAU*s]	Height [mAU]	Area %
1	13.367	BB	0.5503	160.37730	4.22941	50.1837
2	15.869	BB	0.5892	159.20325	3.91783	49.8163

Peak #	RetTime [min]	Type	Width [min]	Area [mAU*s]	Height [mAU]	Area %
1	13.427	MM	0.6384	40.79549	1.06507	8.3199
2	15.834	BB	0.6206	449.54233	11.01129	91.6801

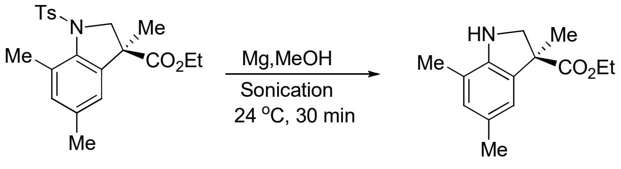

11, 83% ee **12**: 81%, 83% ee

To an oven-dried Schlenk tube equipped with a magnetic stir bar was added the **11** (0.05 mmol) and Mg-powder (18.0 mg, 15 equiv.) under Ar atmosphere. Absolute MeOH (1.4 mL) was added and the reaction was capped with a septum, then sonicated at room temperature until full consumption of starting material as monitored by TLC (around 30 min). After cooling to 0 °C, the reaction mixture was slowly quenched with saturated NH_4Cl. The aqueous phases were extracted with DCM (3 × 10 mL). The combined organic phase was washed with saturated brine (20 mL), dried over Na_2SO_4 and concentrated under reduced pressure. The residue was purified by silica gel column chromatography (hexanes/ethyl acetate = 12:1) to afford **12** (81% yield).
Syrup, **^{1}H NMR** (500 MHz, CD_2Cl_2) δ 6.93 (s, 1H), 6.78 (s, 1H), 4.18 (q, J = 7.0, 2H), 4.13 (d, J = 10.0 Hz, 1H), 3.36 (d, J = 10.0 Hz, 1H), 2.26 (s, 3H), 2.13 (s, 3H), 1.54 (s, 3H), 1.29 (t, J = 7.0 Hz, 3H). **^{13}C NMR** (125 MHz, CD_2Cl_2) δ 174.67 , 146.79 , 131.78 , 129.87 , 128.35 , 121.89 , 119.42 , 60.97 , 57.28 , 52.60 , 24.34 , 20.48 , 16.38 , 13.92 . **HRMS (ESI)** m/z Calcd for $[C_{14}H_{19}NNaO_2, M + Na]^+$: 256.1308; Found: 256.1304.

Optical Rotation: $[\alpha]^{25}_D$ - 18.0 (c = 1.0, CH_2Cl_2). 83% ee (HPLC condition: Chiralpak AD-H column, n-Hexane/i-PrOH = 95:5, flow rate = 1.0 mL/min, wavelength = 254 nm, t_R = 7.89 min for major isomer, t_R = 13.39 min for minor isomer).

Supplementary Figure 67. HPLC Trace of **12**.

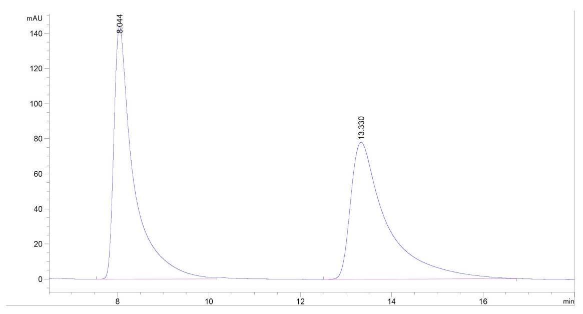

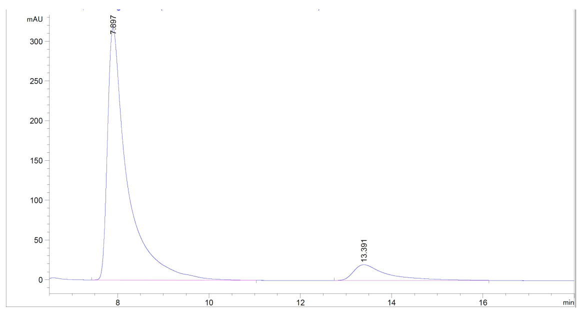

Peak #	RetTime [min]	Type	Width [min]	Area [mAU*s]	Height [mAU]	Area %
1	8.044	BV	0.4132	4336.75879	143.85620	50.2356
2	13.330	BV	0.6554	4296.08105	78.02097	49.7644

Peak #	RetTime [min]	Type	Width [min]	Area [mAU*s]	Height [mAU]	Area %
1	7.897	VB	0.4242	9834.99414	317.76624	91.4566
2	13.391	BB	0.5992	916.51141	19.84012	8.5434

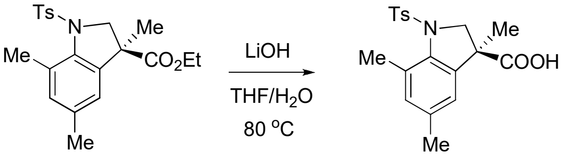

11, 83% ee

13: recrystallization
78%, 96% ee

To a solution of **11** (78 mg, 0.2 mmol) in THF (0.5 mL) were added water (0.5 mL) and LiOH (15 mg, 0.6 mmol). The mixture was allowed to stir at 80 °C overnight. After completion of the reaction (monitored by TLC), the volatiles were evaporated under reduced pressure. The residual aqueous solution was acidified with 3.0 N aqueous HCl solutions (PH < 1.0), and extracted with ethyl acetate. The combined organic layers were dried over Na_2SO_4, filtered and concentrated under

reduced pressure. After recrystallization using diethyl ehter, pure acid **13** was obtained as a white solid.

MP: 188-189 °C. ^{1}**H NMR** (400 MHz, $CDCl_3$) δ 7.44 (d, *J* = 8.3 Hz, 2H), 7.09 (d, *J* = 8.1 Hz, 2H), 6.89 (s, 1H), 6.87 (s, 1H), 4.57 (d, *J* = 12.9 Hz, 1H), 3.77 (d, *J* = 13.0 Hz, 1H), 2.37 (s, 3H), 2.24 (s, 3H), 2.23 (s, 3H), 1.23 (s, 3H). ^{13}**C NMR** (100 MHz, $CDCl_3$) δ 179.50 , 143.91 , 138.41 , 137.66 , 136.11 , 135.58 , 132.66 , 130.25 , 129.43 , 127.74 , 122.78 , 60.78 , 51.29 , 25.61 , 21.47 , 21.05 , 20.42 . **HRMS (ESI)** m/z Calcd for $[C_{19}H_{21}NNaO_4S, M + Na]^+$: 382.1083; Found: 382.1081.

Optical Rotation: $[\alpha]^{25}_{D}$ **6.0 (*c* = 0.31, $CHCl_3$).** 96% ee (HPLC condition: Chiralpak AS-H column, *n*-Hexane/*i*-PrOH = 80:20, flow rate = 1.0 mL/min, wavelength = 254 nm, t_R = 9.14 min for minor isomer, t_R = 16.41 min for major isomer).

Supplementary Figure 68. HPLC Trace of **13**.

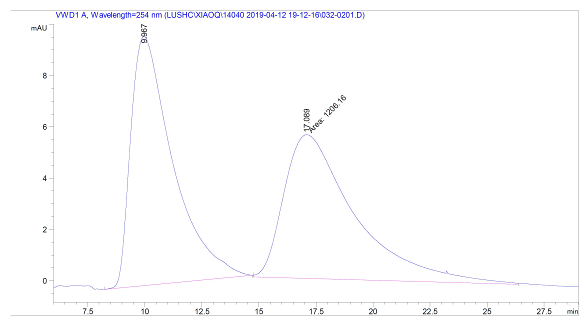

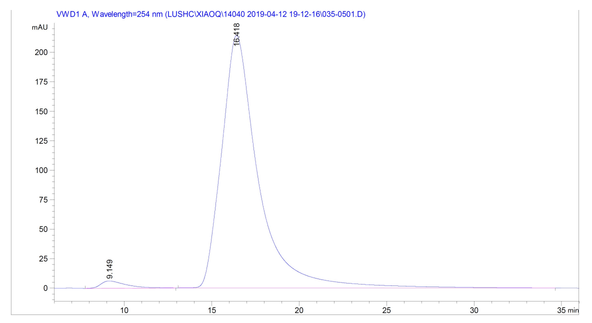

Peak #	RetTime [min]	Type	Width [min]	Area [mAU*s]	Height [mAU]	Area %
1	9.967	BB	1.7031	1242.75635	9.72328	50.7472
2	17.089	MM	3.5838	1206.16187	5.60932	49.2528

Peak #	RetTime [min]	Type	Width [min]	Area [mAU*s]	Height [mAU]	Area %
1	9.149	BB	1.3445	617.25018	6.18922	1.9290
2	16.418	BB	2.0810	3.13816e4	214.10754	98.0710

NMR spectra

Supplementary Figure 69. ^{1}H and ^{13}C NMR Spectra of **1a**.

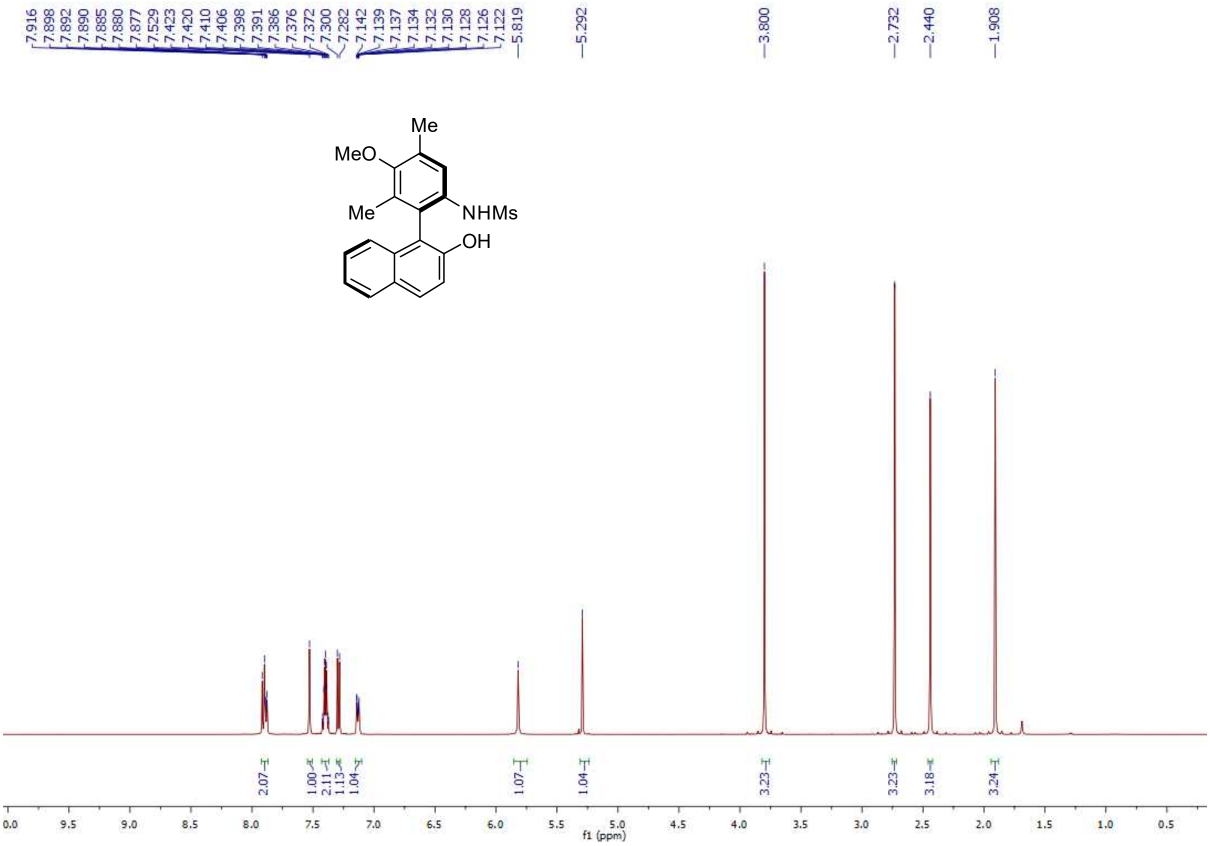

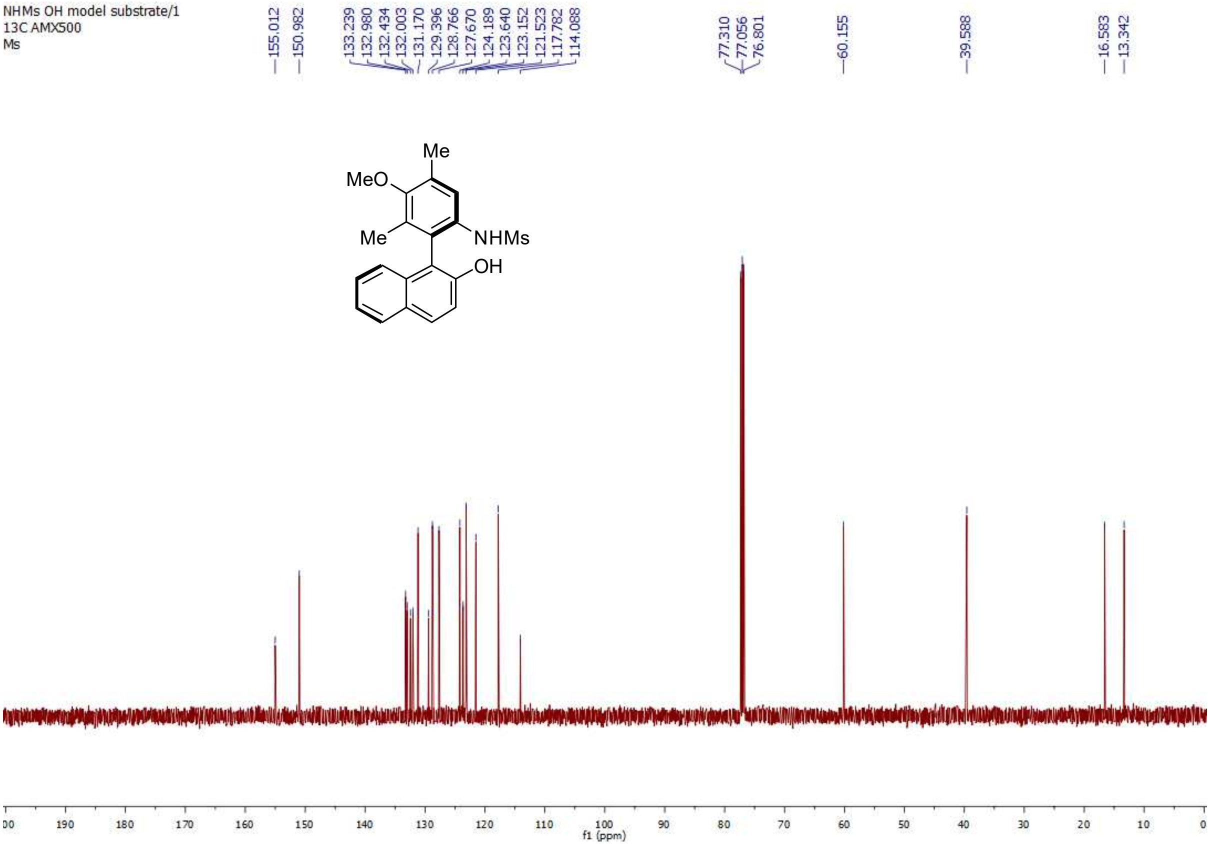

Supplementary Figure 70. ^{1}H and ^{13}C NMR Spectra of **1b**.

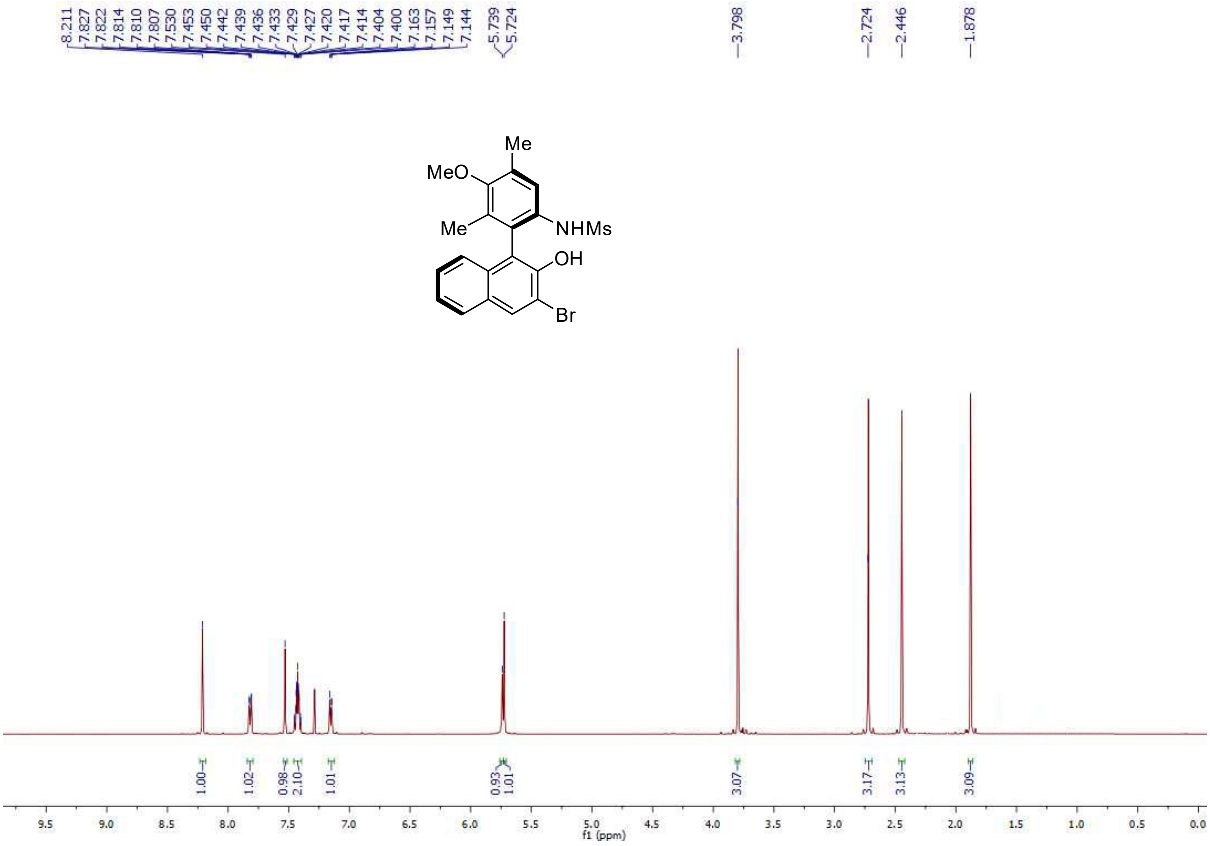

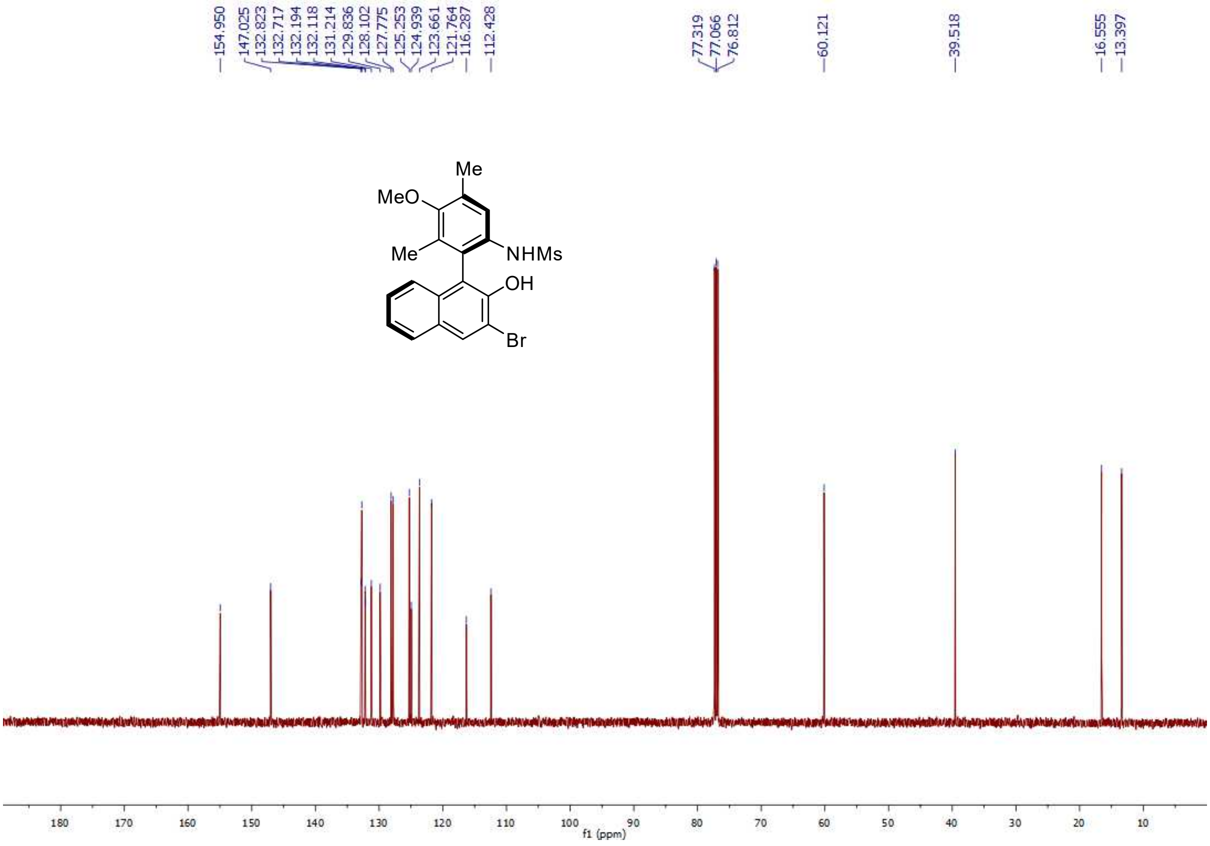

Supplementary Figure 71. ^{1}H and ^{13}C NMR Spectra of **1c**.

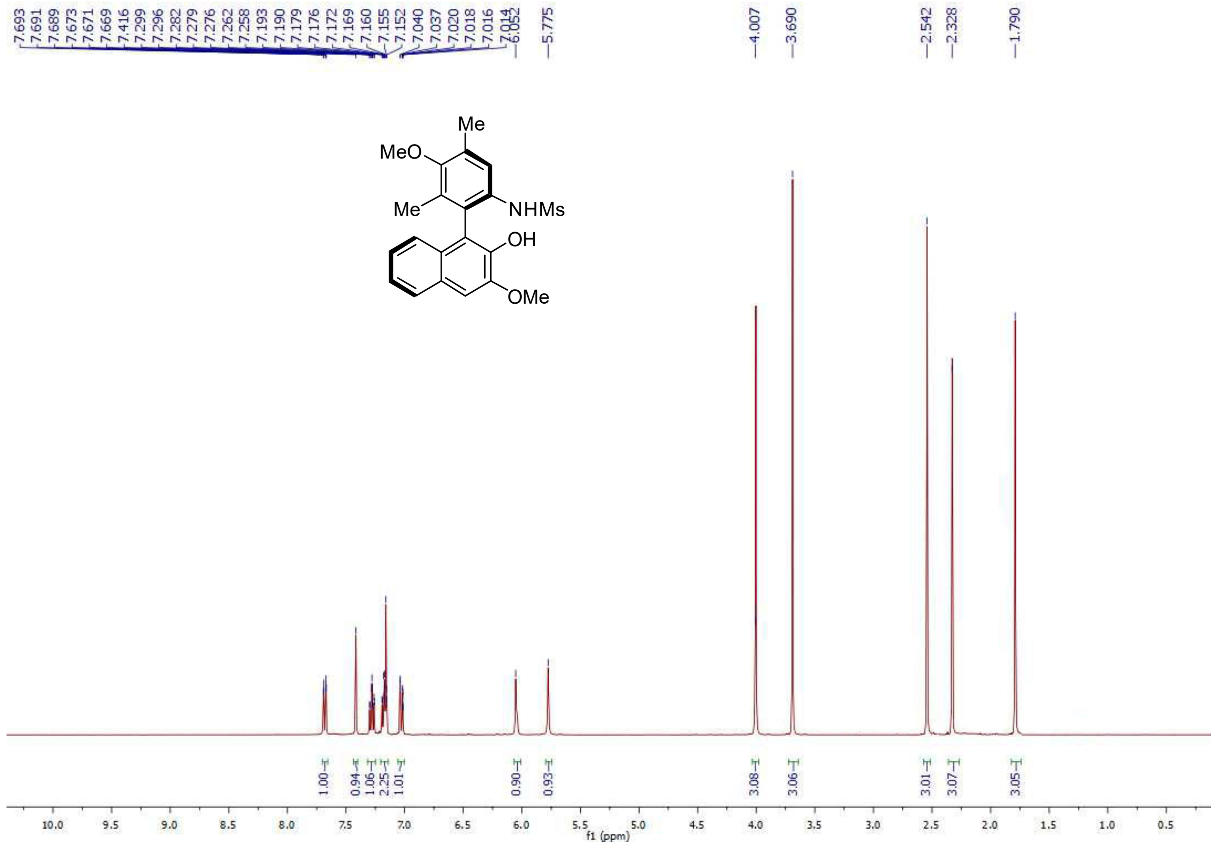

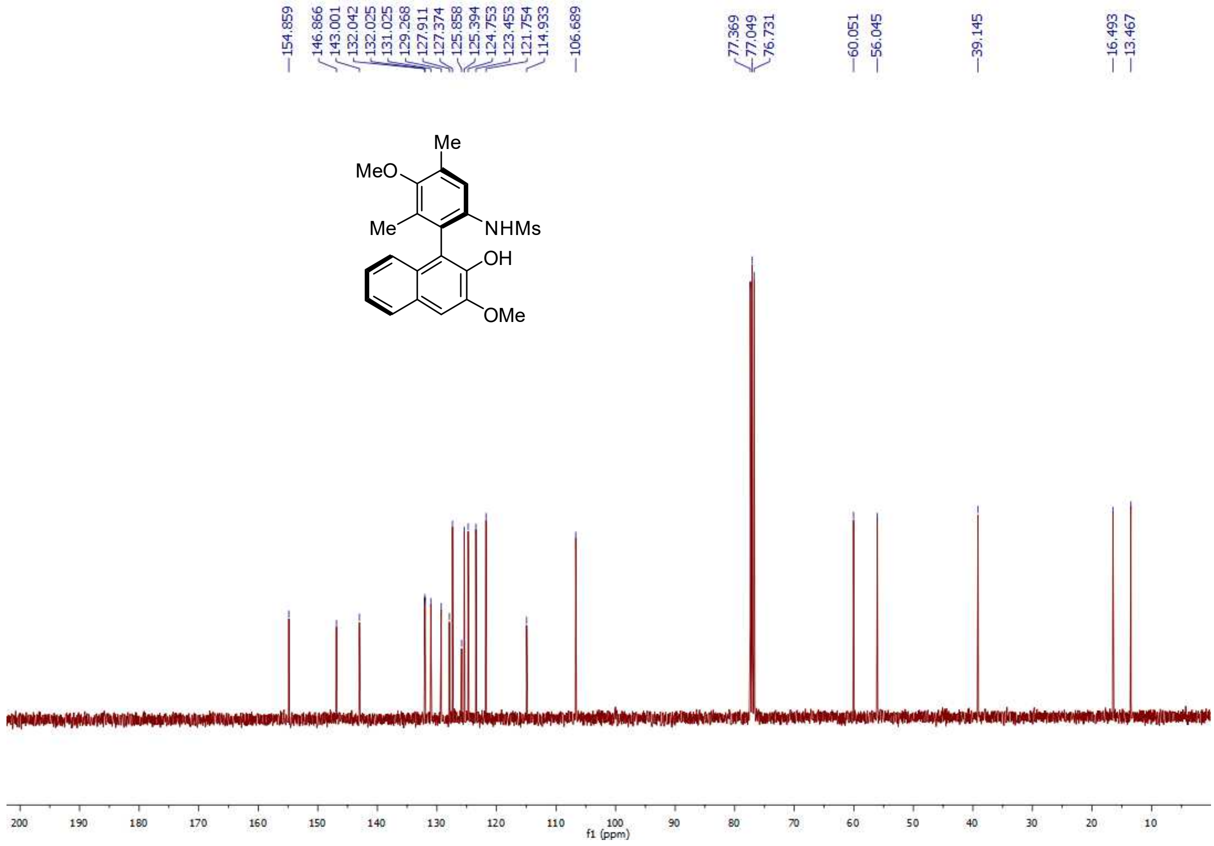

Supplementary Figure 72. ^{1}H and ^{13}C NMR Spectra of **1d**.

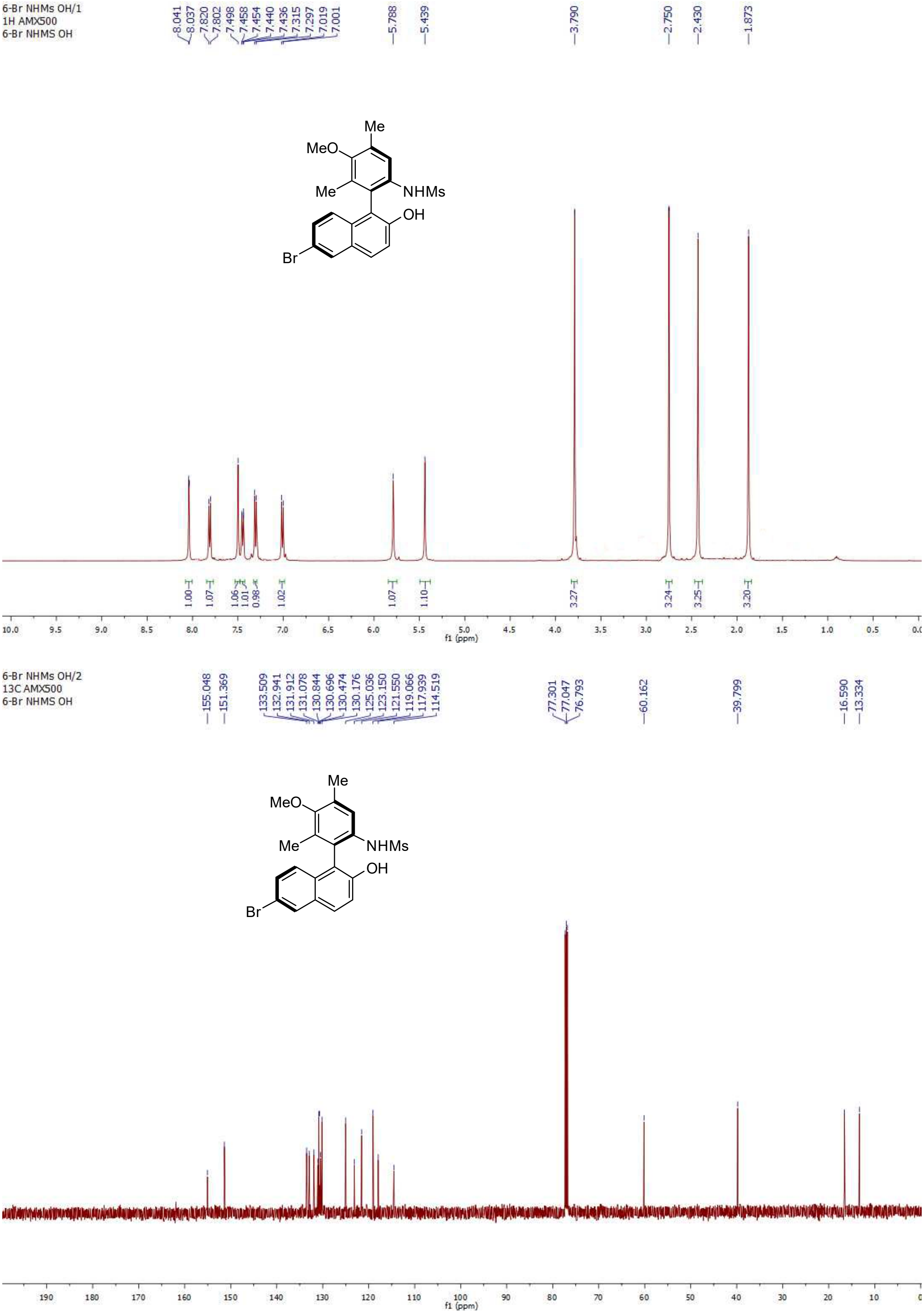

Supplementary Figure 73. ^{1}H and ^{13}C NMR Spectra of **1e**.

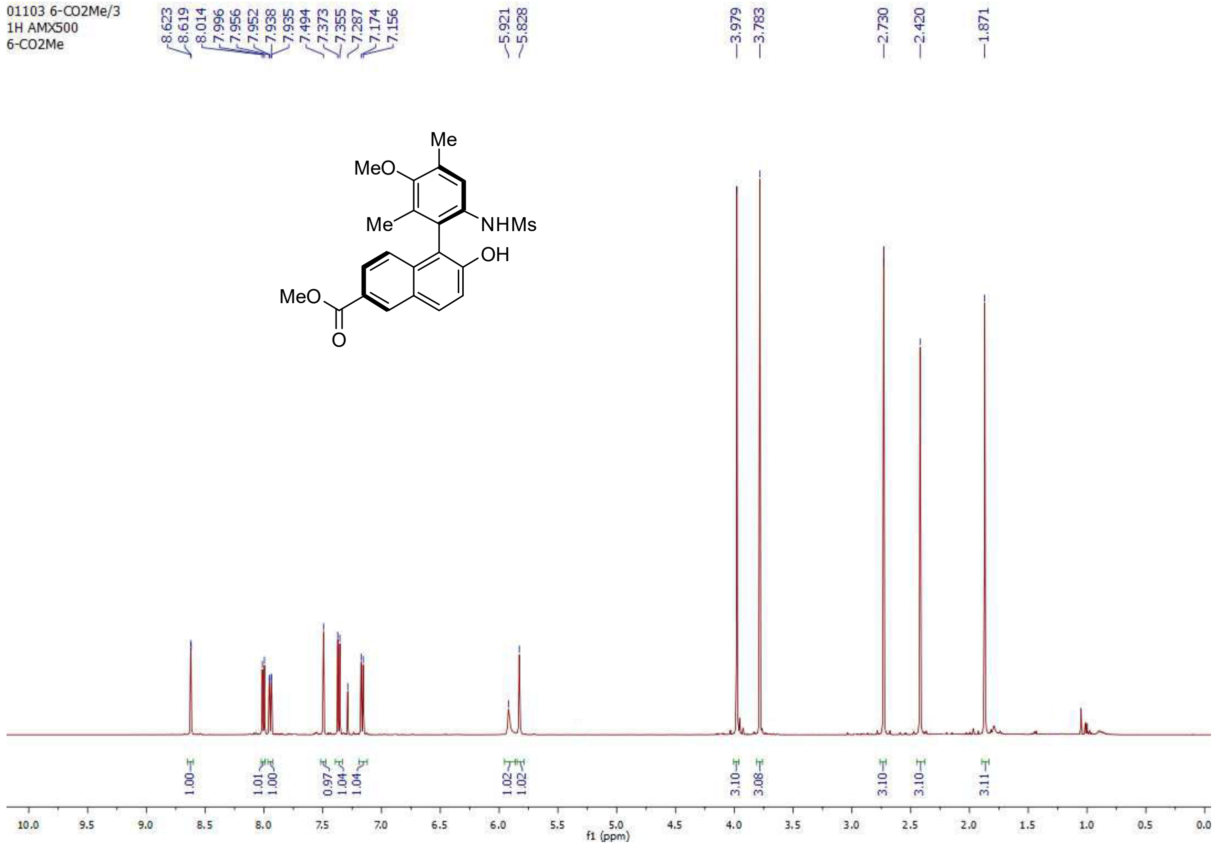

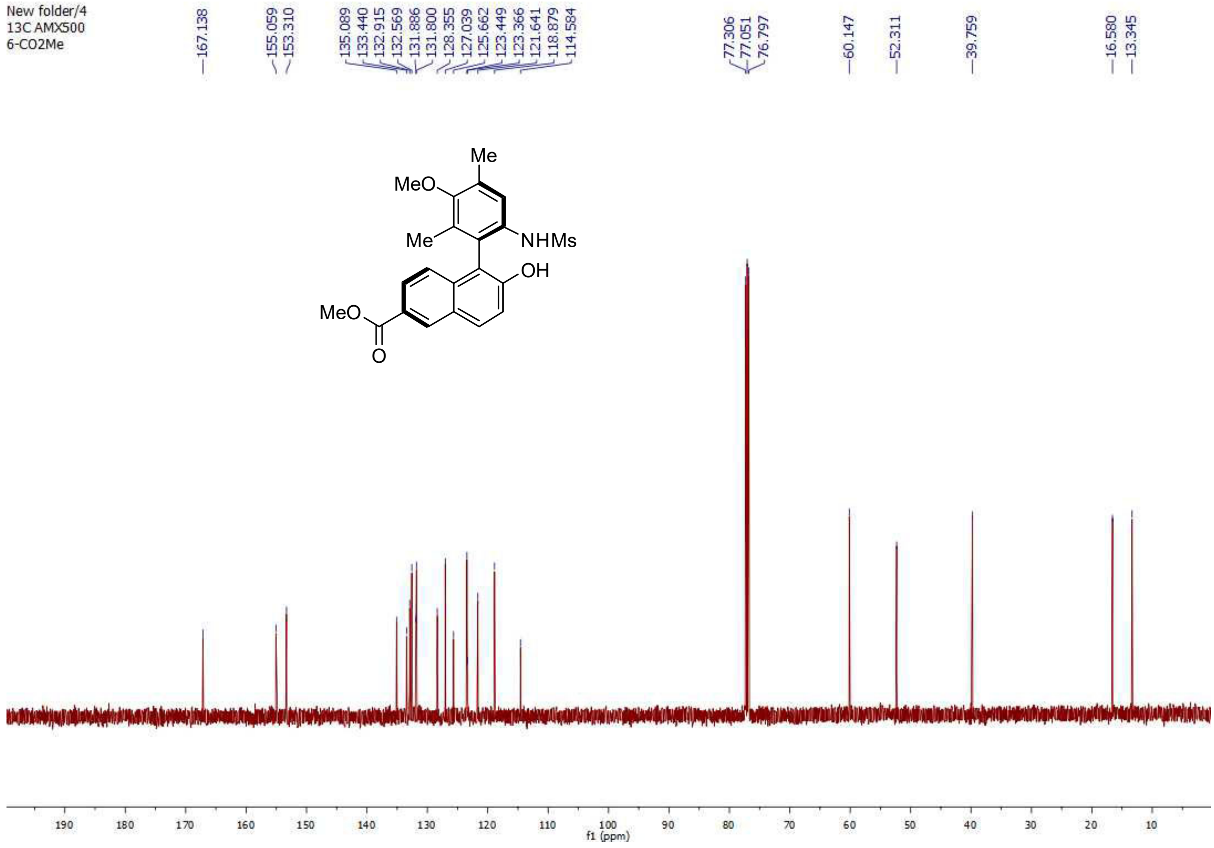

Supplementary Figure 74. ^{1}H and ^{13}C NMR Spectra of **1f**.

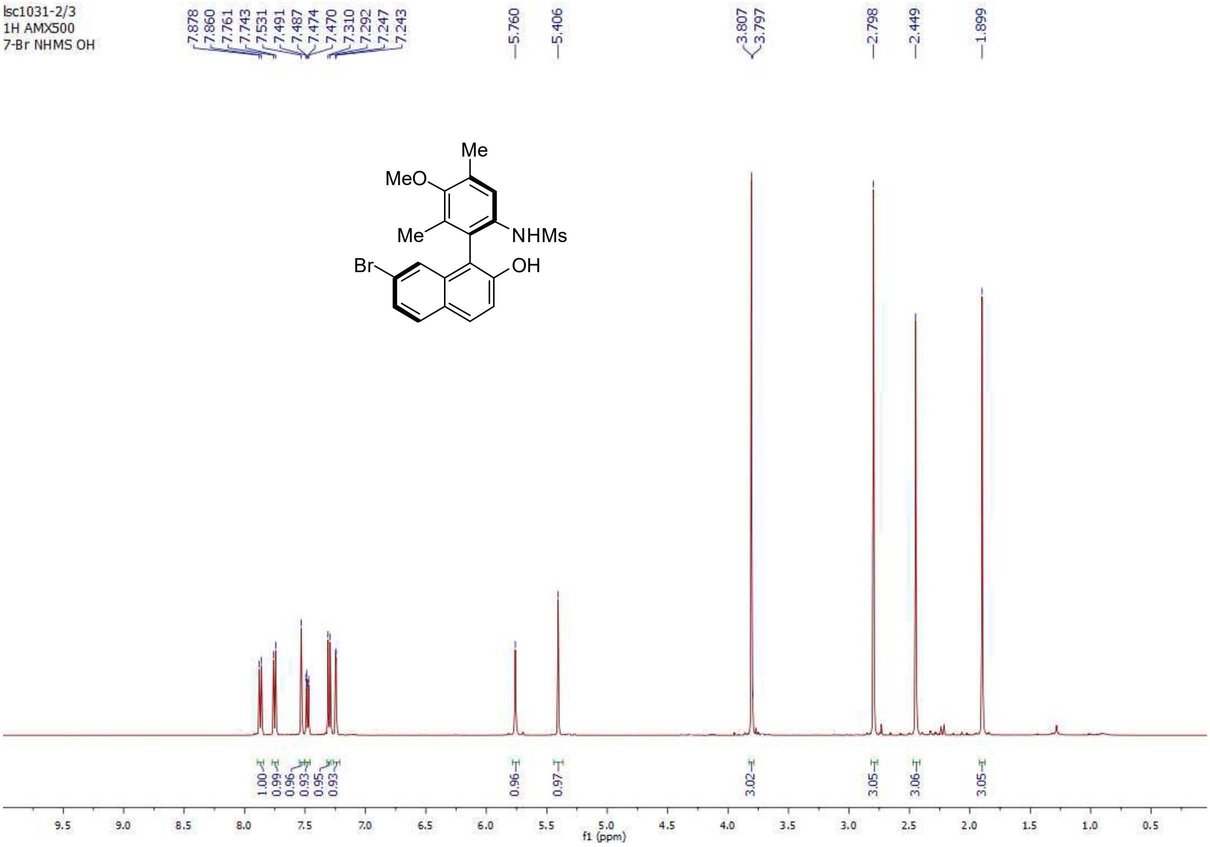

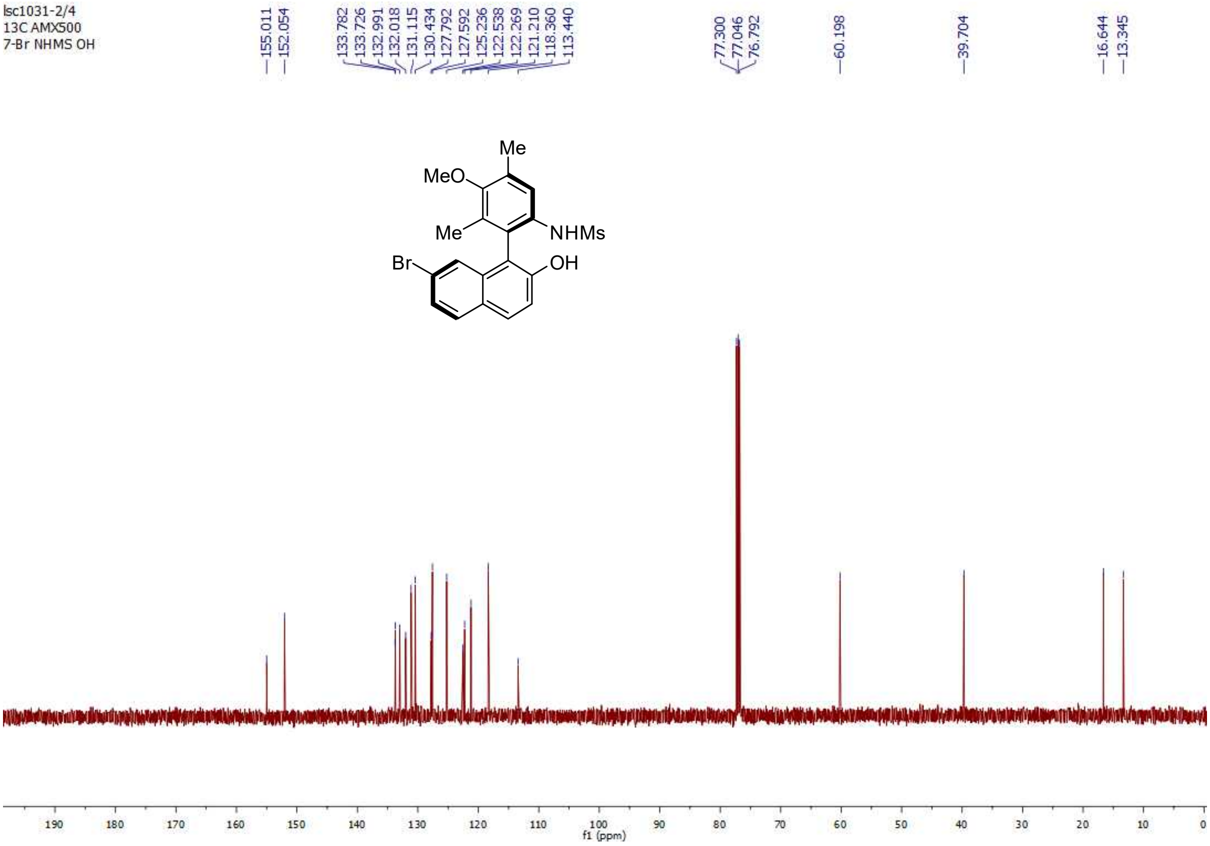

Supplementary Figure 75. ^{1}H and ^{13}C NMR Spectra of **1g**.

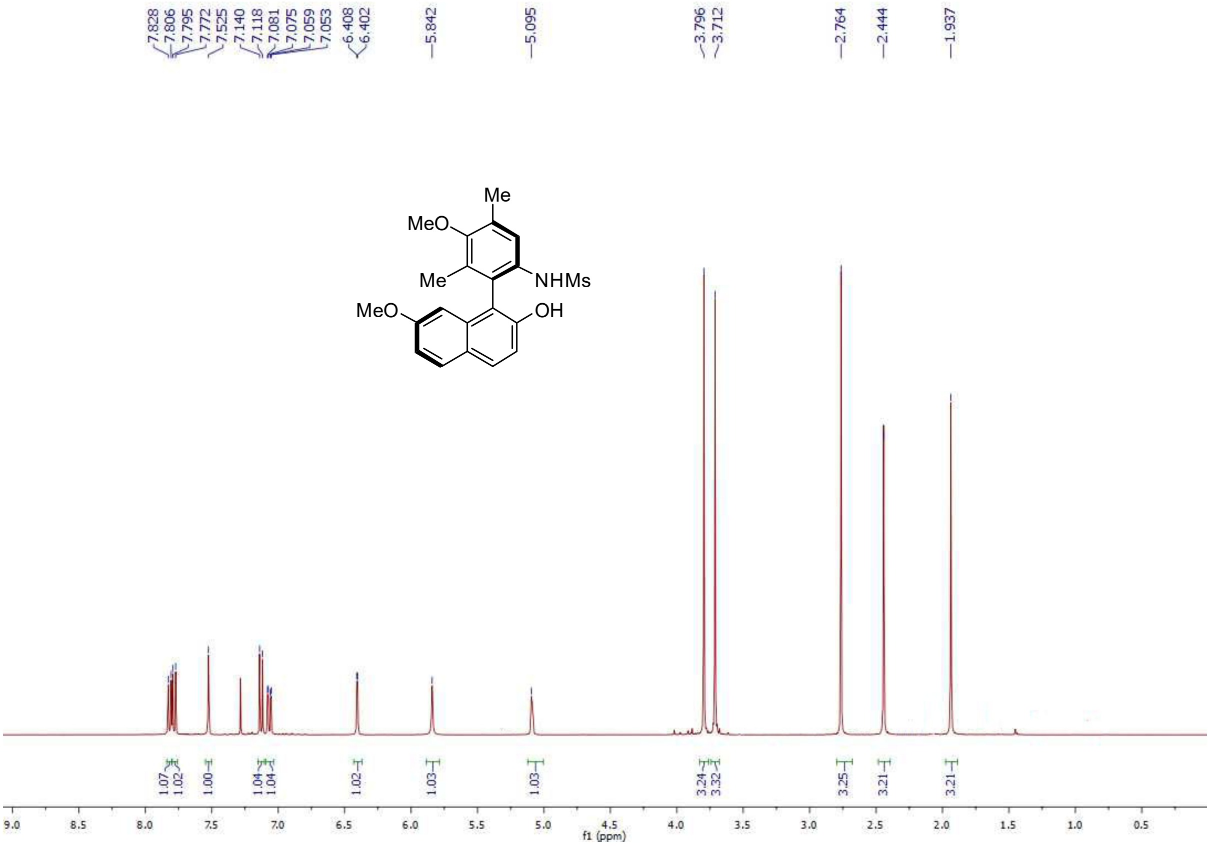

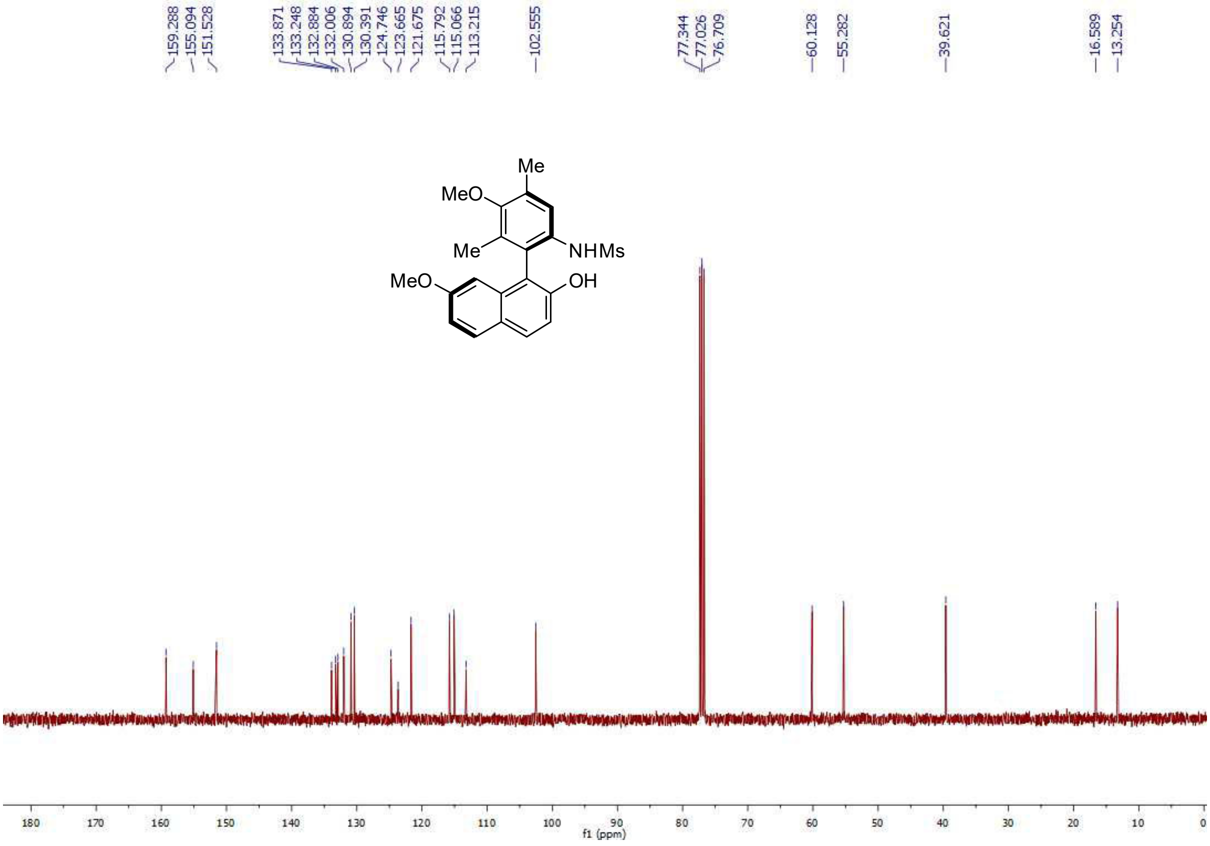

Supplementary Figure 76. ^{1}H and ^{13}C NMR Spectra of **1h**.

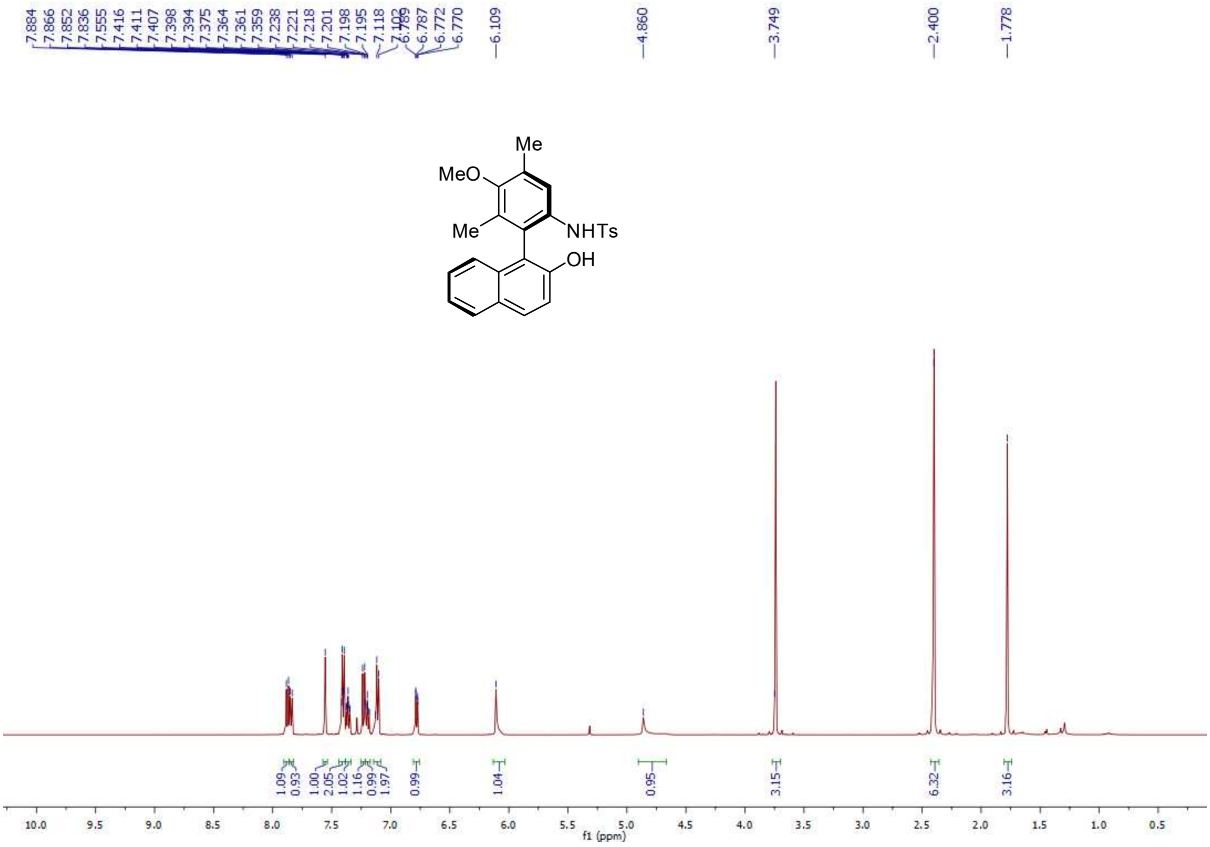

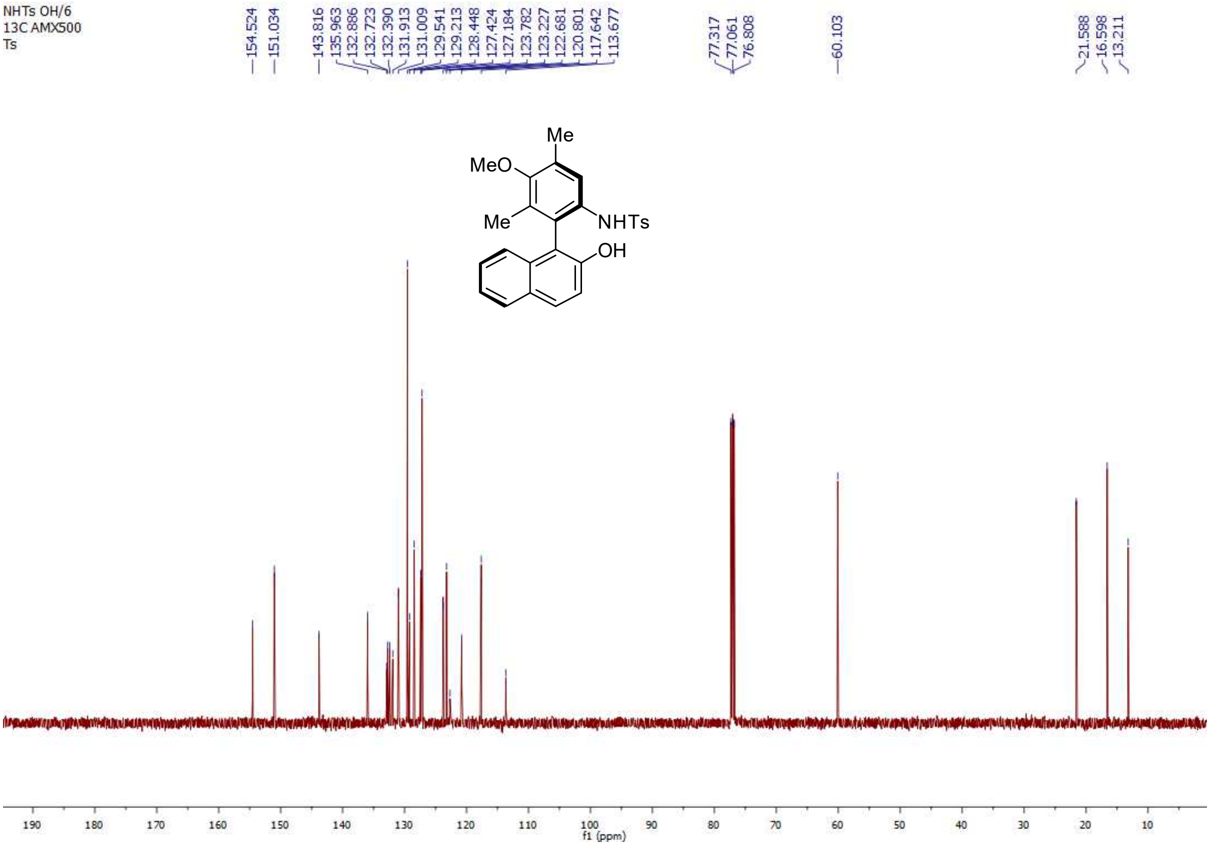

Supplementary Figure 77. ^{1}H and ^{13}C NMR Spectra of **1i**.

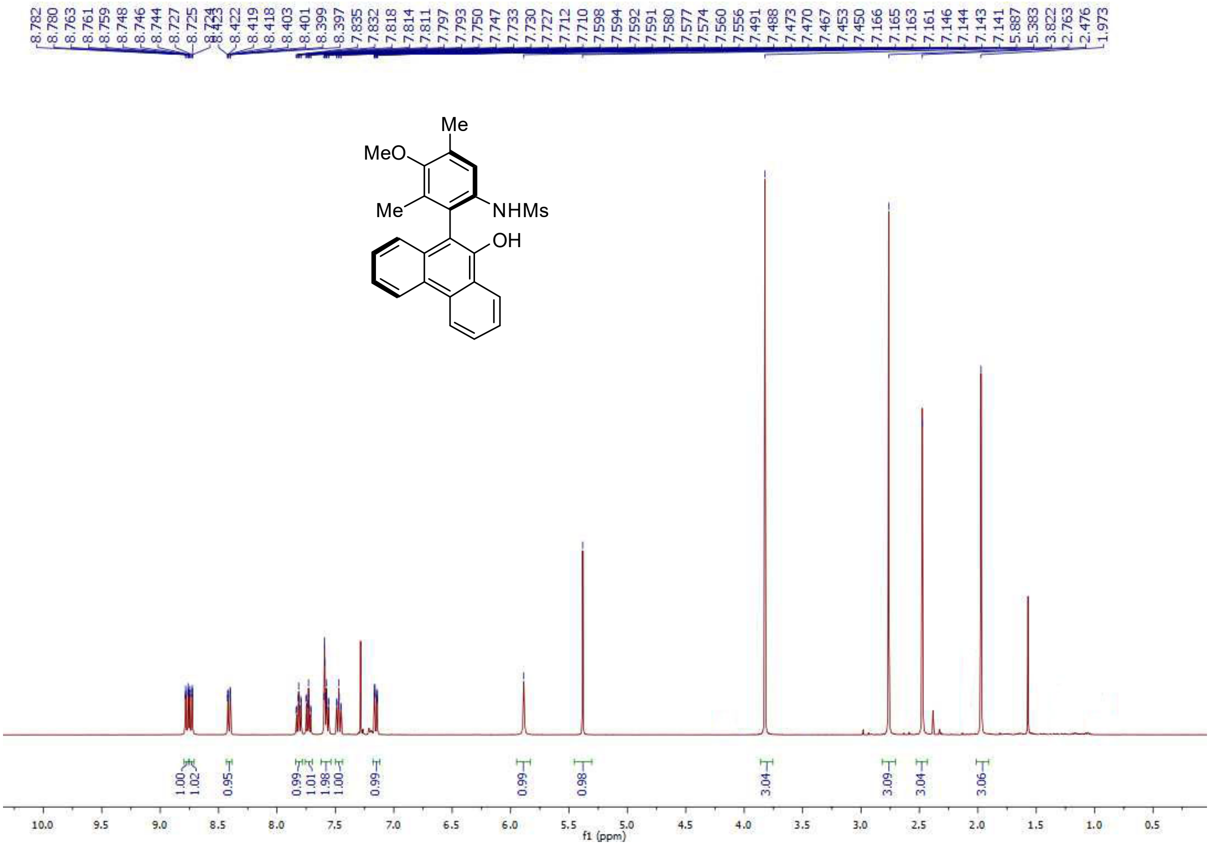

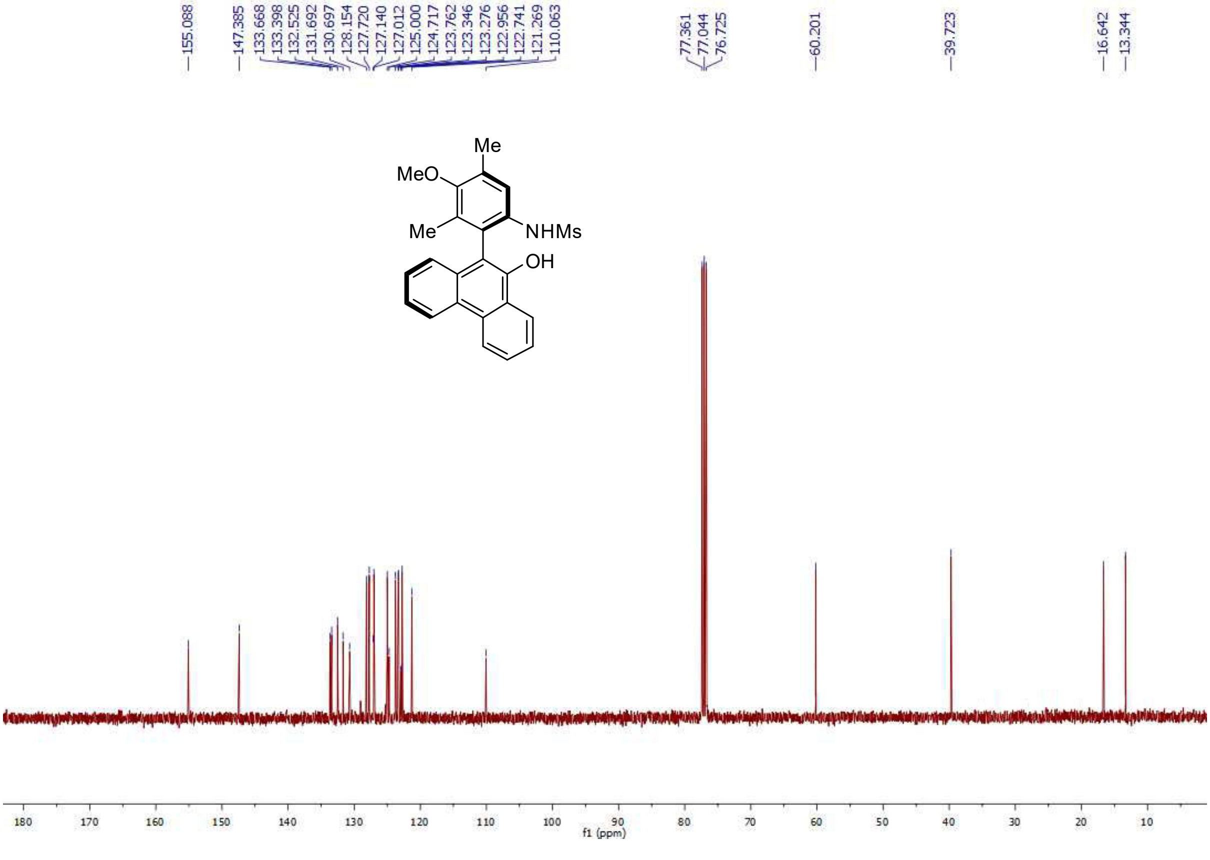

Supplementary Figure 78. ^{1}H and ^{13}C NMR Spectra of **1j**.

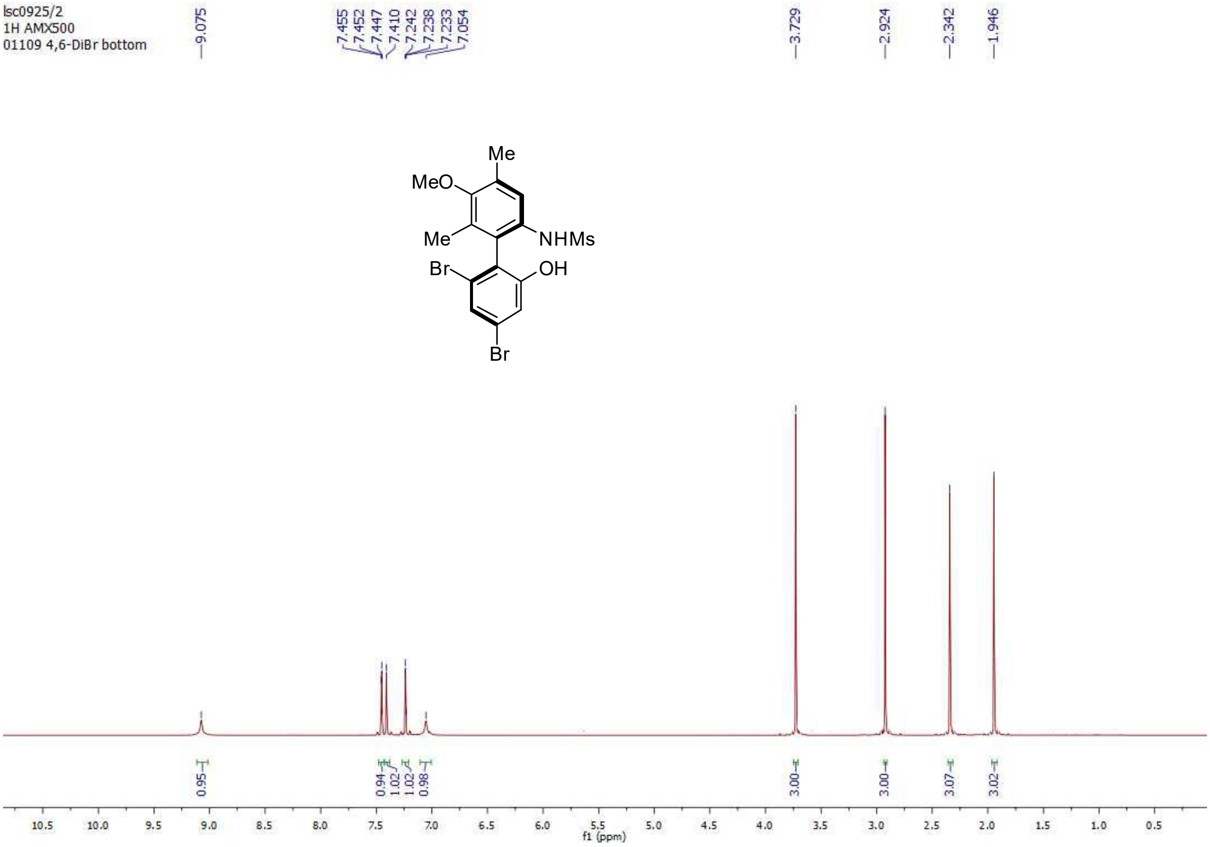

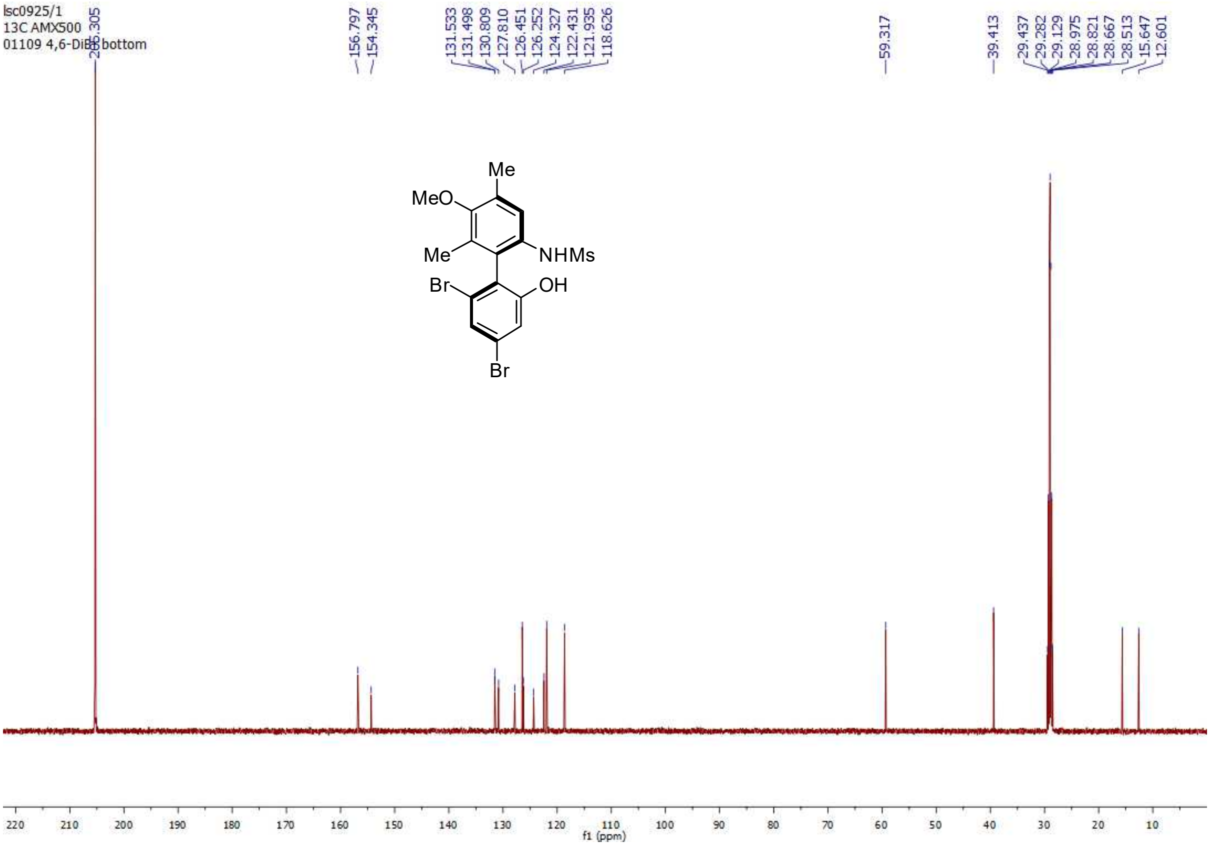

Supplementary Figure 79. ^{1}H and ^{13}C NMR Spectra of **1k**.

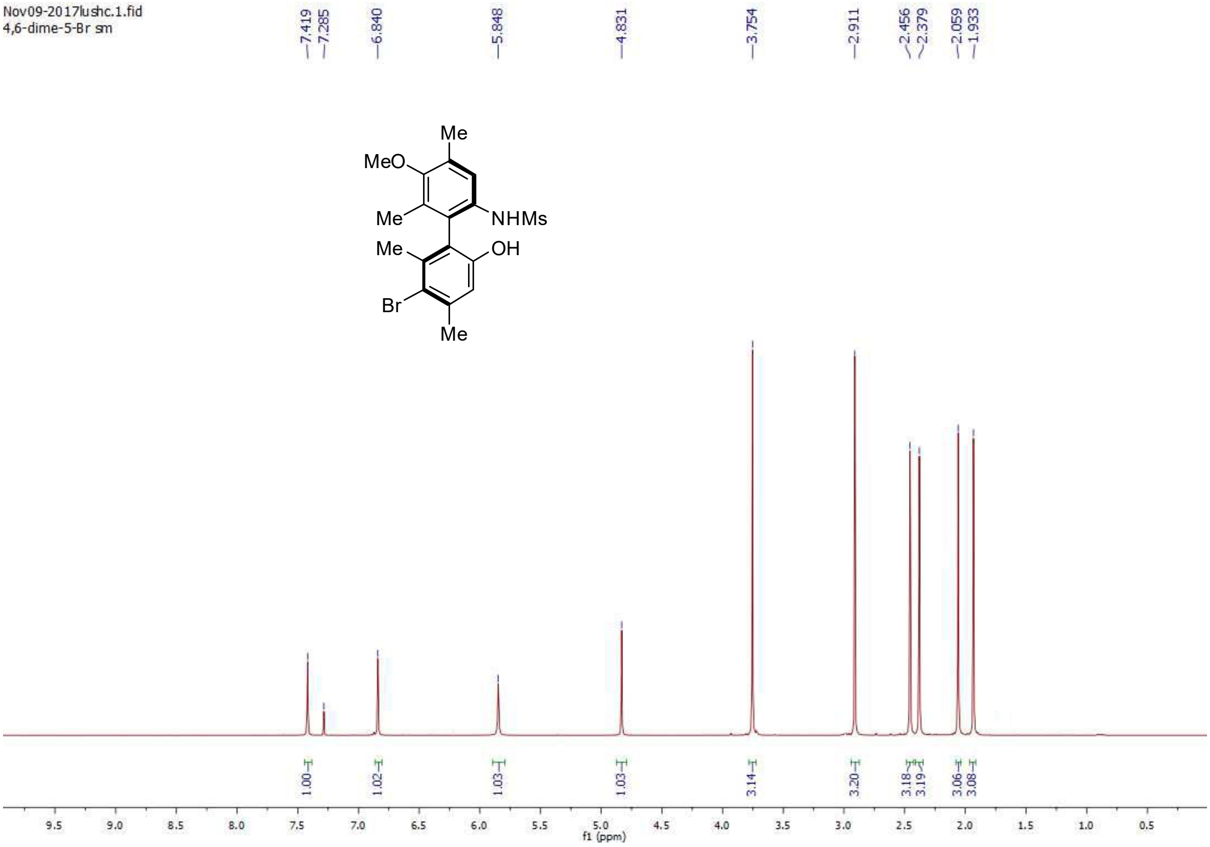

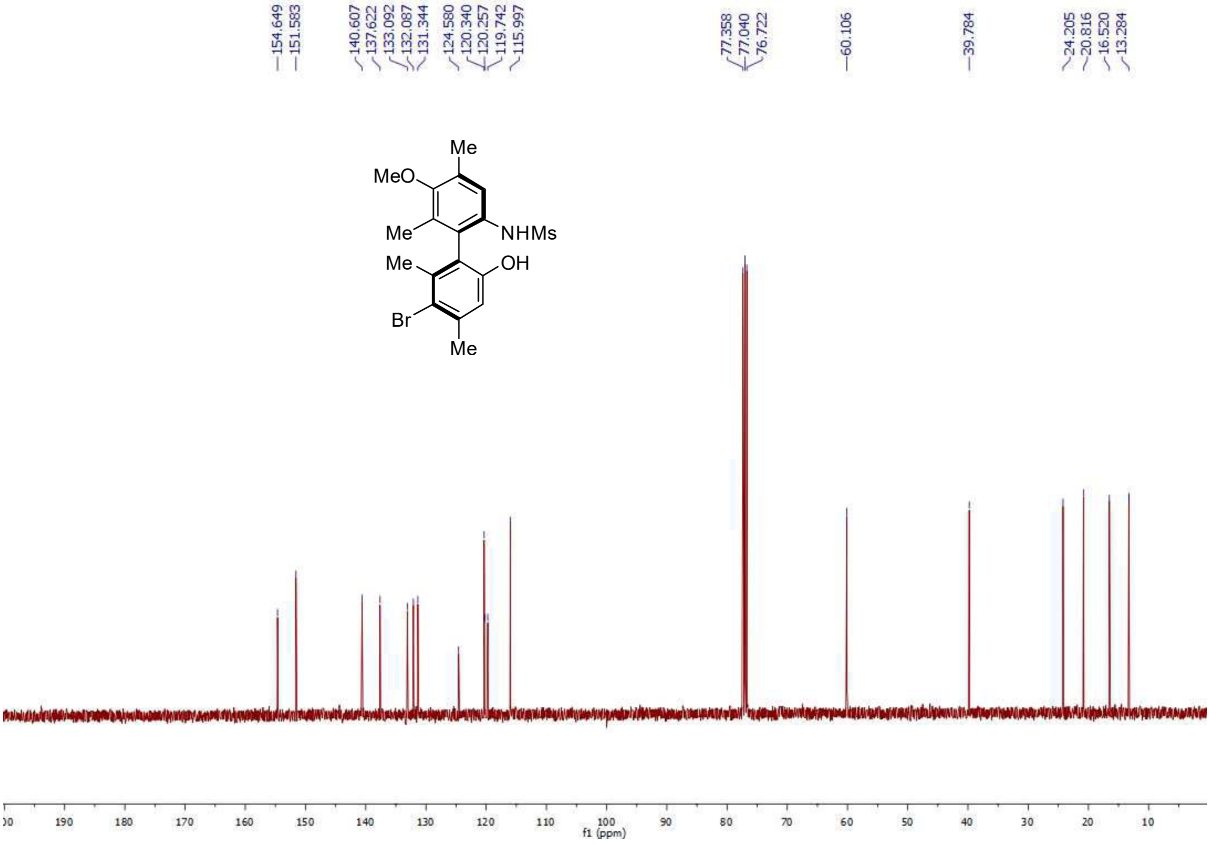

Supplementary Figure 80. ^{1}H and ^{13}C NMR Spectra of **1l**.

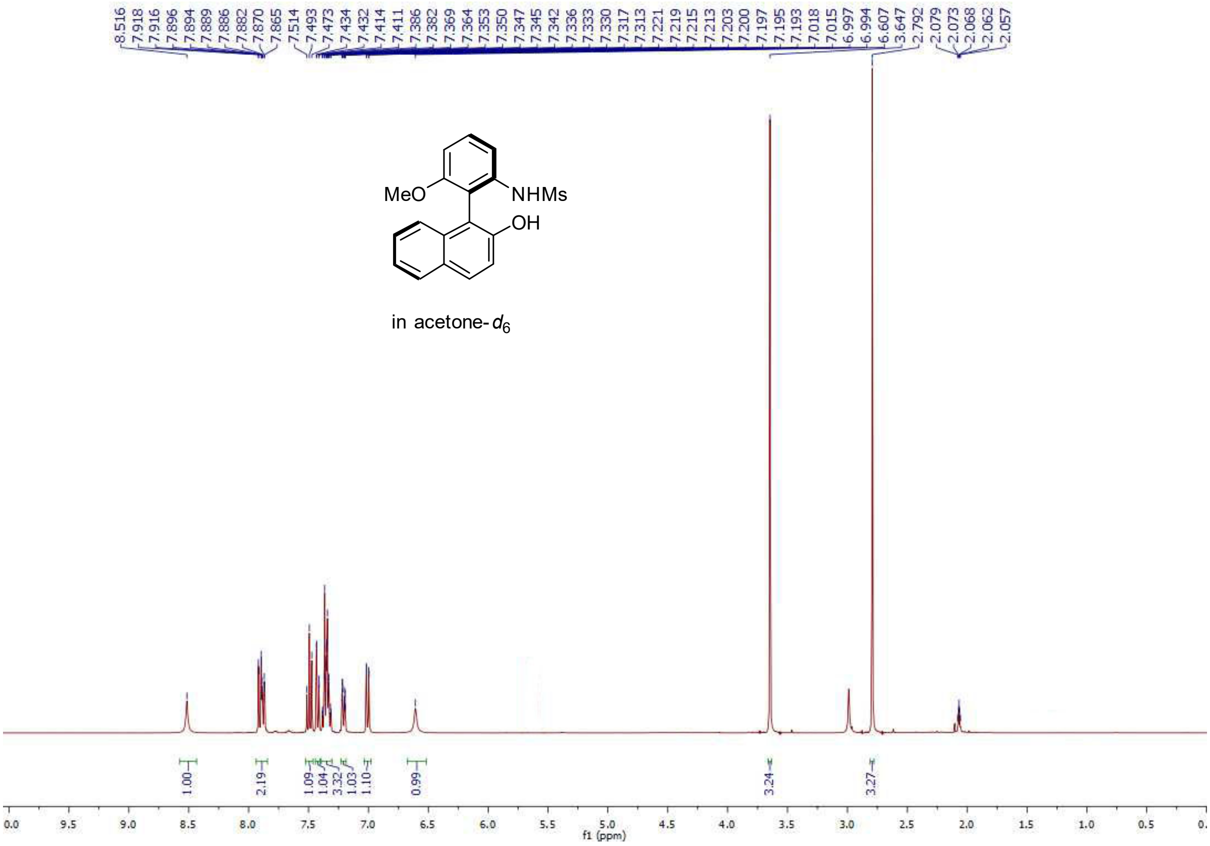

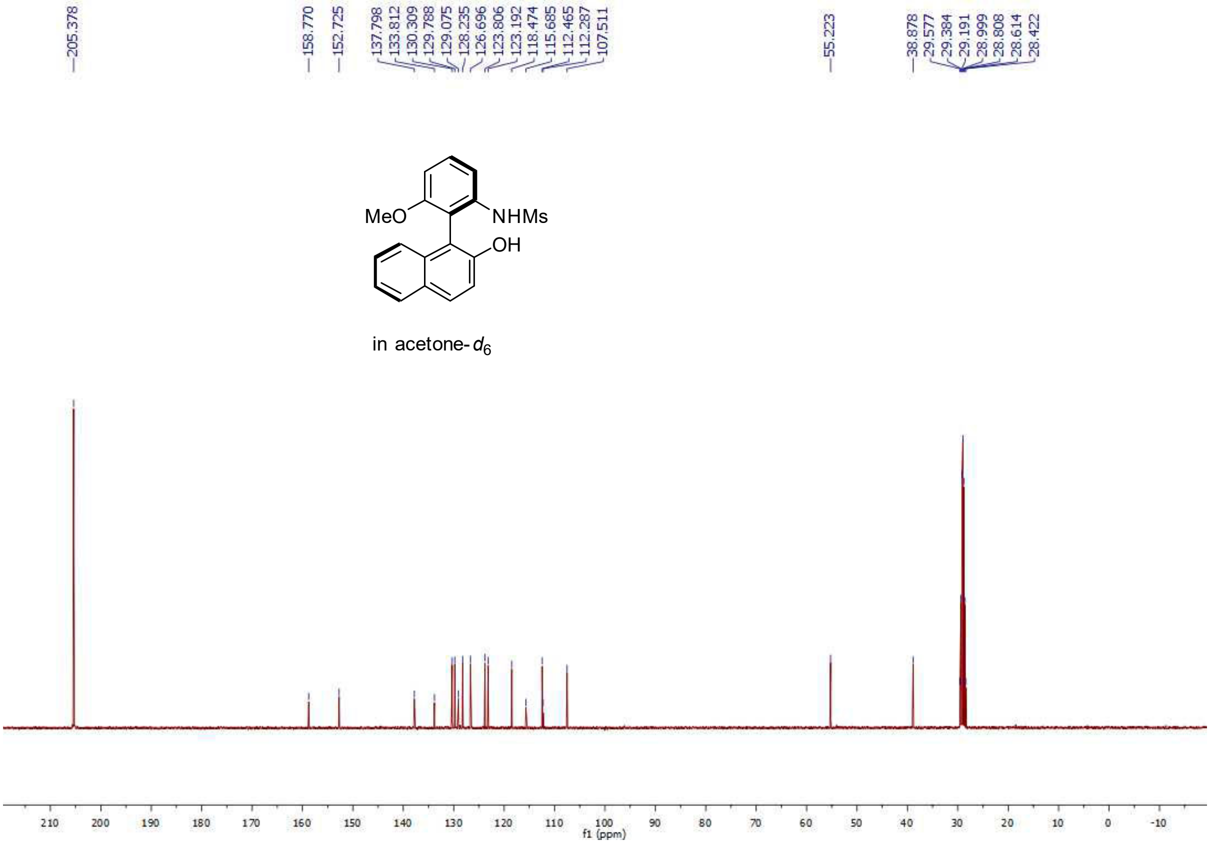

Supplementary Figure 81. ^{1}H and ^{13}C NMR Spectra of **1m**.

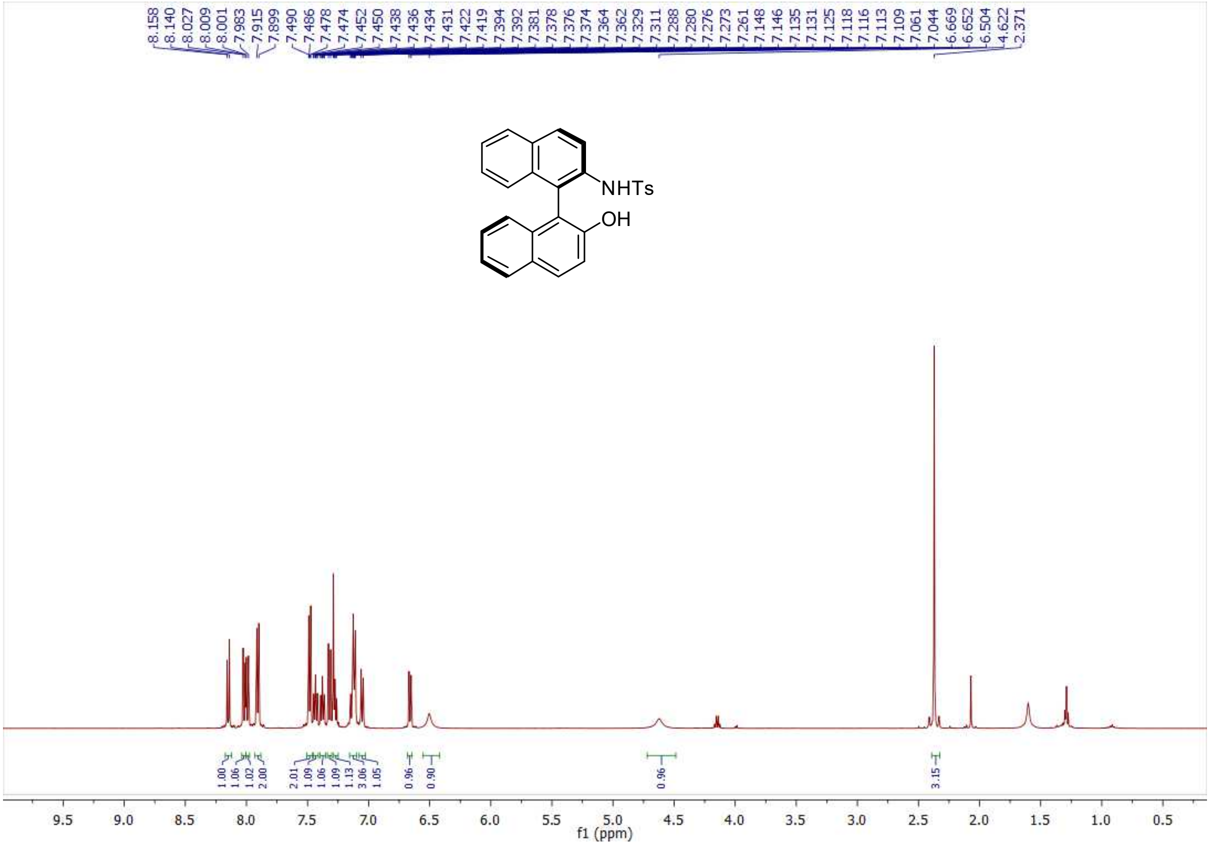

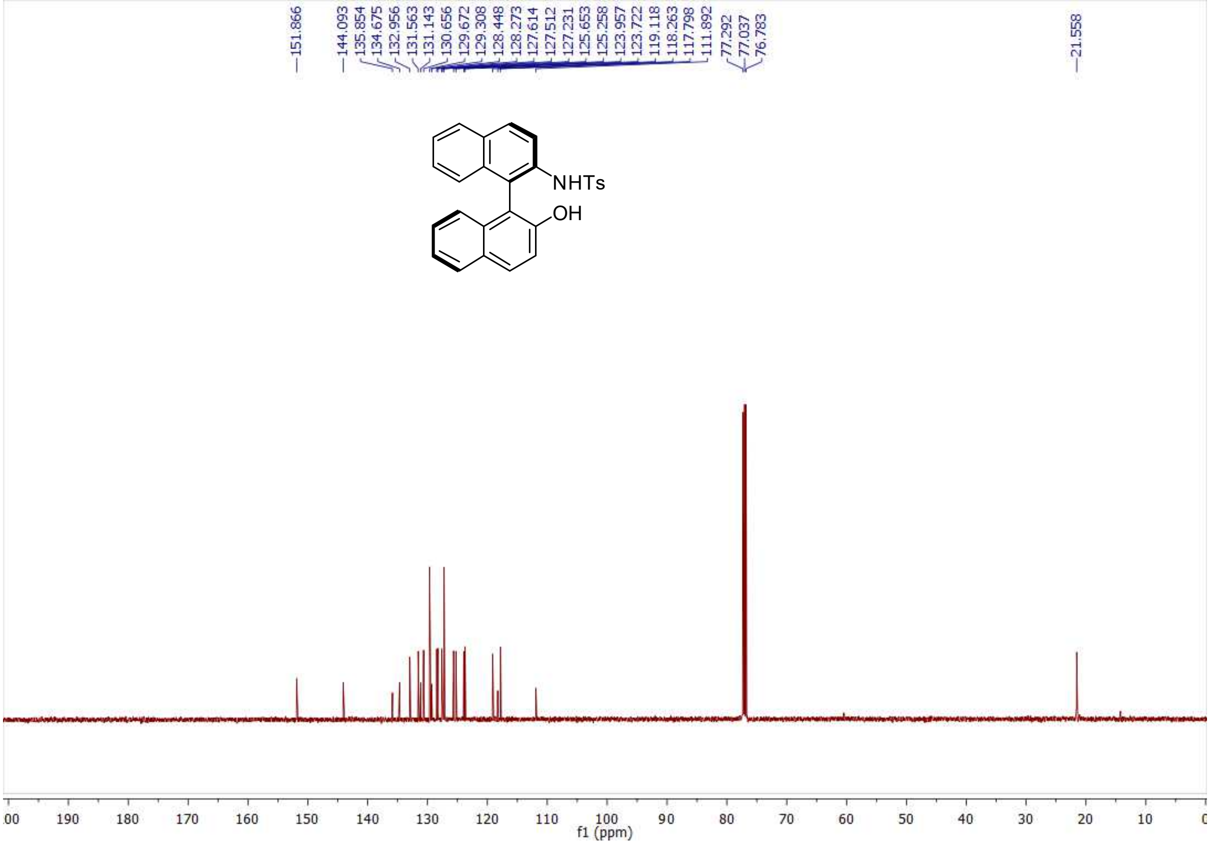

Supplementary Figure 82. ^{1}H and ^{13}C NMR Spectra of **1n**.

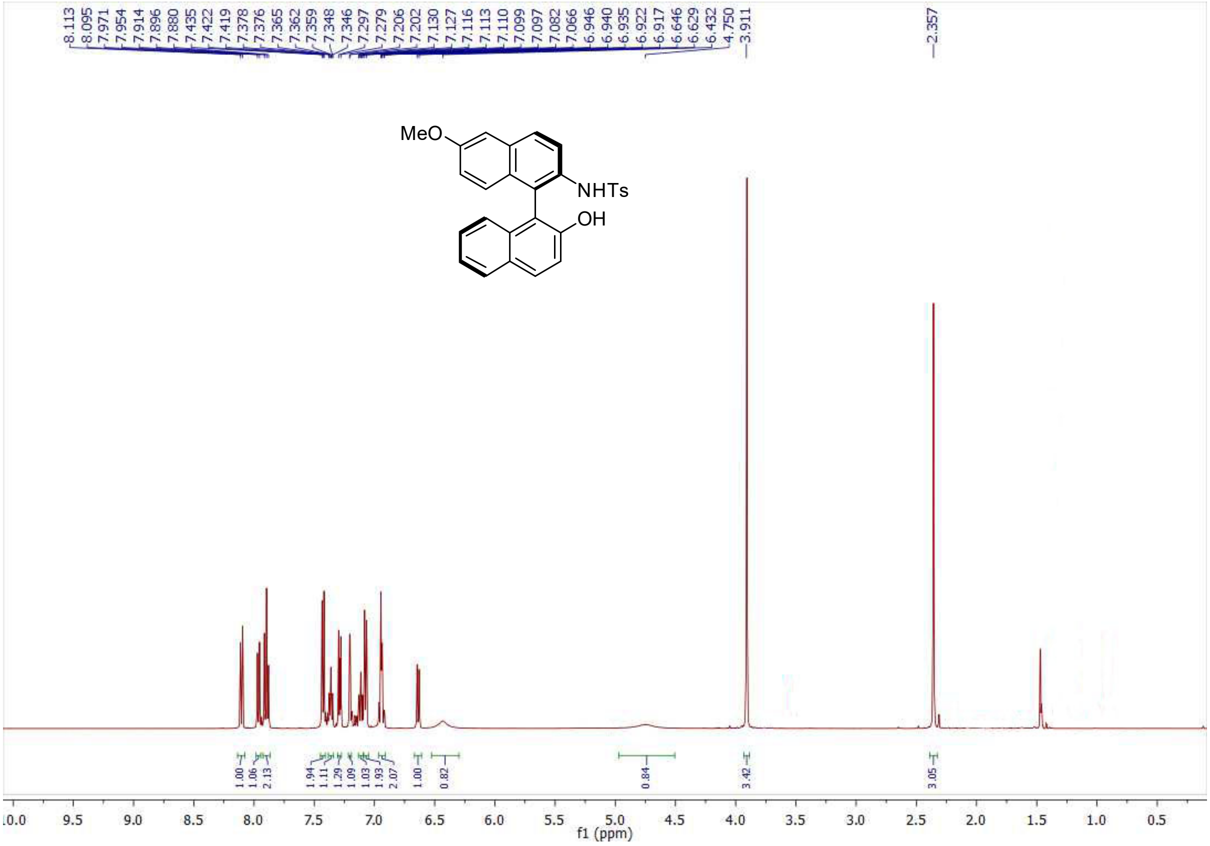

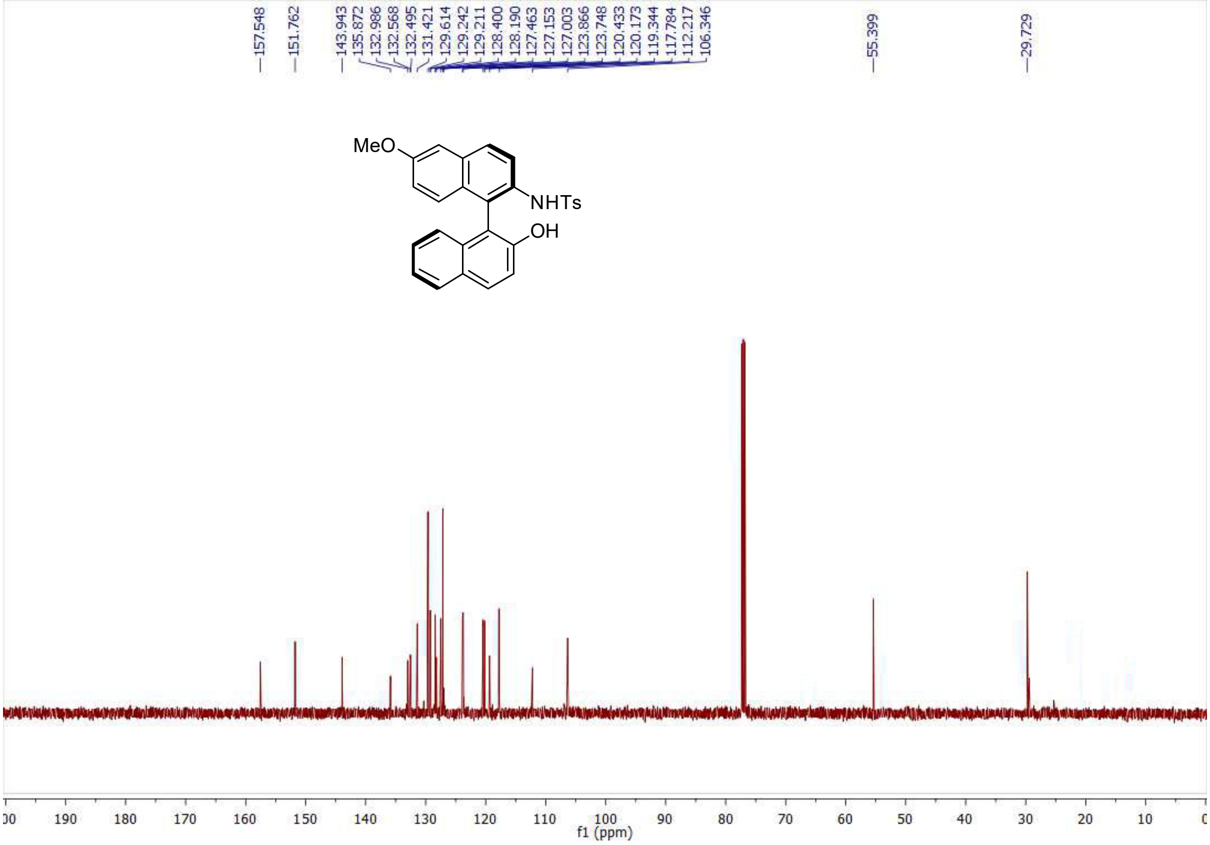

Supplementary Figure 83. ^{1}H and ^{13}C NMR Spectra of **1o**.

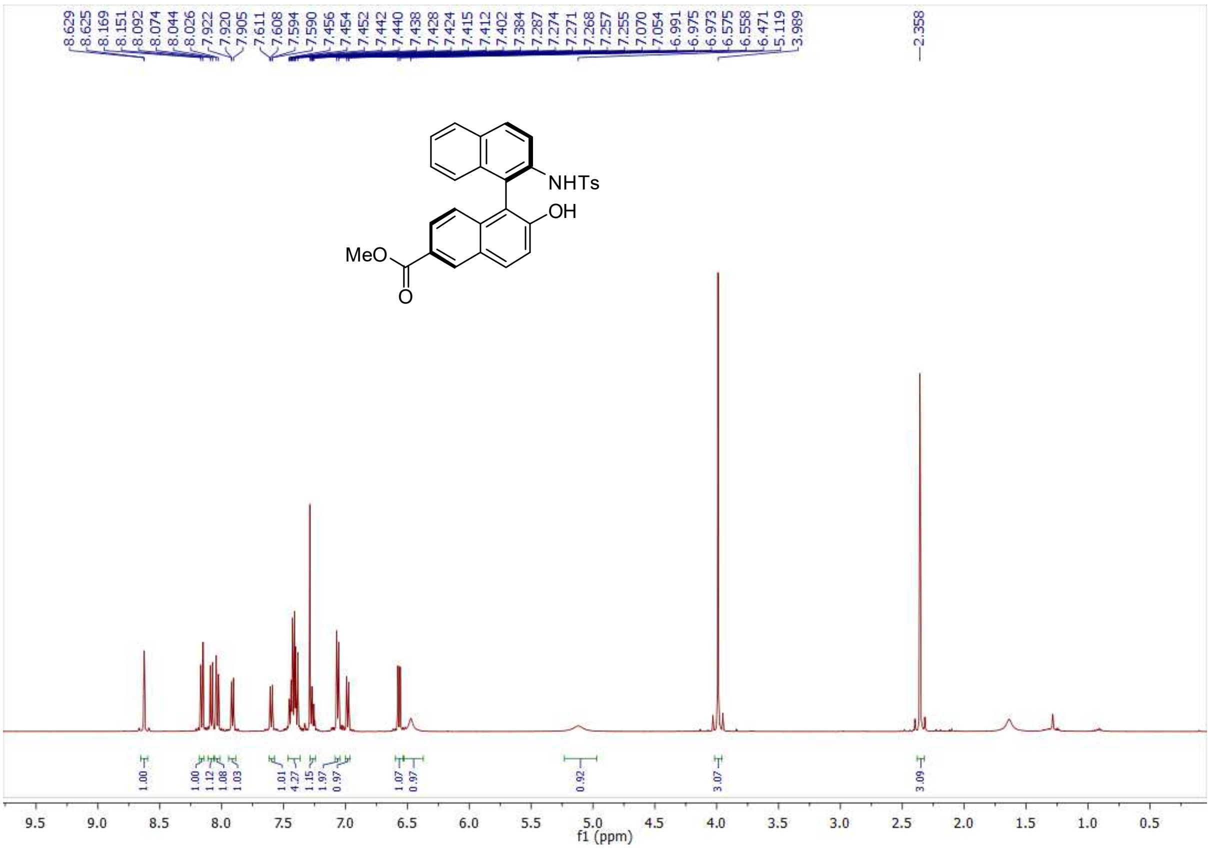

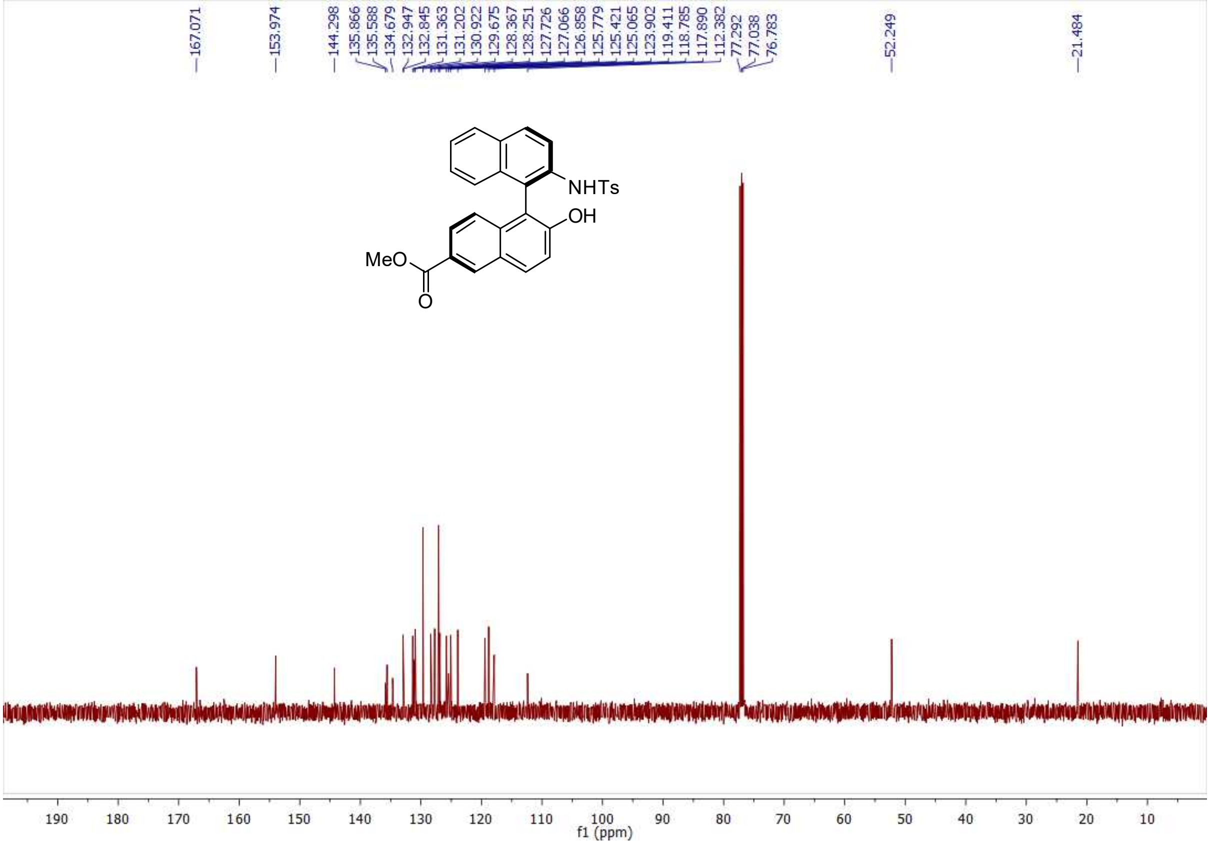

Supplementary Figure 84. ^{1}H and ^{13}C NMR Spectra of **1p**.

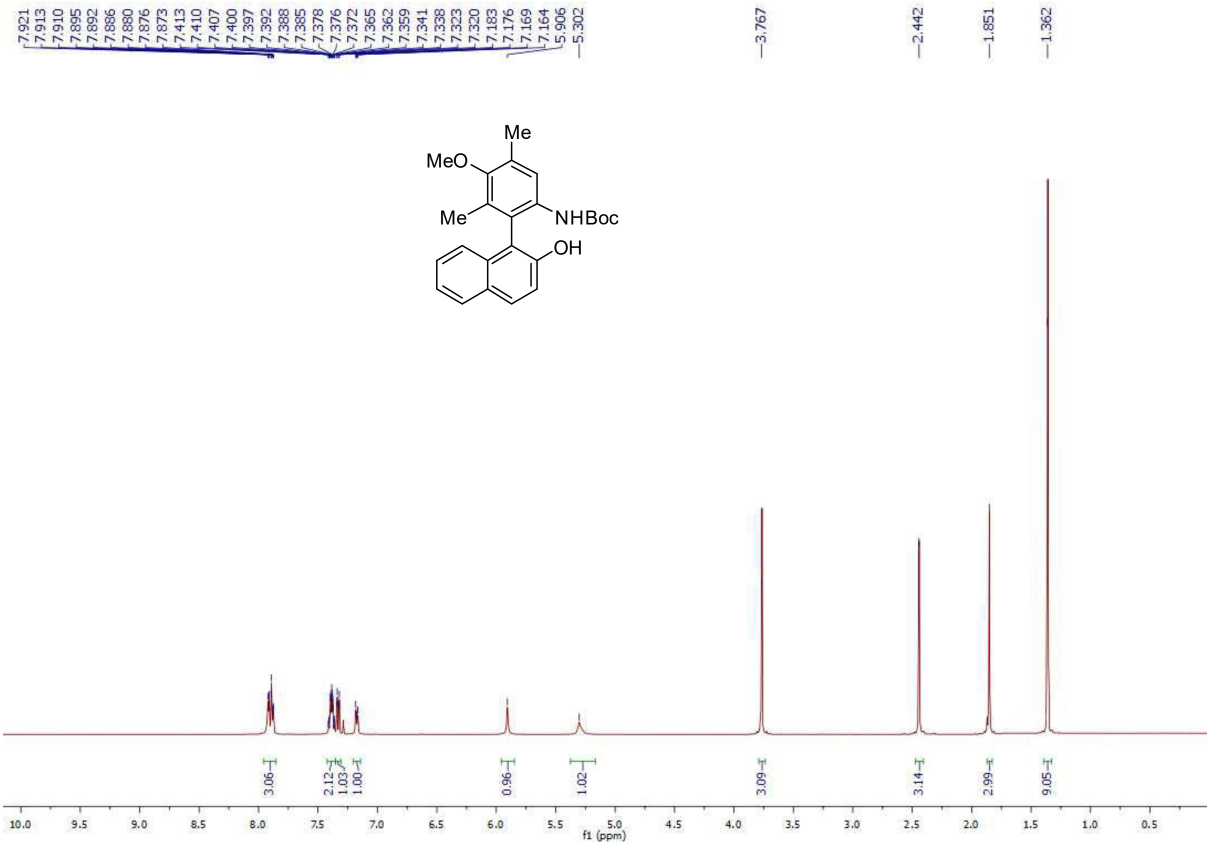

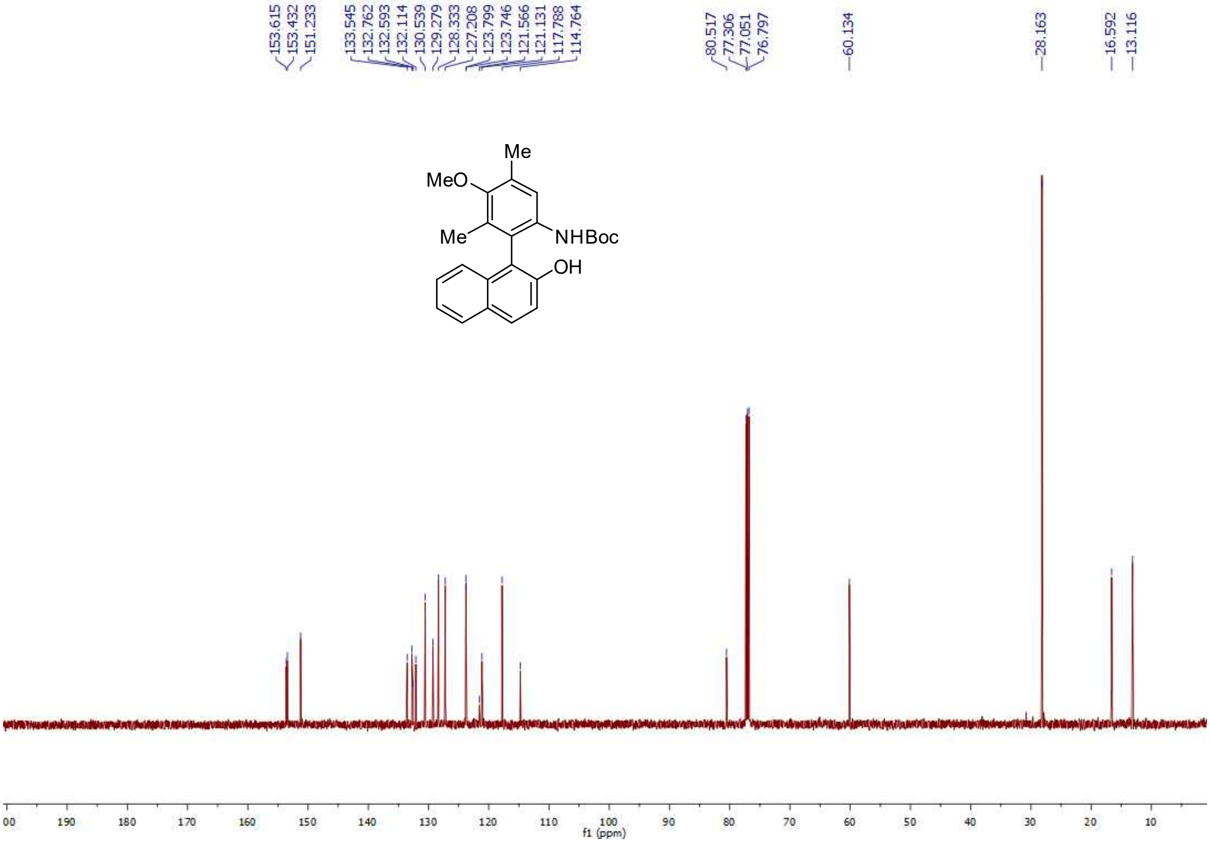

Supplementary Figure 85. ^{1}H and ^{13}C NMR Spectra of **1q**.

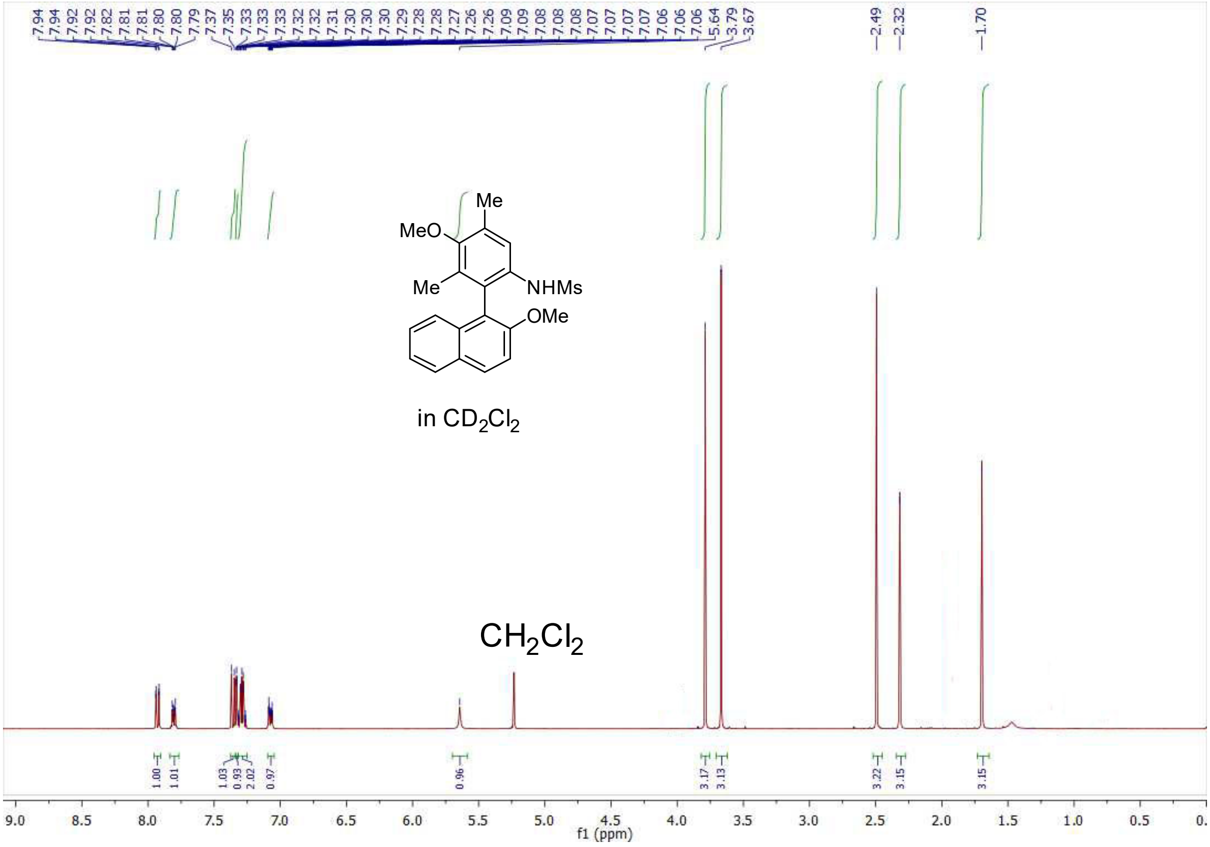

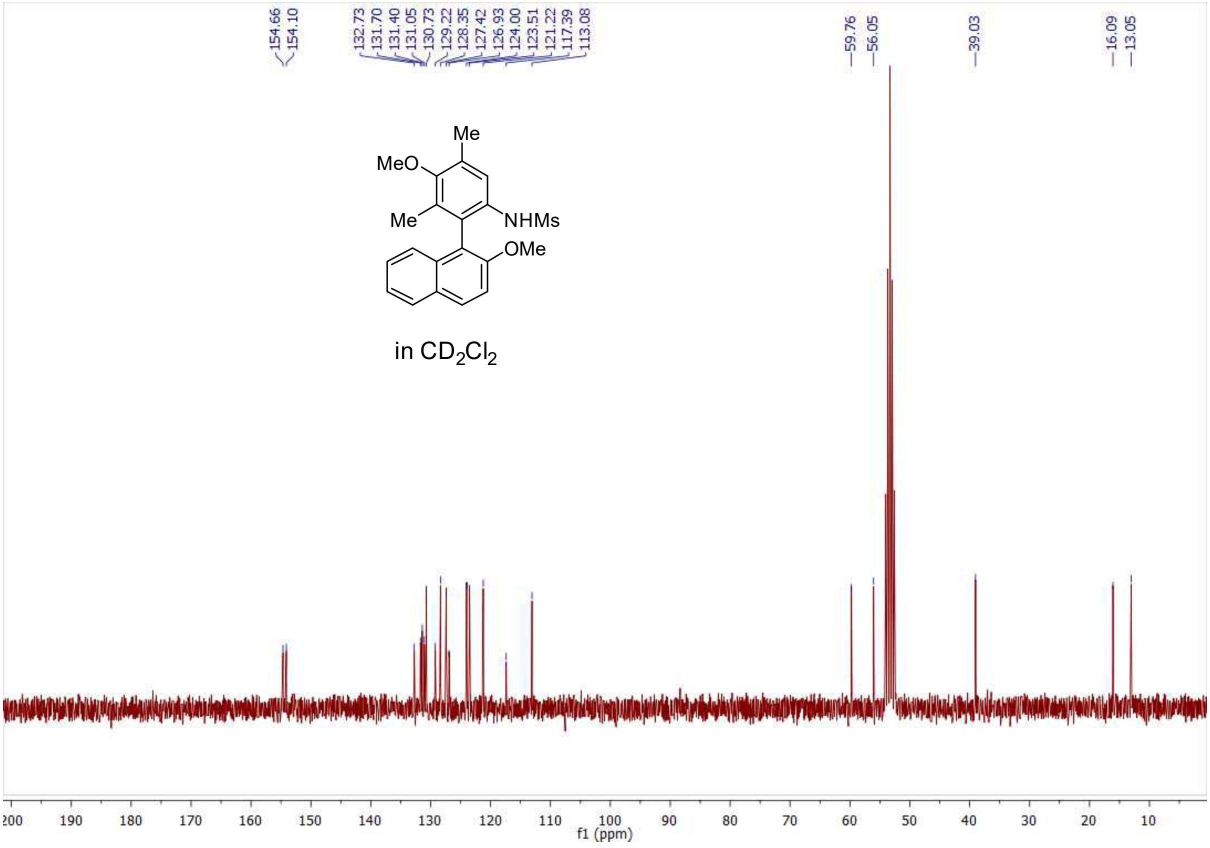

Supplementary Figure 86. ^{1}H and ^{13}C NMR Spectra of **3a**.

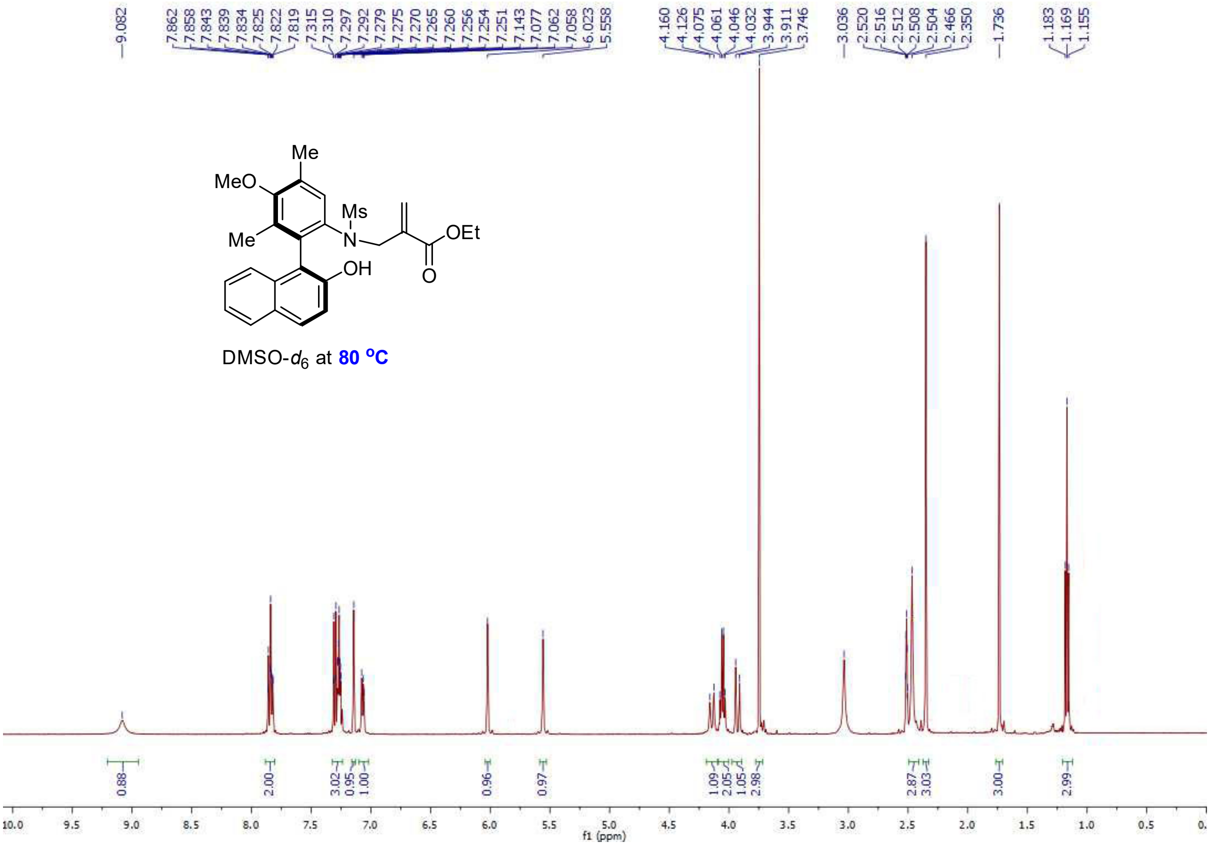

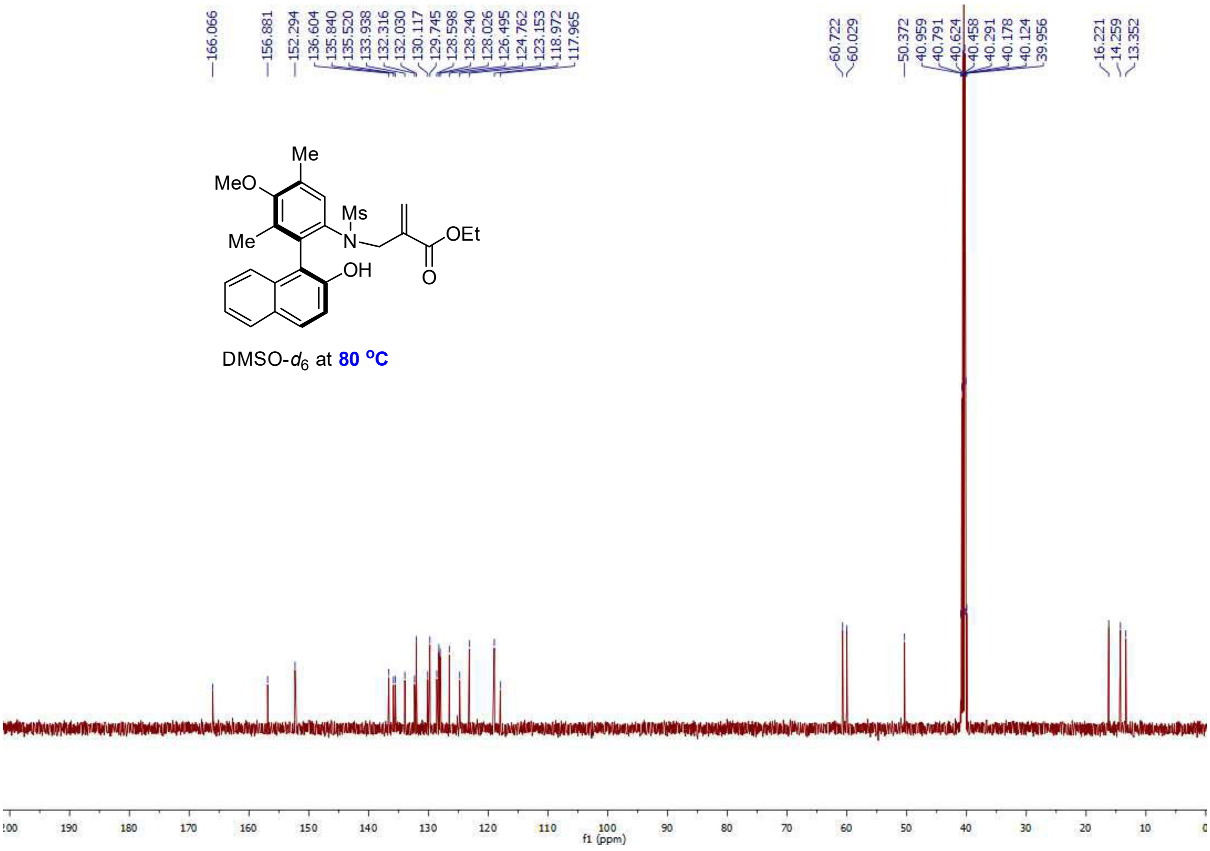

Supplementary Figure 87. ^{1}H and ^{13}C NMR Spectra of **4a**.

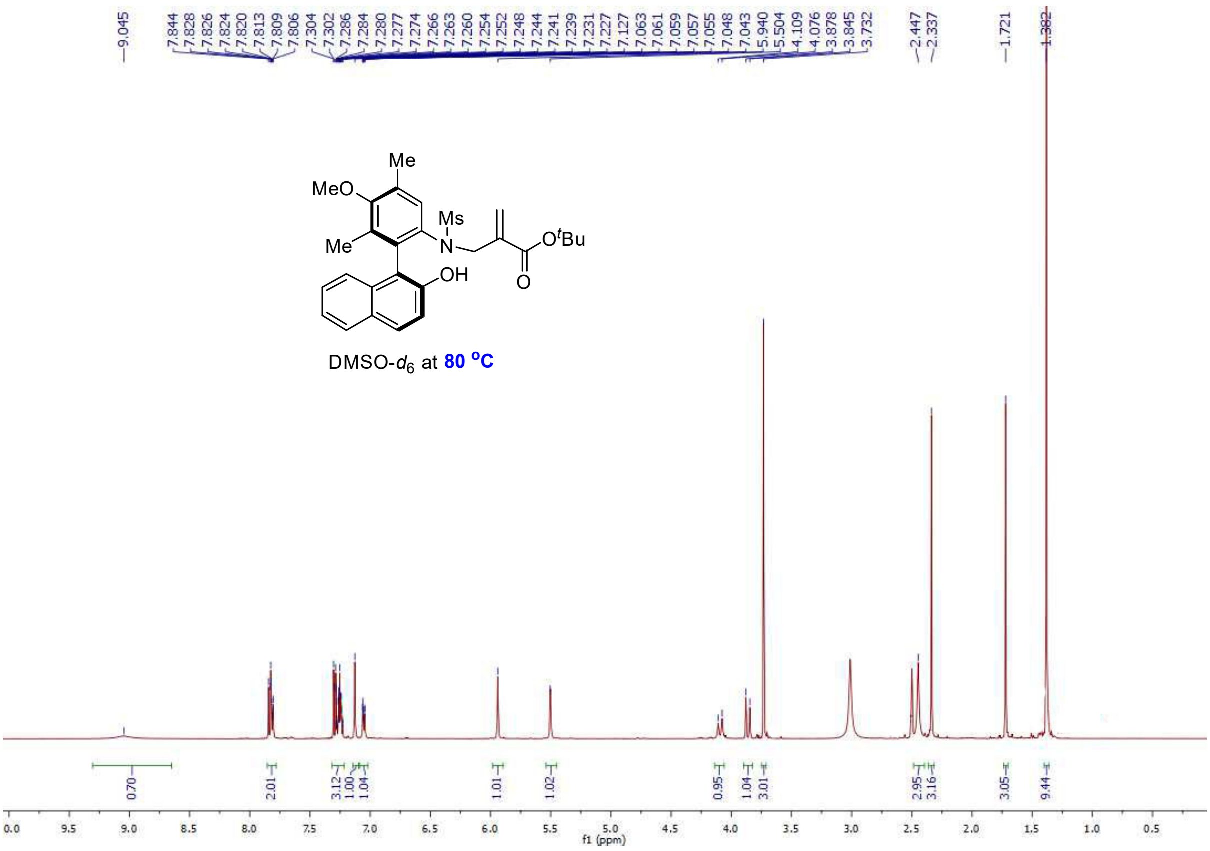

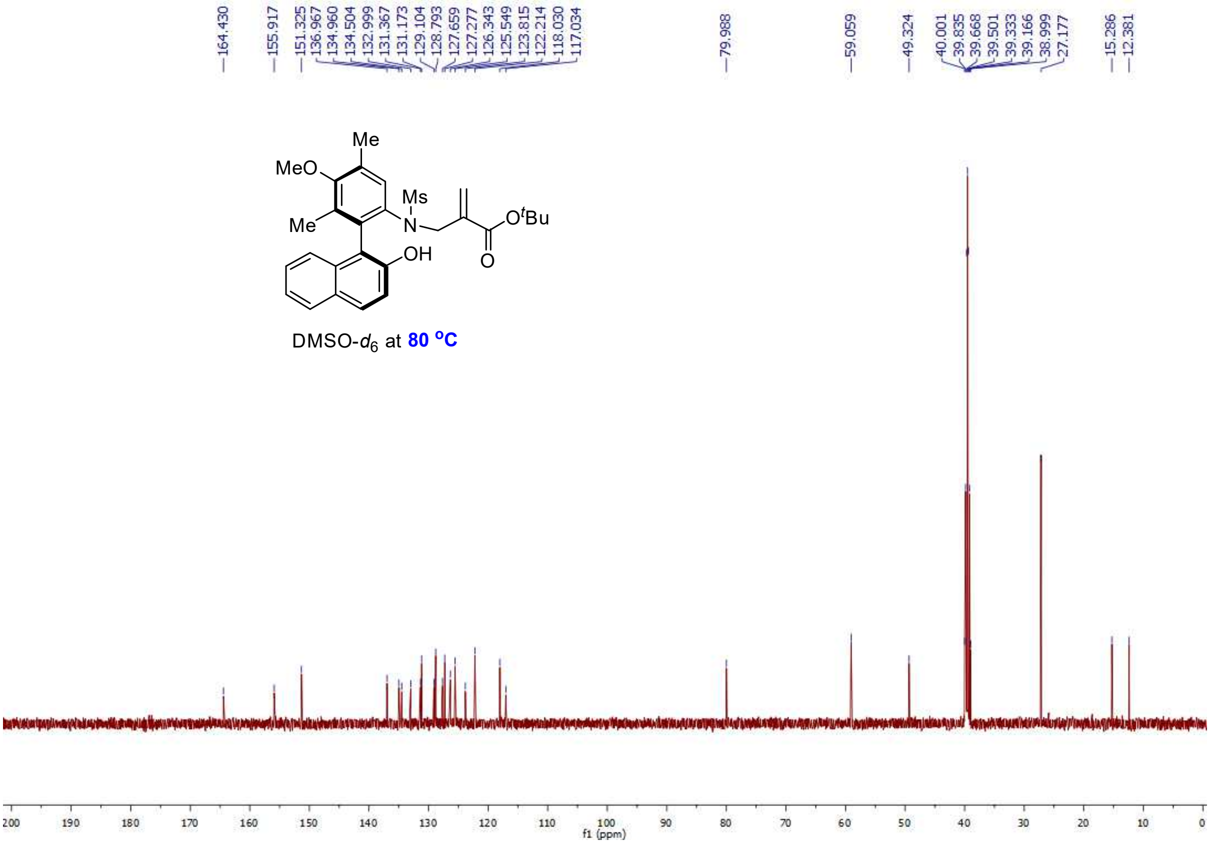

Supplementary Figure 88. ^{1}H and ^{13}C NMR Spectra of **4b**.

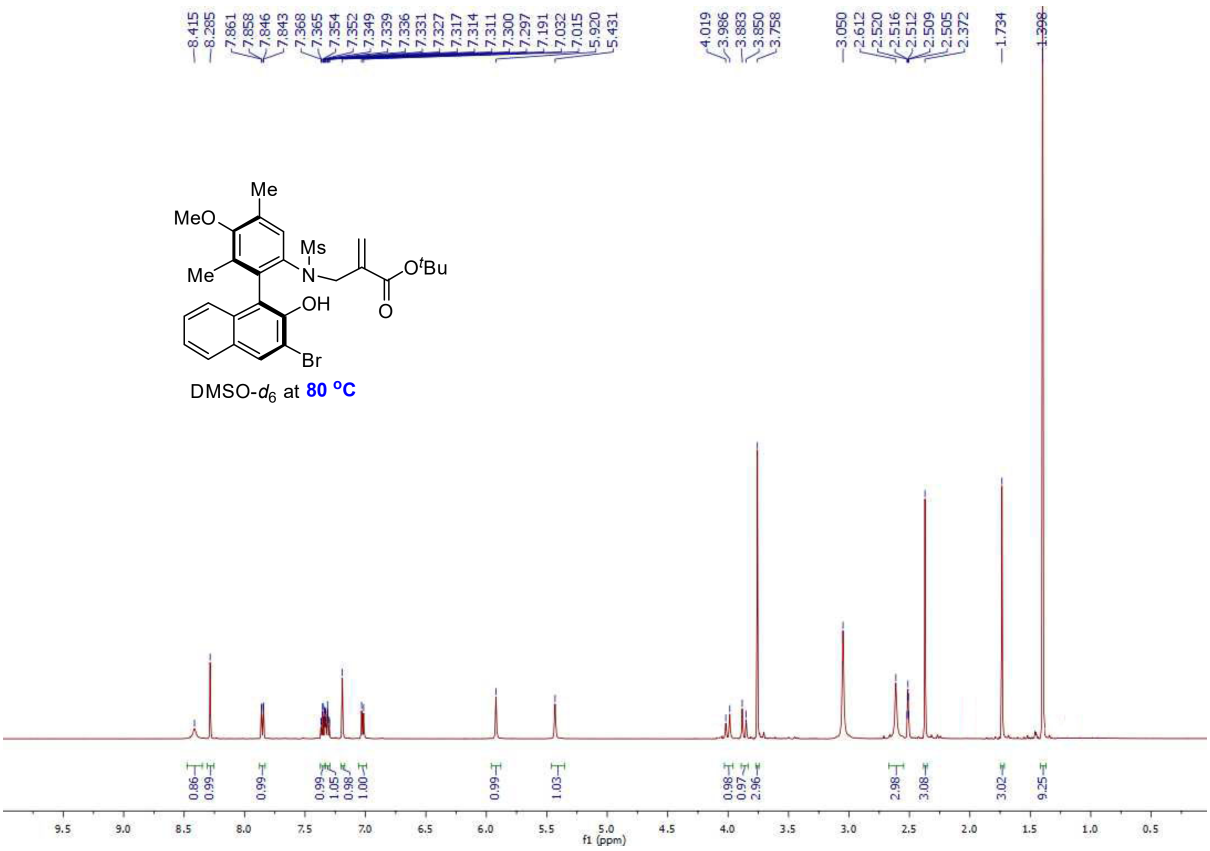

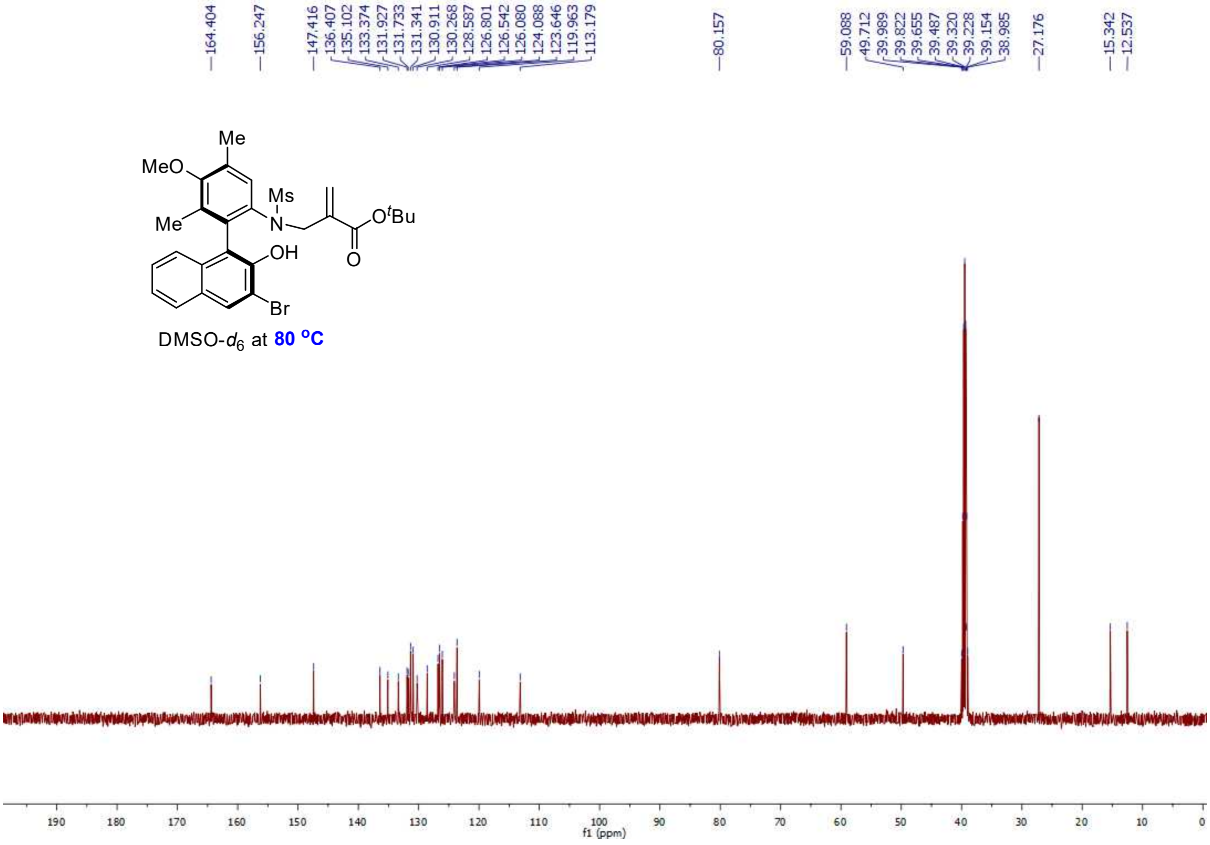

Supplementary Figure 89. ^{1}H and ^{13}C NMR Spectra of **4c**.

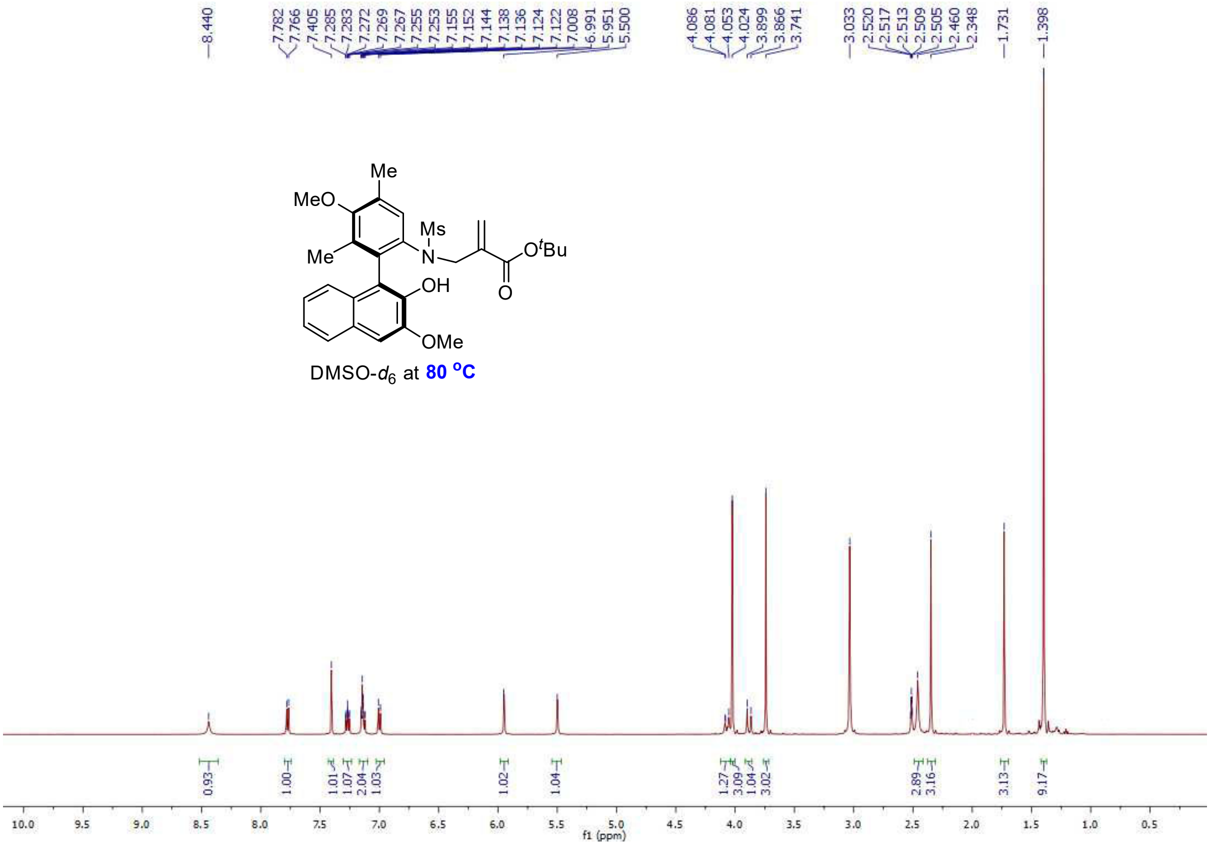

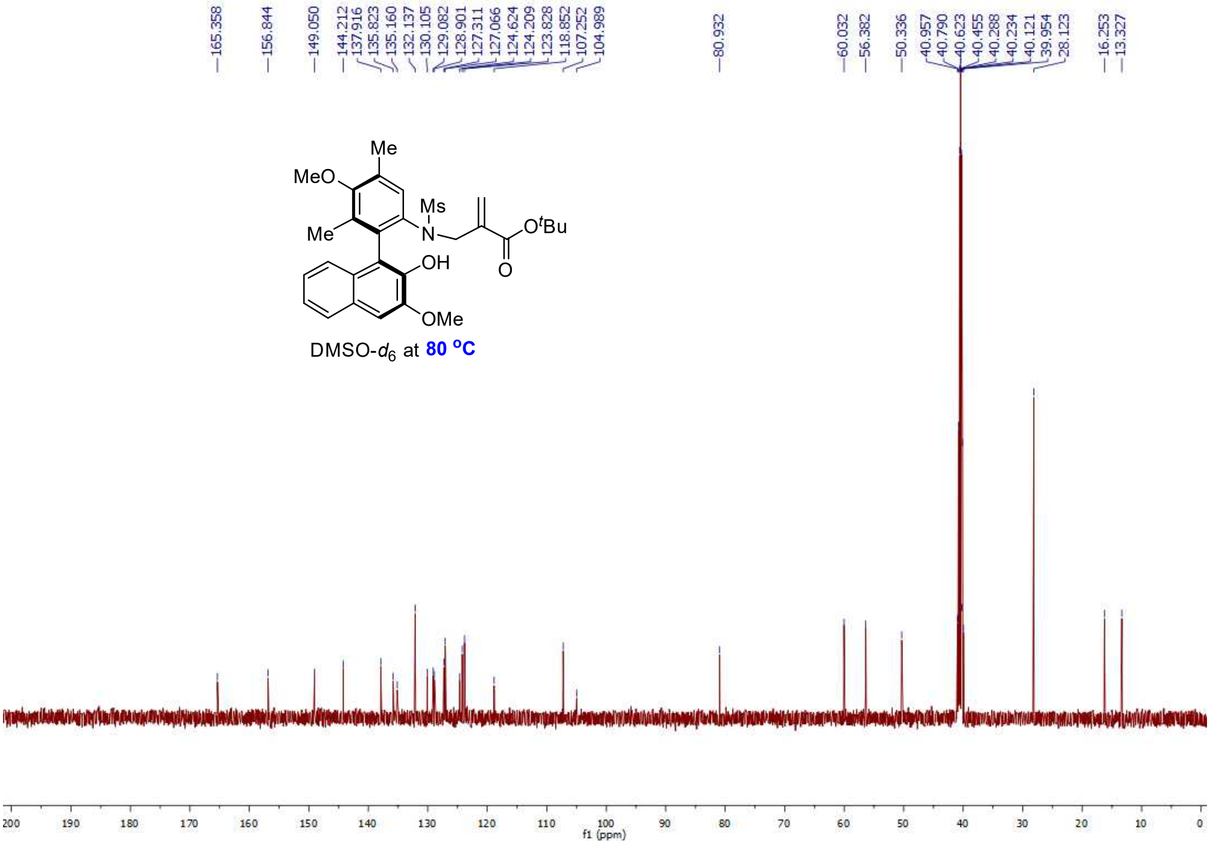

Supplementary Figure 90. ^{1}H and ^{13}C NMR Spectra of **4d**.

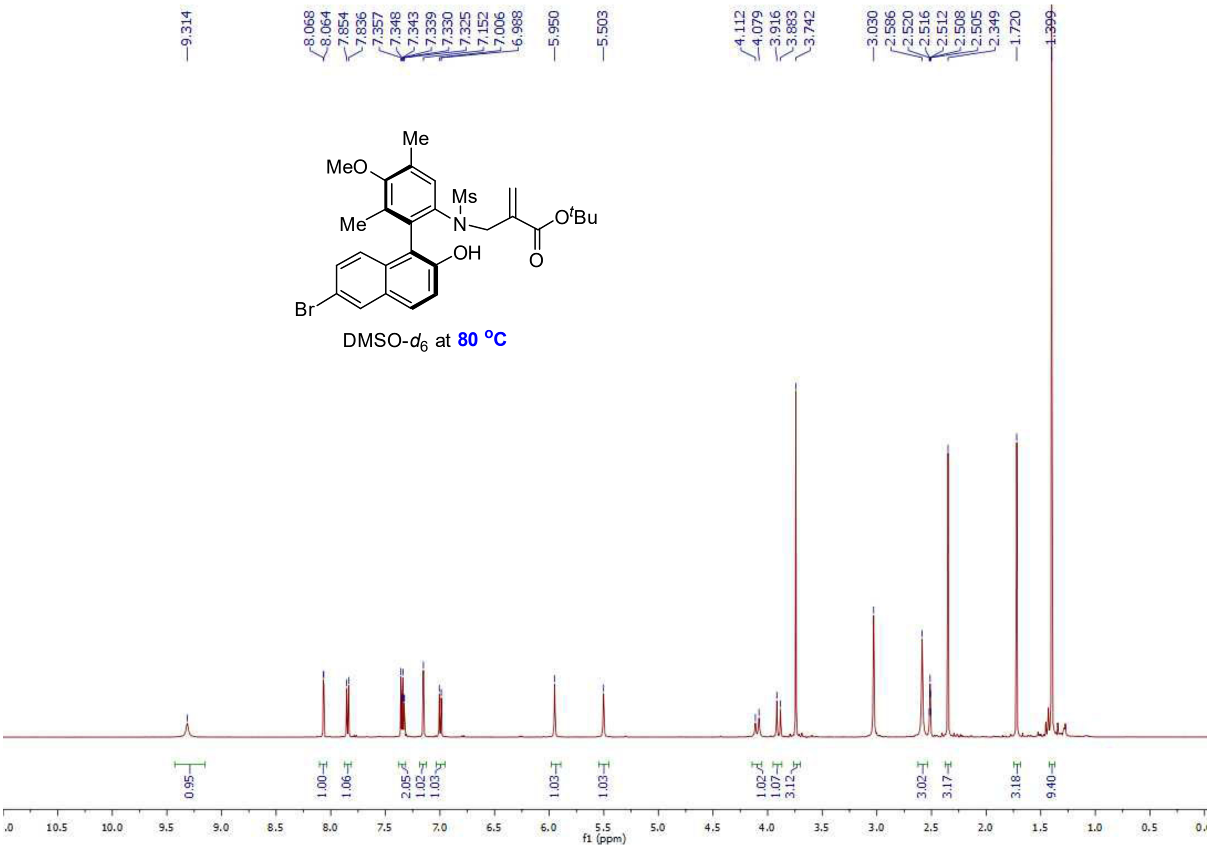

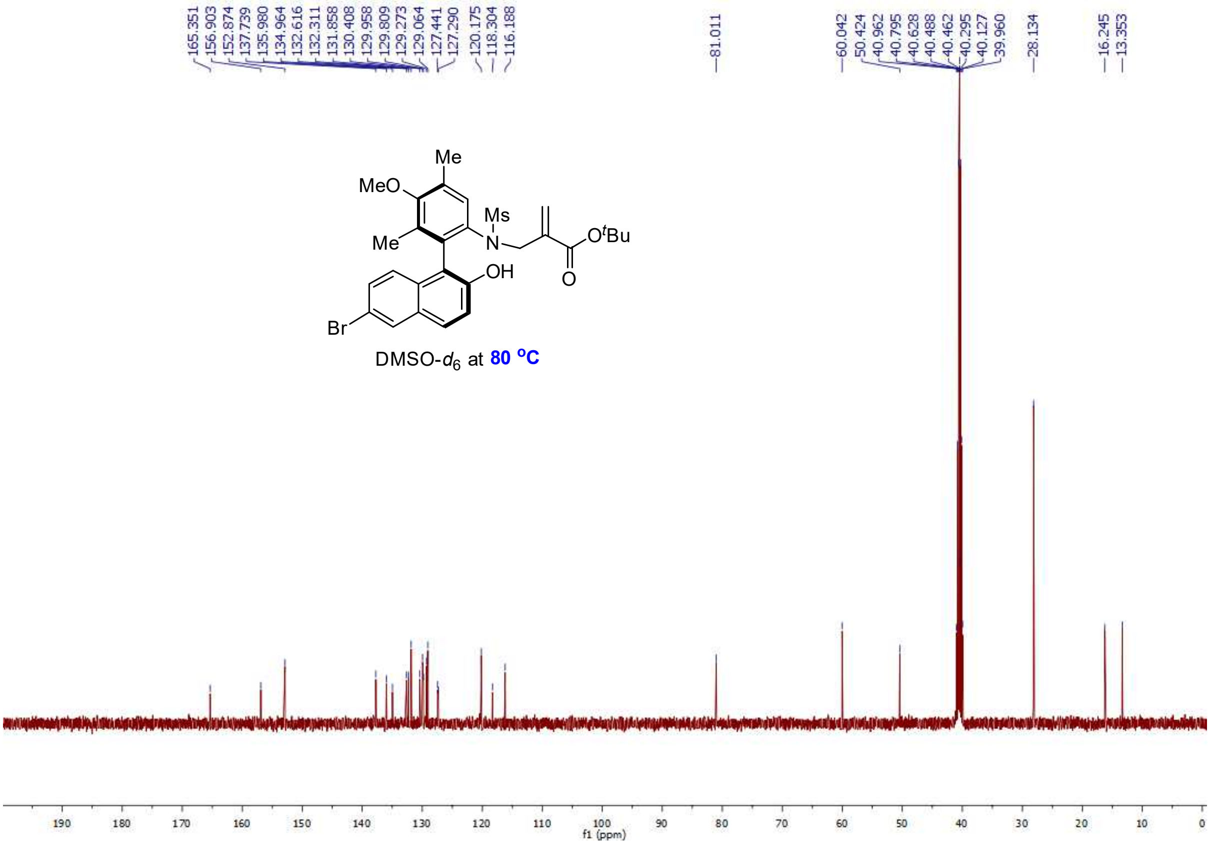

Supplementary Figure 91. ^{1}H and ^{13}C NMR Spectra of **4e**.

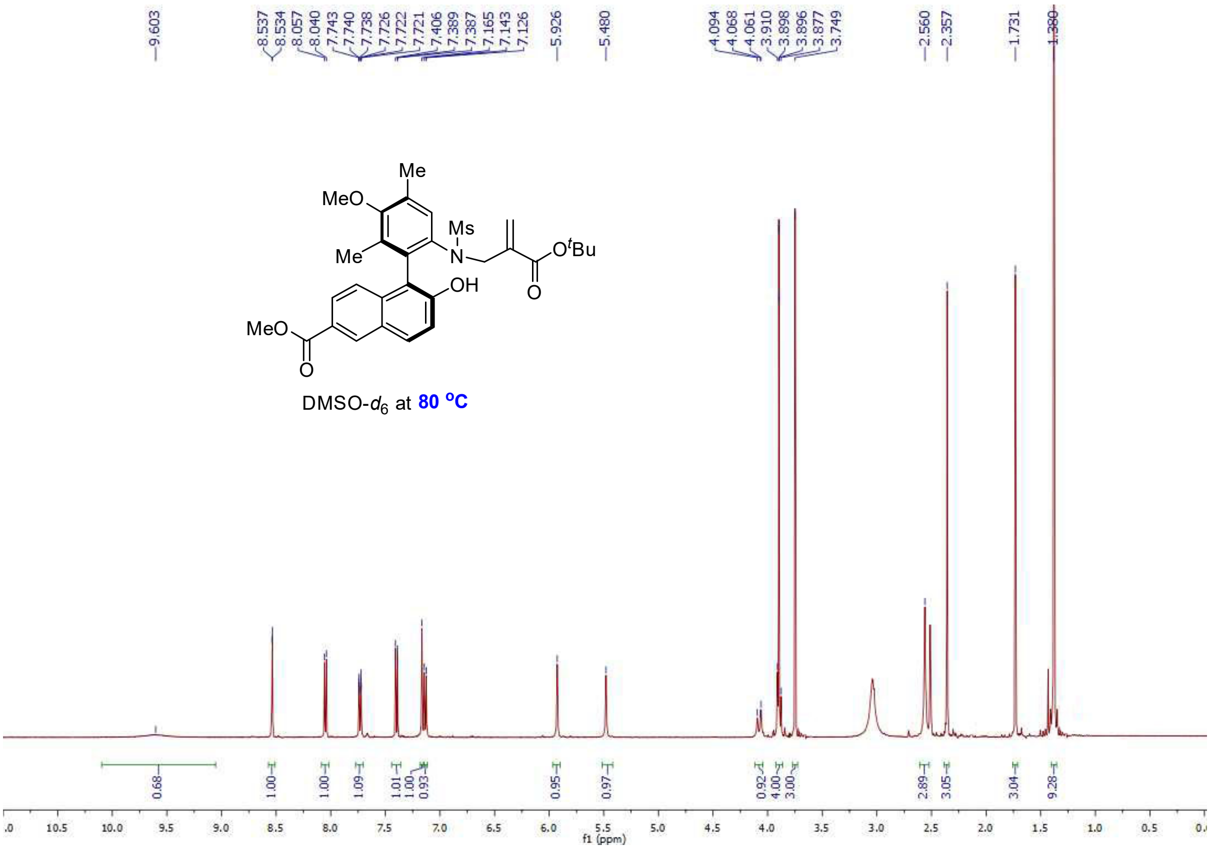

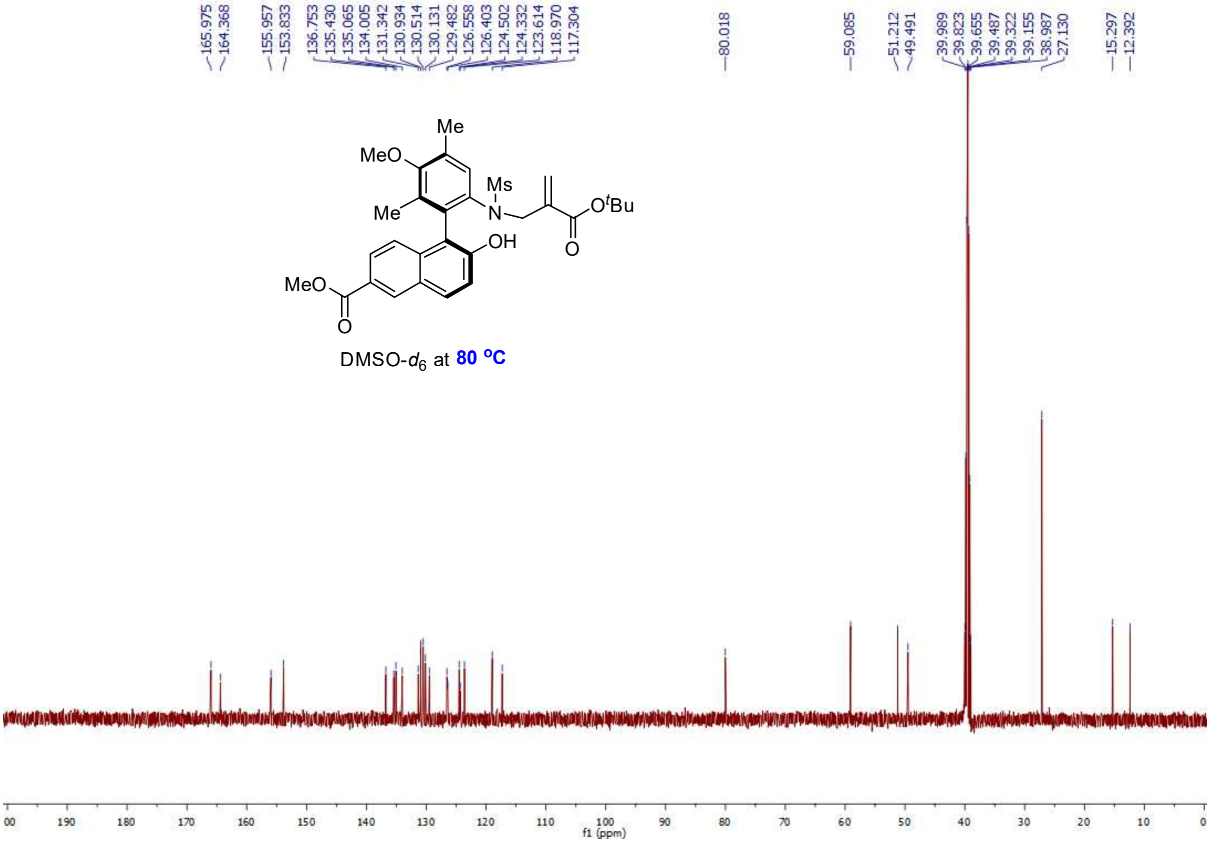

Supplementary Figure 92. ^{1}H and ^{13}C NMR Spectra of **4f**.

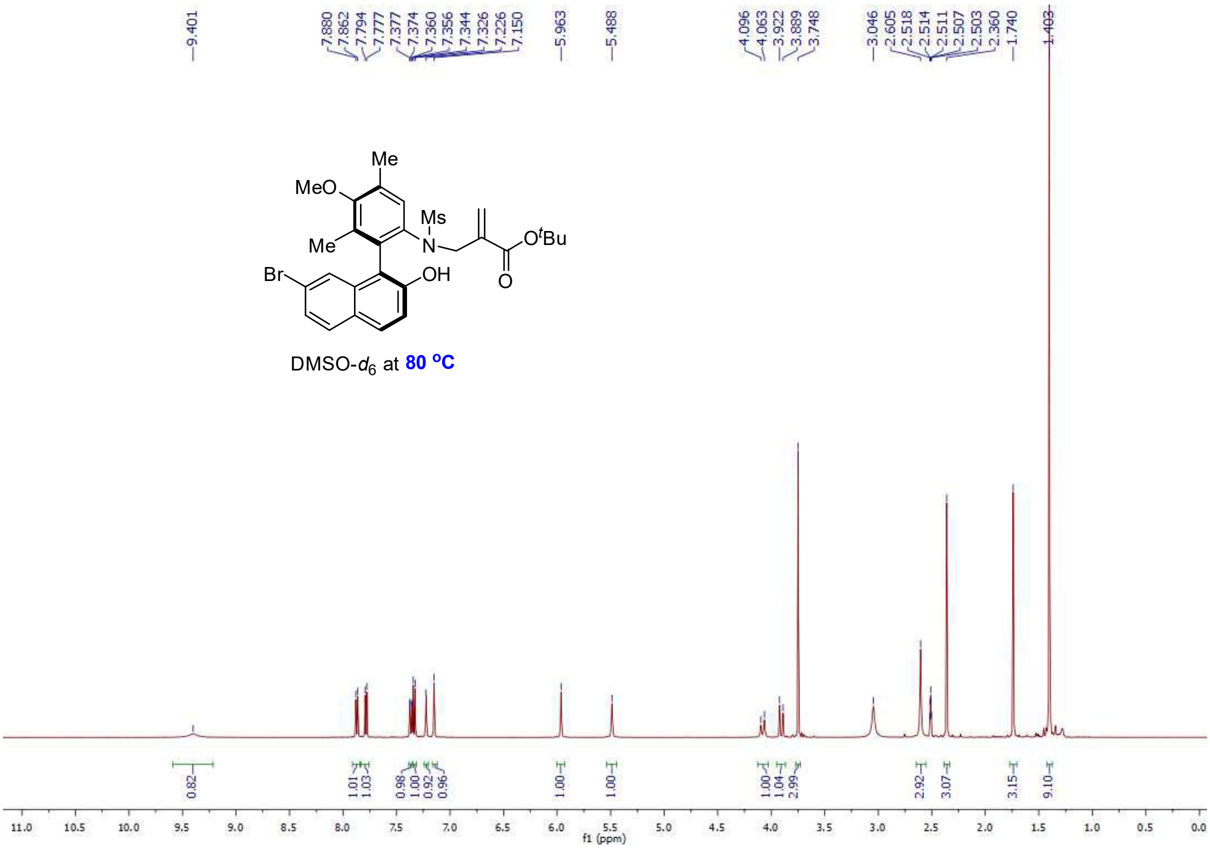

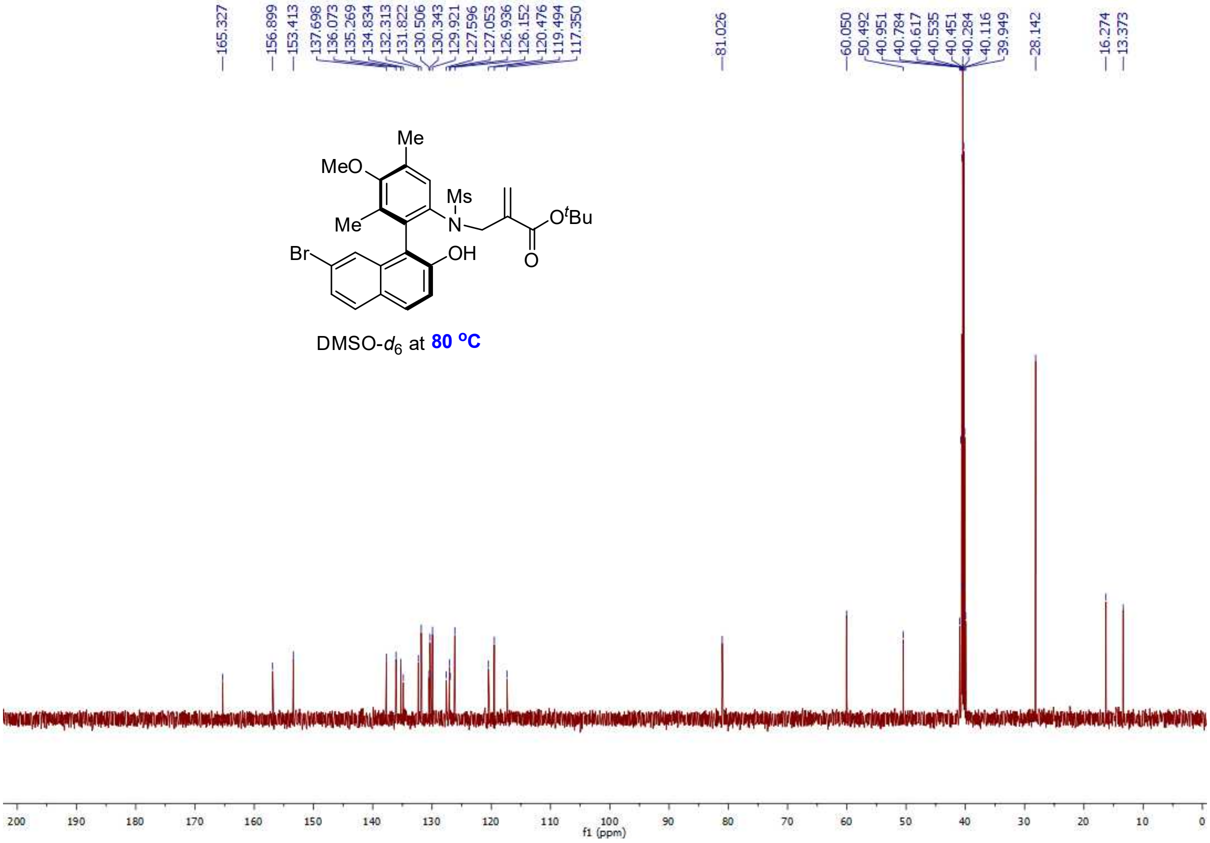

Supplementary Figure 93. ^{1}H and ^{13}C NMR Spectra of **4g**.

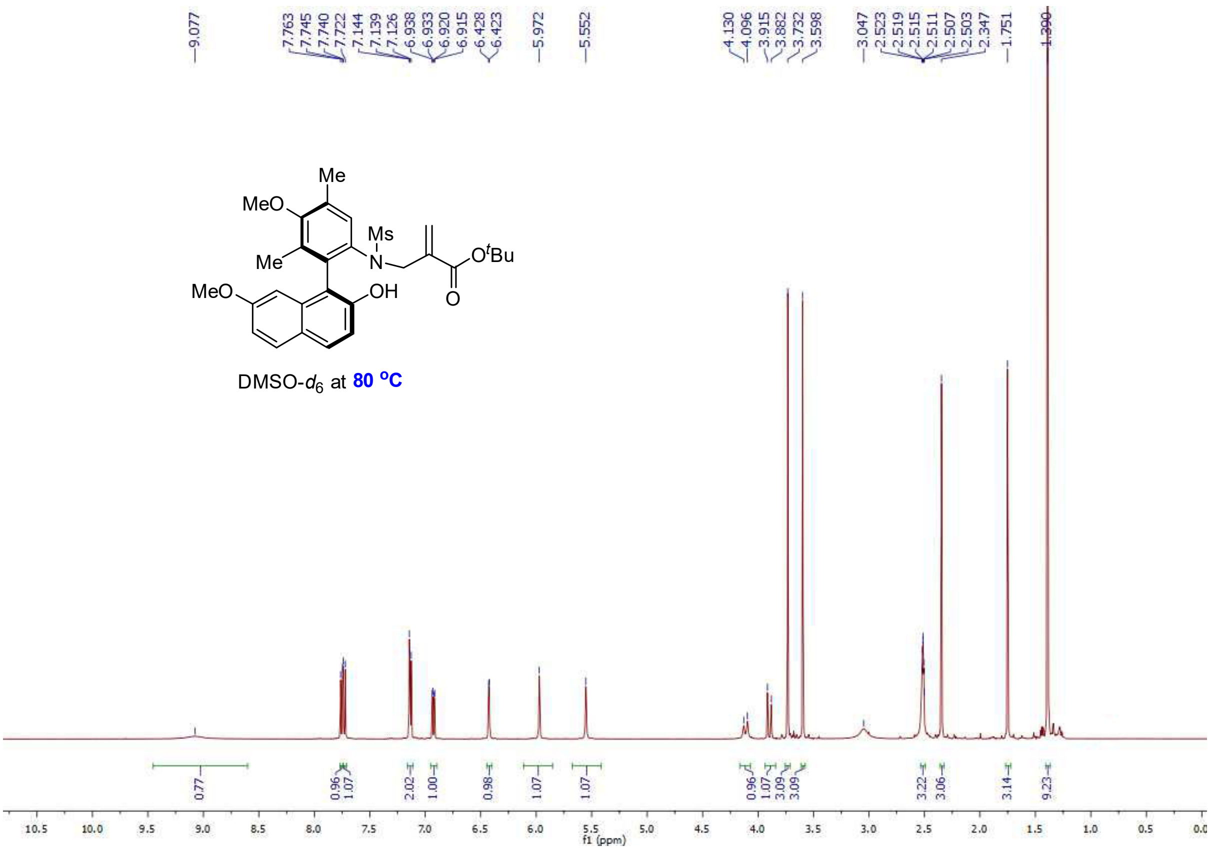

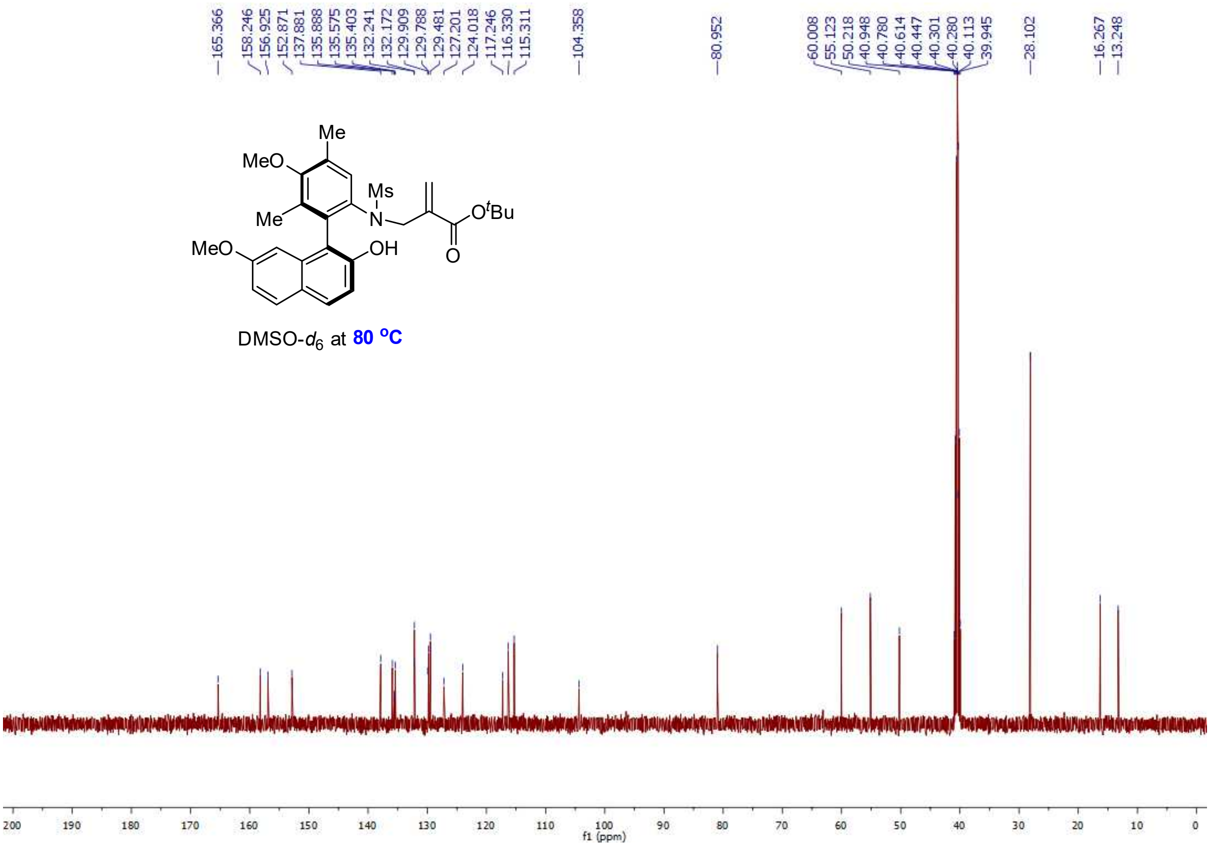

Supplementary Figure 94. ^{1}H and ^{13}C NMR Spectra of **4h**.

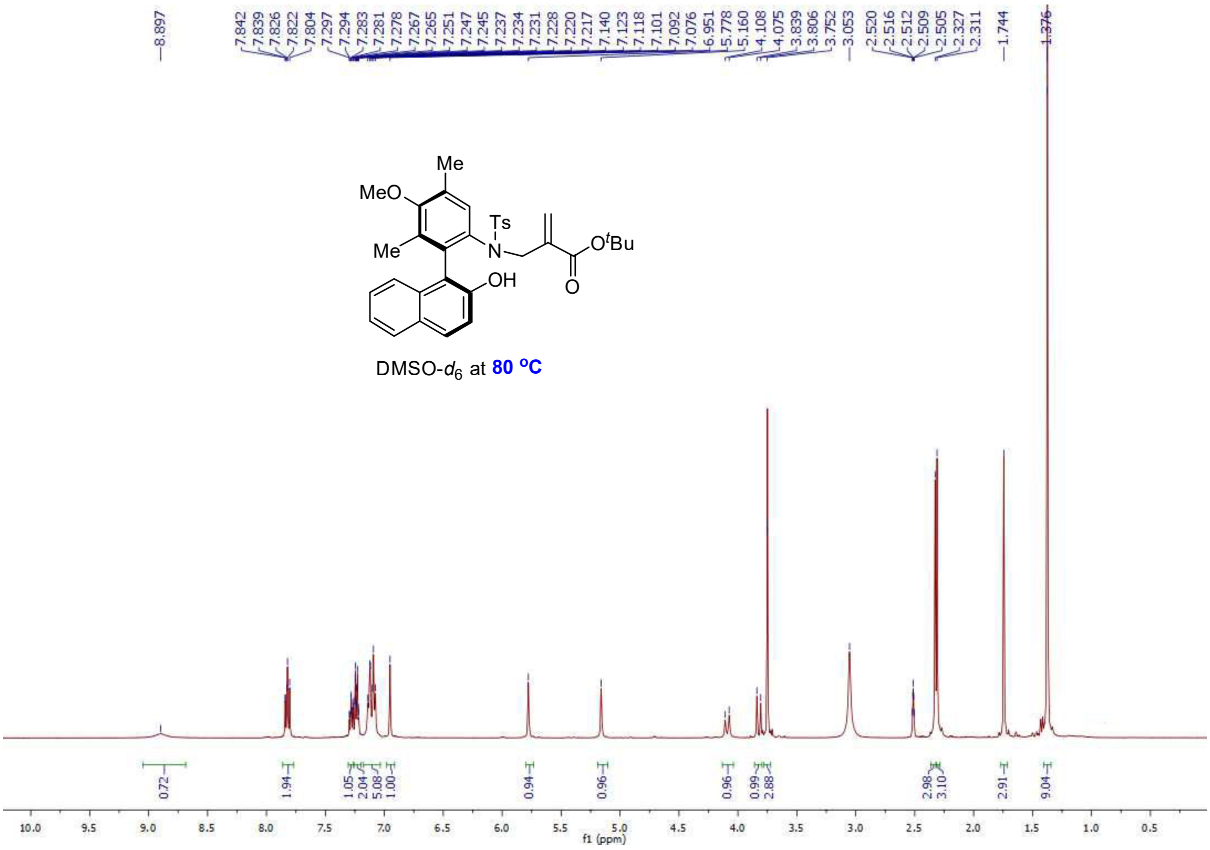

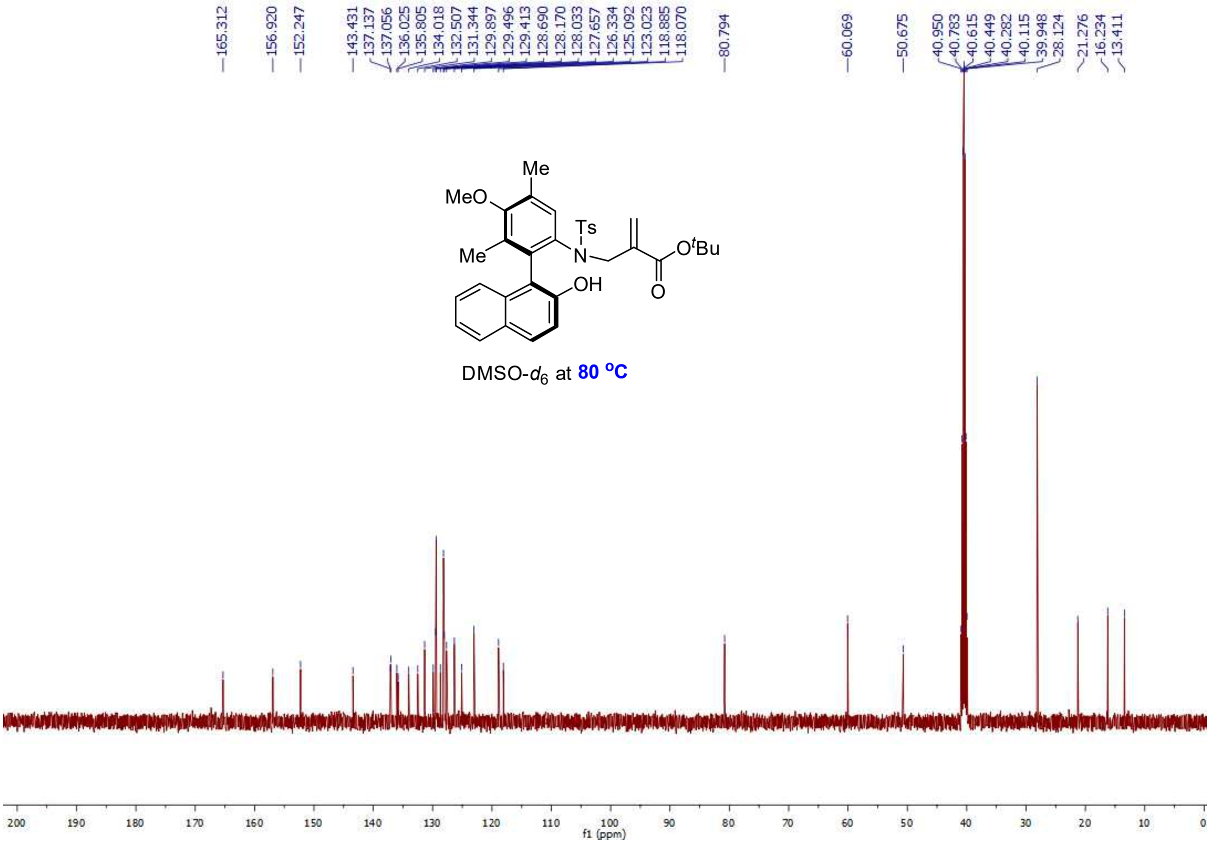

Supplementary Figure 95. ^{1}H and ^{13}C NMR Spectra of **4i**.

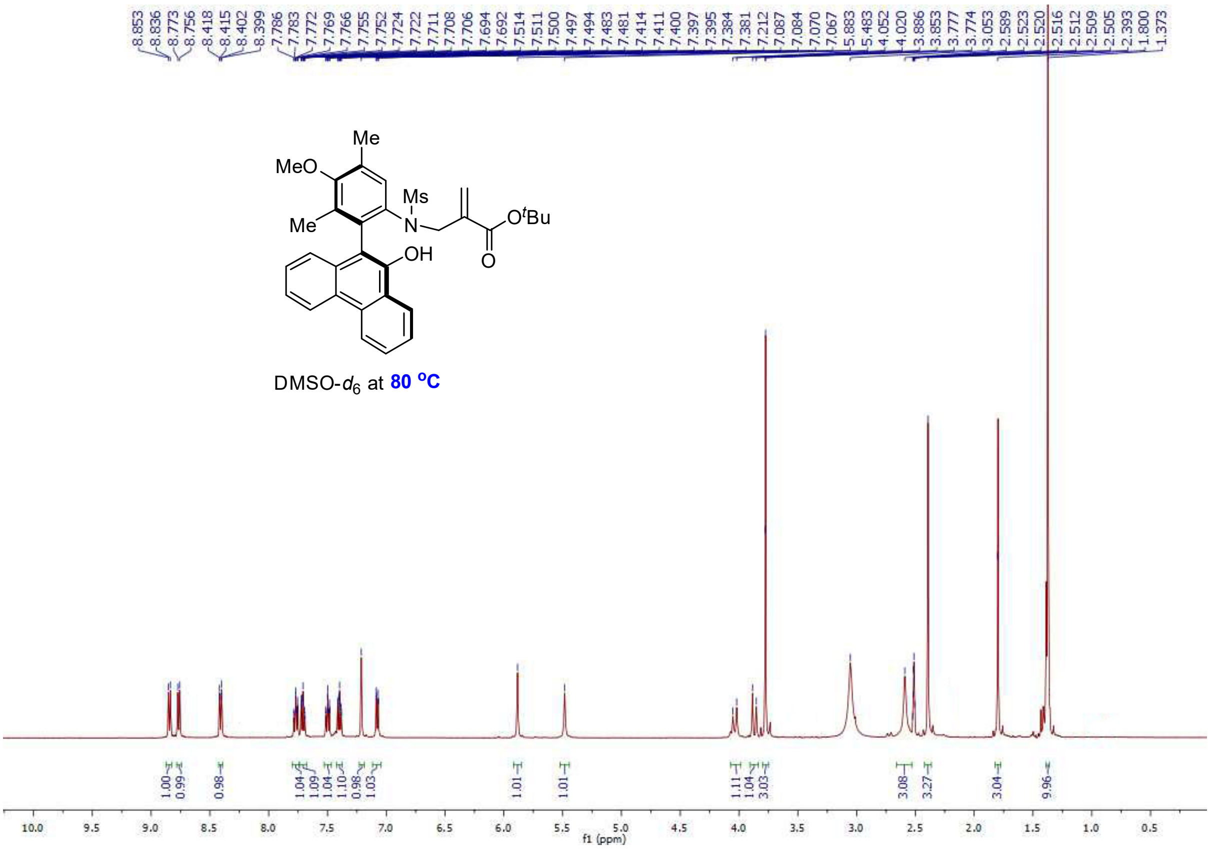

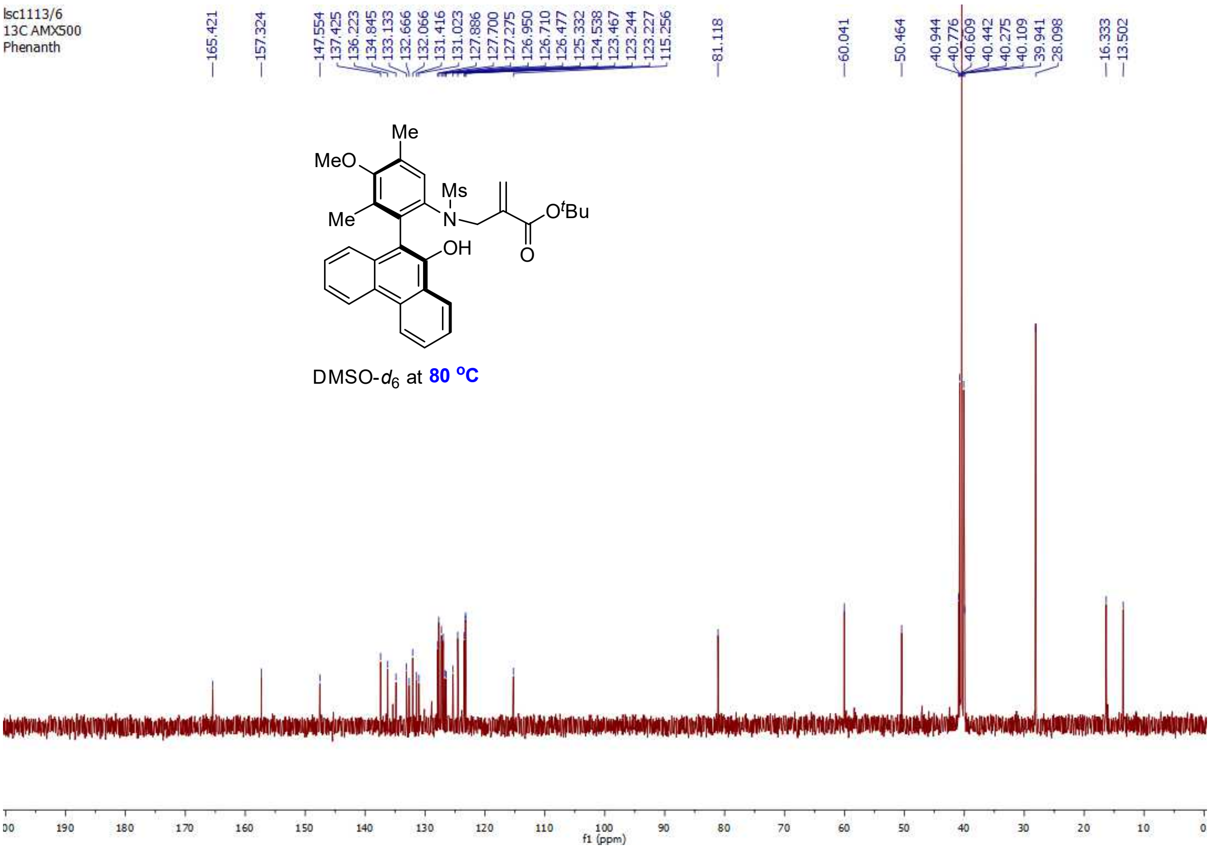

Supplementary Figure 96. ^{1}H and ^{13}C NMR Spectra of **4j**.

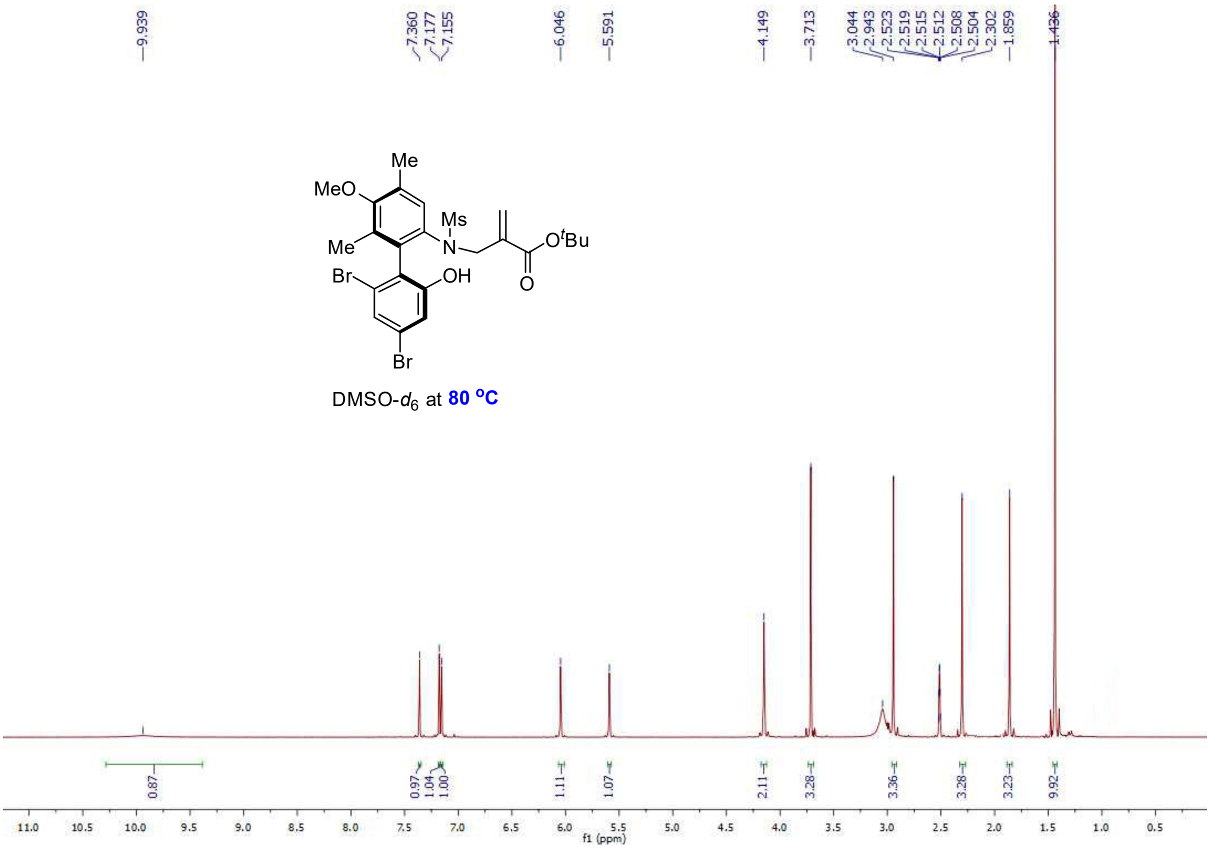

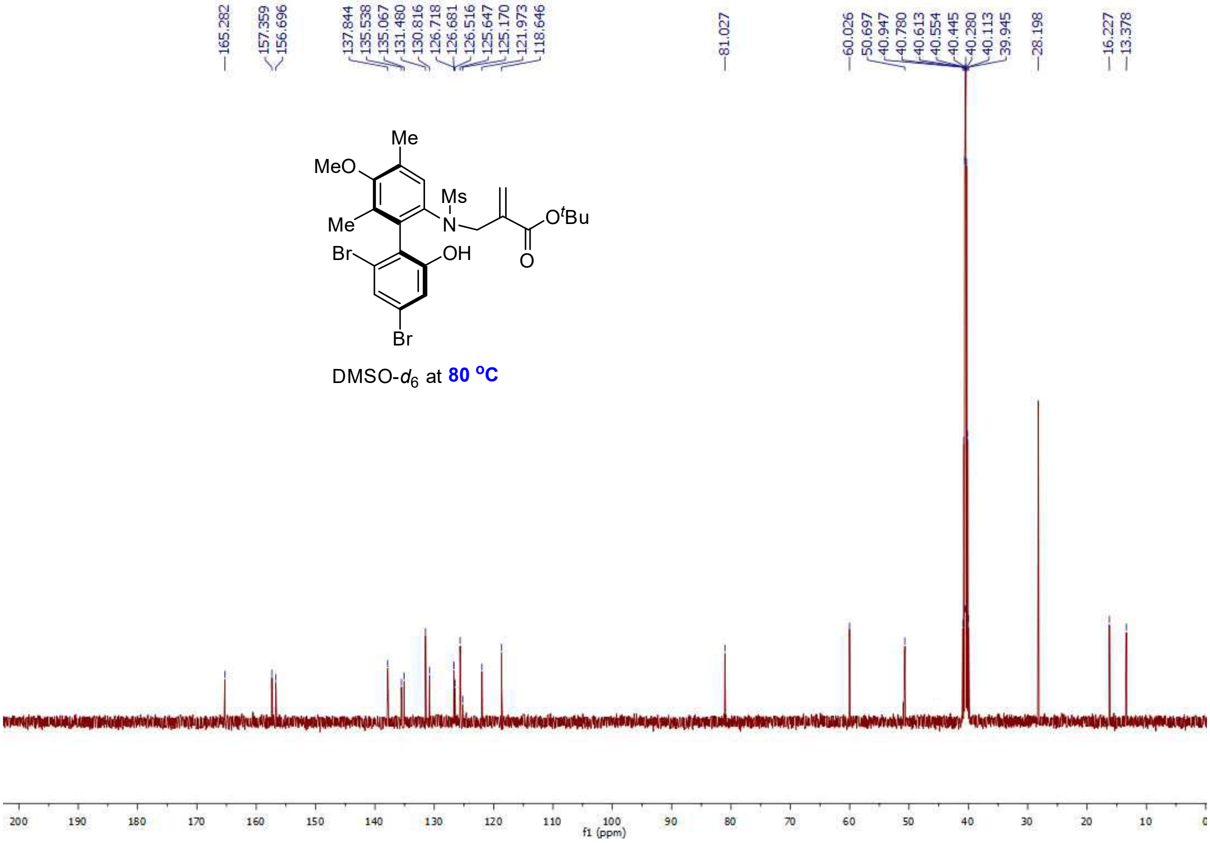

Supplementary Figure 97. ¹H and ¹³C NMR Spectra of **4k**.

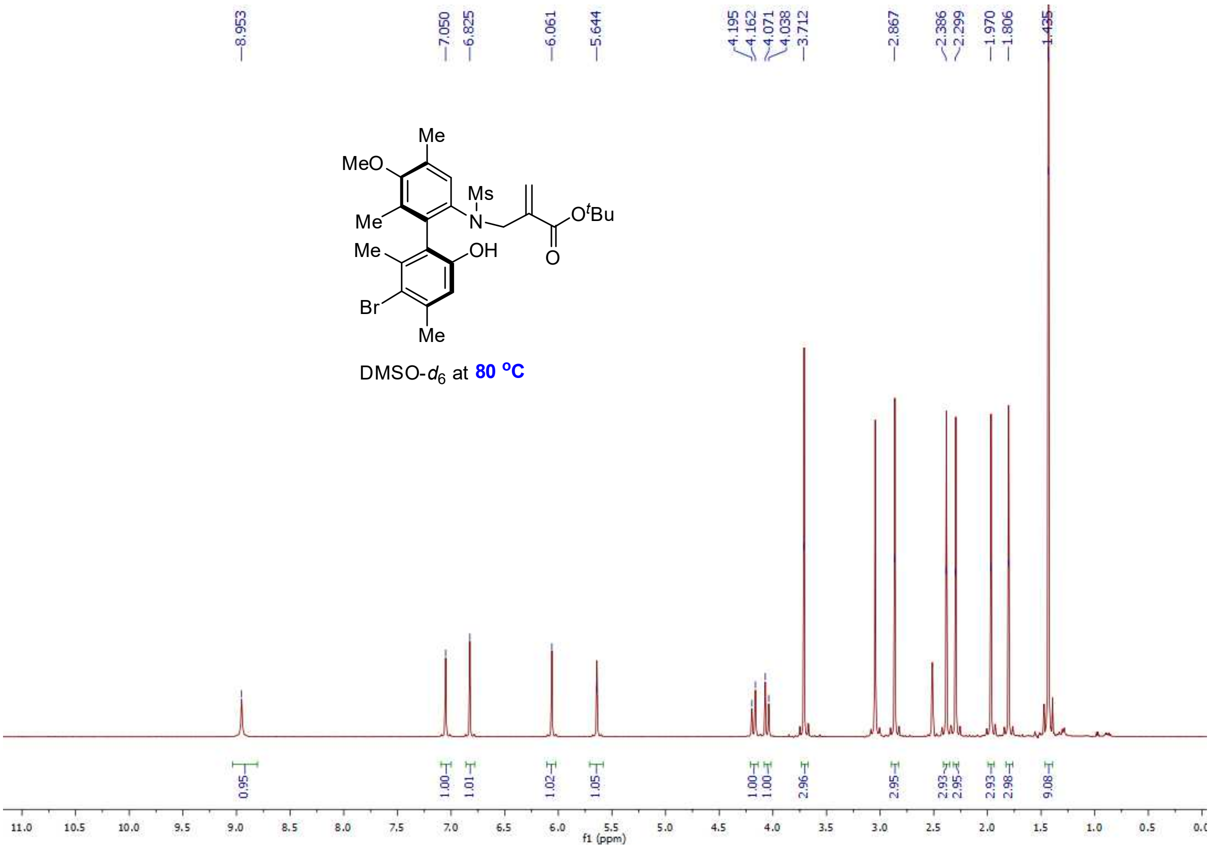

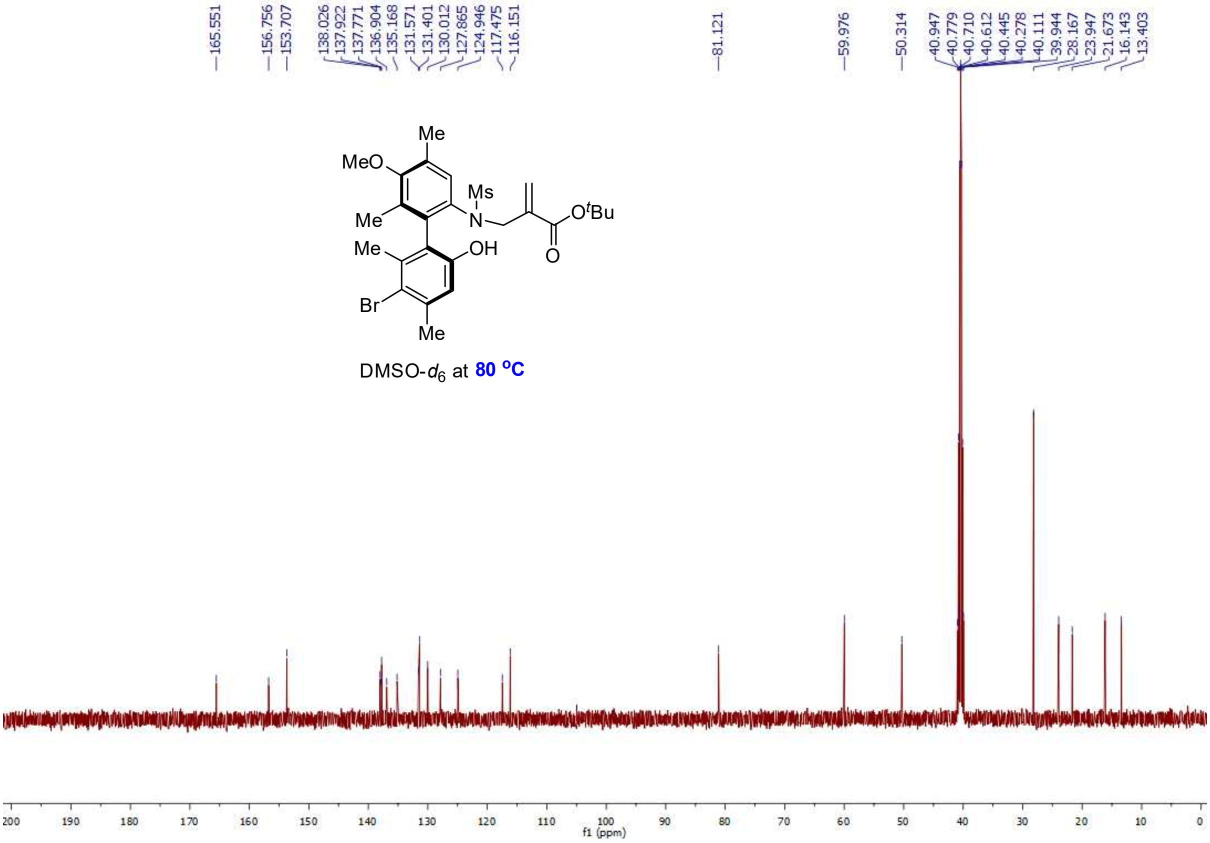

Supplementary Figure 98. ^{1}H and ^{13}C NMR Spectra of **4l**.

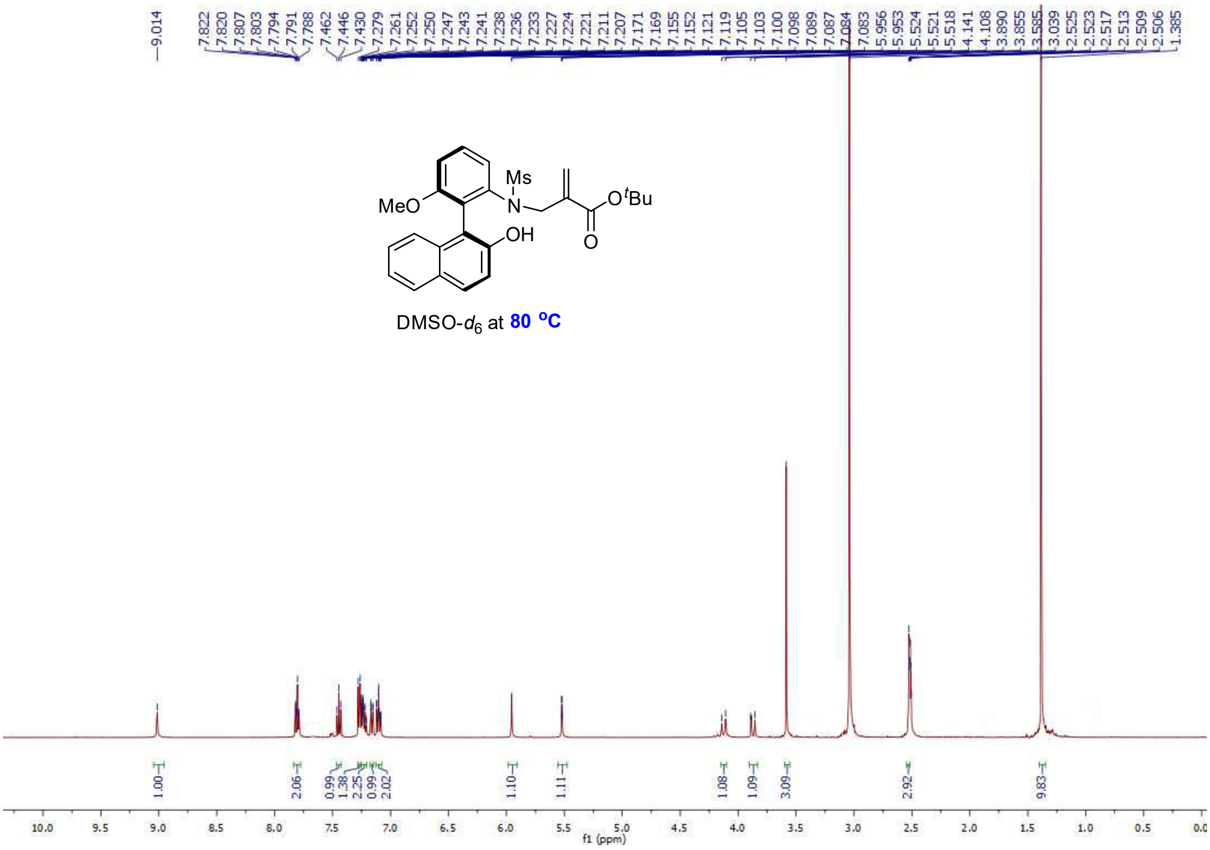

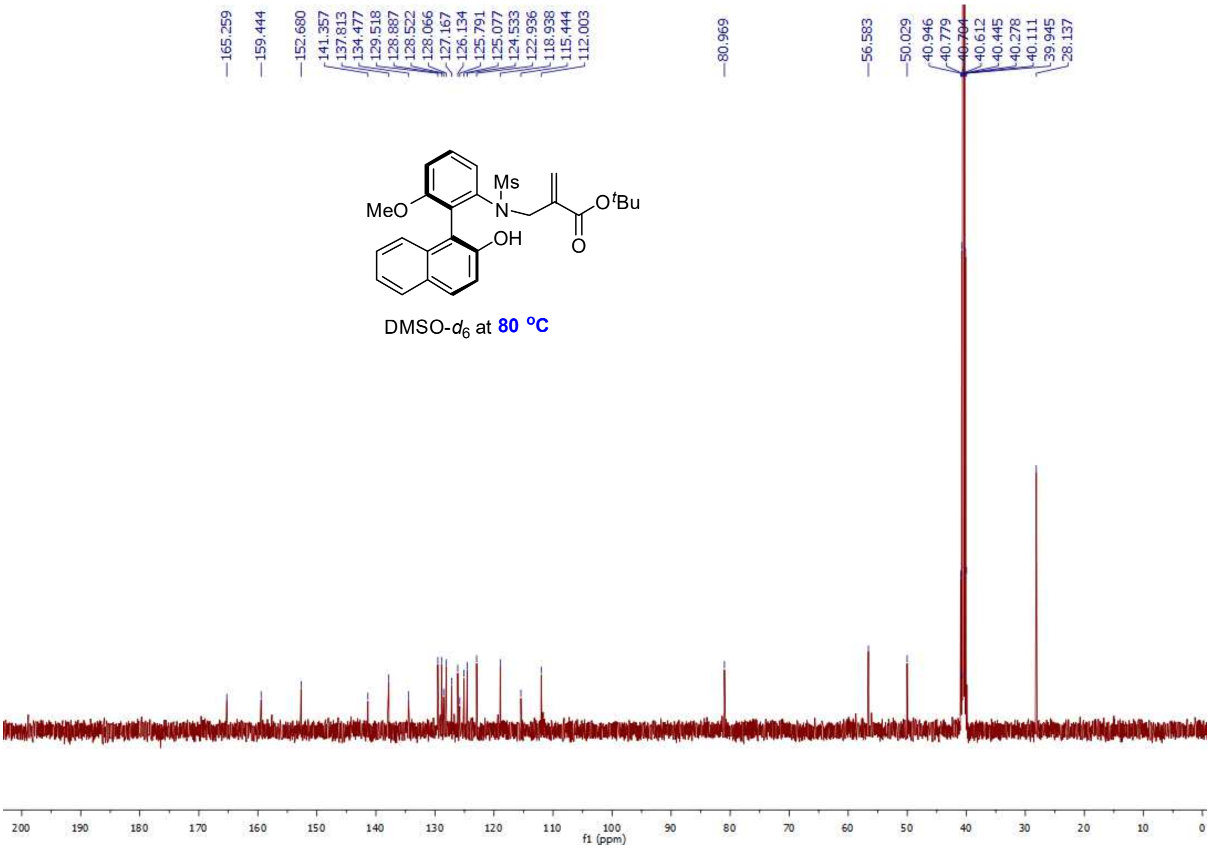

Supplementary Figure 99. ^{1}H and ^{13}C NMR Spectra of **4m**.

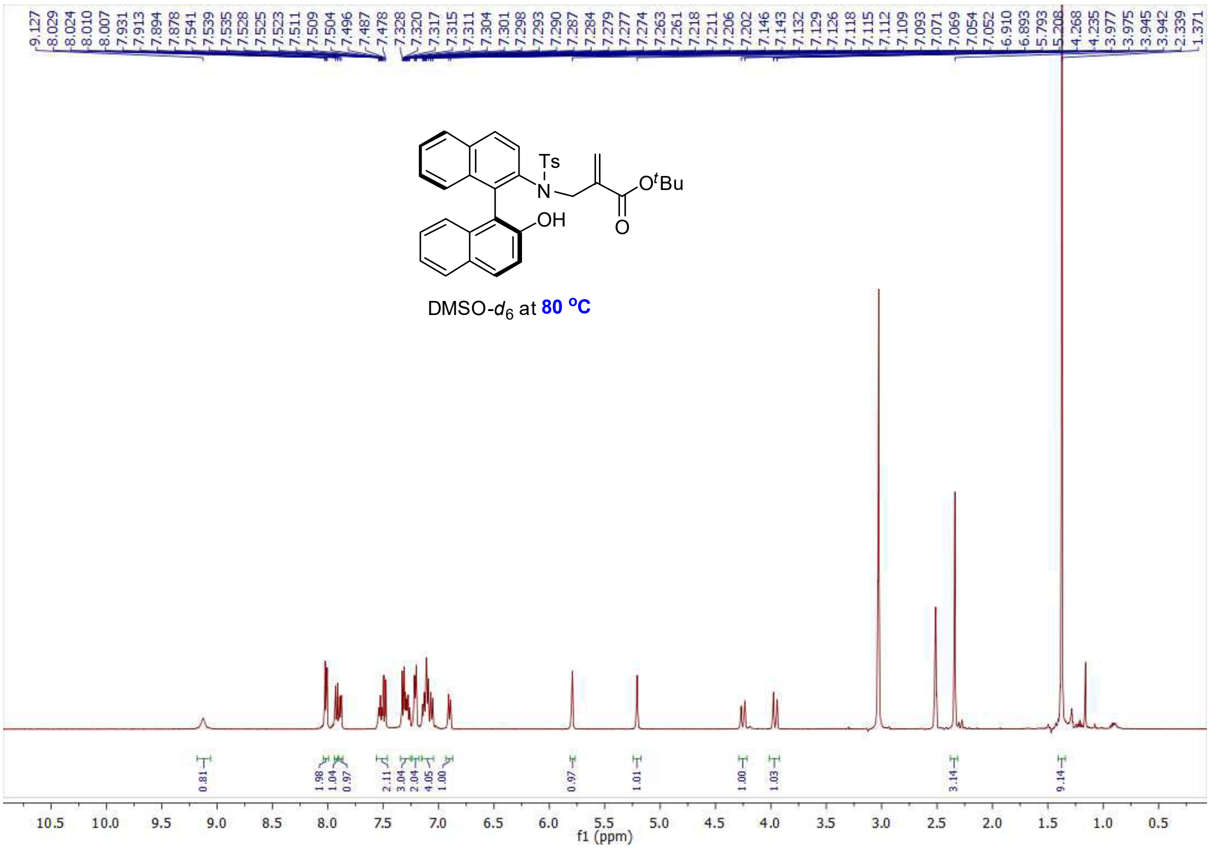

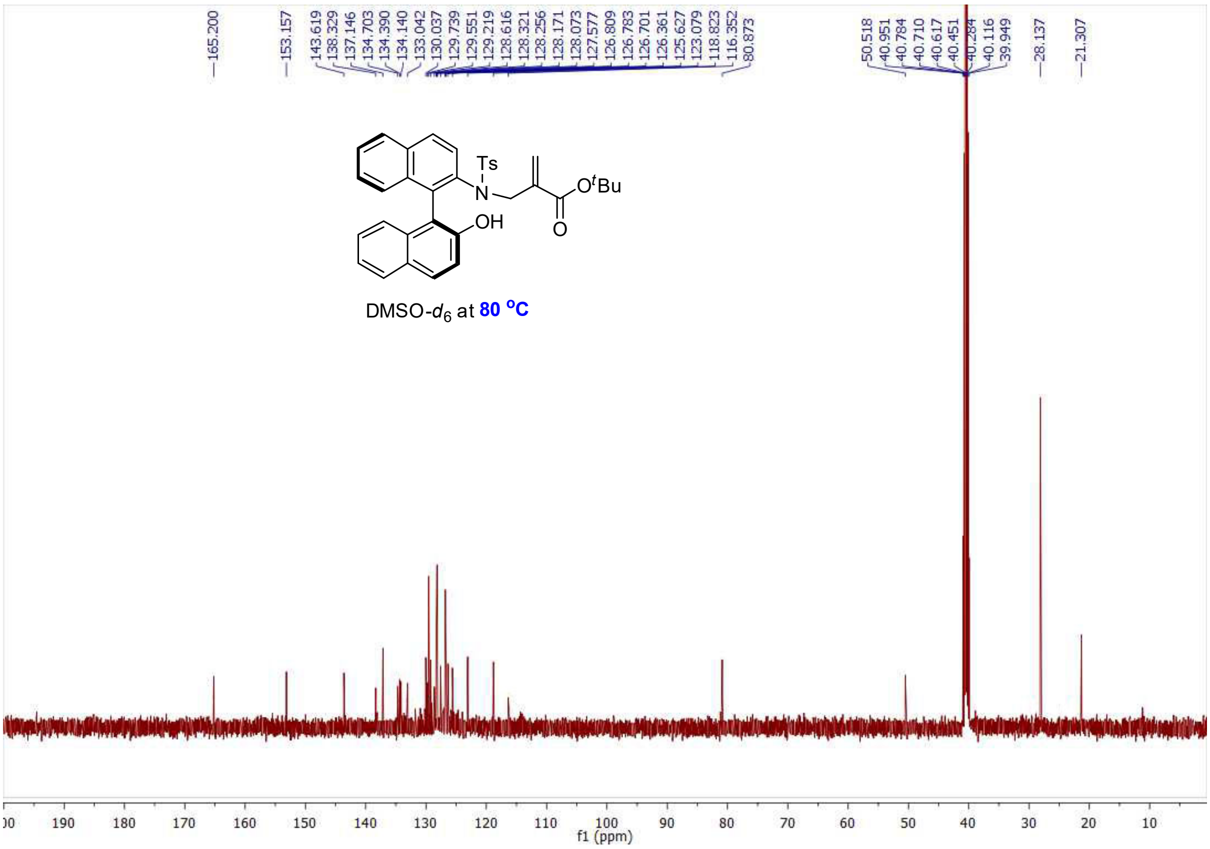

Supplementary Figure 100. ^{1}H and ^{13}C NMR Spectra of **4n**.

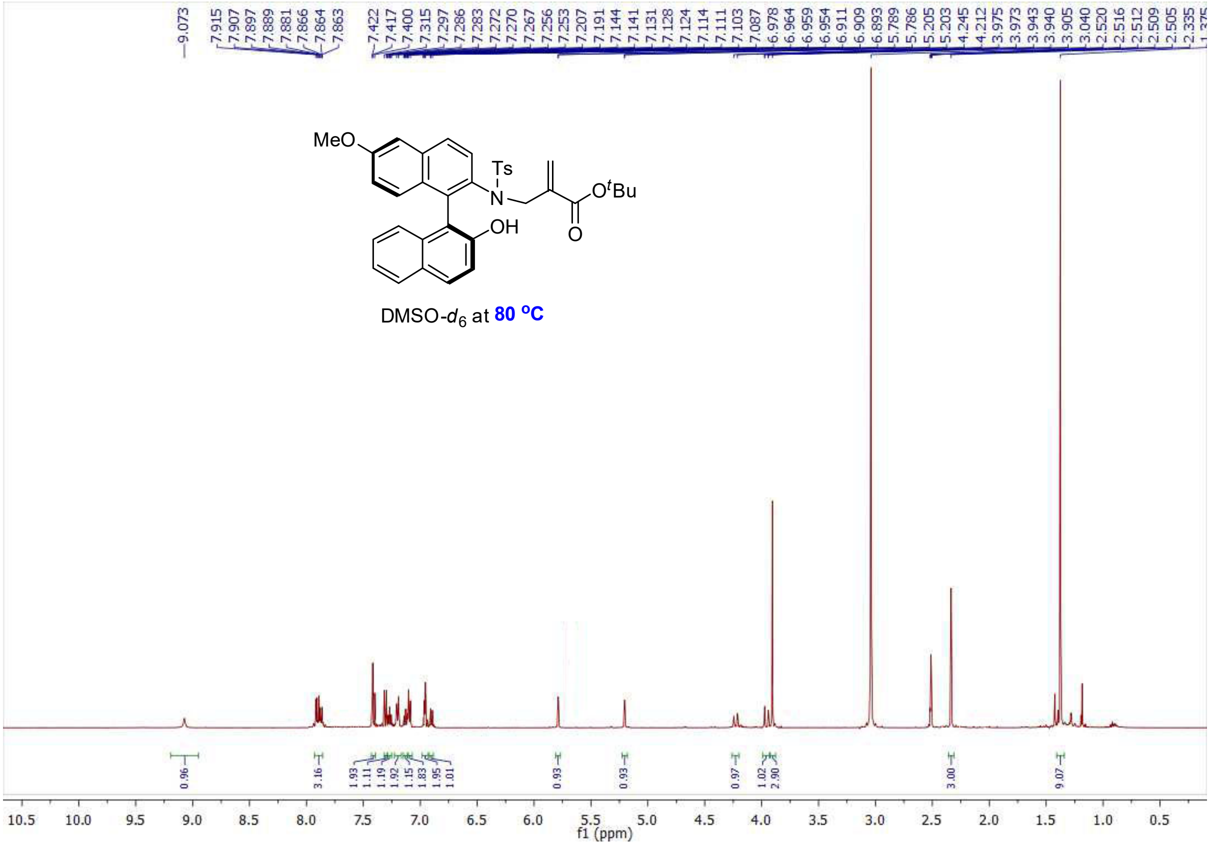

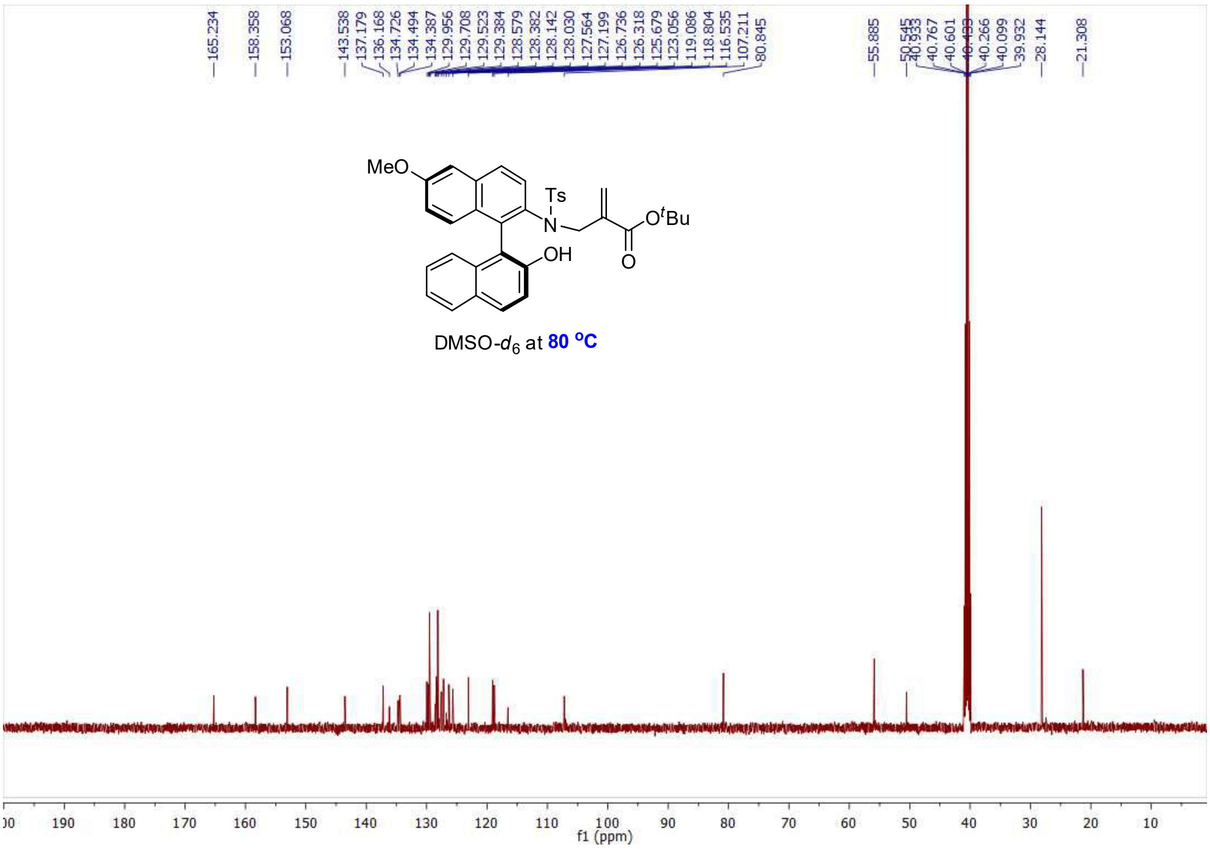

Supplementary Figure 101. ^{1}H and ^{13}C NMR Spectra of **4o**.

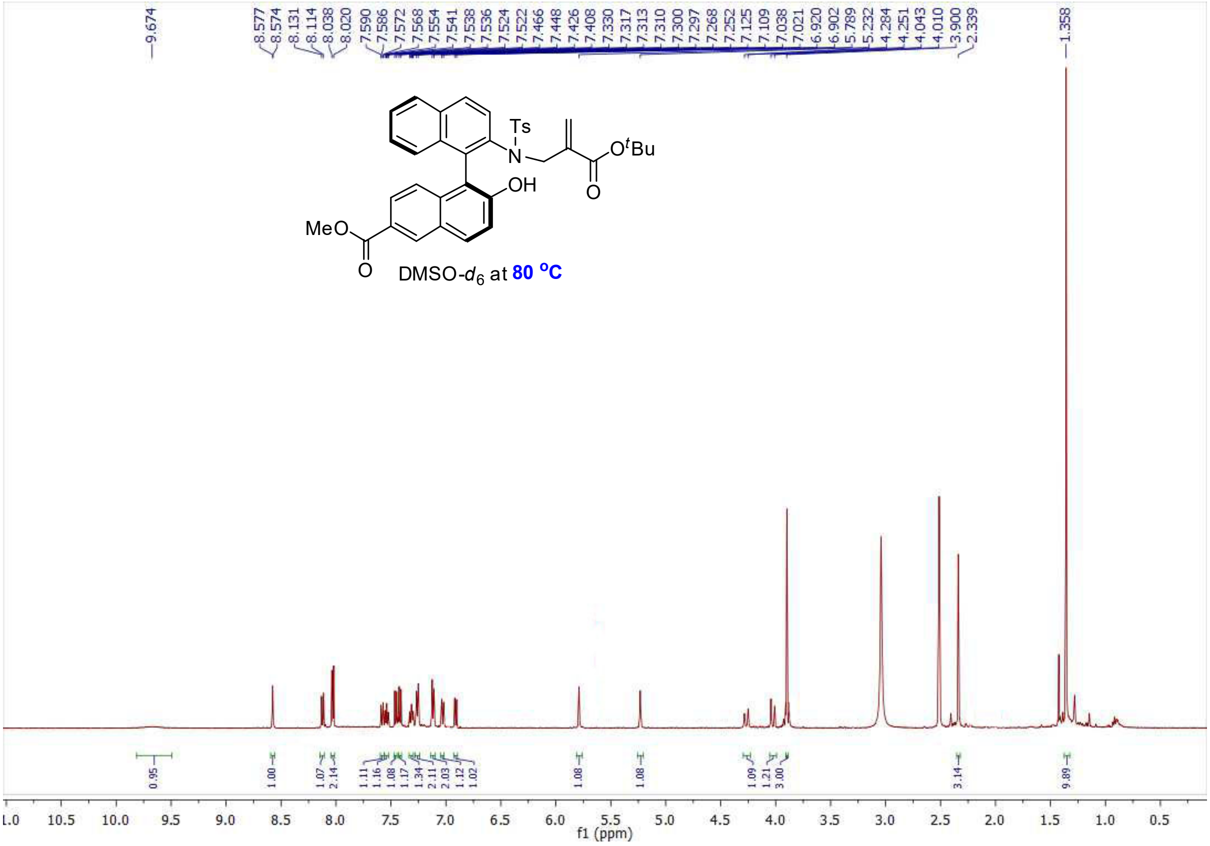

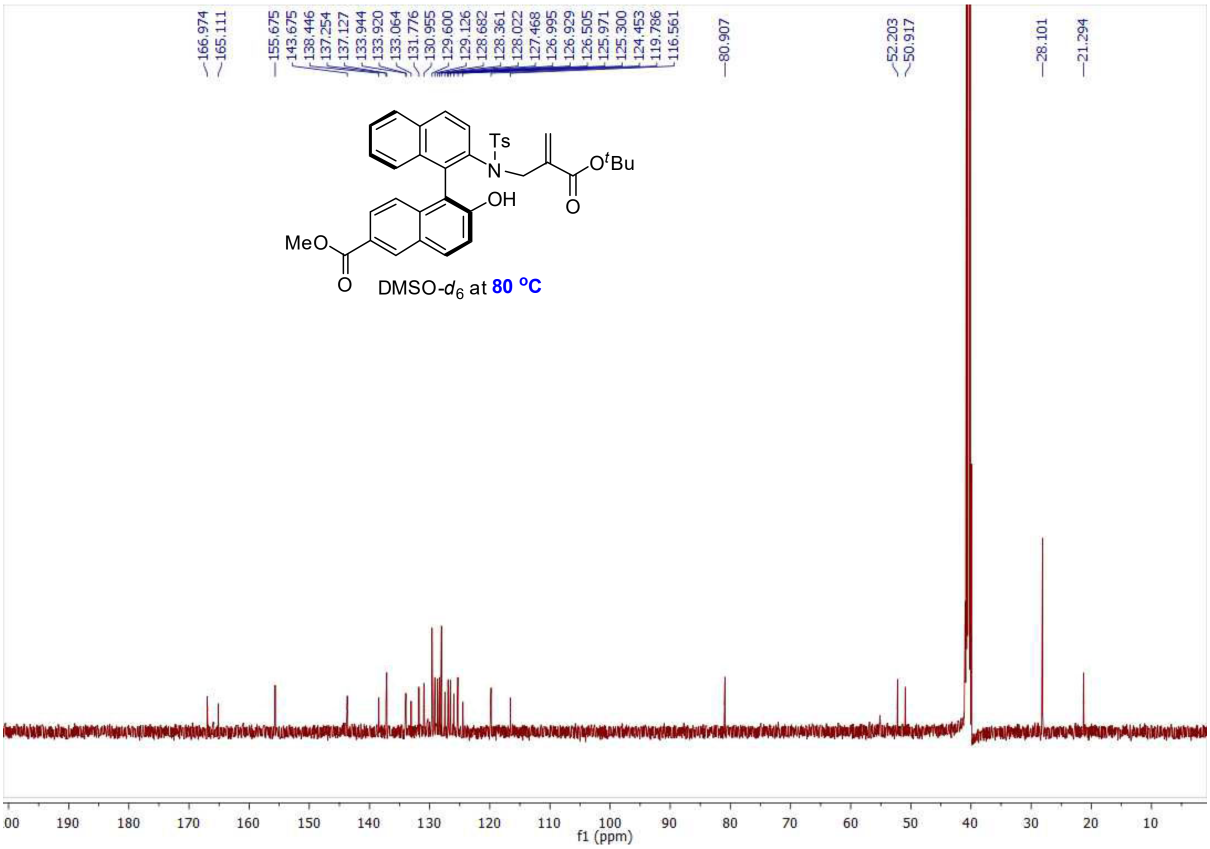

Supplementary Figure 102. ^{1}H and ^{13}C NMR Spectra of **4p**.

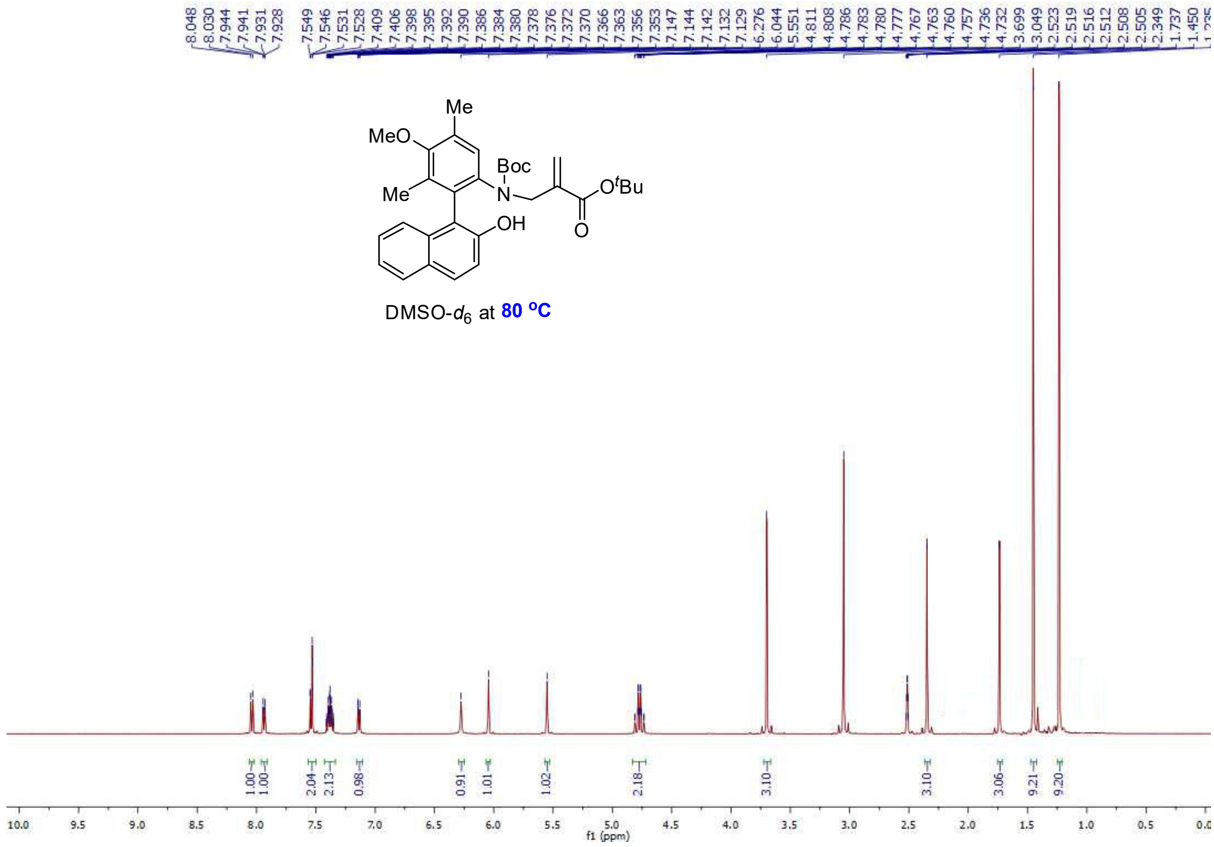

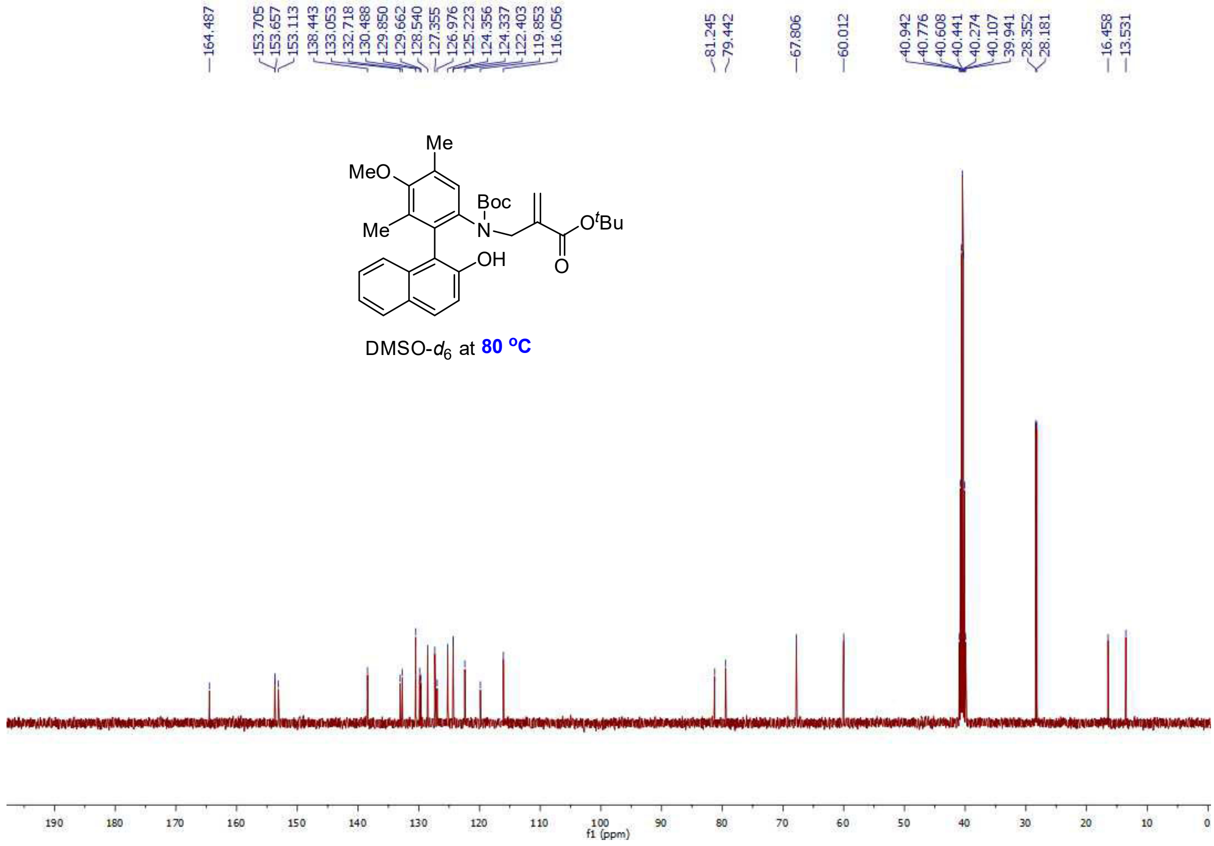

Supplementary Figure 103. ^{1}H and ^{13}C NMR Spectra of **4q**.

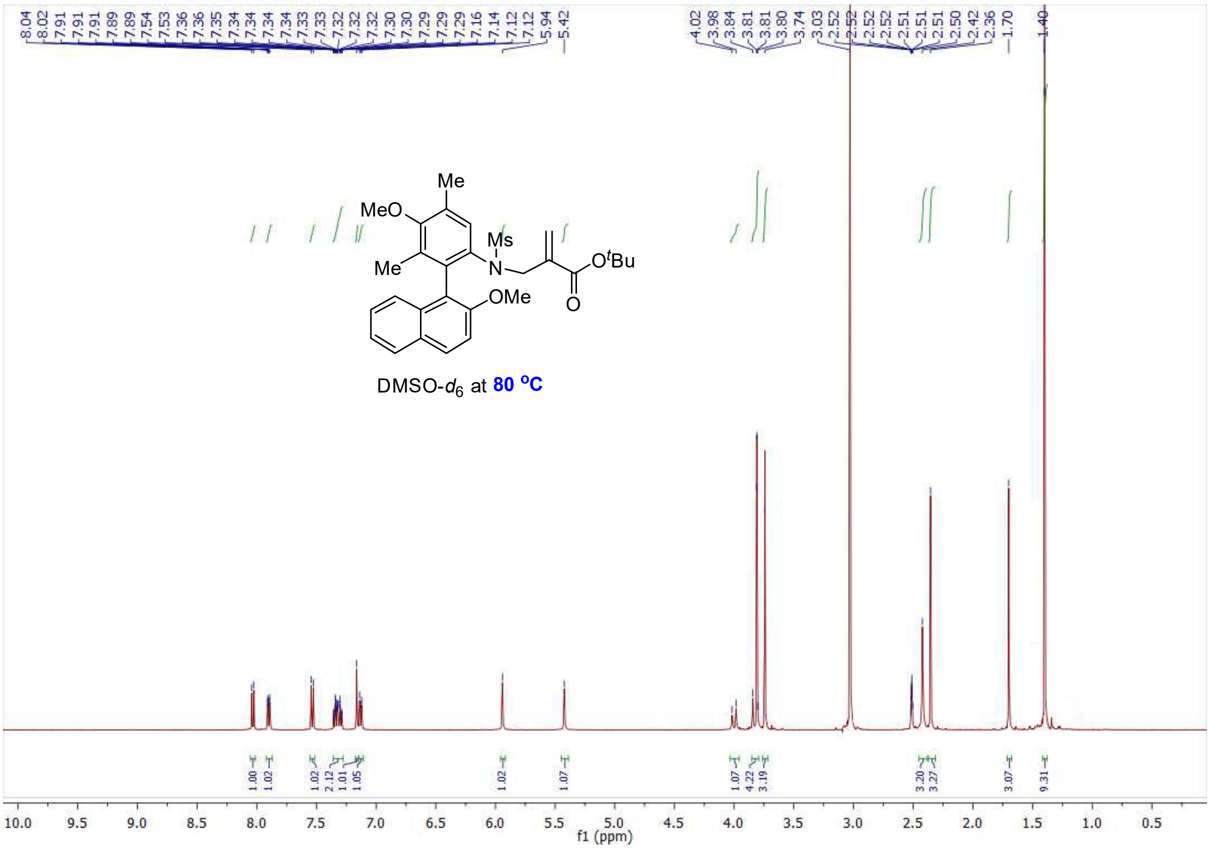

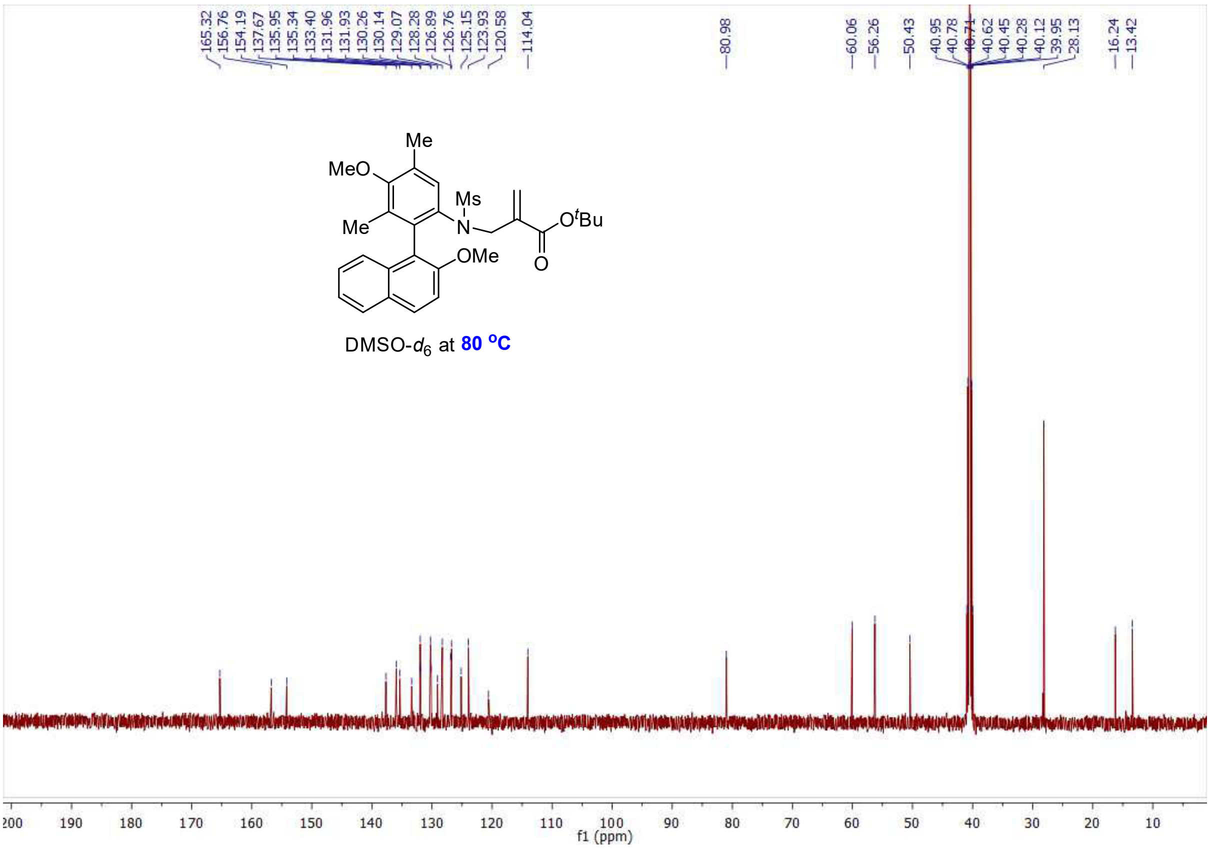

Supplementary Figure 104. ^{1}H and ^{13}C NMR Spectra of **5a**.

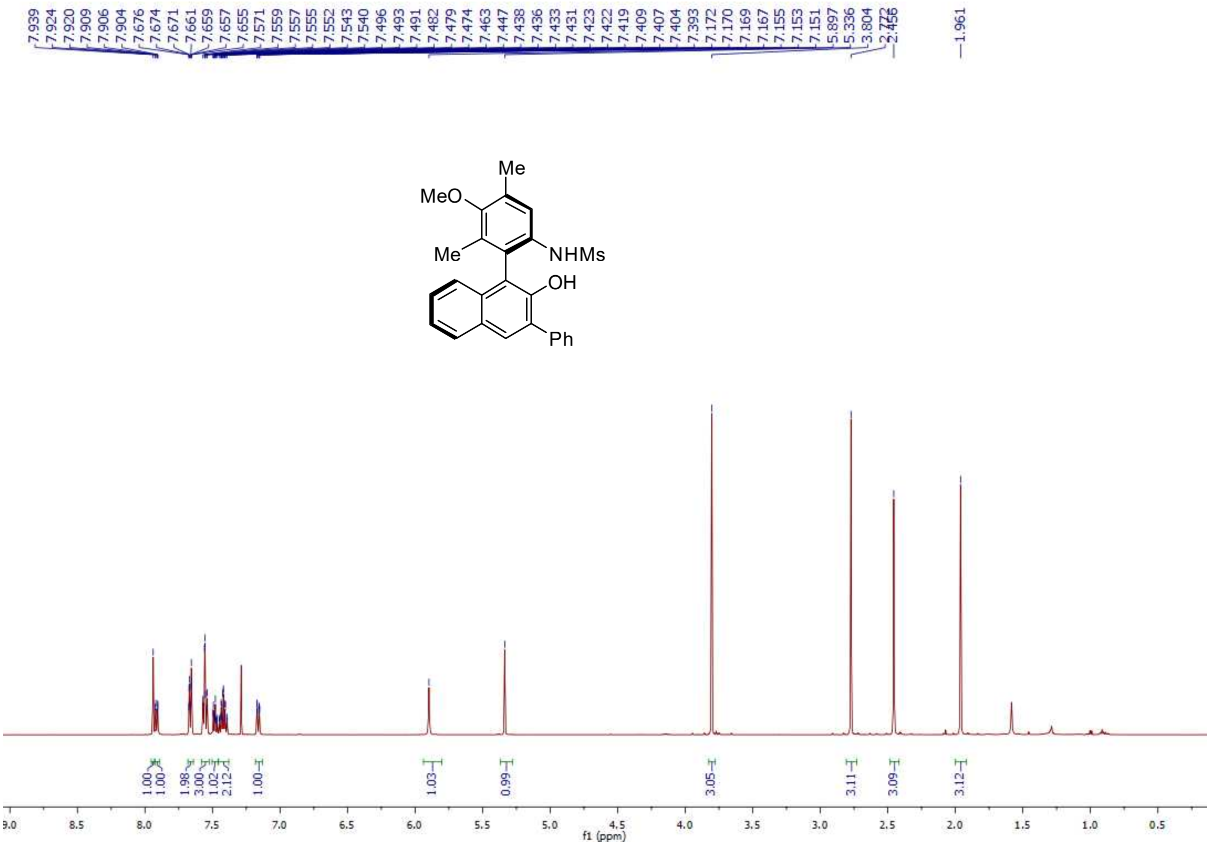

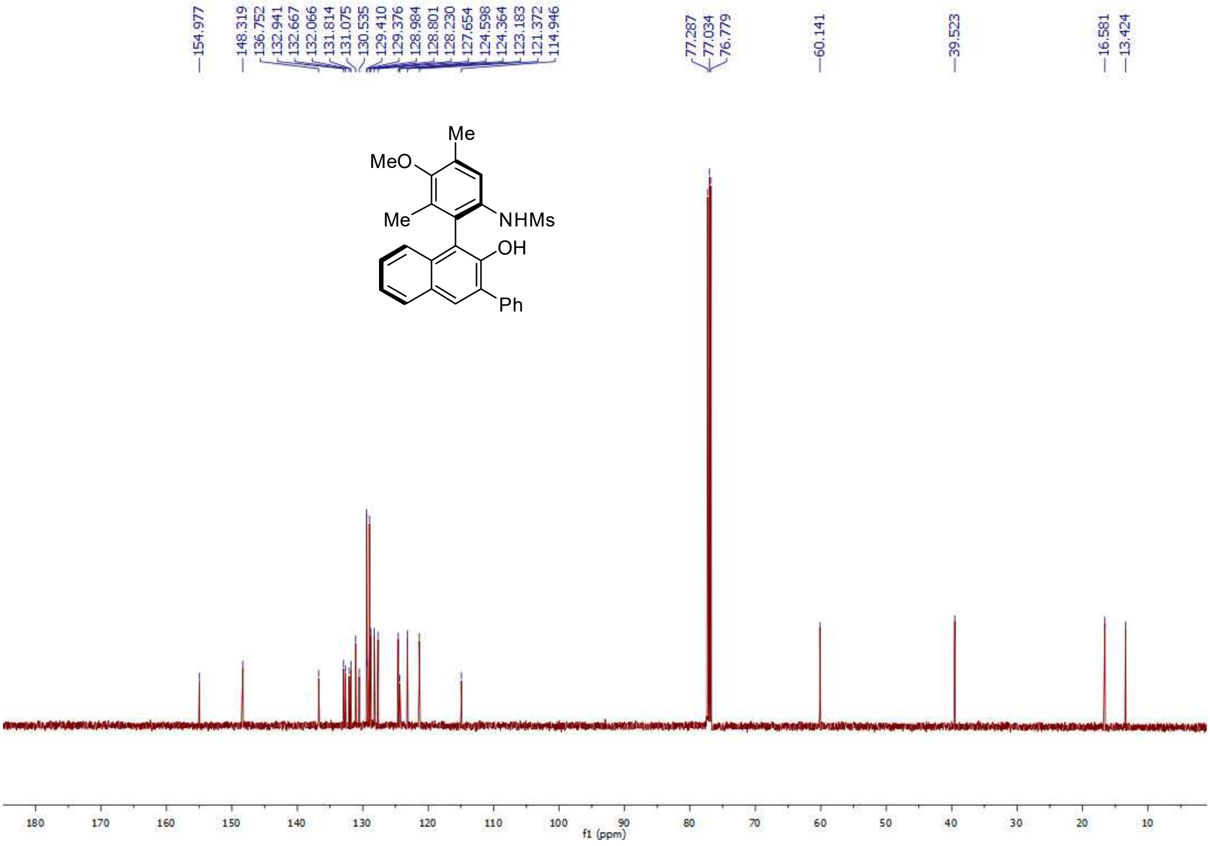

Supplementary Figure 105. ^{1}H and ^{13}C NMR Spectra of **5b**.

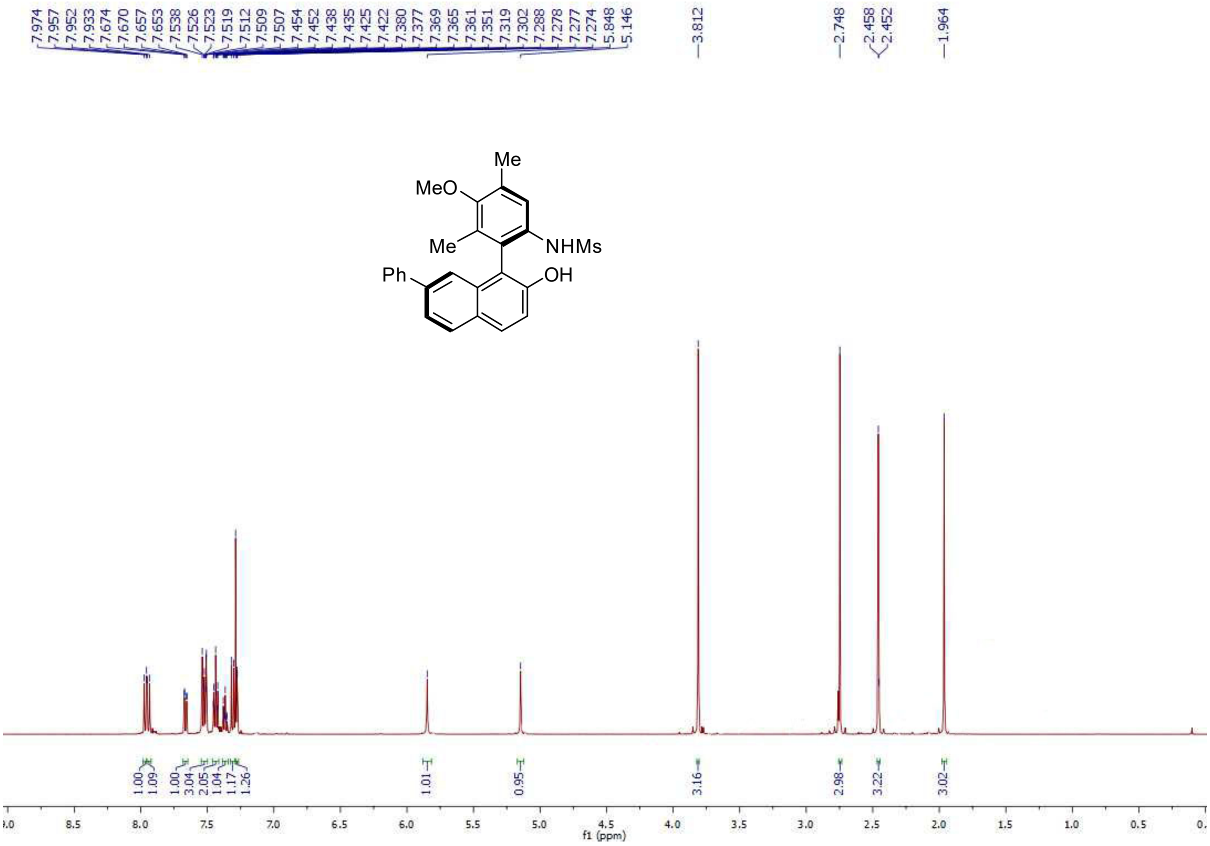

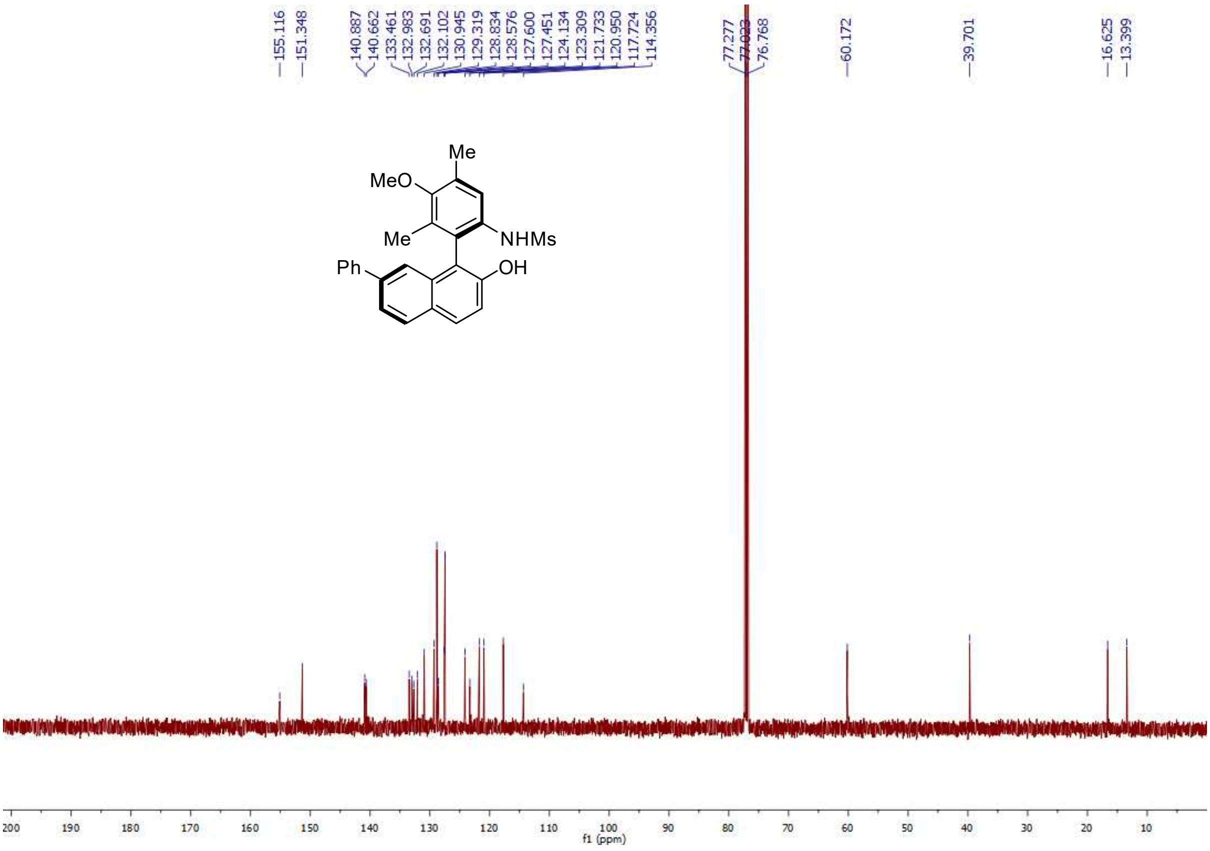

Supplementary Figure 106. ^{1}H and ^{13}C NMR Spectra of **6a**.

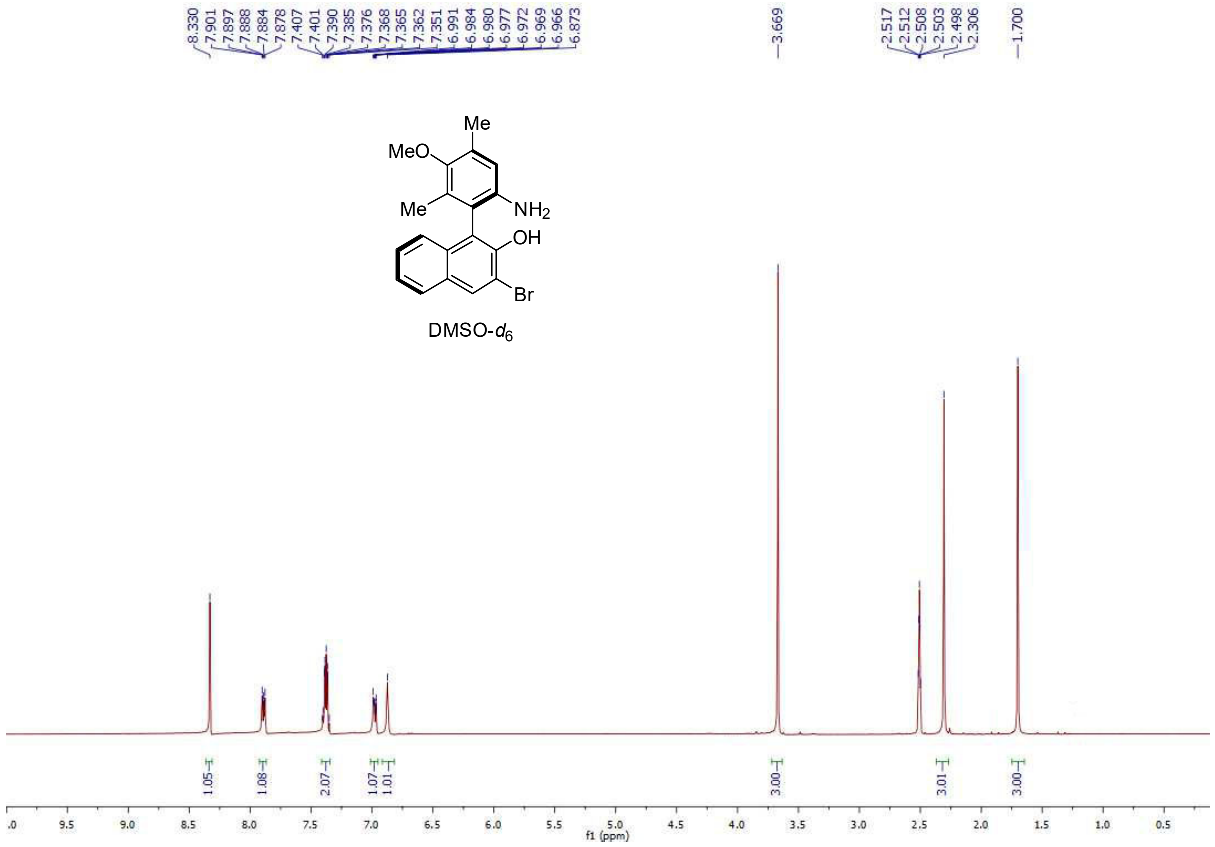

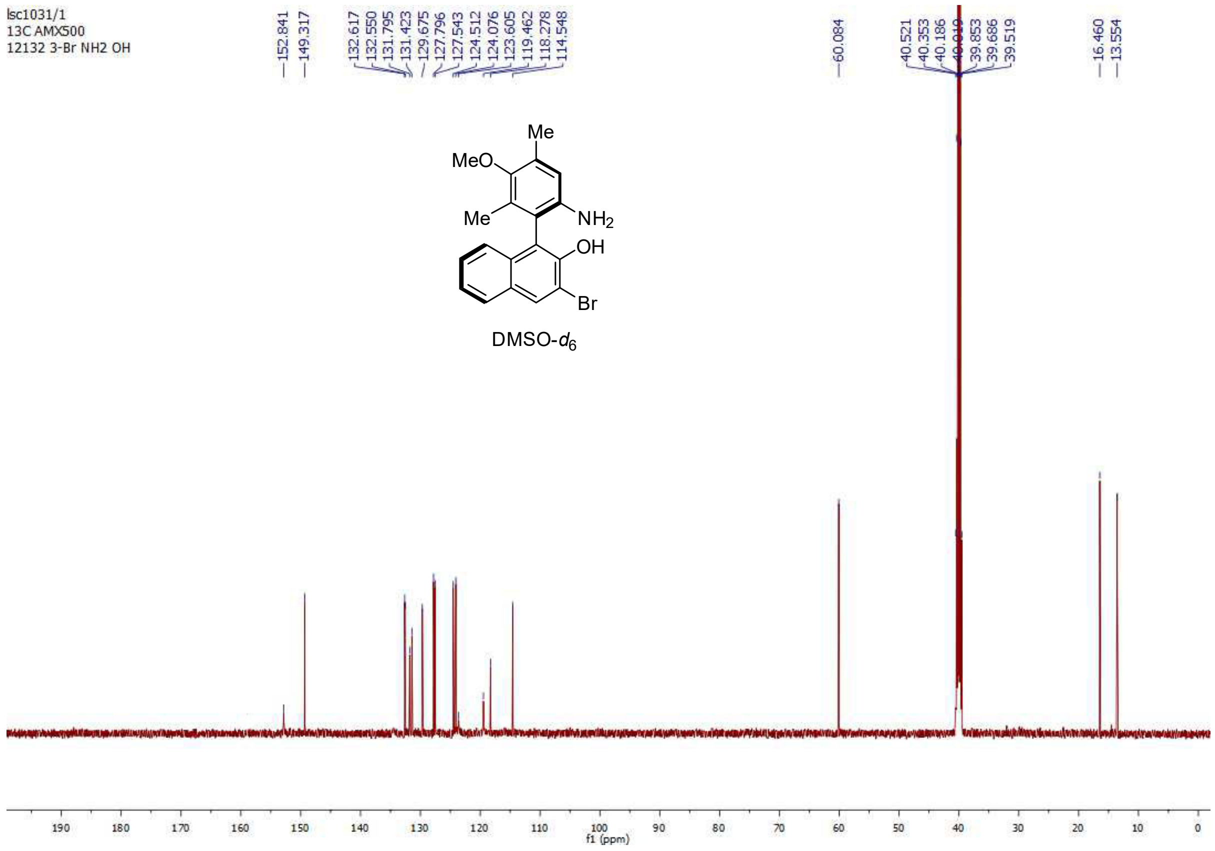

Supplementary Figure 107. ^{1}H and ^{13}C NMR Spectra of **6b**.

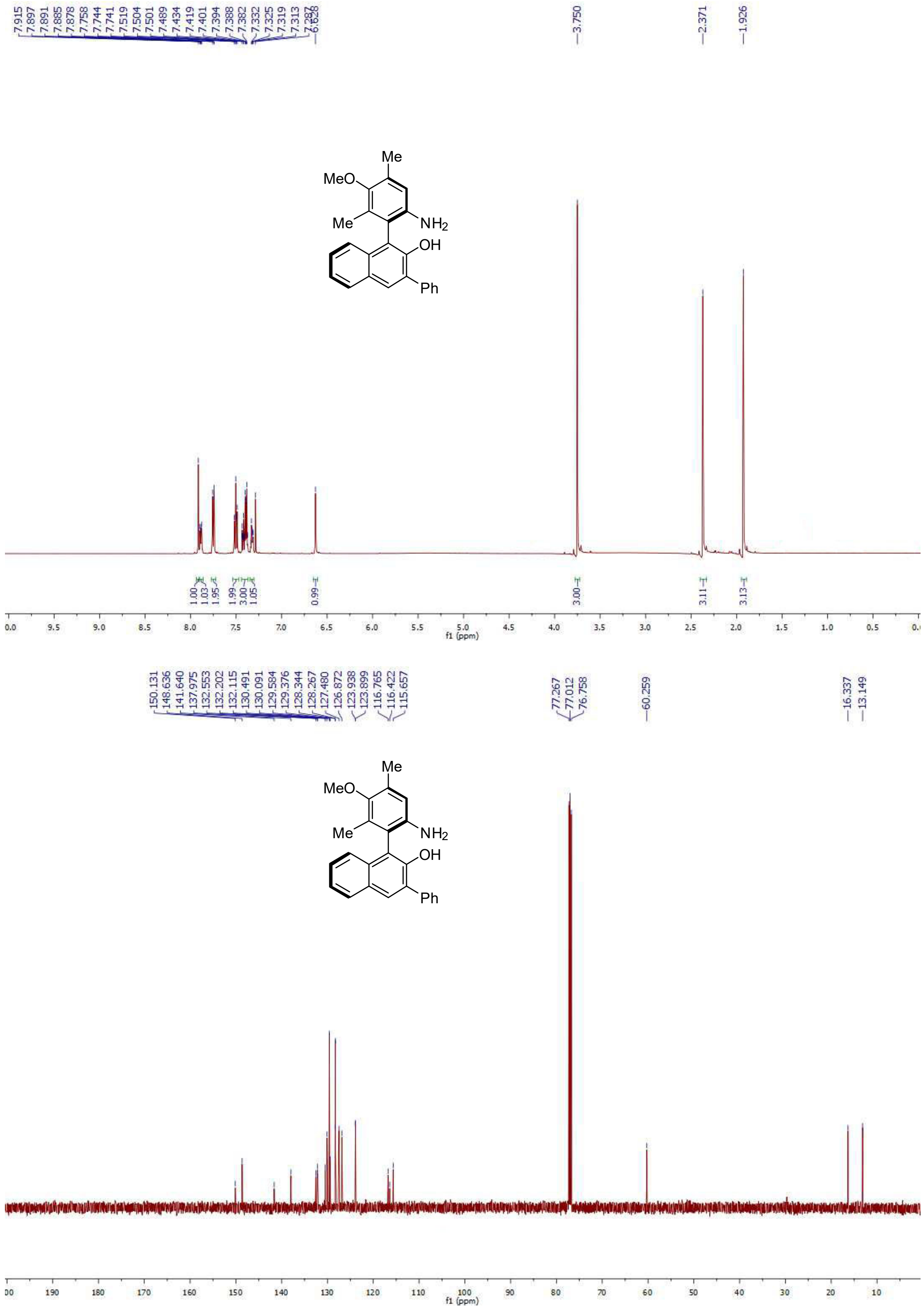

Supplementary Figure 108. ^{1}H and ^{13}C NMR Spectra of **7**.

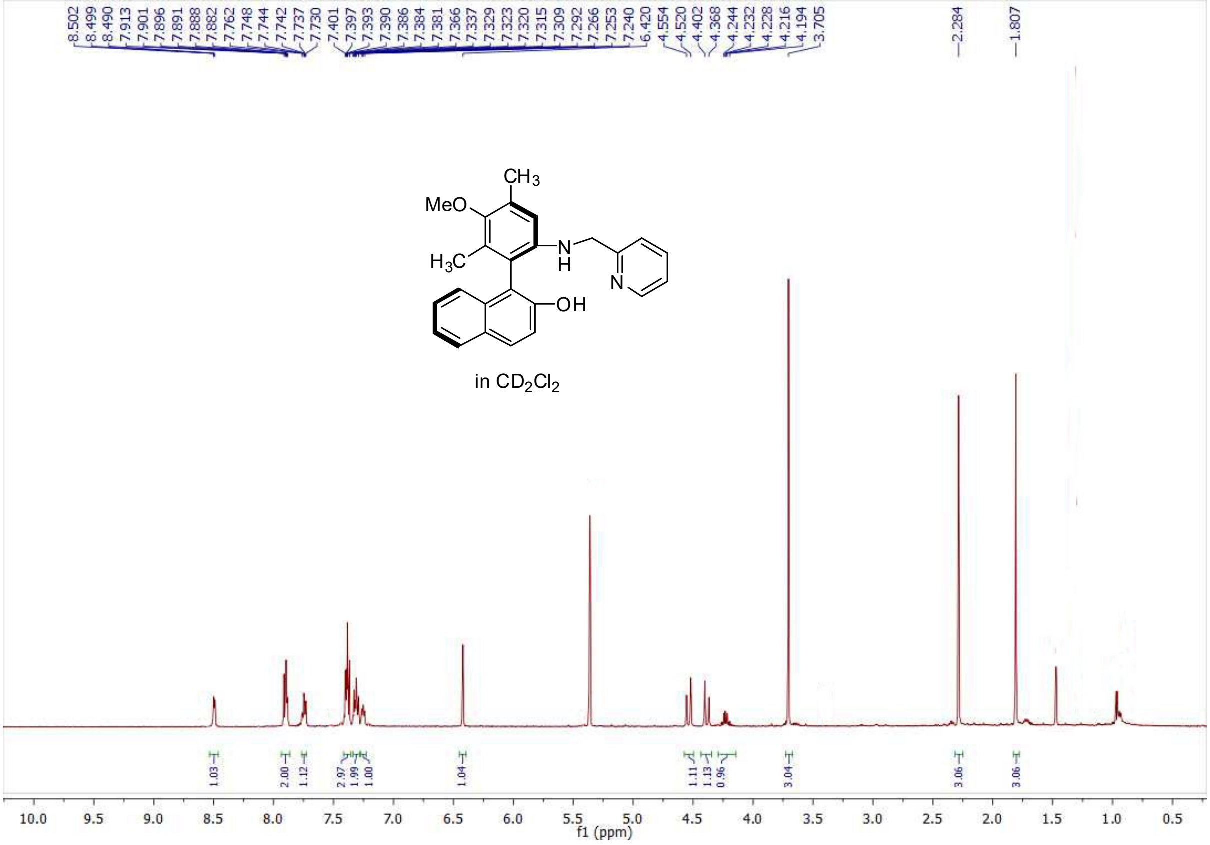

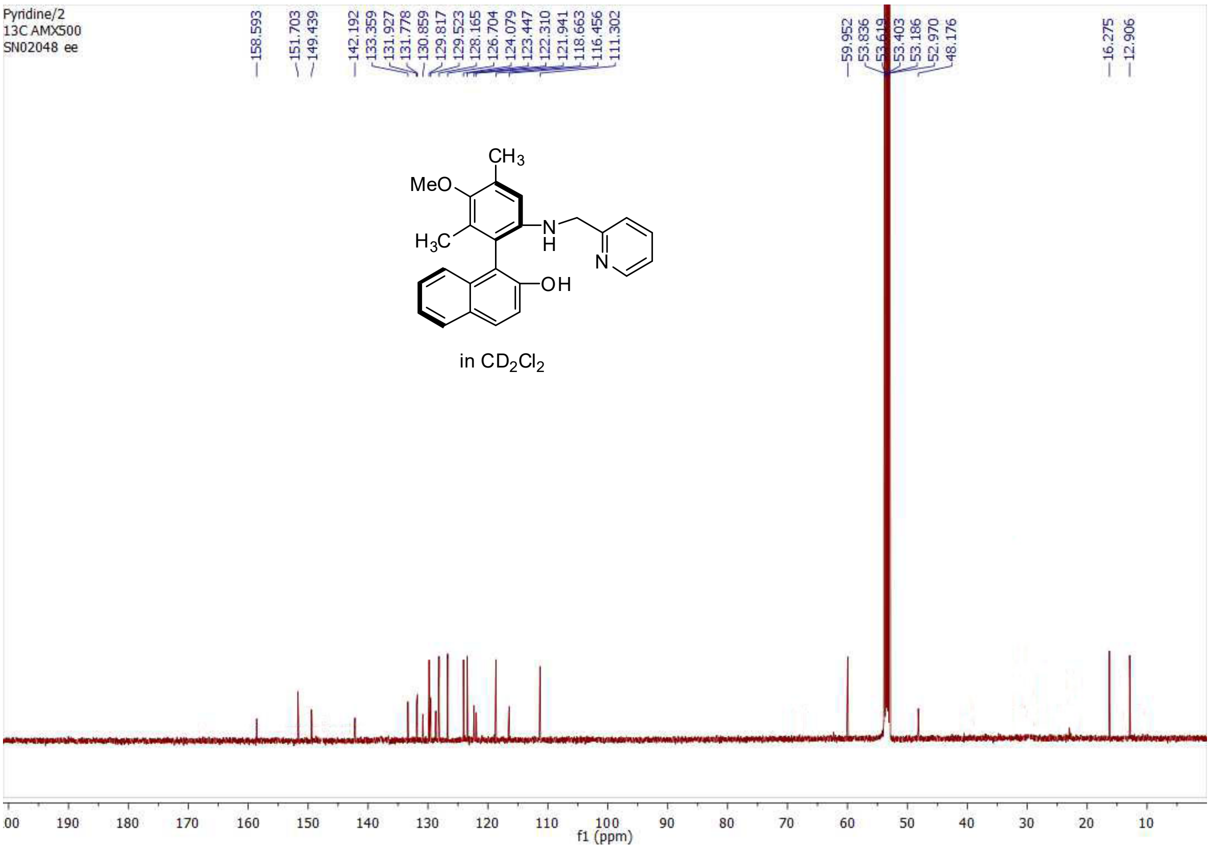

Supplementary Figure 109. ^{1}H and ^{13}C NMR Spectra of **8**.

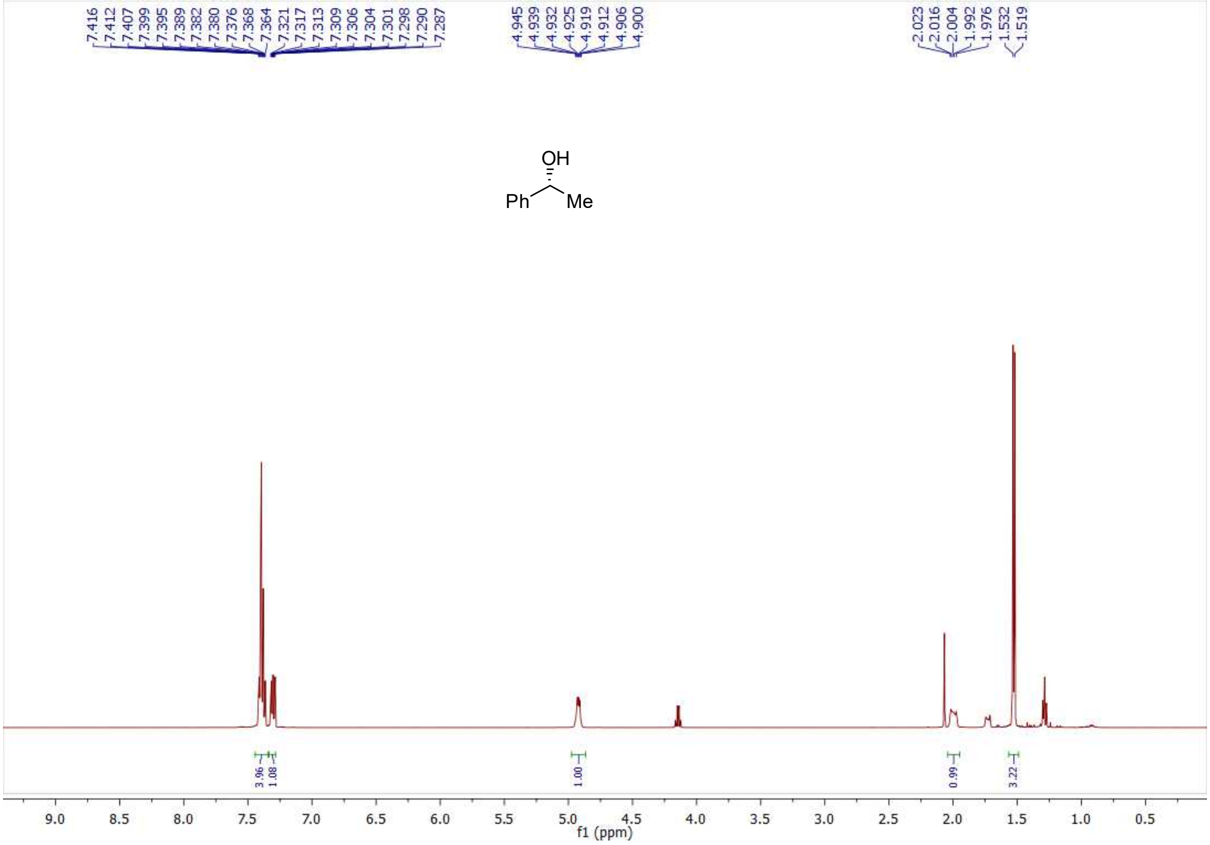

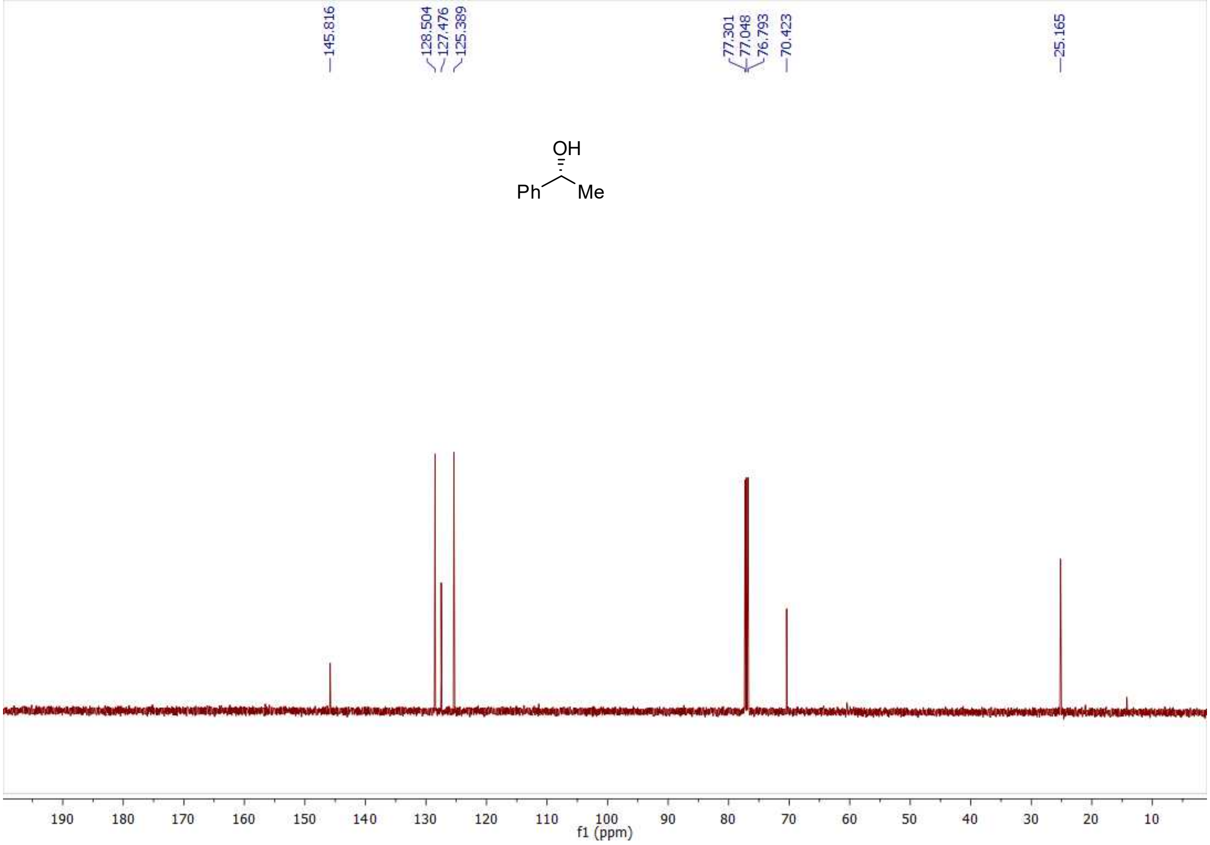

Supplementary Figure 110. ^{1}H and ^{13}C NMR Spectra of **9aa**.

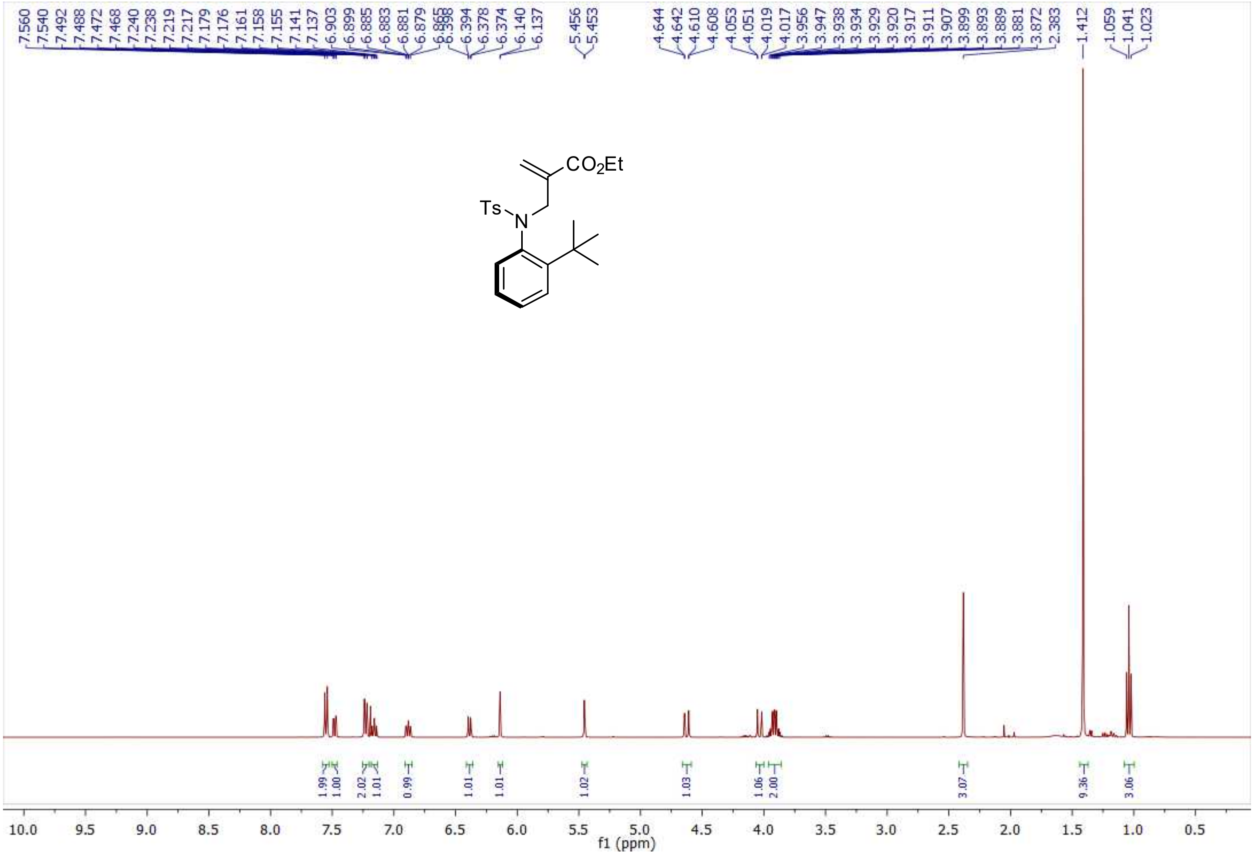

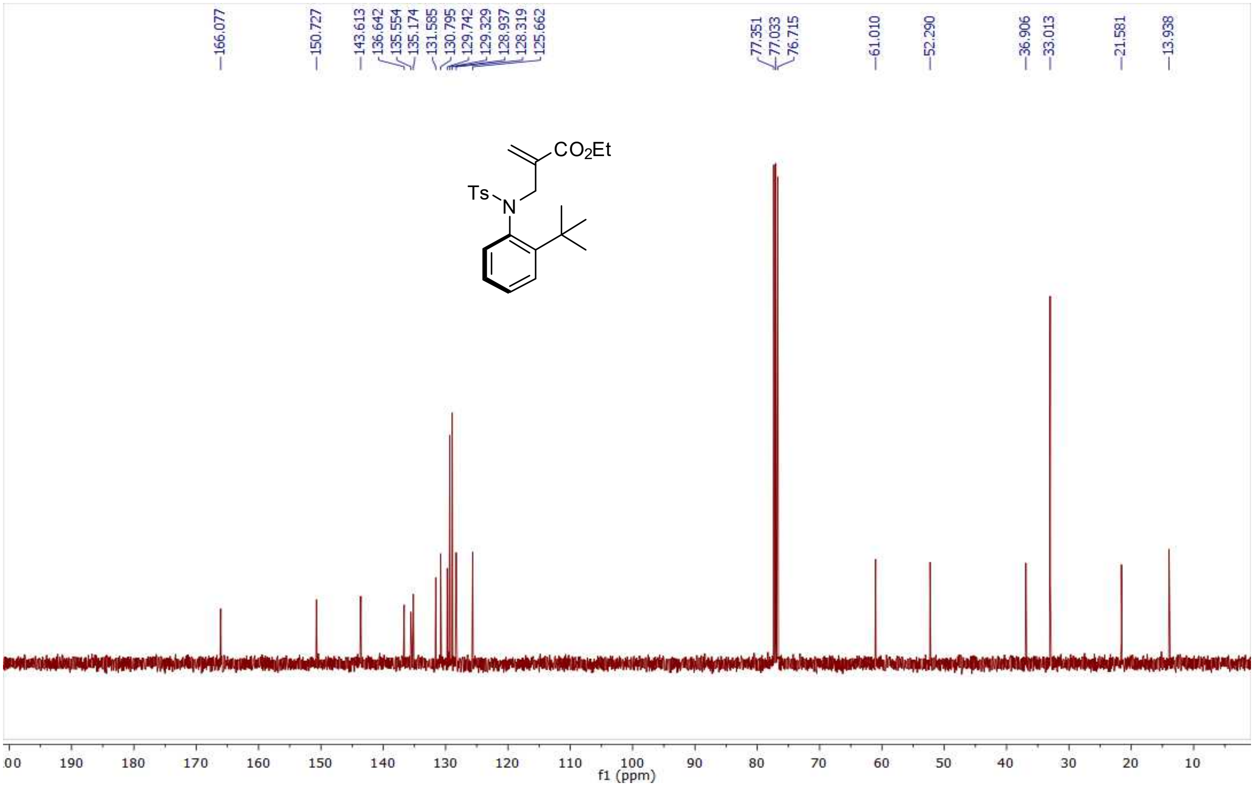

Supplementary Figure 111. ^{1}H and ^{13}C NMR Spectra of **9ba**.

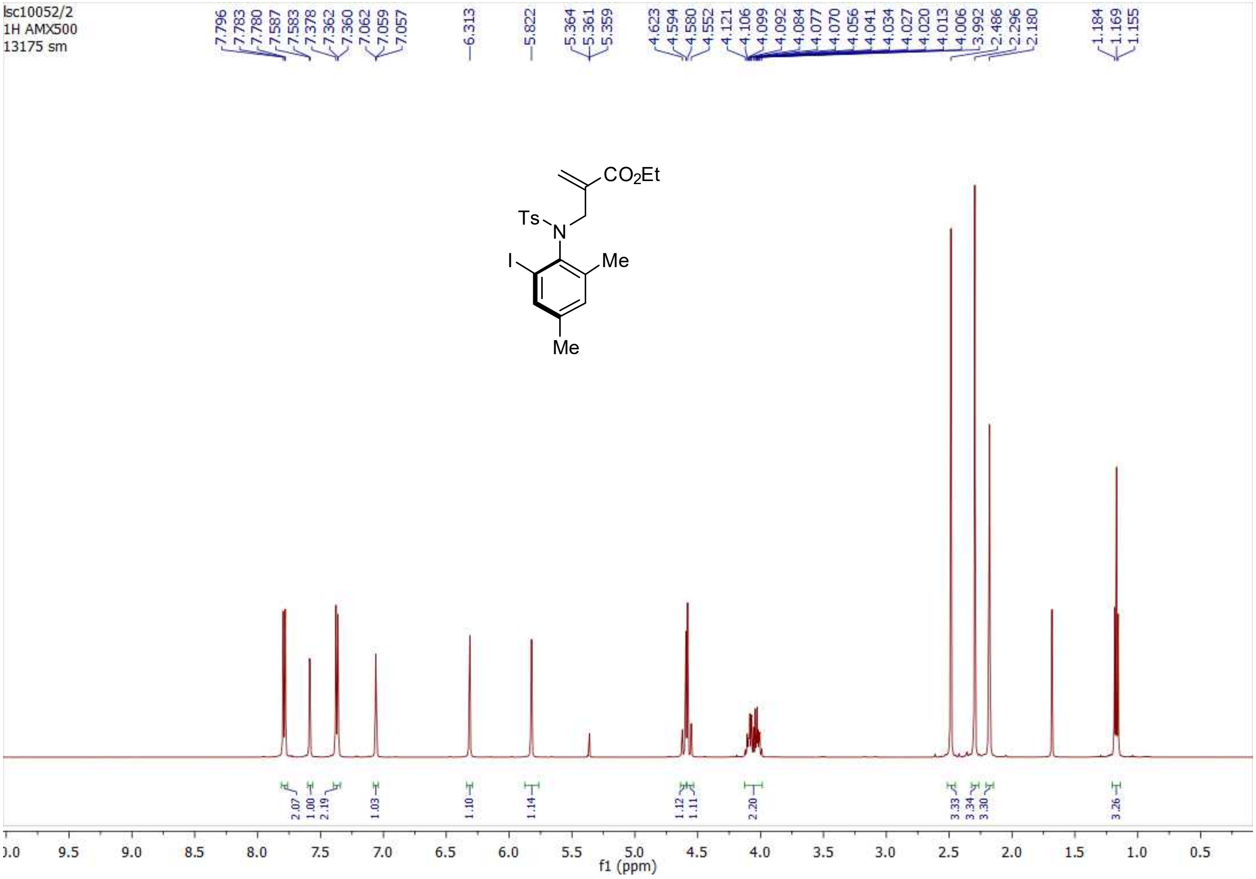

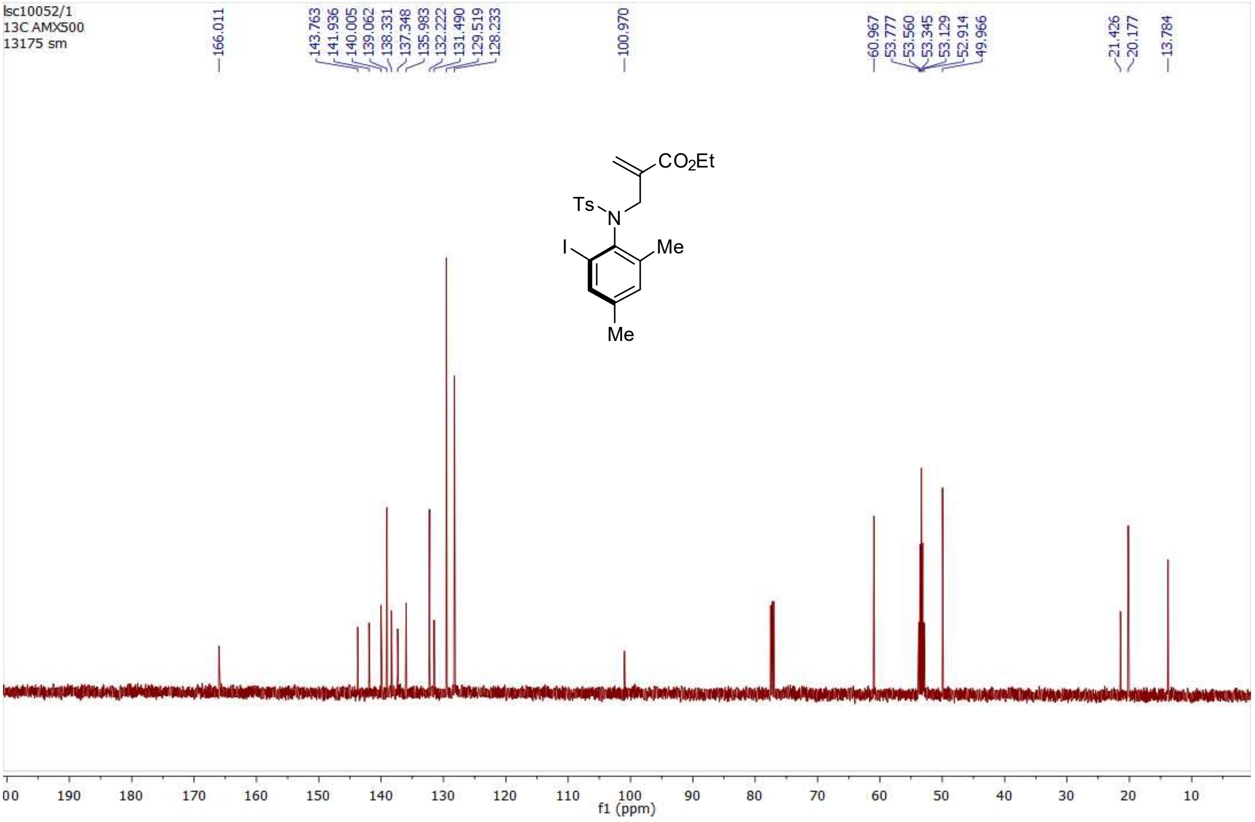

Supplementary Figure 112. ^{1}H and ^{13}C NMR Spectra of **9bb**.

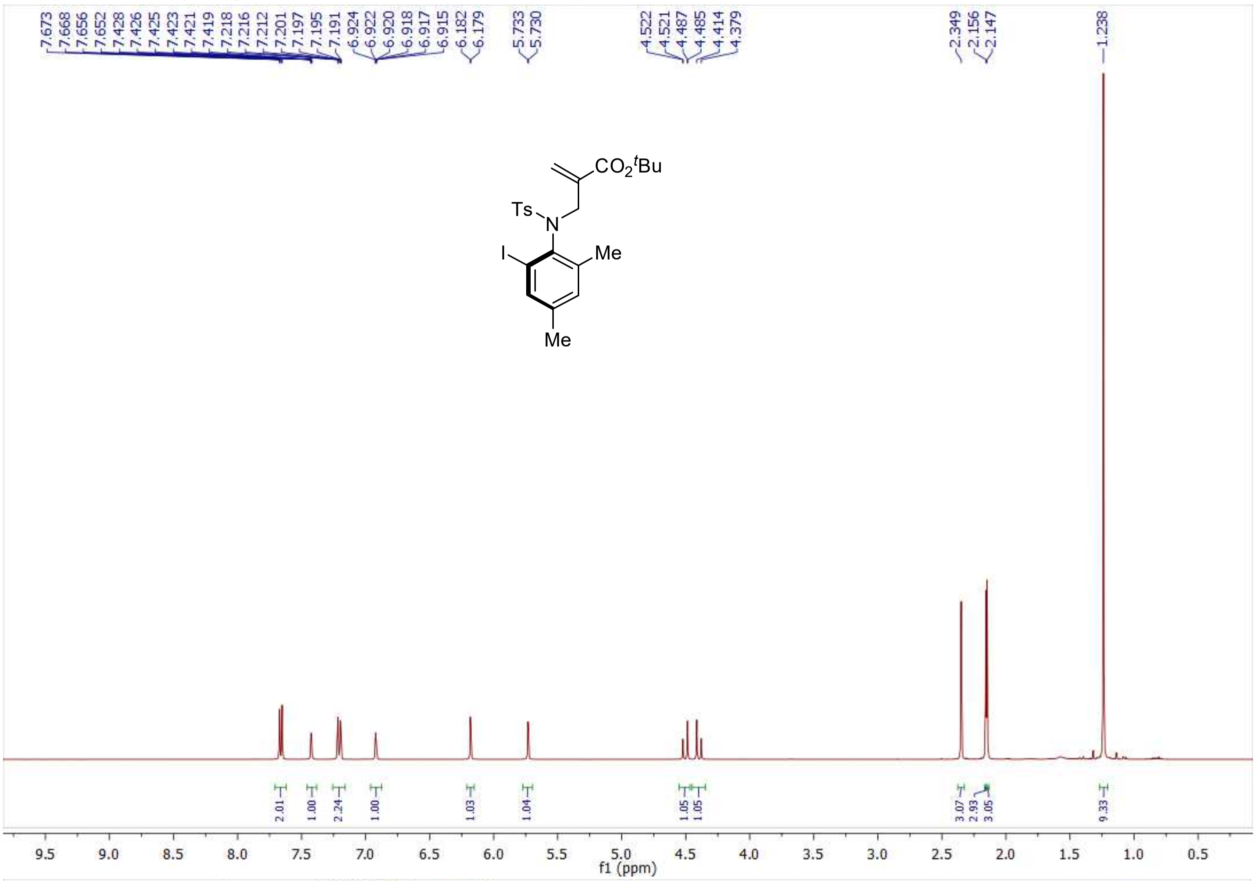

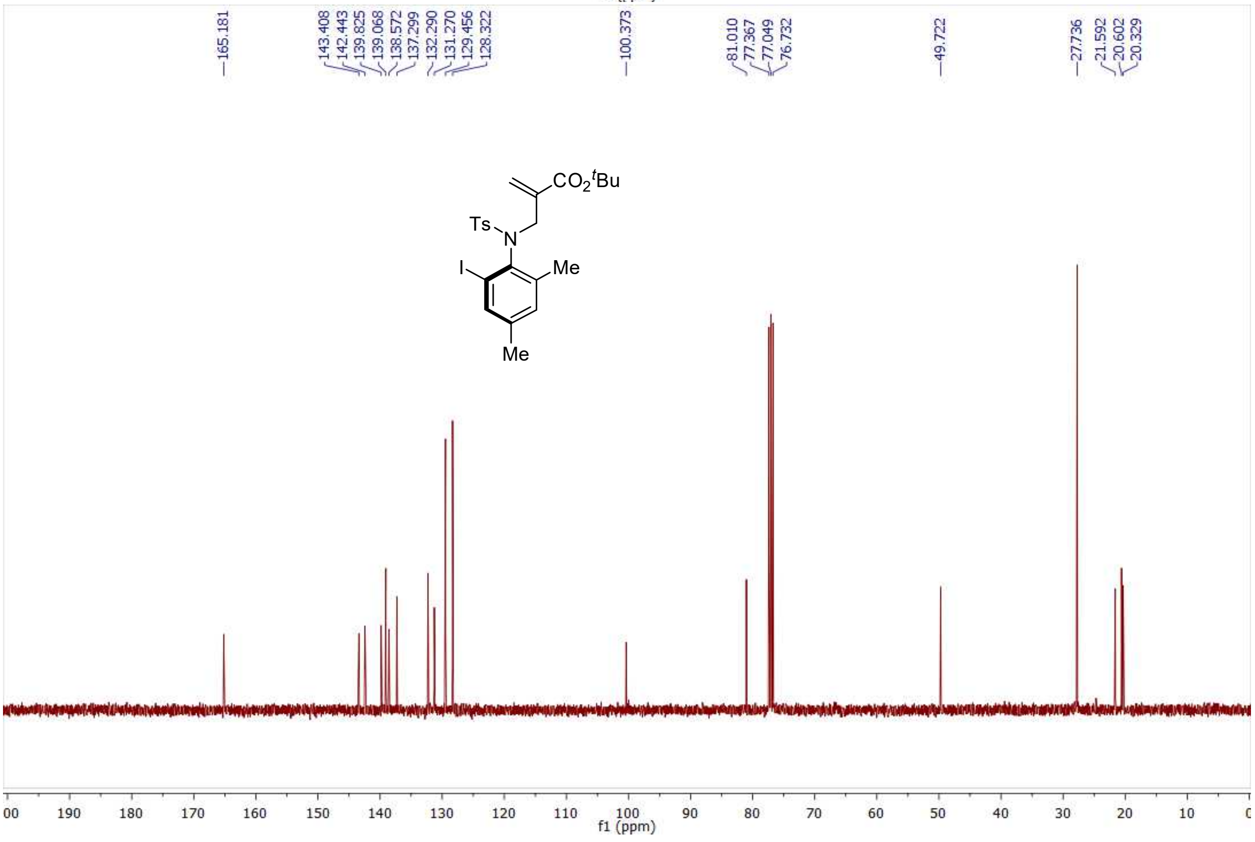

Supplementary Figure 113. ^{1}H and ^{13}C NMR Spectra of **9bc**.

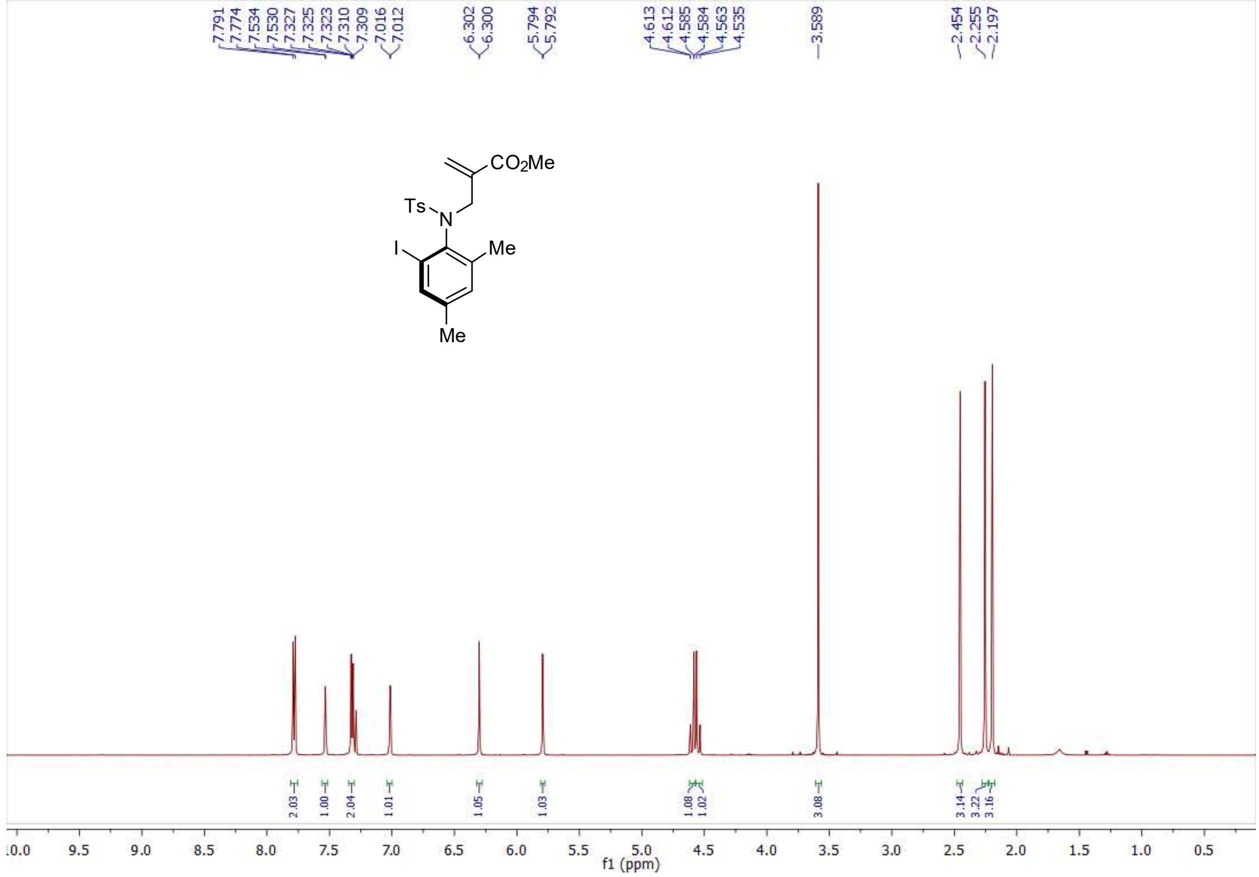

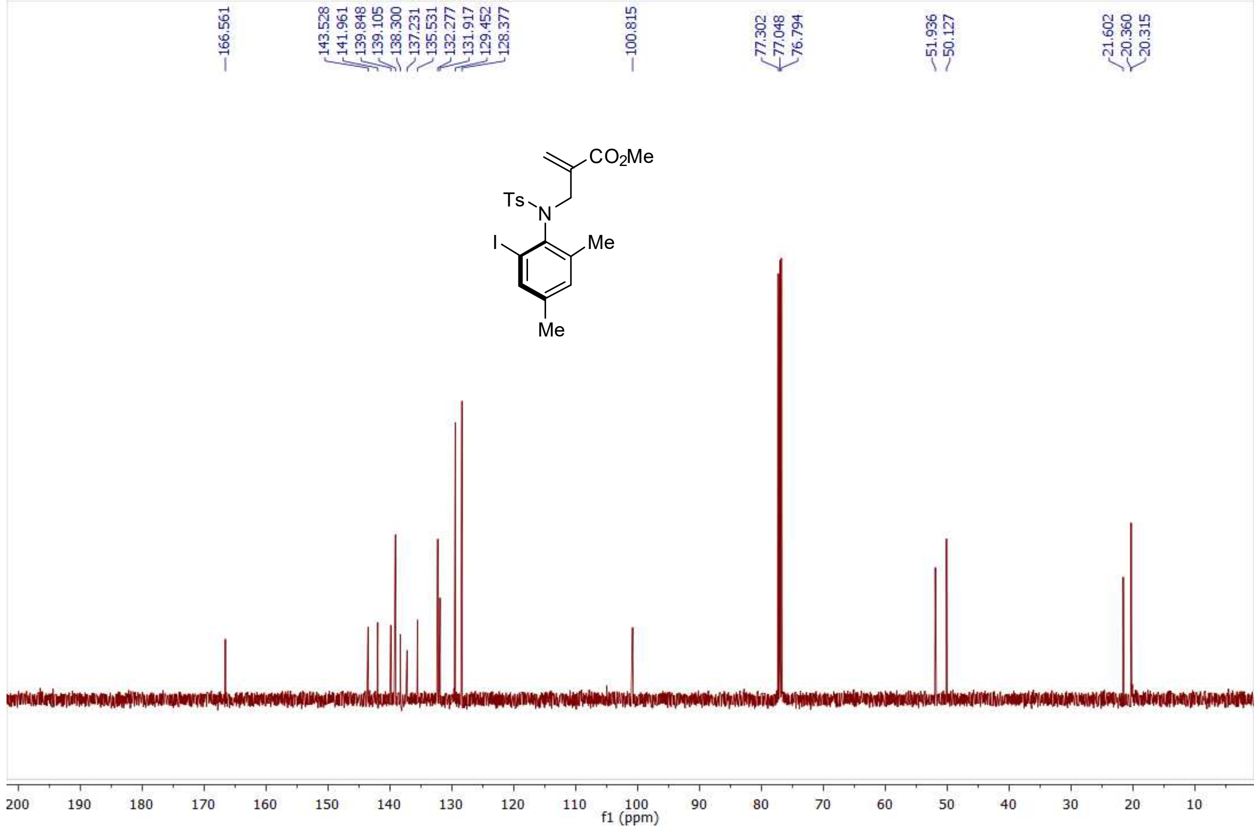

Supplementary Figure 114. ^{1}H and ^{13}C NMR Spectra of **9bd**.

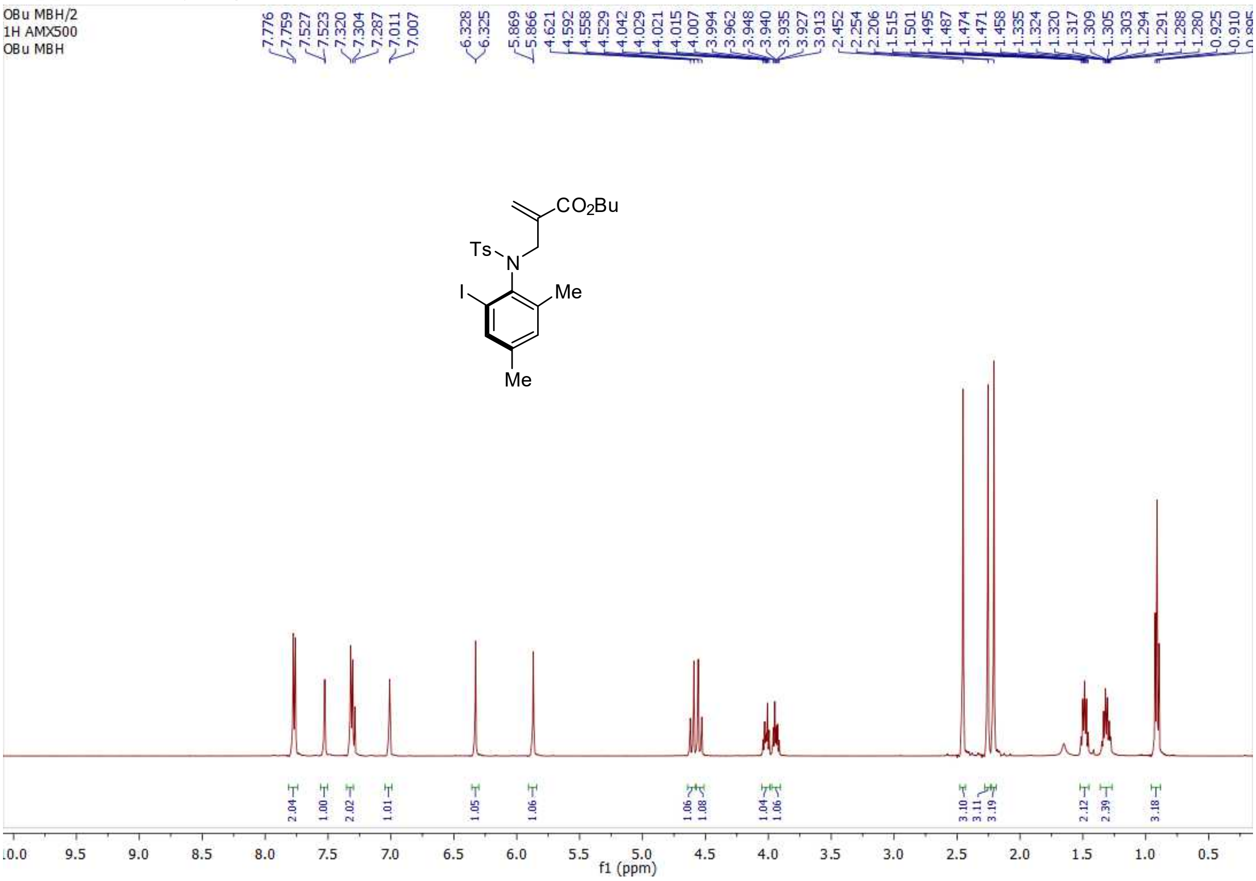

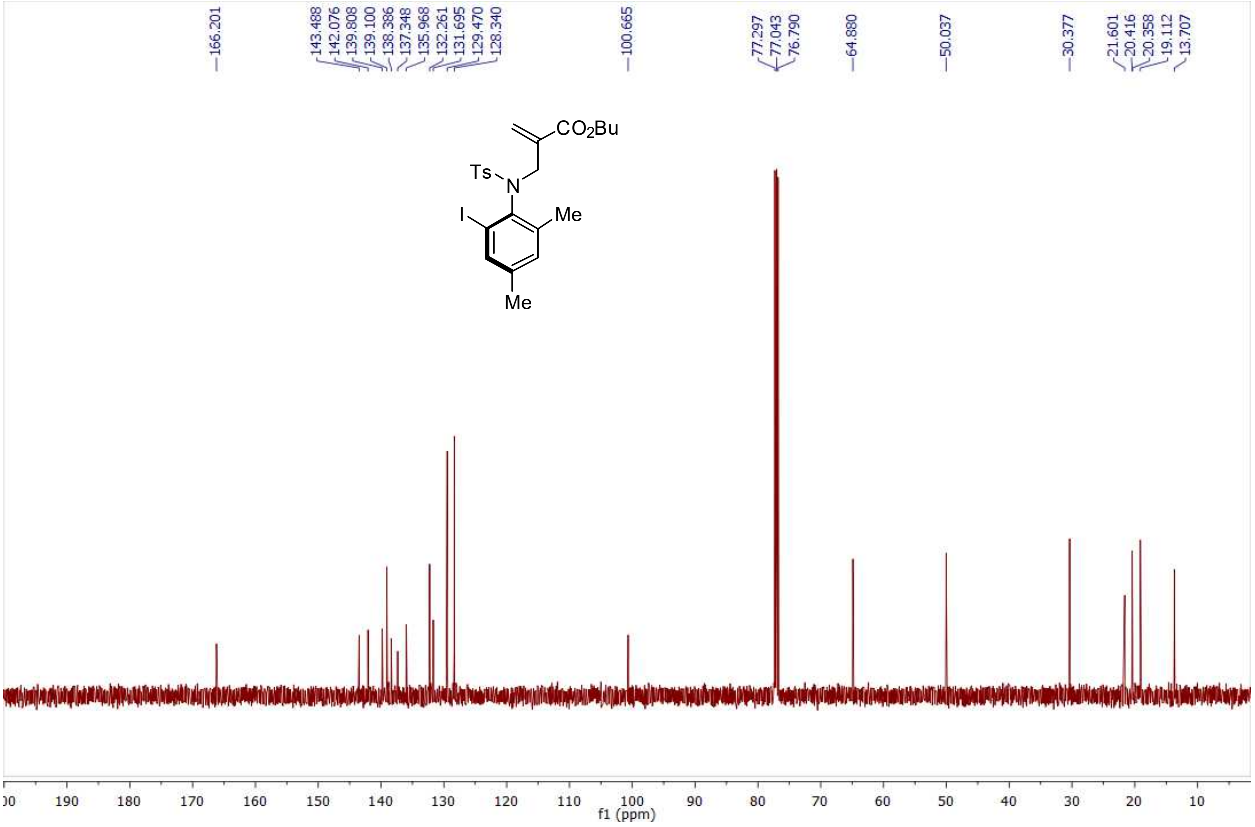

Supplementary Figure 115. ^{1}H and ^{13}C NMR Spectra of **9be**.

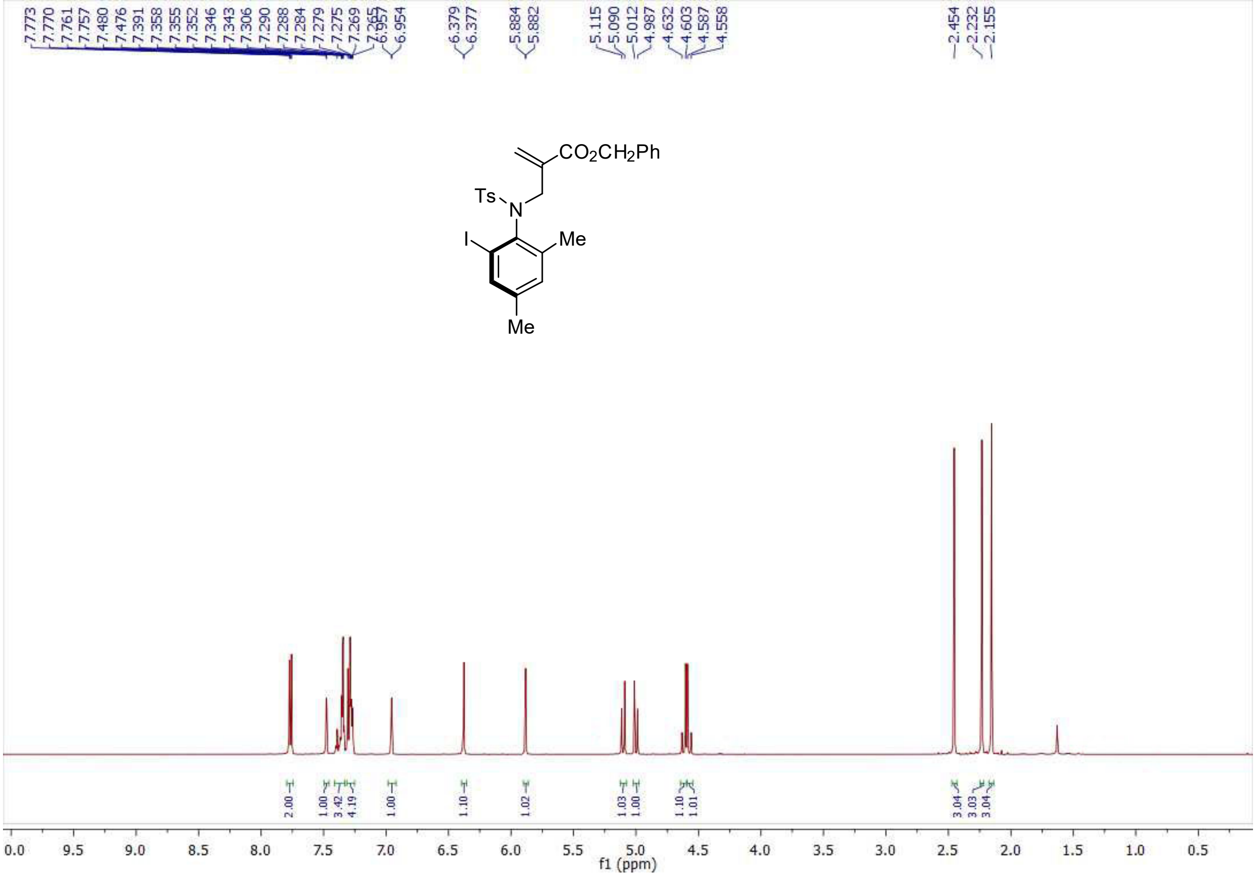

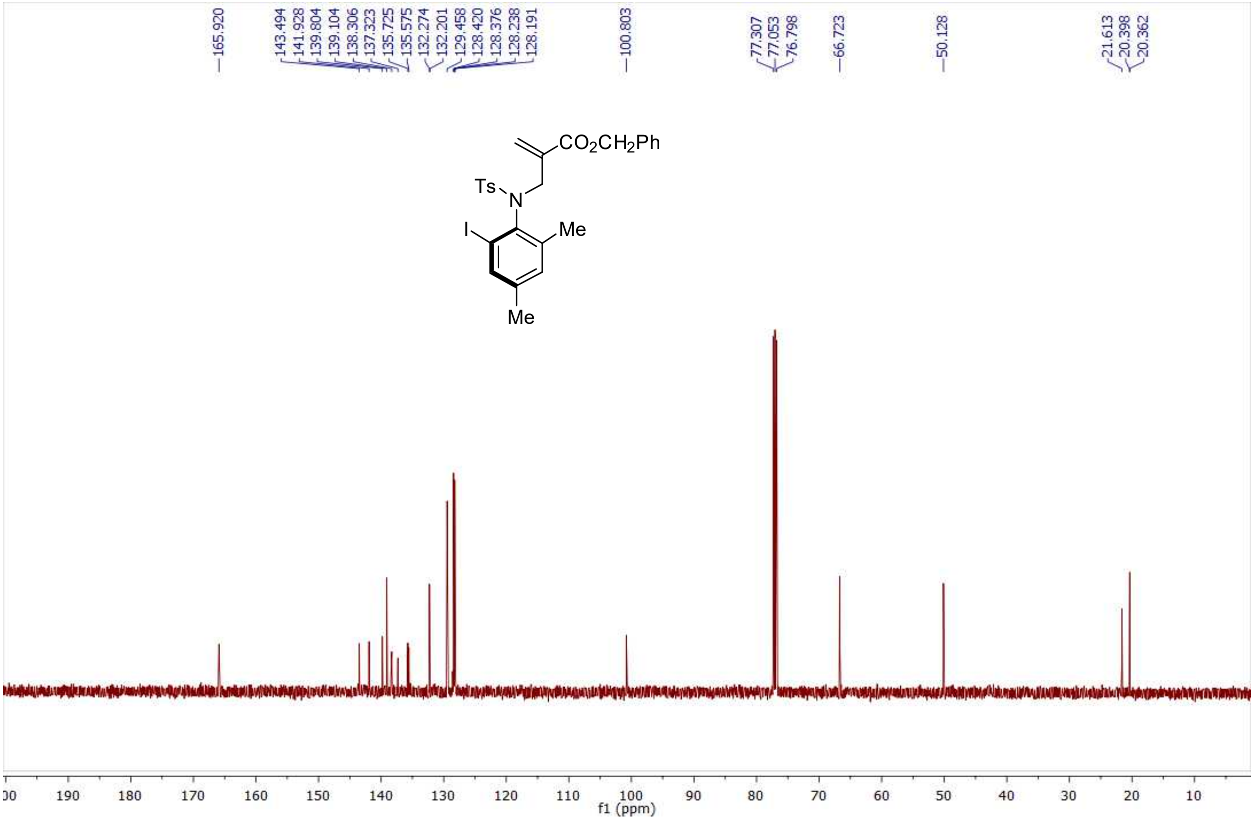

Supplementary Figure 116. ^{1}H and ^{13}C NMR Spectra of **9ca**.

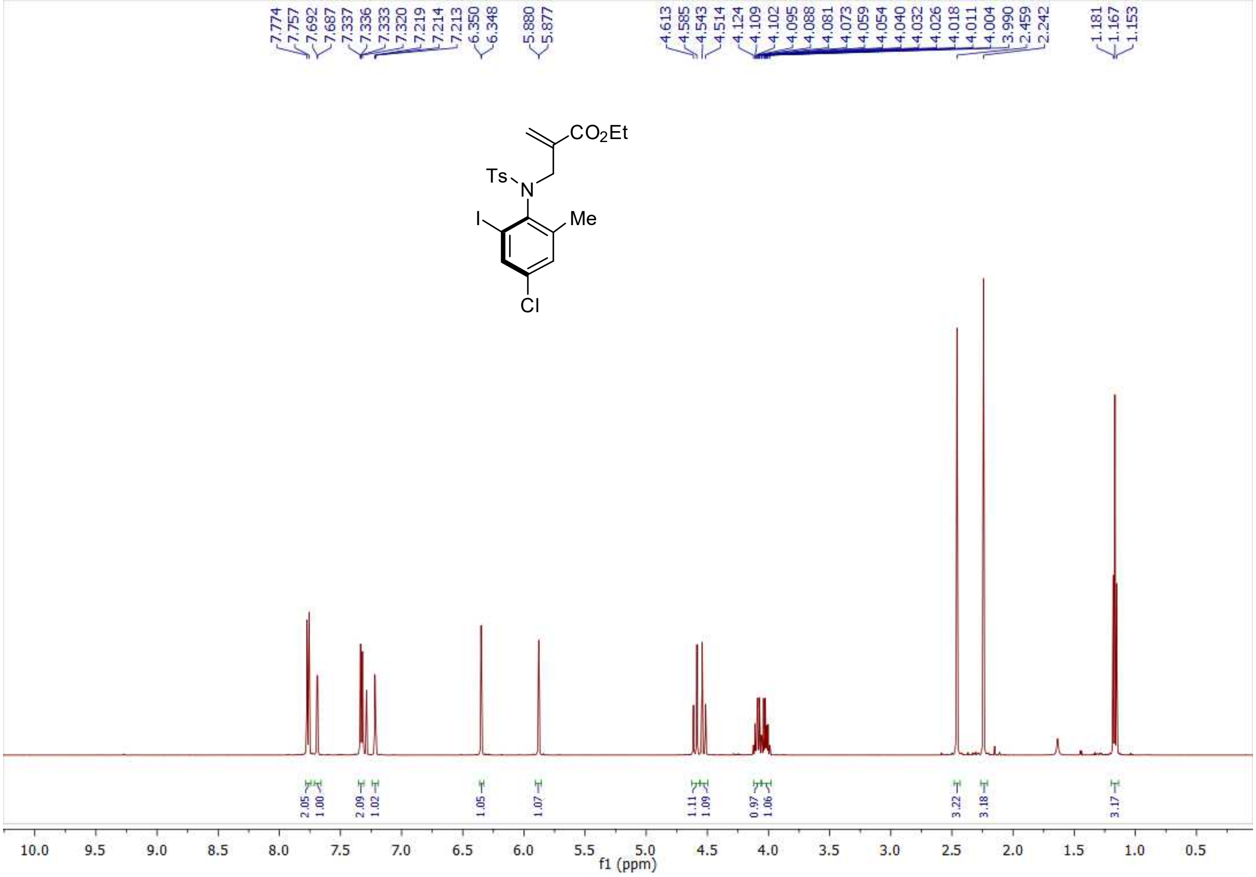

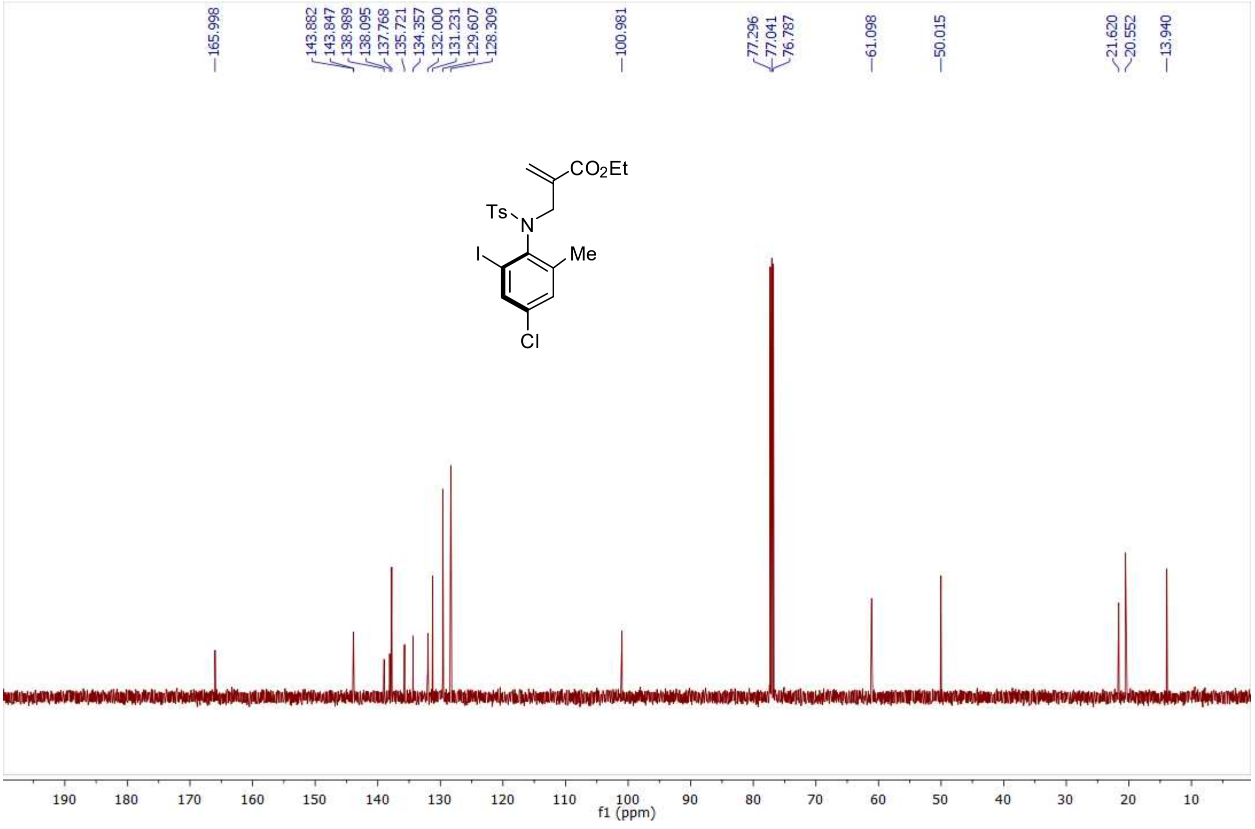

Supplementary Figure 117. ^{1}H and ^{13}C NMR Spectra of **9da**.

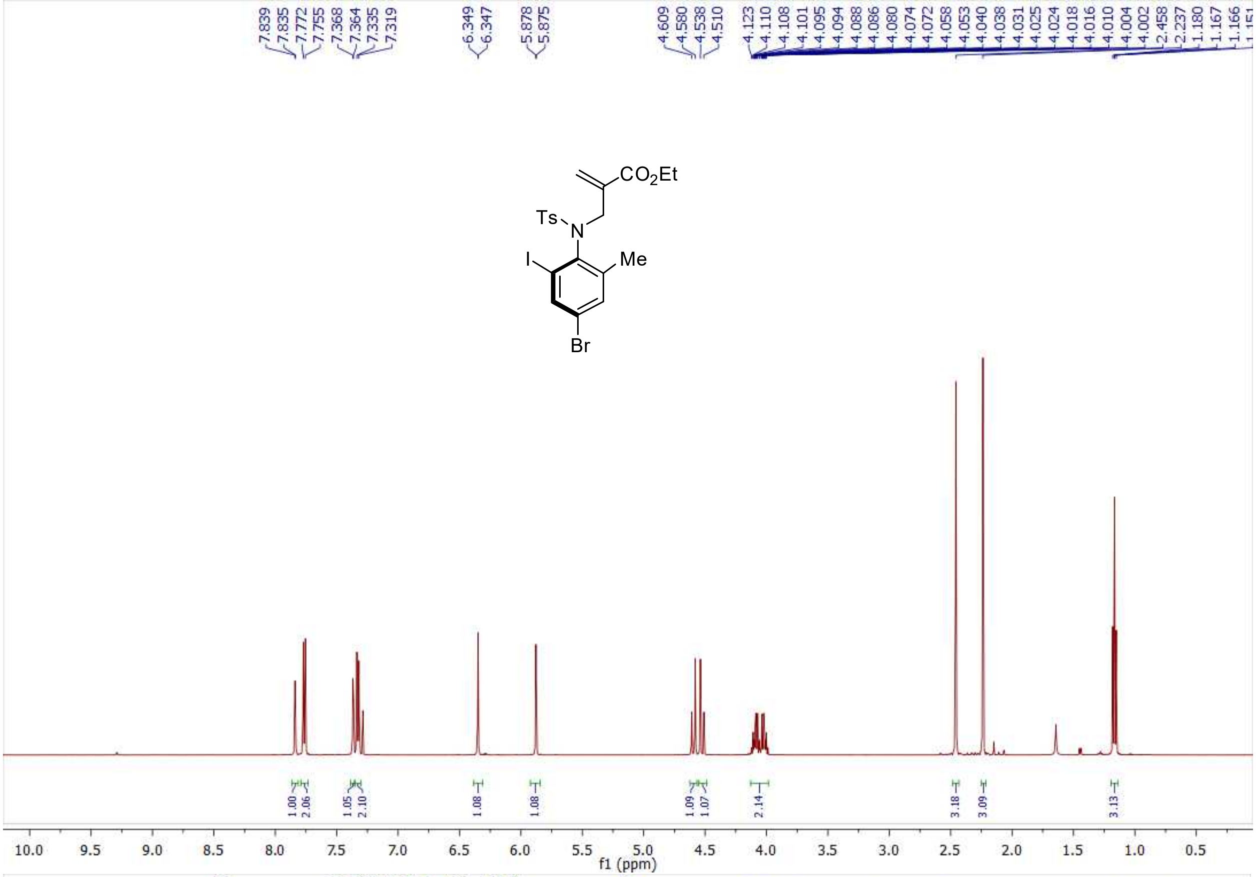

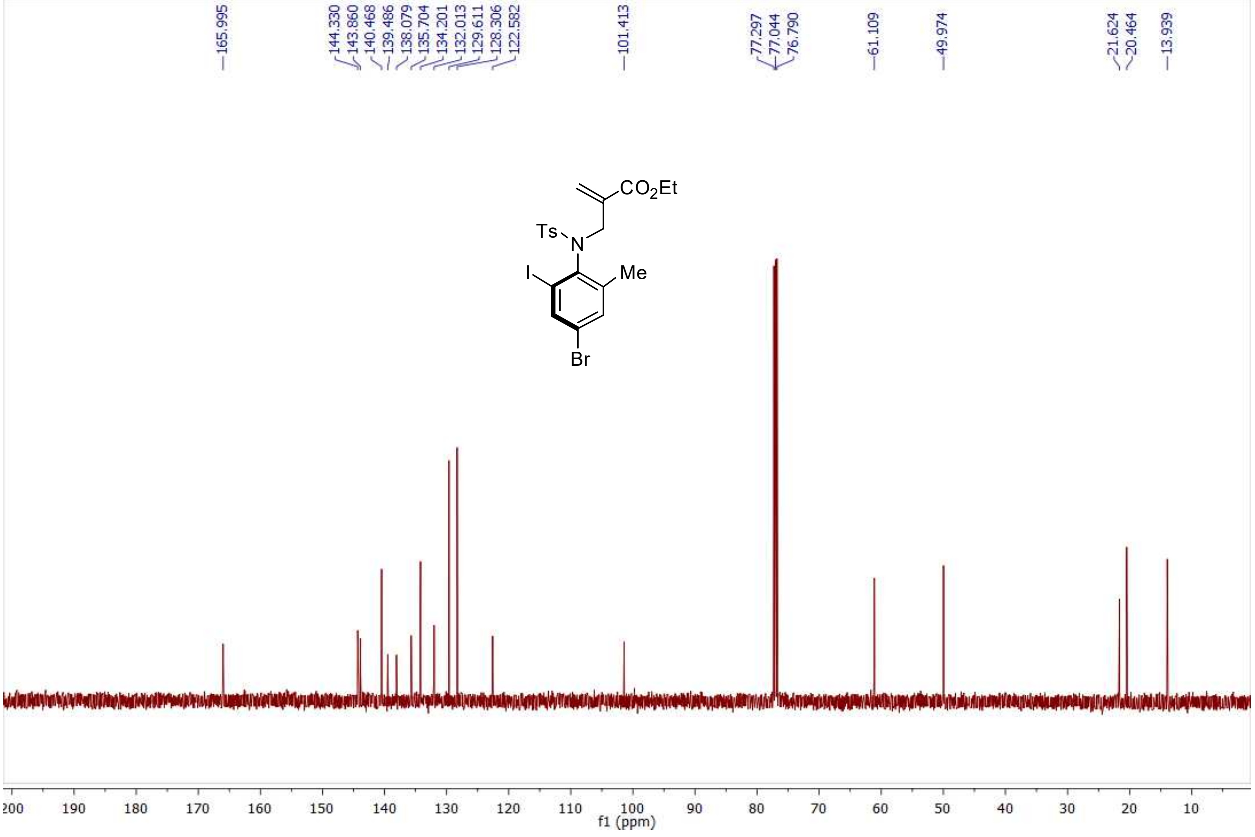

Supplementary Figure 118. ^{1}H and ^{13}C NMR Spectra of **9ea**.

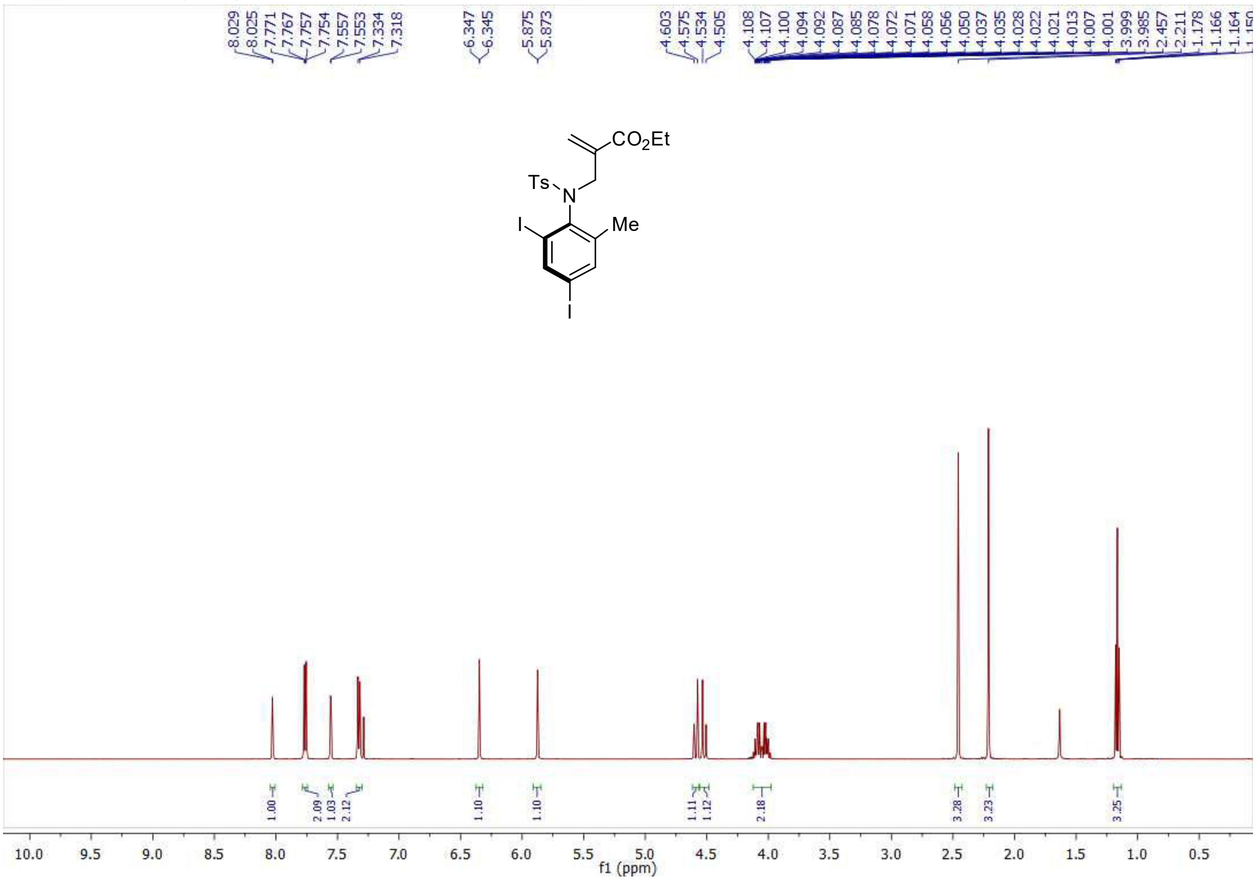

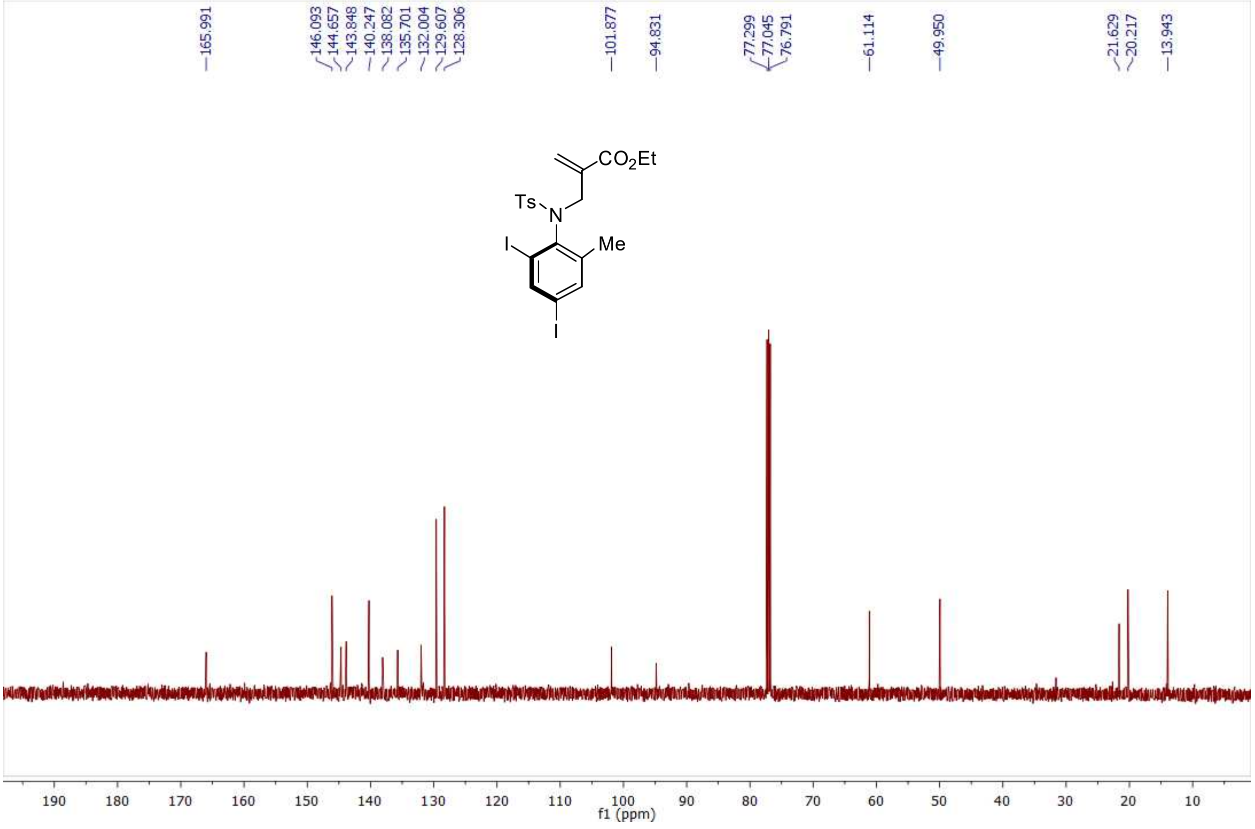

Supplementary Figure 119. ^{1}H and ^{13}C NMR Spectra of **9fa**.

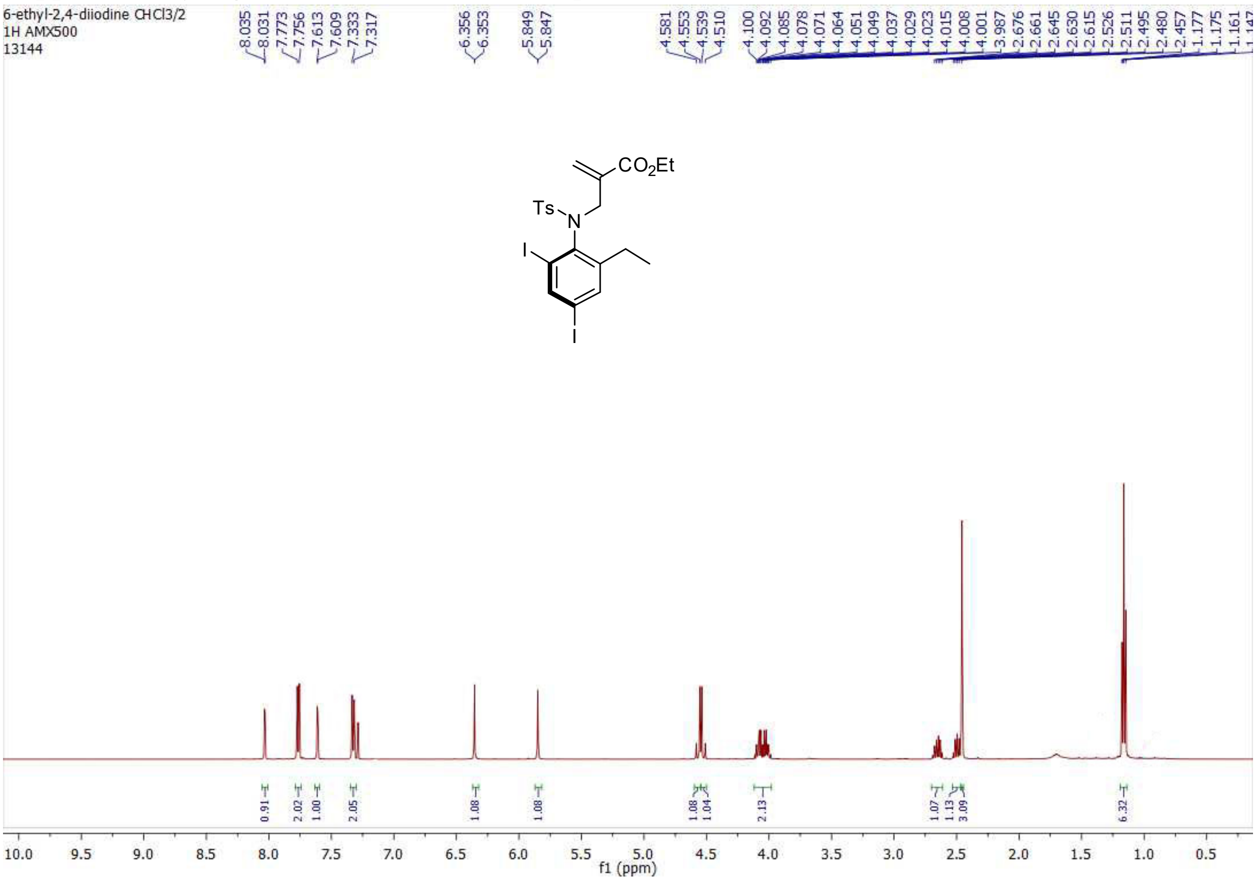

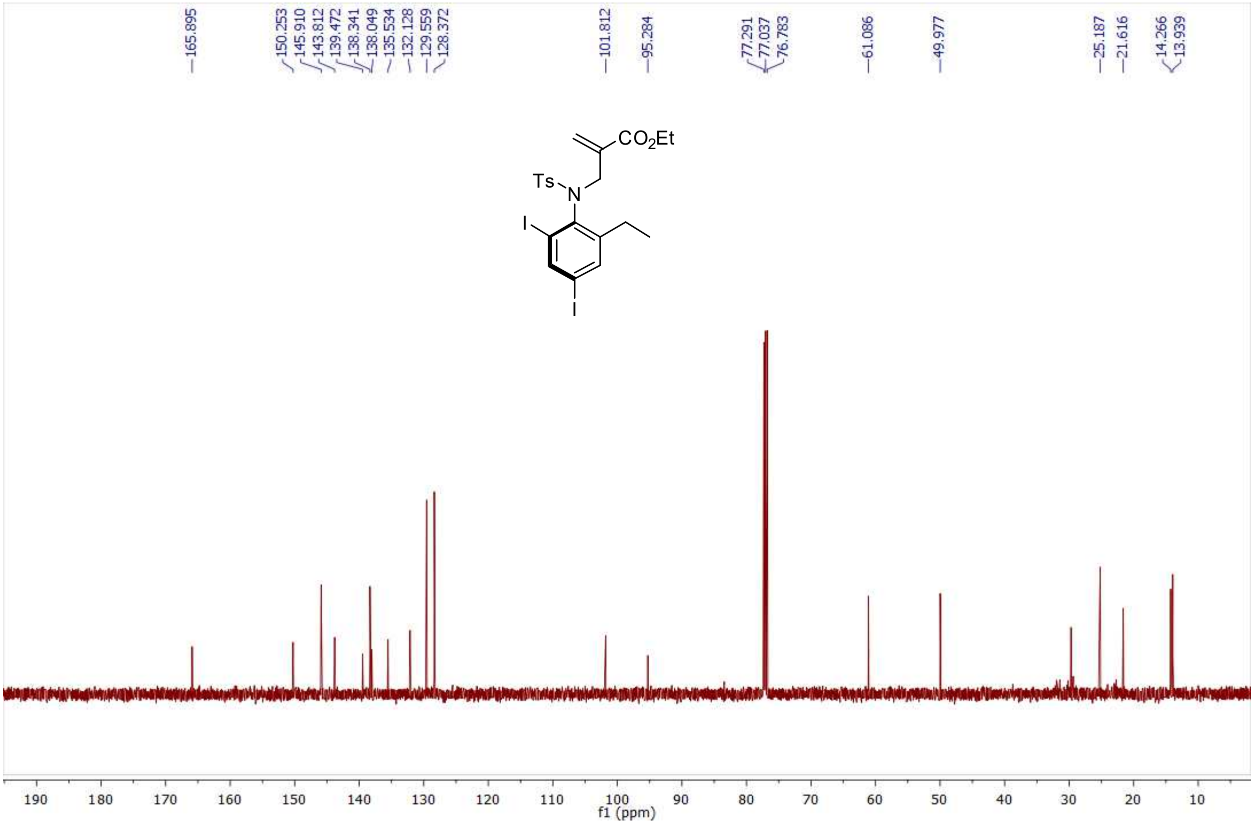

Supplementary Figure 120. ^{1}H and ^{13}C NMR Spectra of **9ga**.

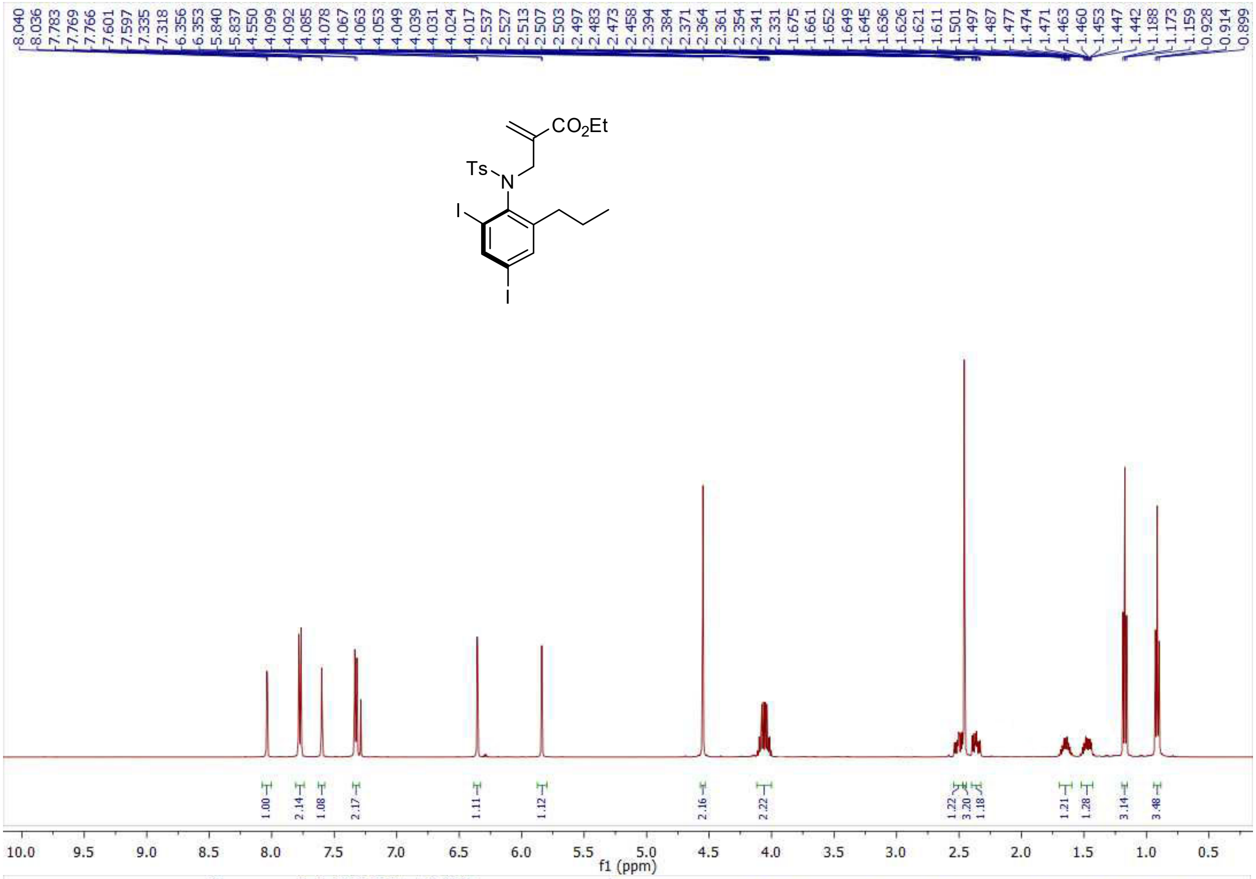

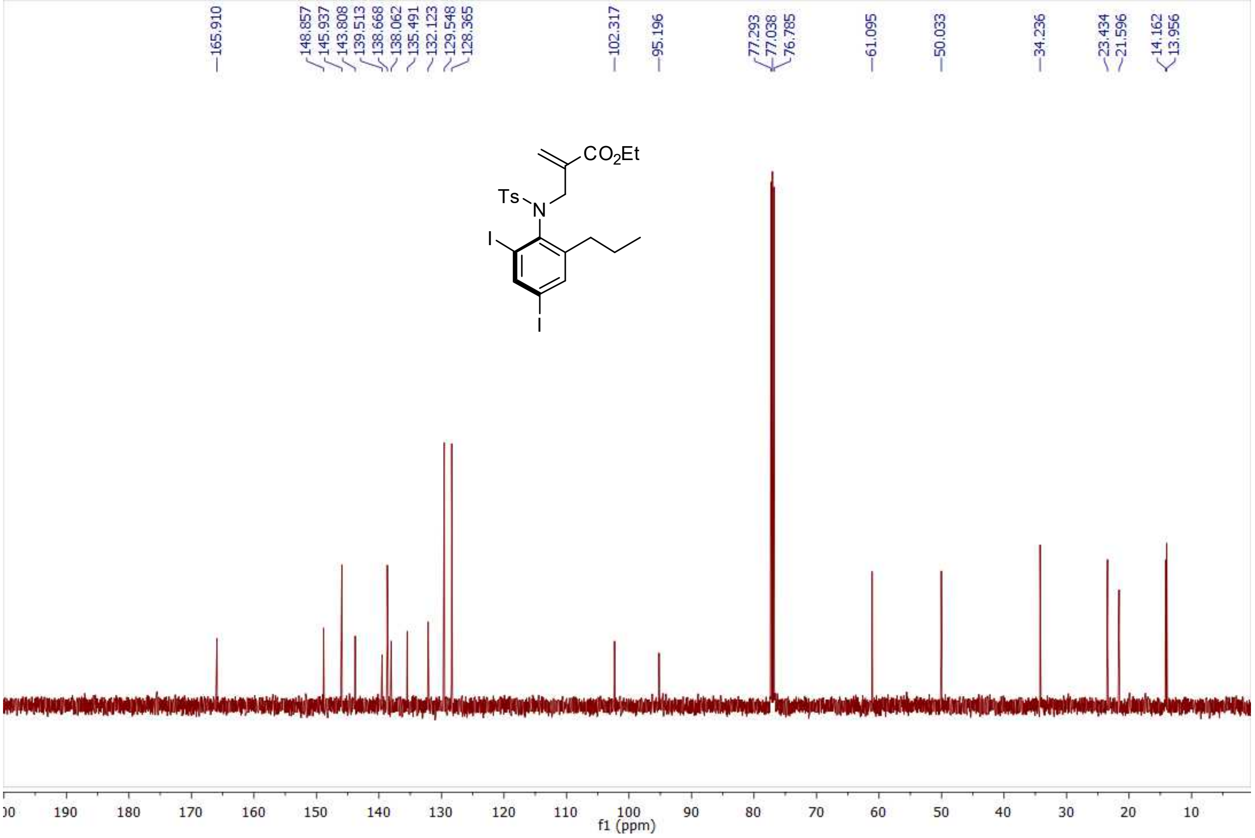

Supplementary Figure 121. ^{1}H and ^{13}C NMR Spectra of **9ha**.

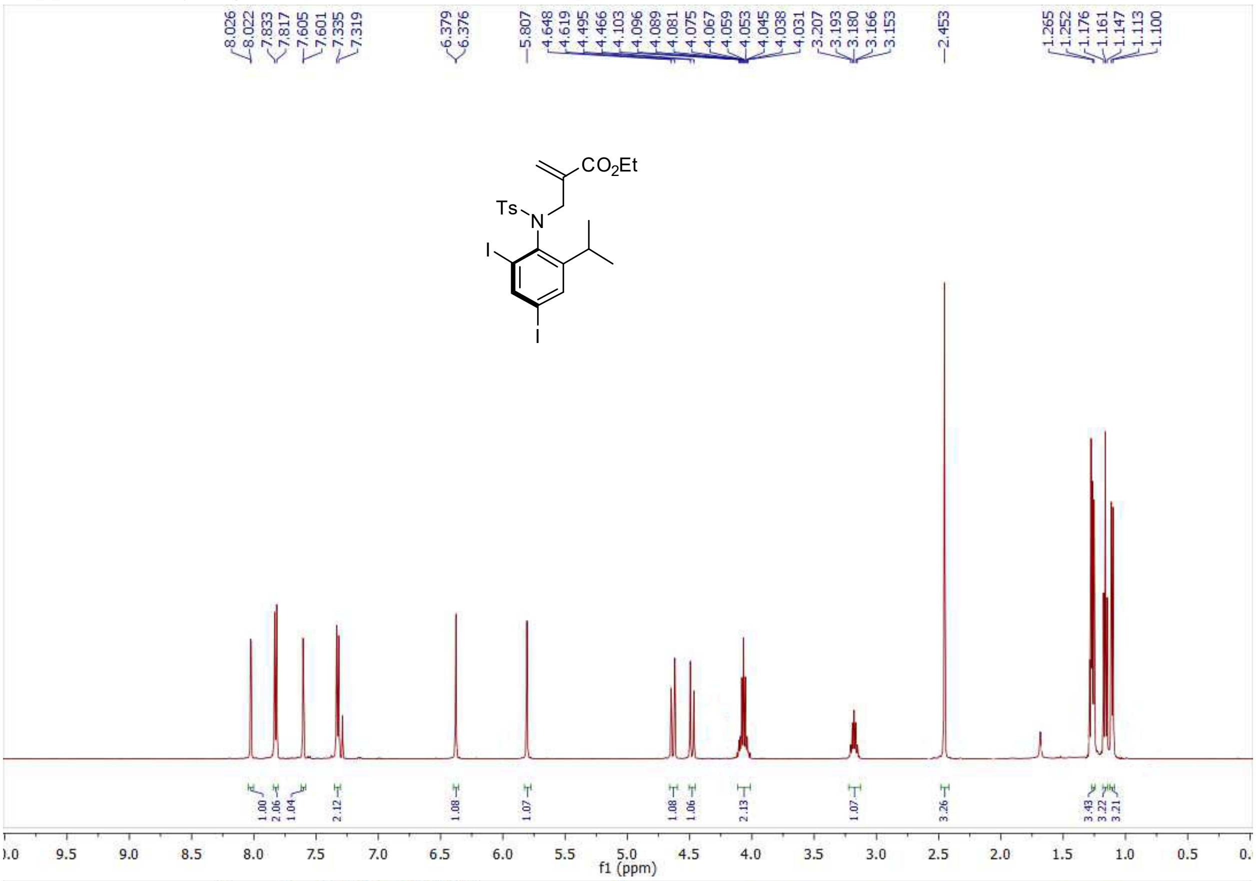

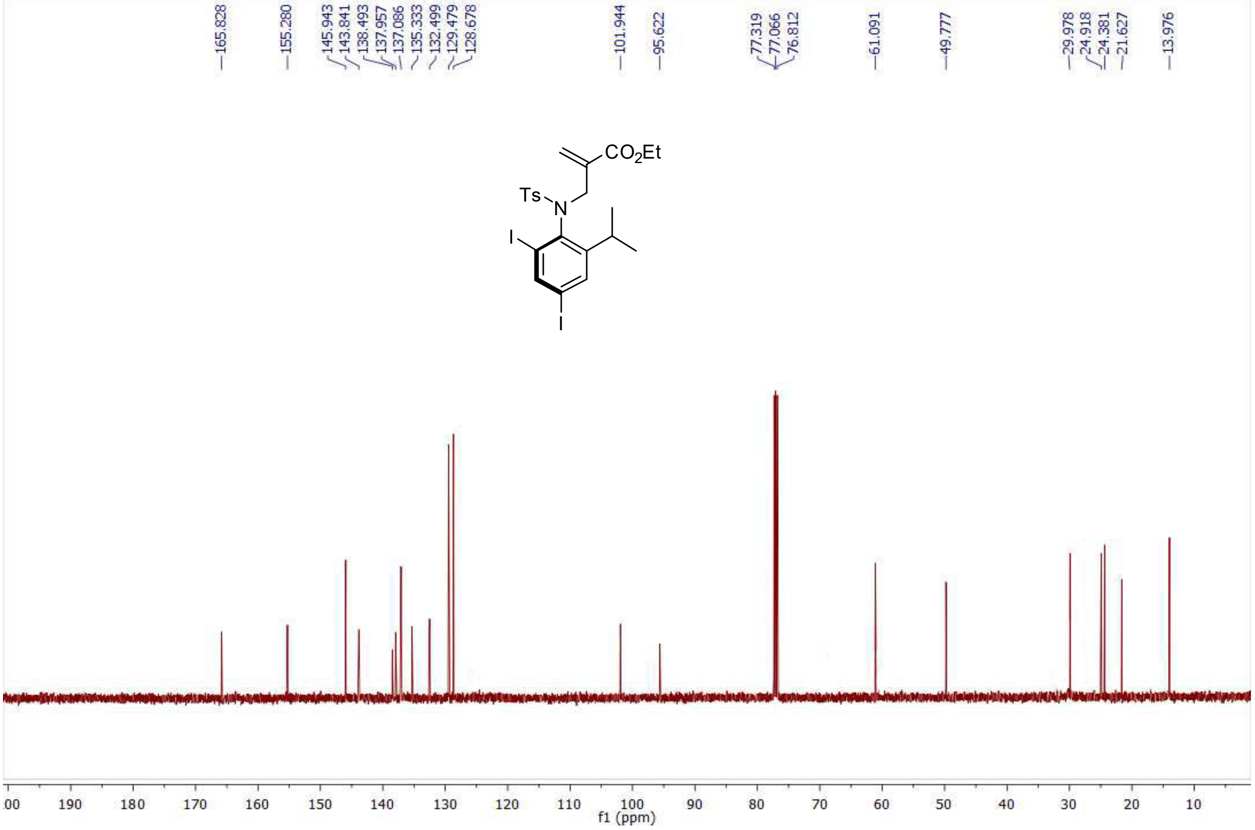

Supplementary Figure 122. ^{1}H and ^{13}C NMR Spectra of **9ia**.

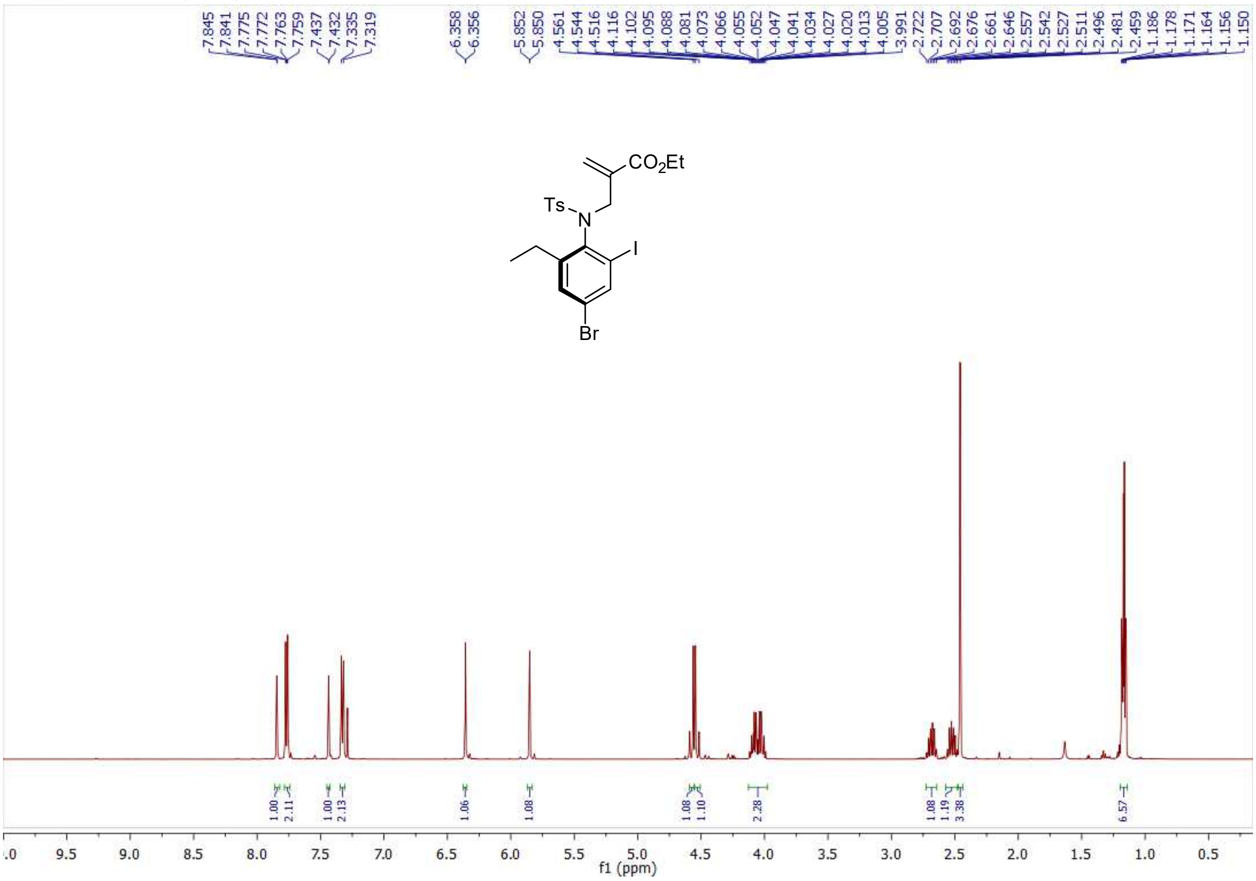

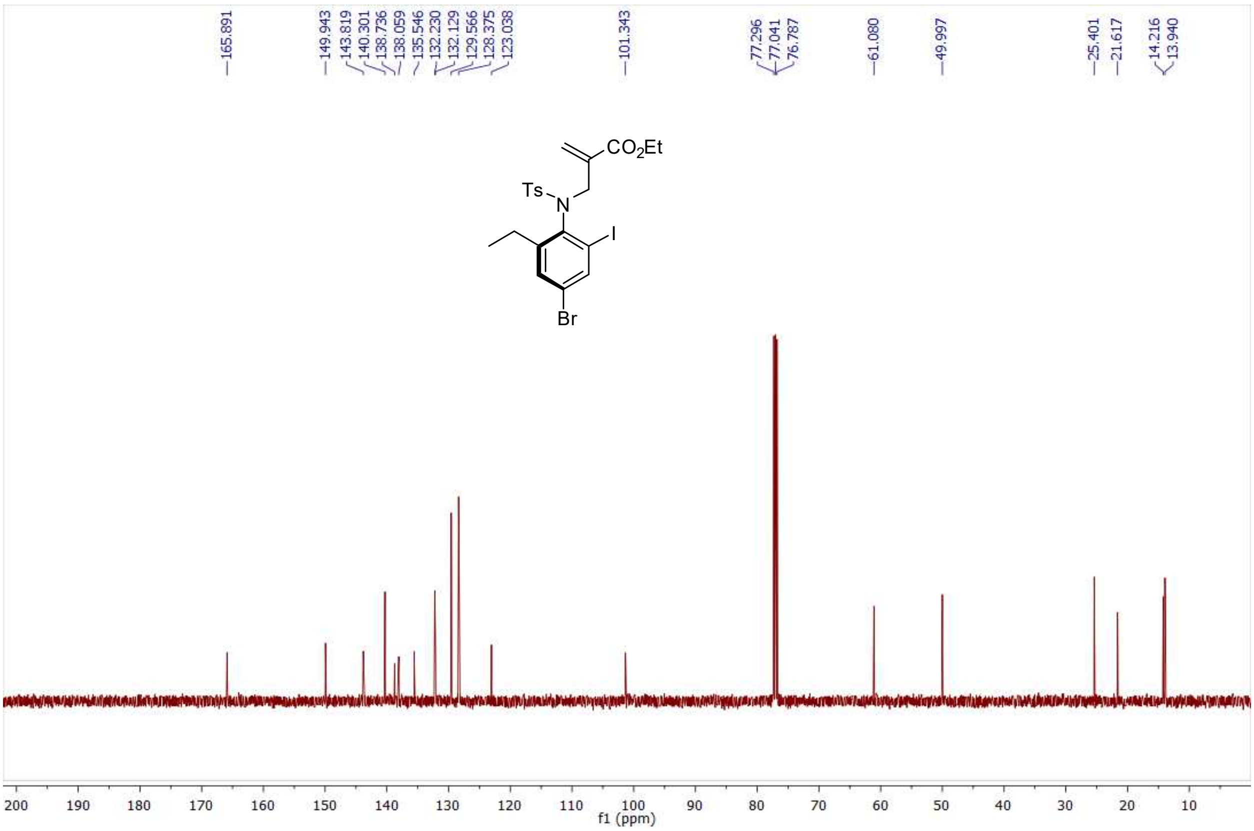

Supplementary Figure 123. ^{1}H and ^{13}C NMR Spectra of **9ja**.

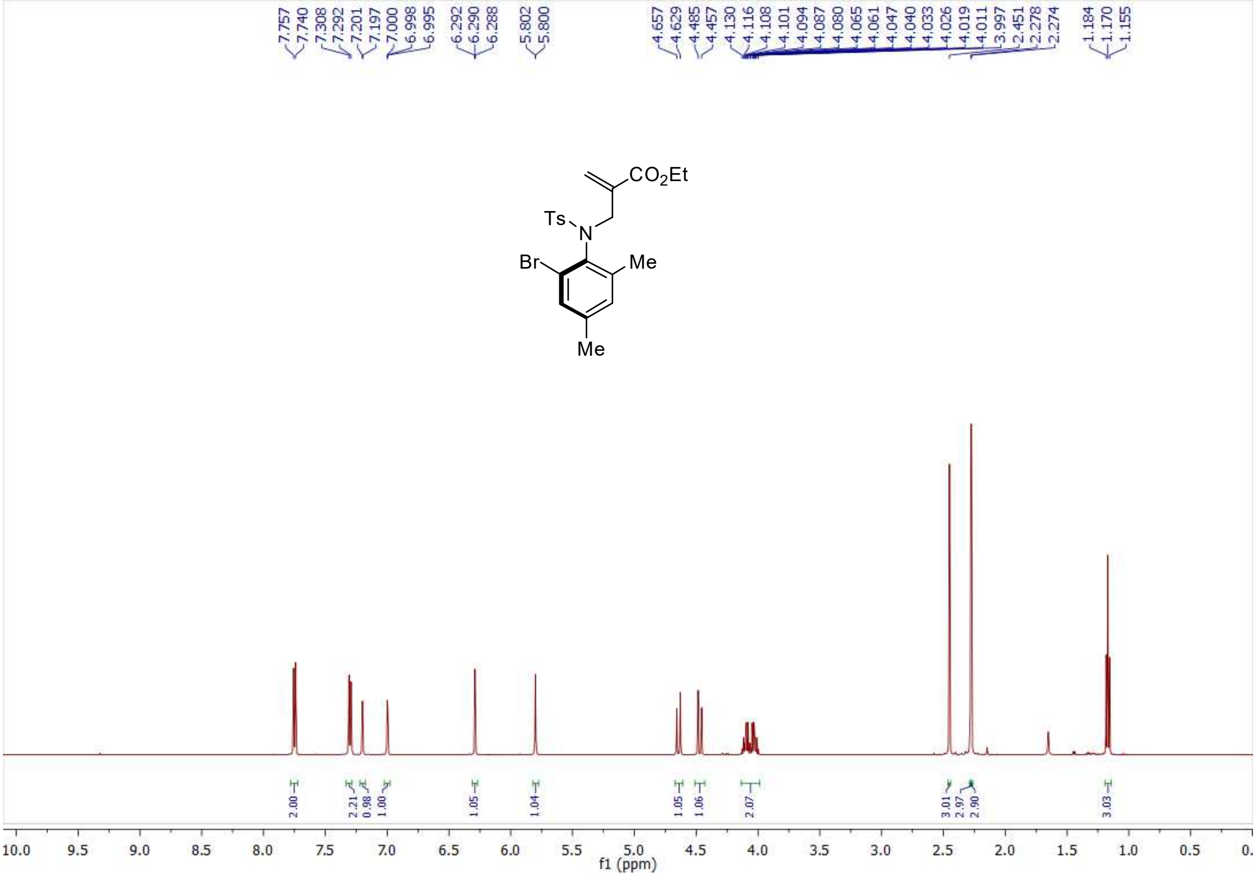

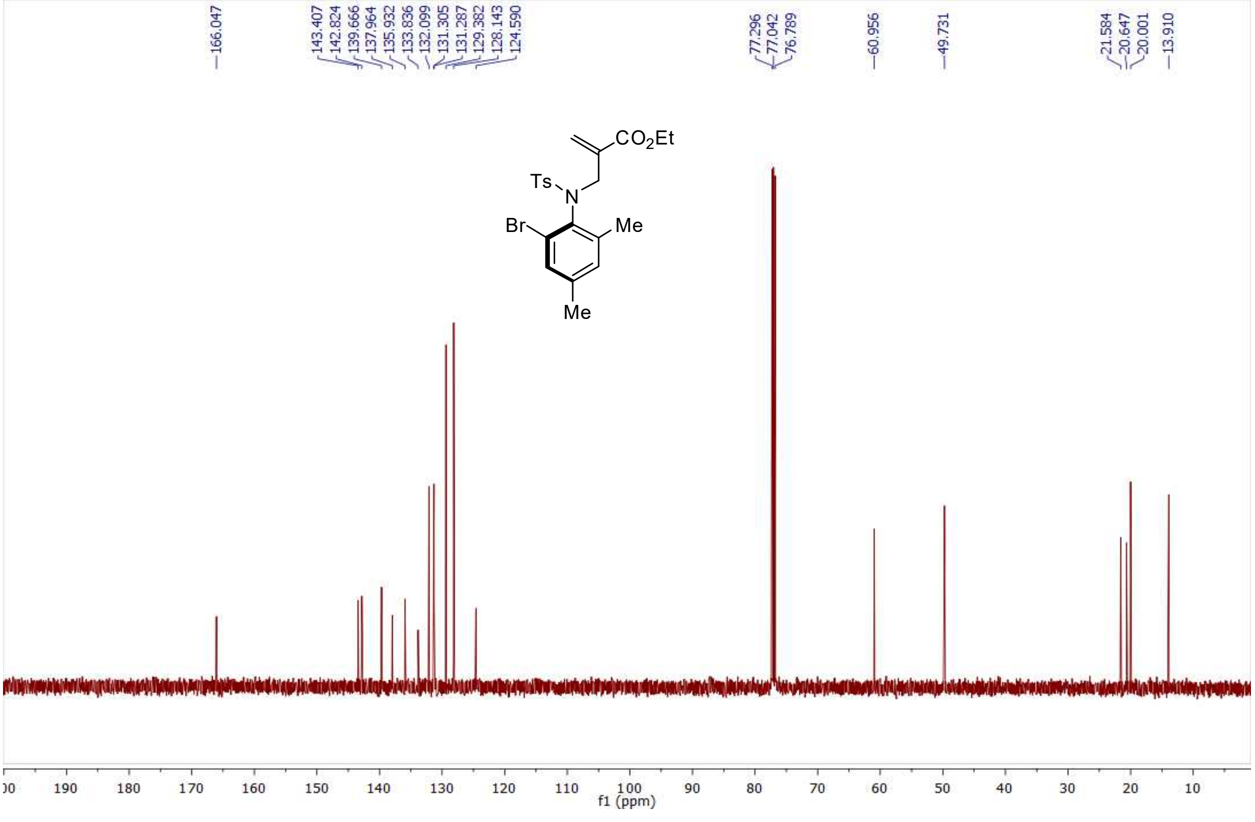

Supplementary Figure 124. ^{1}H and ^{13}C NMR Spectra of **9ka**.

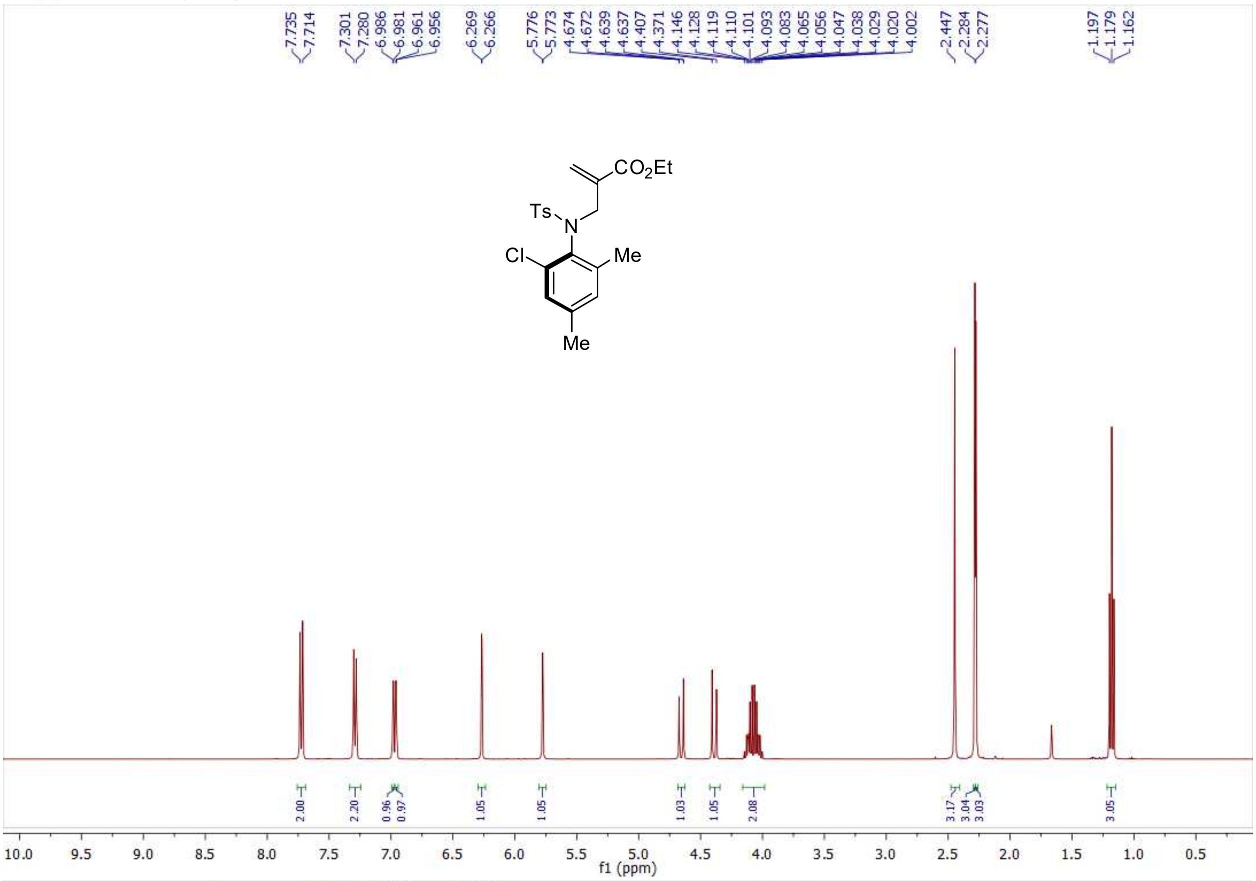

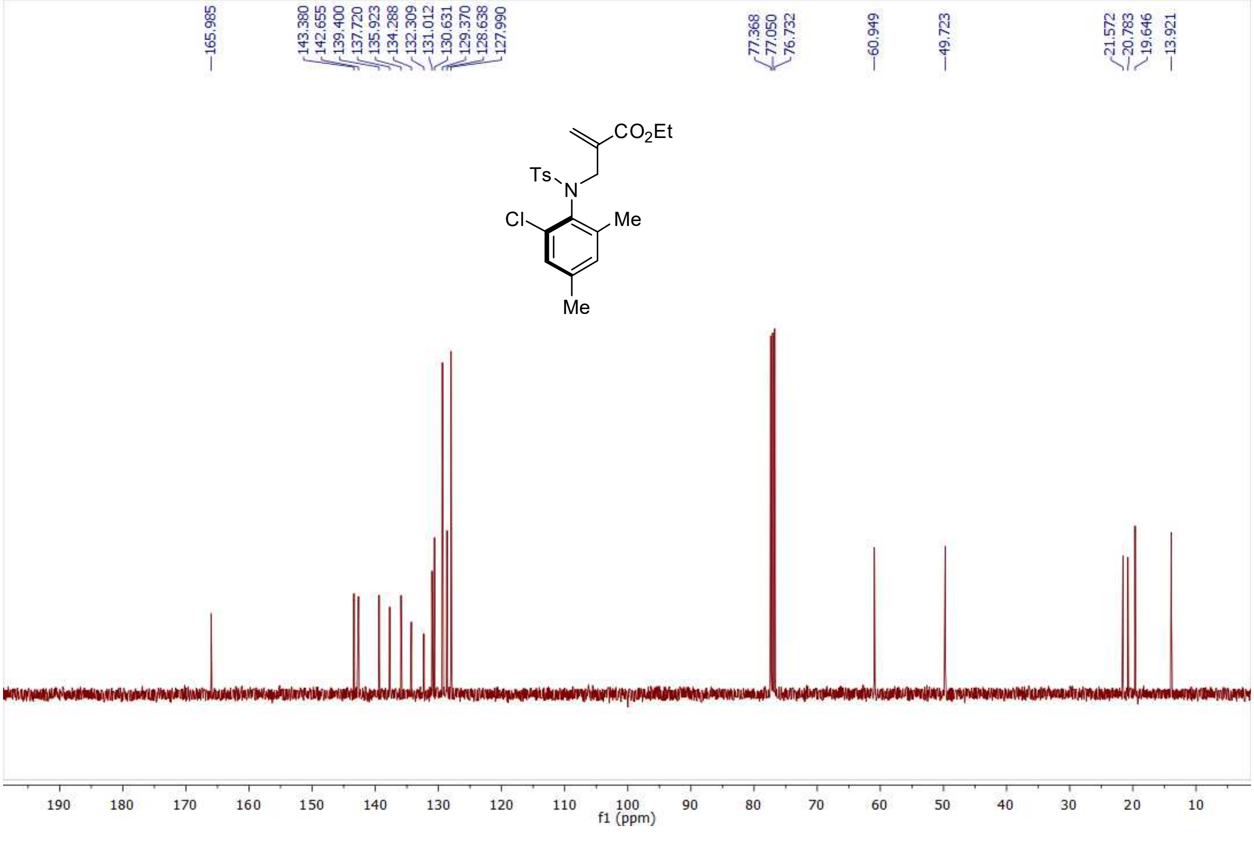

Supplementary Figure 125. ^1H and ^{13}C NMR Spectra of **9la**.

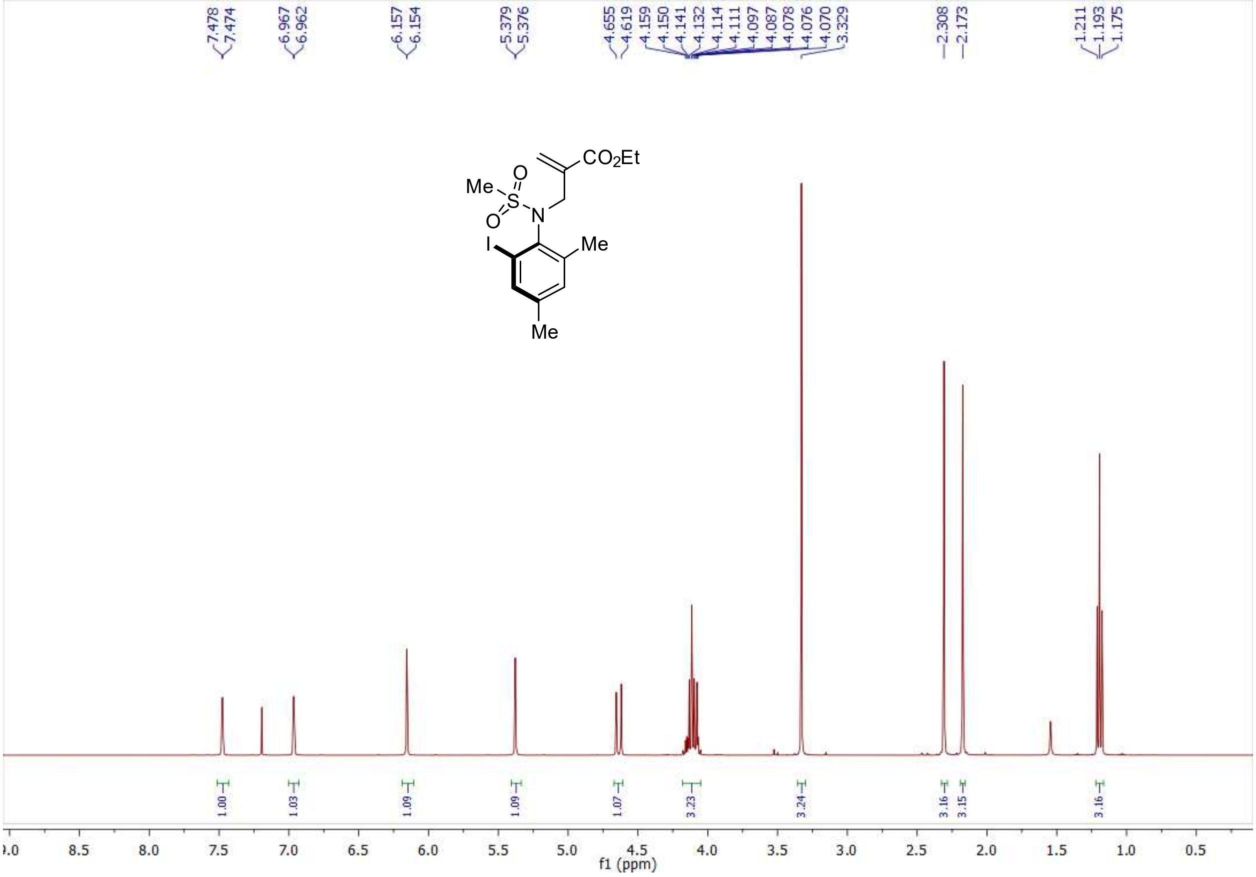

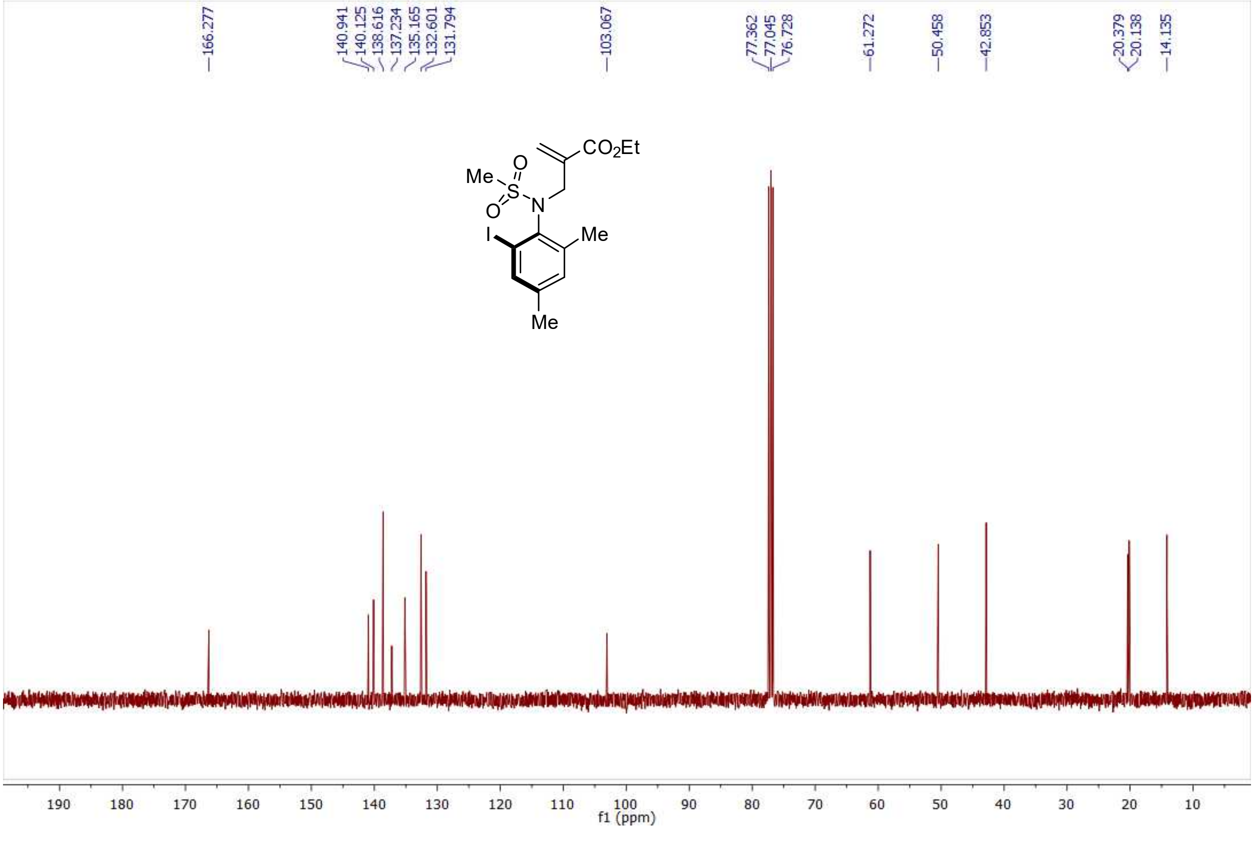

Supplementary Figure 126. ^{1}H and ^{13}C NMR Spectra of **9ma**.

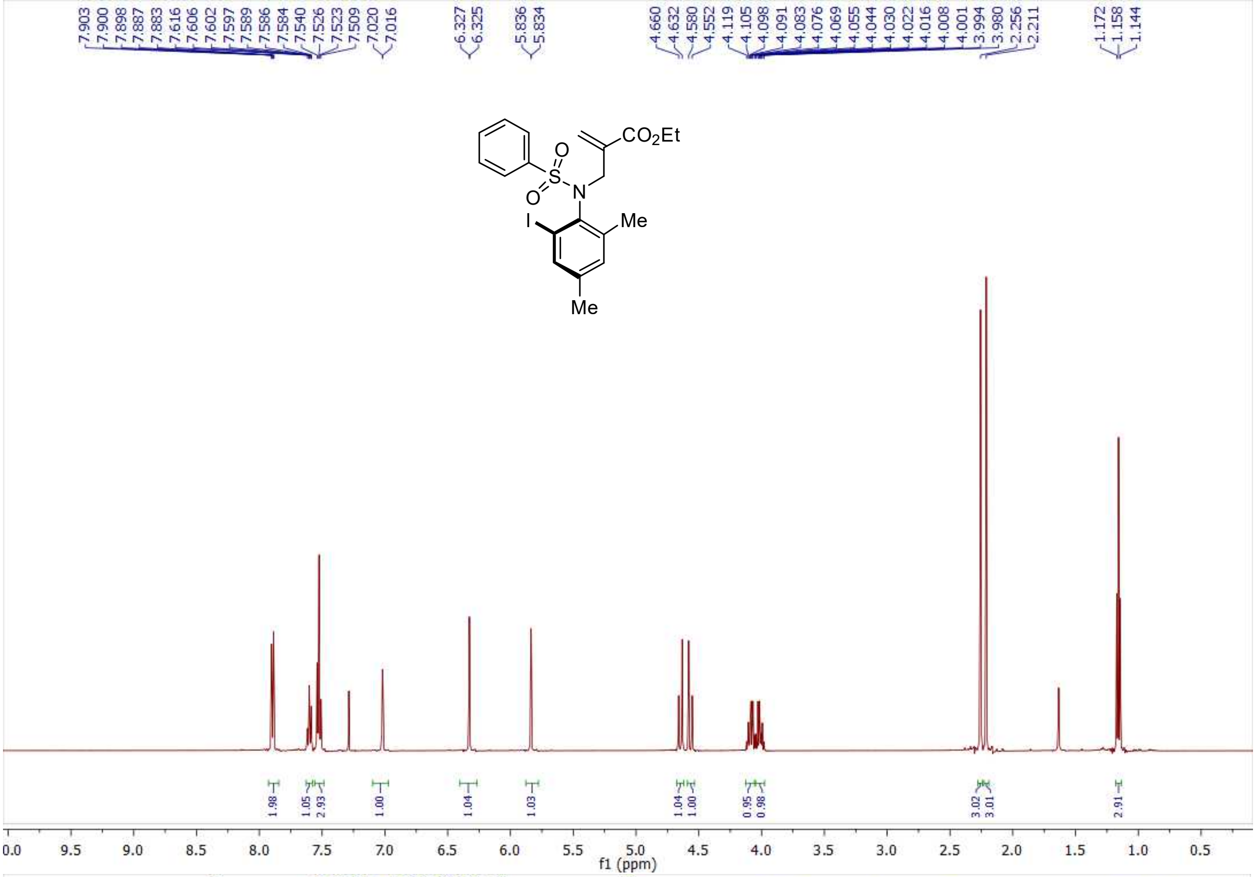

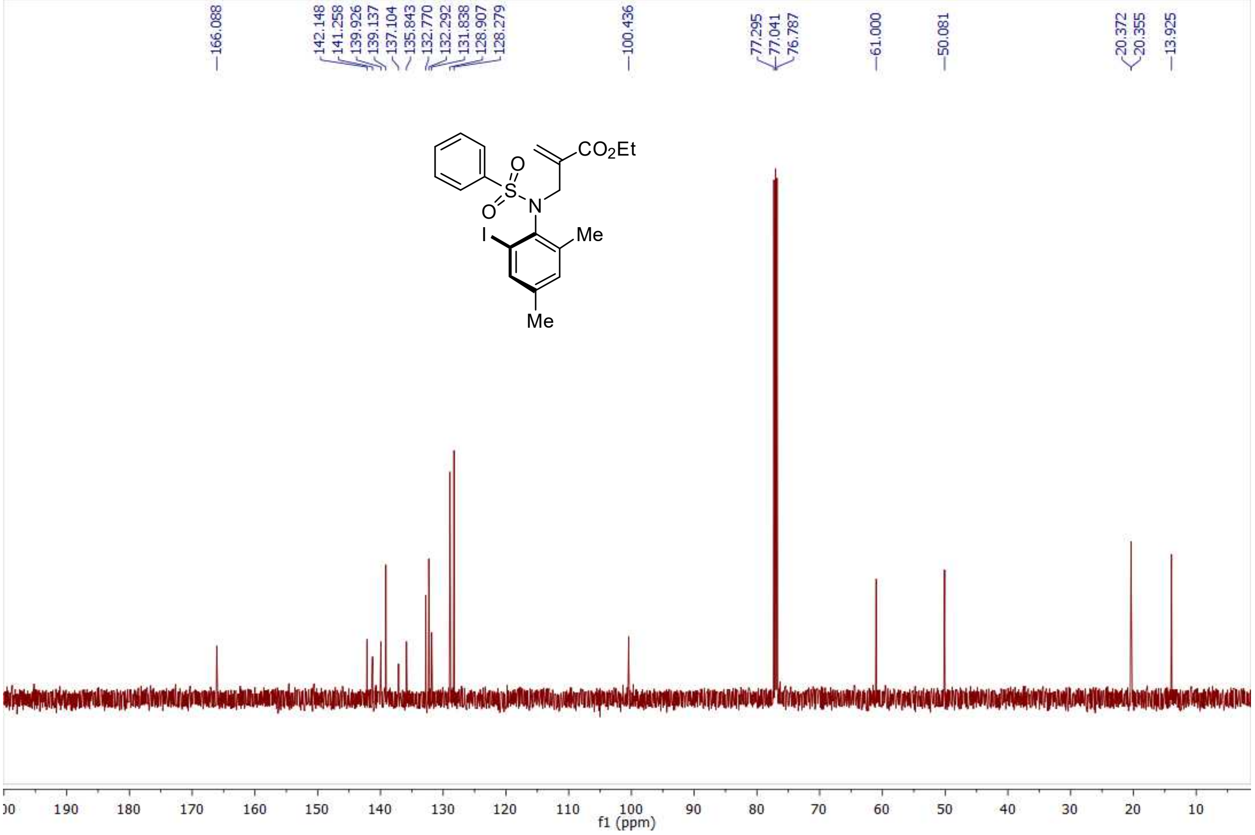

Supplementary Figure 127. ^{1}H and ^{13}C NMR Spectra of **9na**.

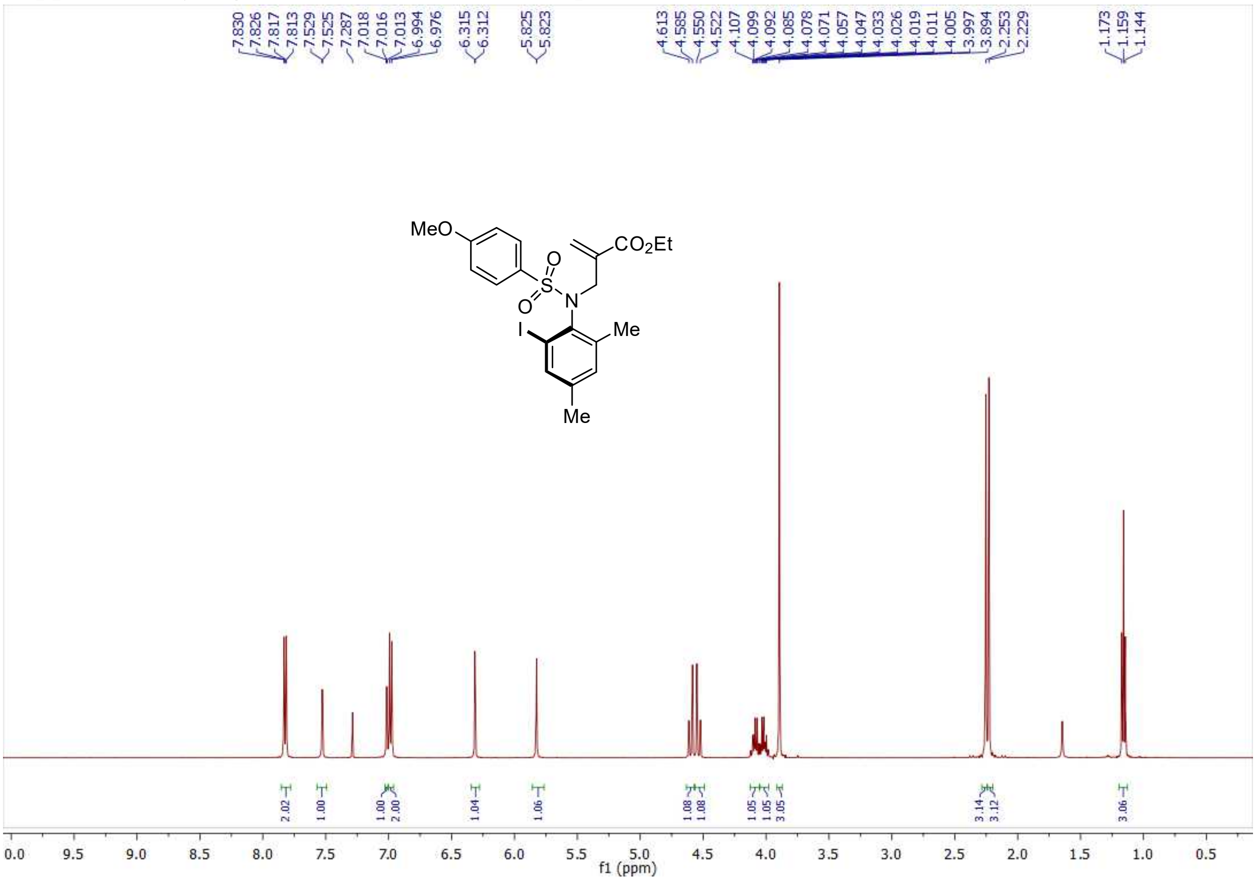

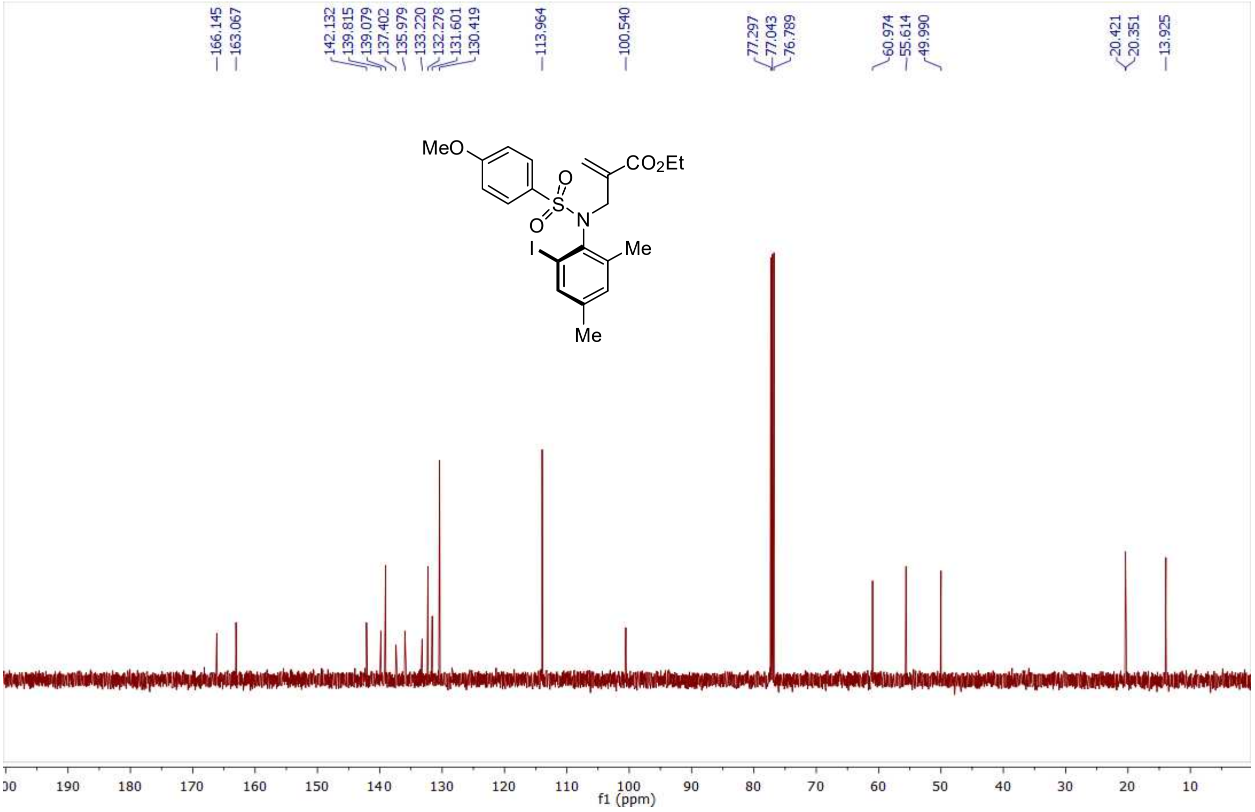

Supplementary Figure 128. ^{1}H and ^{13}C NMR Spectra of **9oa**.

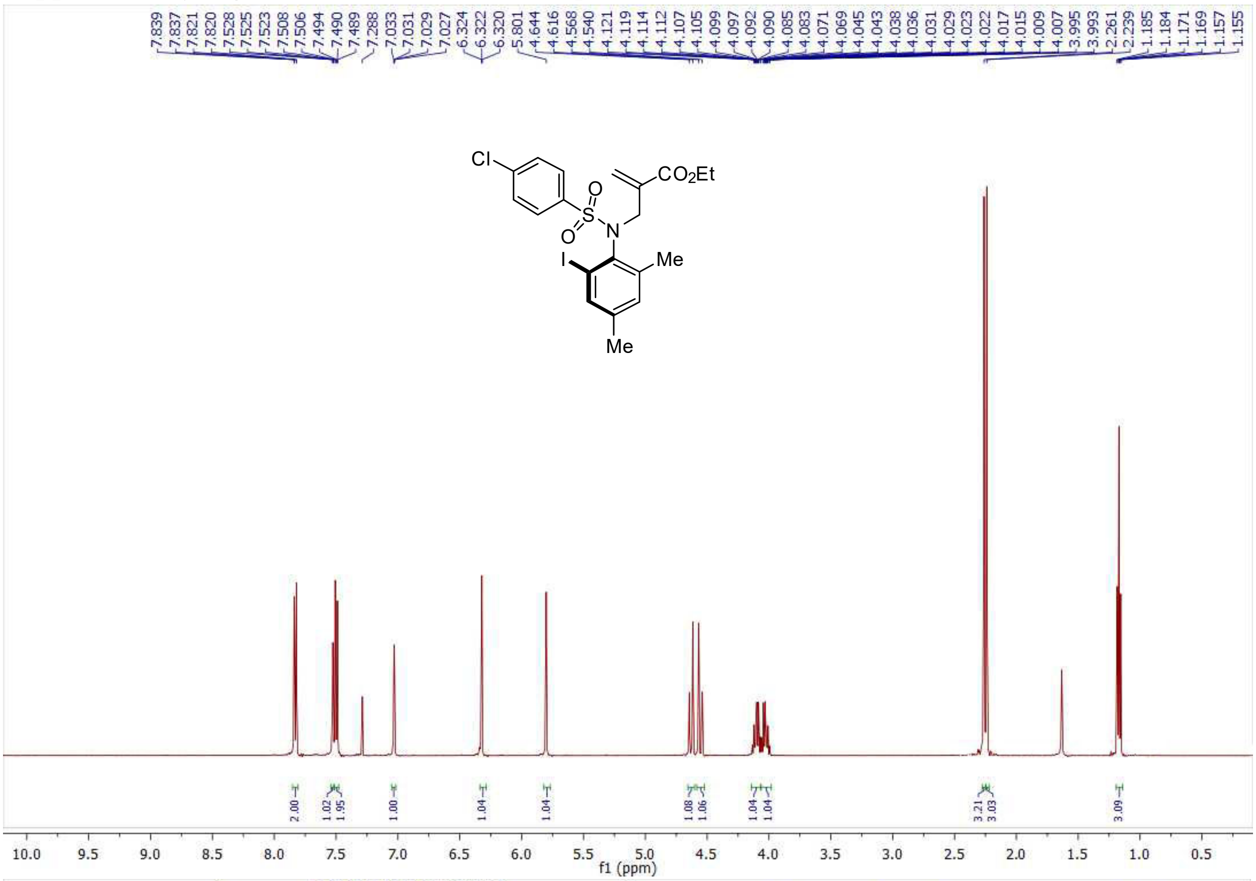

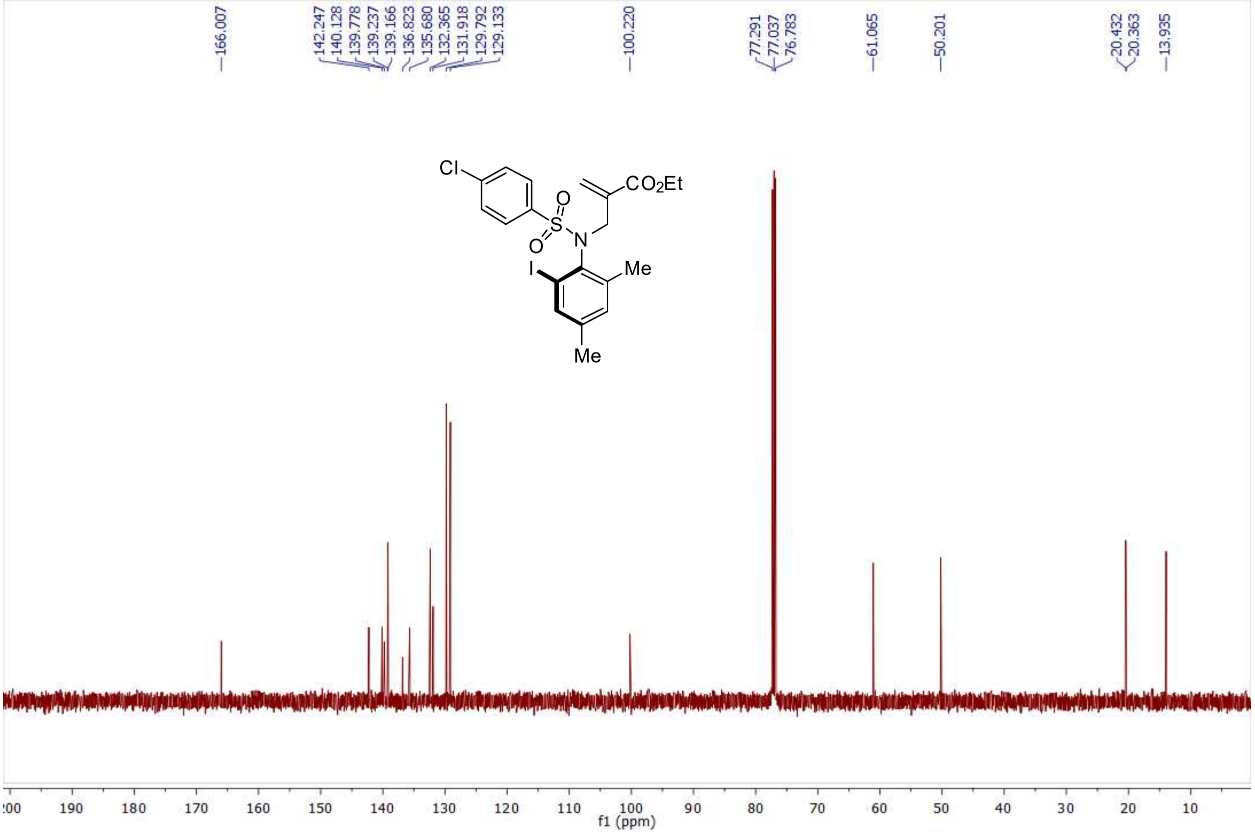

Supplementary Figure 129. ^{1}H and ^{13}C NMR Spectra of **9pa**.

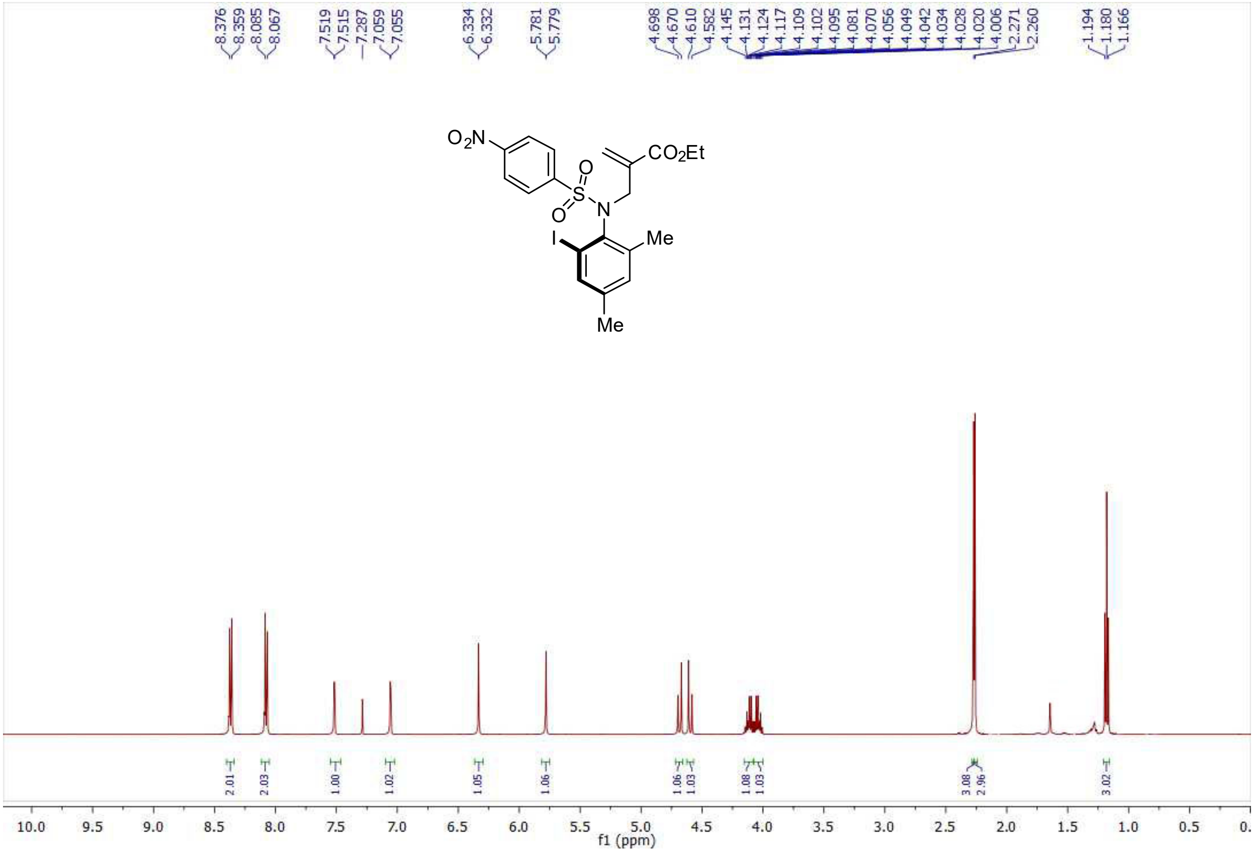

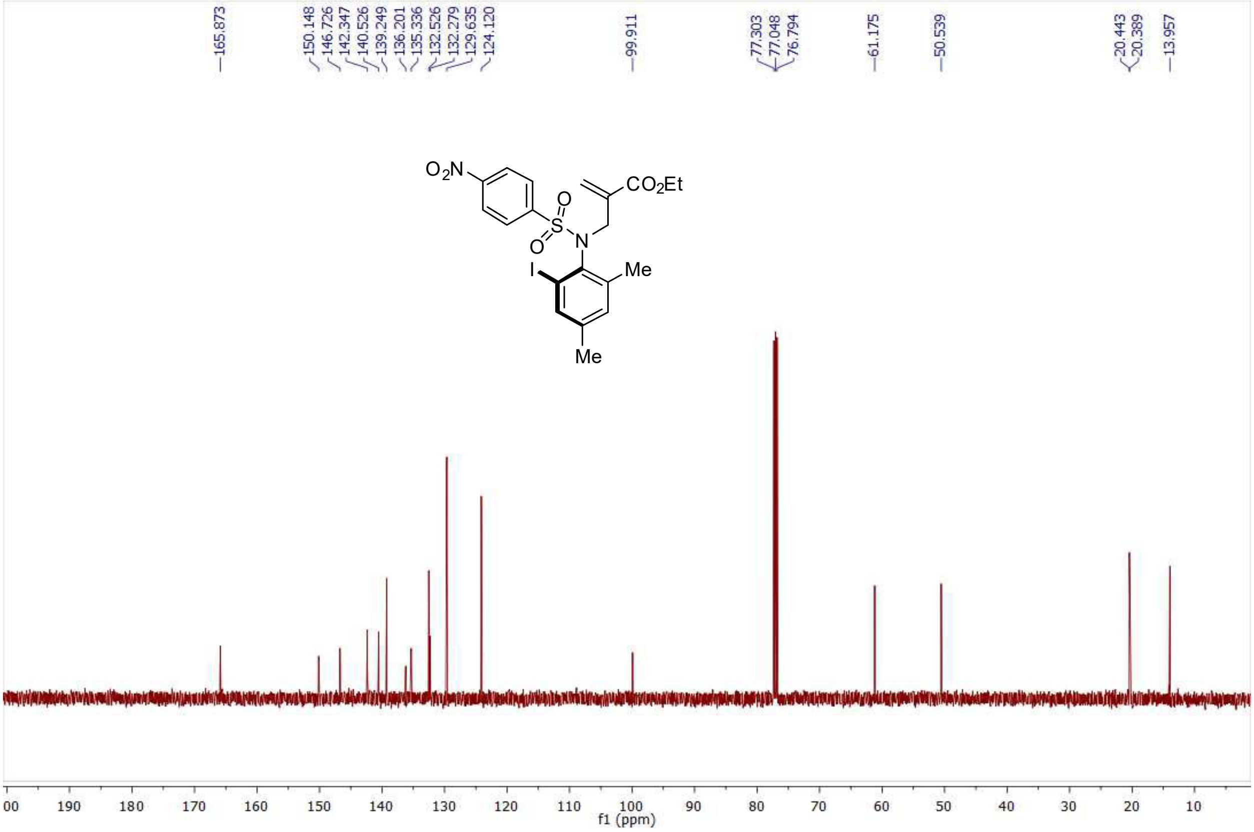

Supplementary Figure 130. ^{1}H and ^{13}C NMR Spectra of **9qa**.

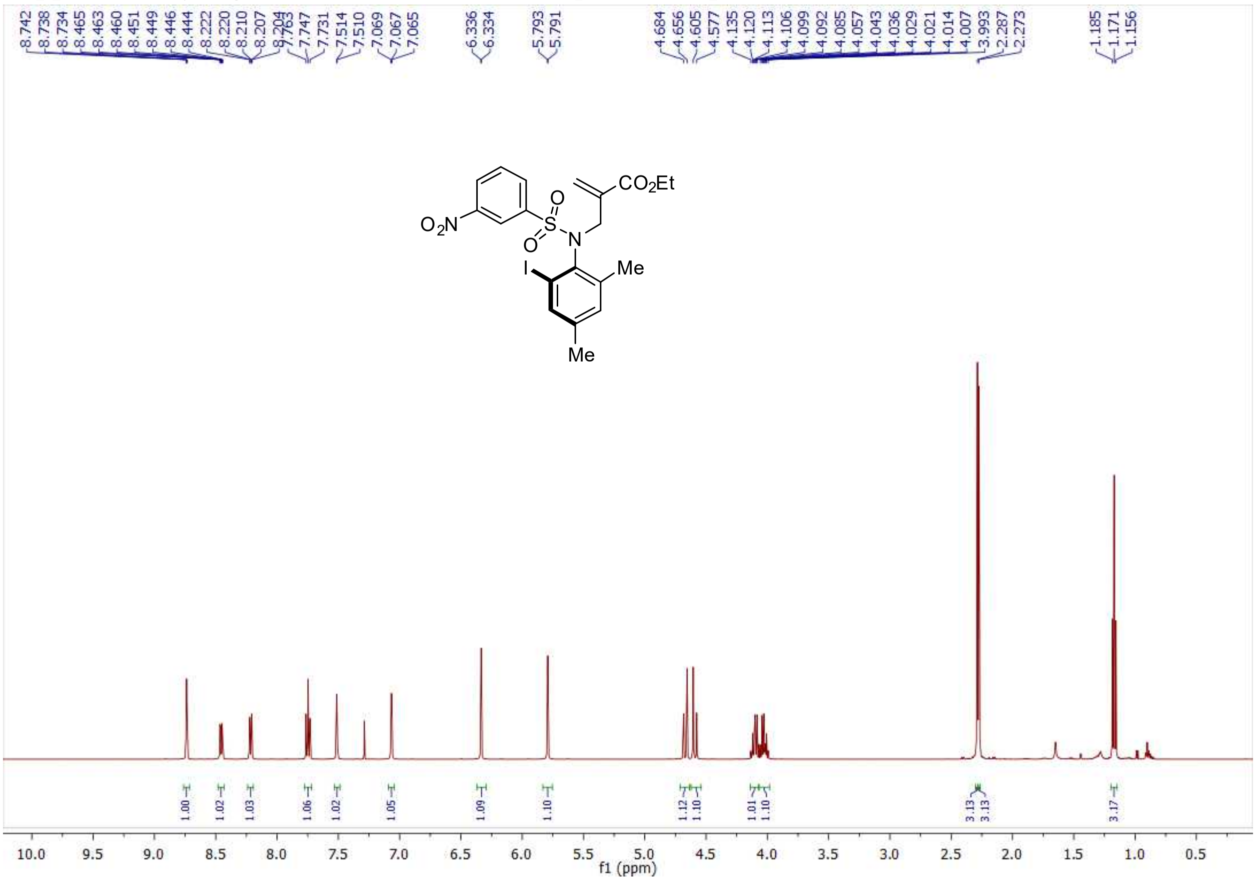

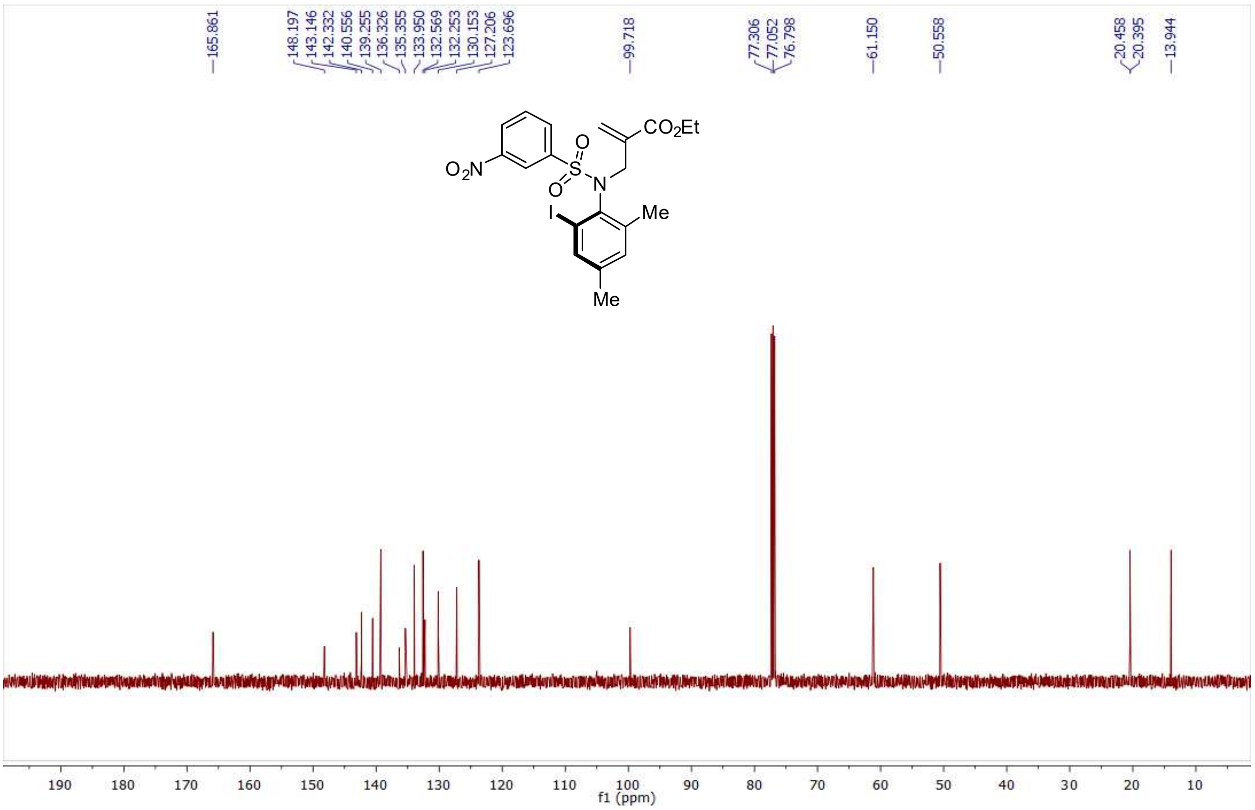

Supplementary Figure 131. ^{1}H and ^{13}C NMR Spectra of **11**.

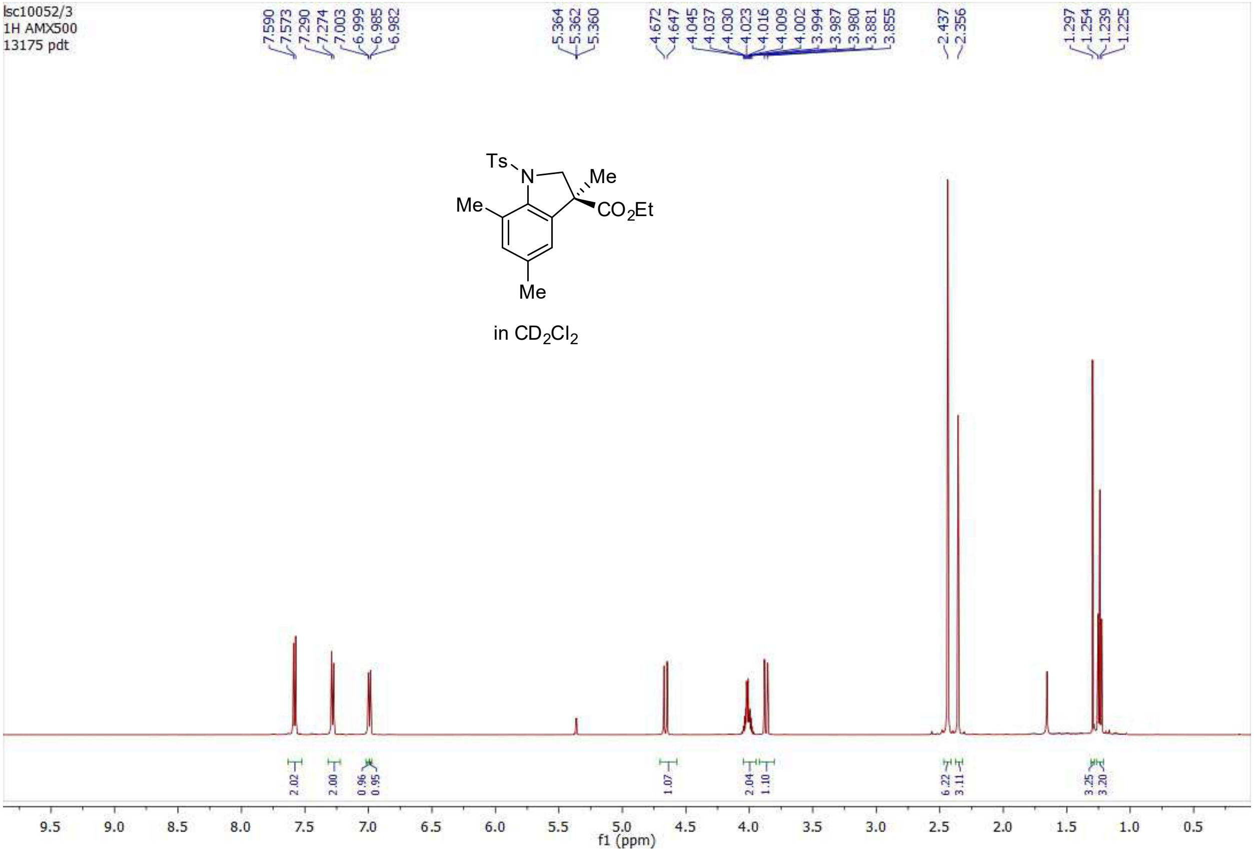

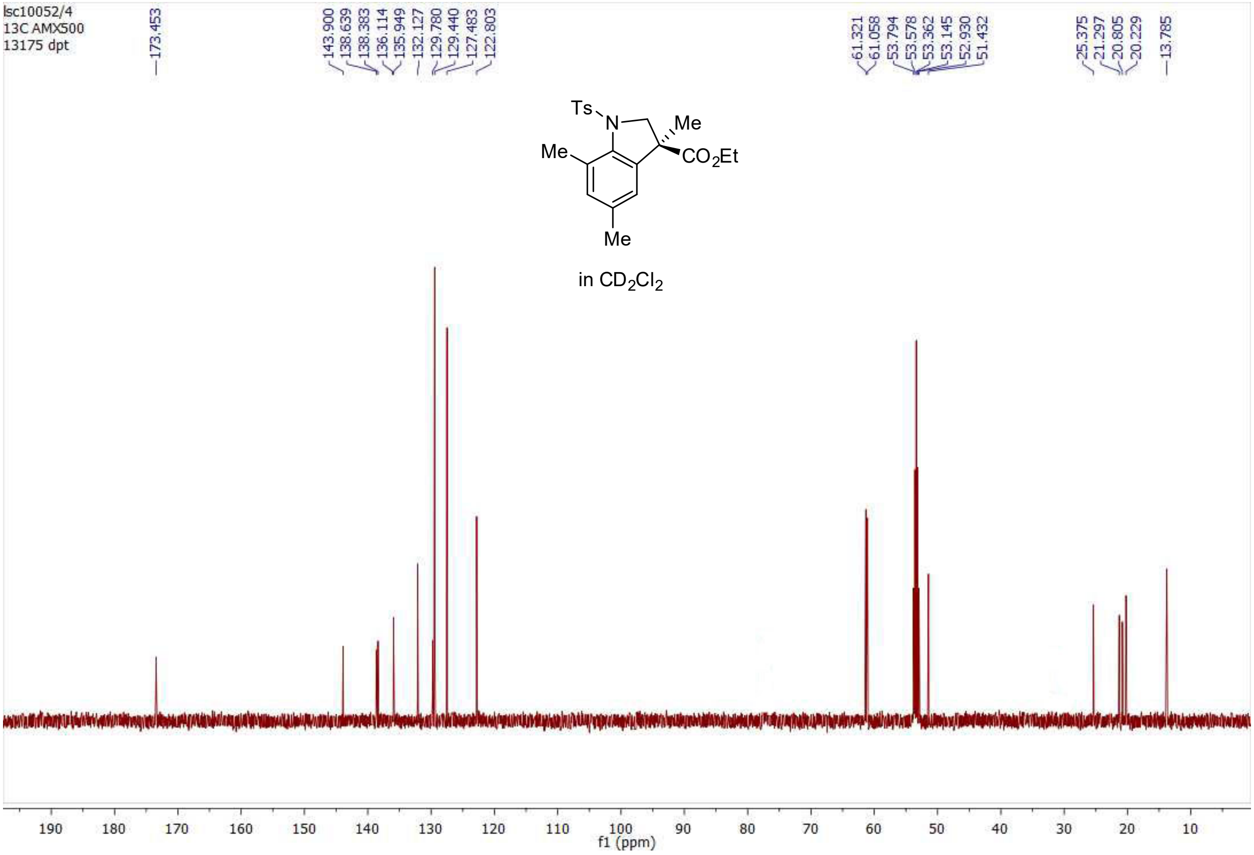

Supplementary Figure 132. ^{1}H and ^{13}C NMR Spectra of **12**.

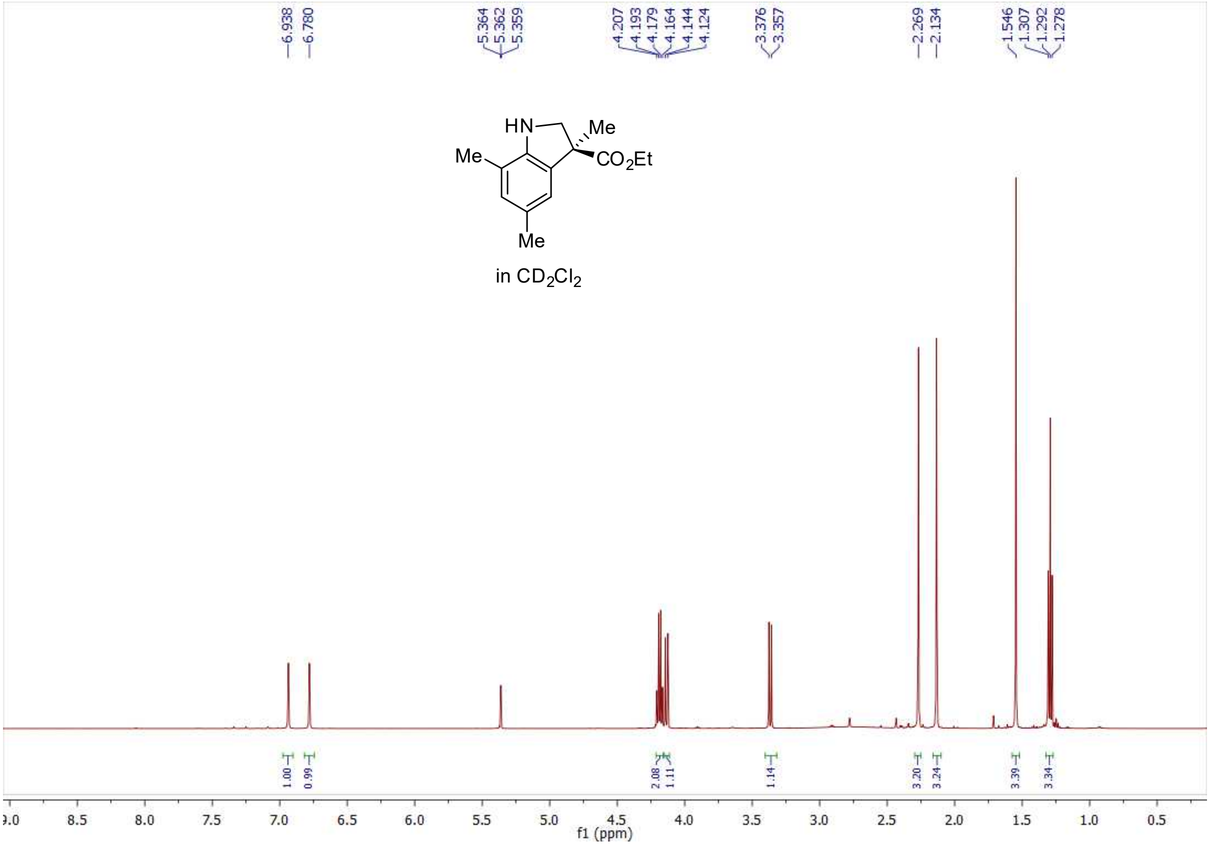

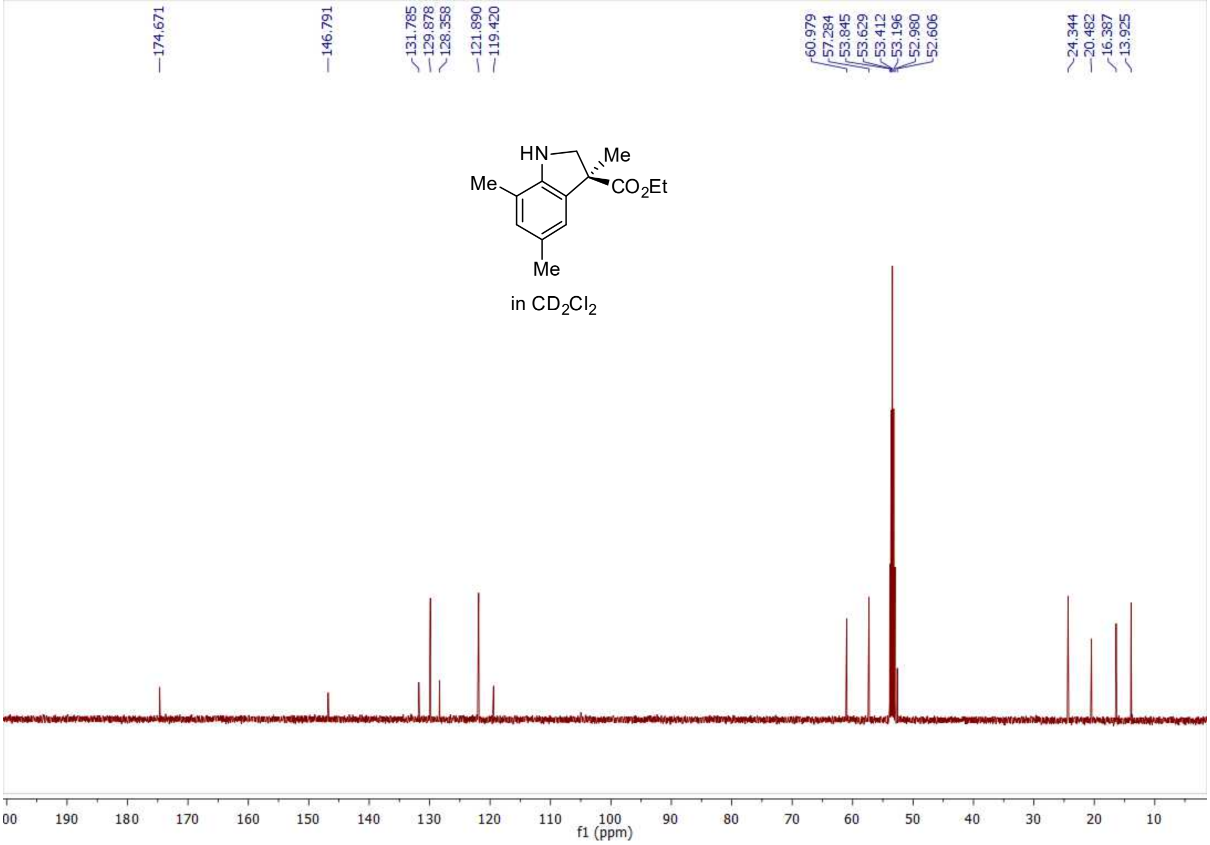

Supplementary Figure 133. ^{1}H and ^{13}C NMR Spectra of **13**.

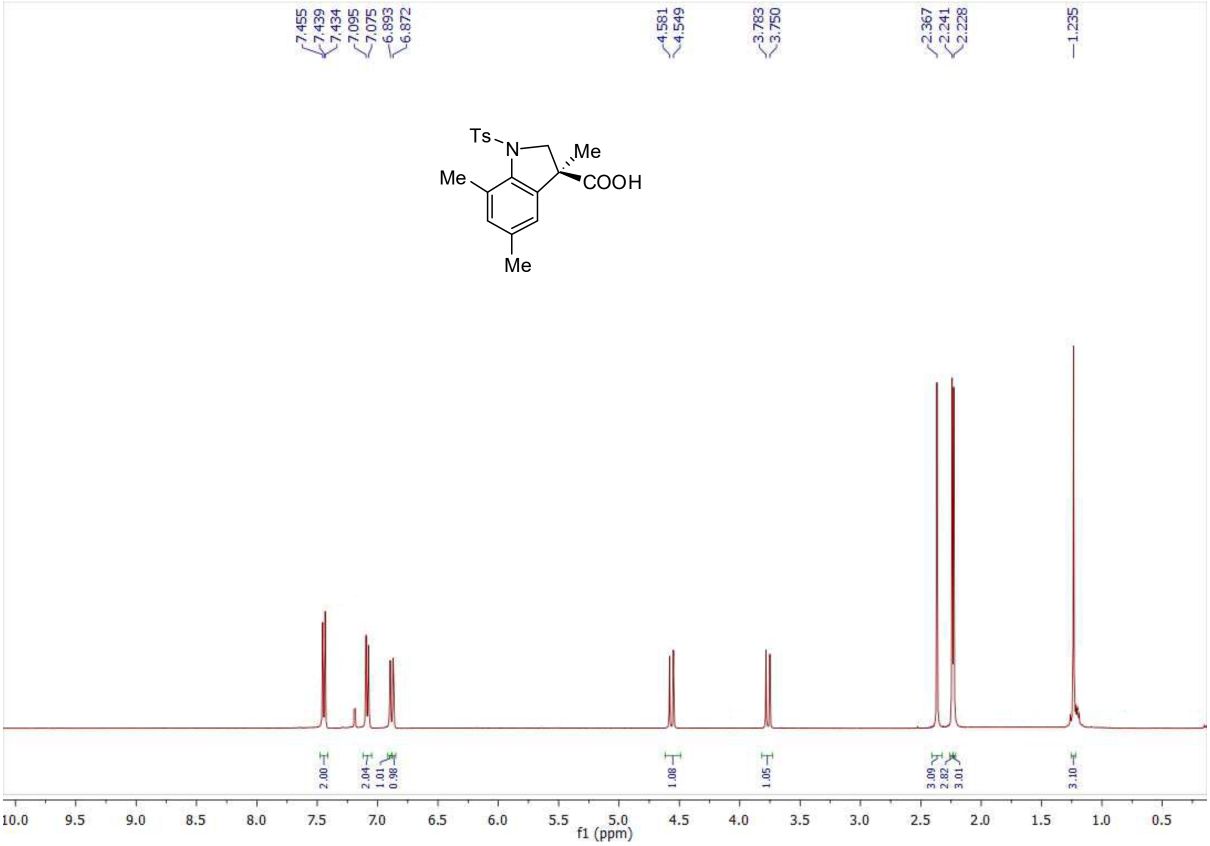

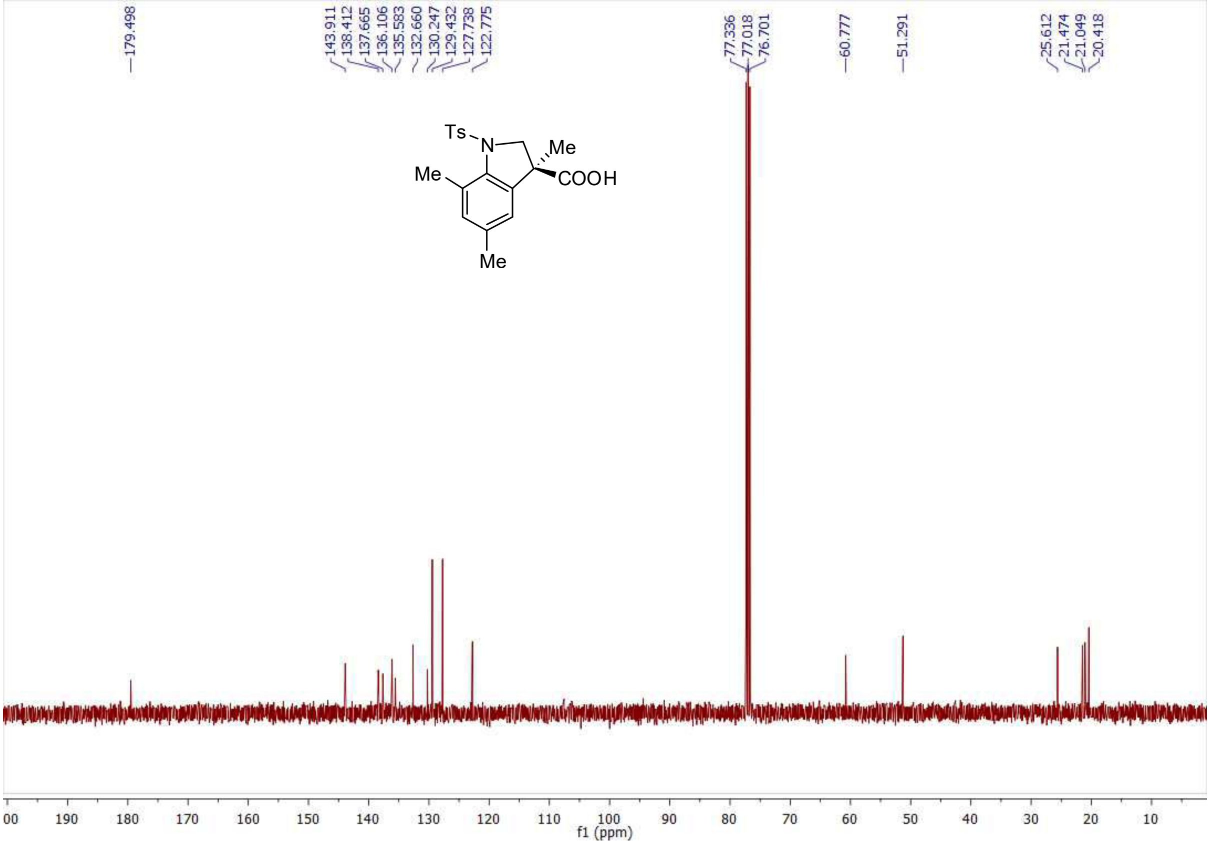

Supplementary References

[1] J. Chen, Y. Huang, *Org. Lett.* **2017**, *19*, 5609-5612.

[2] H. Gao, Q.-L. Xu, C. Keene, M. Yousufuddin, D. H. Ess, L. Kürti, *Angew. Chem. Int. Ed.* **2016**, *55*, 566-571.

[3] H. Gao, D. H. Ess, M. Yousufuddin, L. Kürti, *J. Am. Chem. Soc.* **2013**, *135*, 7086–7089.

[4] R. A. Singer, J. R. Brock, E. M. Carreira, *Helv. Chim. Acta.* **2003**, *86*, 1040–1044.

[5] H. Forkosh, V. Vershinin, H. Reiss, D. Pappo, *Org. Lett.,* **2018**, *20*, 2459-2463.

[6] S. Shirakawa, X. Wu, K. Maruoka, *Angew. Chem. Int. Ed.* **2013**, *52*, 14200–14203

[7] H. Liu, S. Liu, H. Zhou, Q. Liu, C. Wang, *RSC Adv.*, **2018**, *8*, 14829-14832.
